# Organocatalytic, enantioselective synthesis of benzoxaboroles *via* Wittig/oxa-Michael reaction Cascade of α-formyl boronic acids†
†Electronic supplementary information (ESI) available. CCDC 1487136. For ESI and crystallographic data in CIF or other electronic format see DOI: 10.1039/c6sc04522g



**DOI:** 10.1039/c6sc04522g

**Published:** 2017-01-30

**Authors:** Gurupada Hazra, Sanjay Maity, Sudipto Bhowmick, Prasanta Ghorai

**Affiliations:** a Department of Chemistry , Indian Institute of Science Education and Research Bhopal , Bhopal By-pass Road, Bhauri , Bhopal-462066 , India . Email: pghorai@iiserb.ac.in

## Abstract

An unprecedented enantioselective synthesis of 3-substituted benzoxaboroles has been developed. An *in situ* generated *ortho*-boronic acid containing chalcone provides the chiral benzoxaboroles *via* an asymmetric oxa-Michael addition of hydroxyl group attached to the boronic acid triggered by the cinchona alkaloid based chiral amino-squaramide catalysts. In general, good yields with good to excellent enantioselectivities (up to 99%) were obtained. The resulting benzoxaboroles were converted to the corresponding chiral β-hydroxy ketones without affecting the enantioselectivity.

## Abstract 2

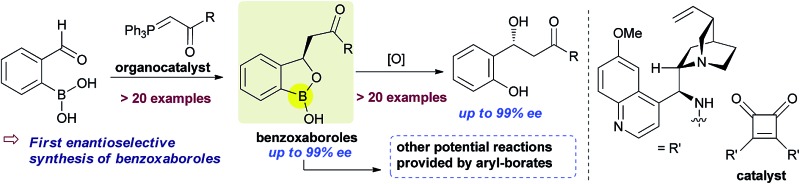
An enantioselective synthesis of 3-substituted benzoxaboroles has been developed using organocatalysts with good to excellent enantioselectivities (up to 99%). The resulting benzoxaboroles were converted to the chiral β-hydroxy ketones without affecting the enantioselectivity.

## Introduction

**Fig. 1:**
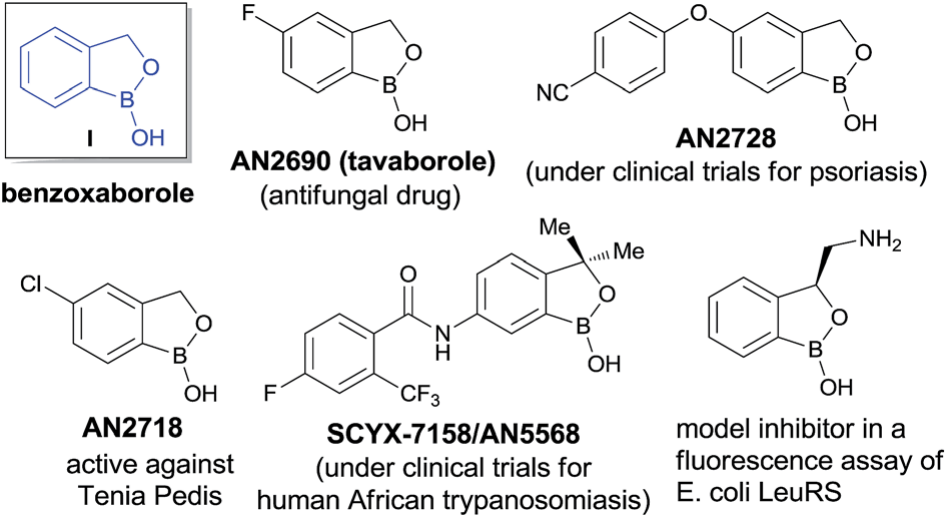
Pharmaceuticals featuring benzoxaborole moiety.

Benzoxaboroles, an important class of boron containing molecules, has recently acquired significant attention towards its applications for the development of new drugs.^1,2^ The Lewis acidity of the boron and its easy conversion from trigonal to tetrahedral geometry enables benzoxaboroles to bind to the active site of various enzymes and thereby inhibit their activity.^1,3^ Ever since the discovery of the exceptional sugar-binding properties at physiological conditions^4^ as well as the finding of antifungal activity of AN2690 (Fig. 1),^5^ benzoxaboroles have been extensively studied for therapeutic applications. This leads to the development of benzoxaboroles with antibacterial,^6^ antiviral,^7^ anti-parasitic,^8^ anti-inflammatory,^9^ and antimalarial^10^ activities as well as β-lactamase inhibitor.^1^

**Scheme 1:**
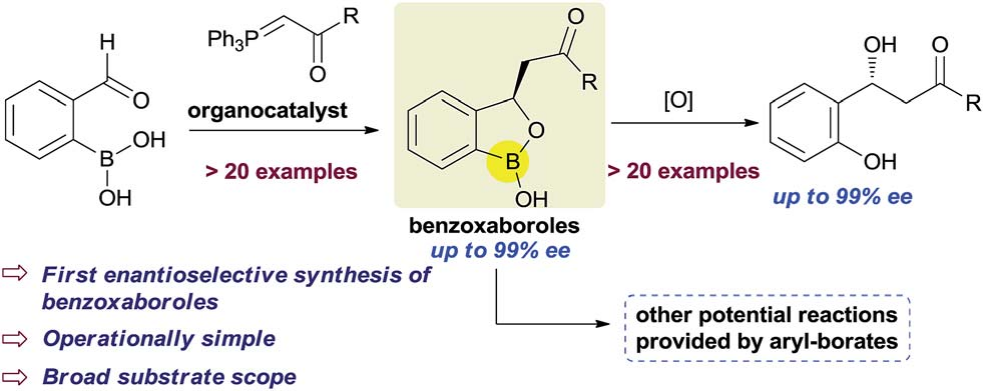
Catalytic enantioselective synthesis of benzoxaboroles.

Very recently, KERYDIN was approved for onychomycosis treatment,^11^ AN2728 (ref. 12) and AN2898 (ref. 13) are currently under clinical trials for psoriasis. SCYX-7158/AN5568 entered into clinical trials for the treatment of human African trypanosomiasis.^14^ Thus, the intrinsic reactivity and metabolic stability of benzoxaborole motif have made it a new “privileged scaffold” for the design of new drugs.^15^ Furthermore, the materials conjugated with benzoxaborole exhibit a unique structural assembly which enables to unfold multidentate interactions to improve selective binding.^16^ This property has extended their applications in supramolecular^17^ and materials chemistry.^18^ Apart from these, benzoxaboroles are versatile building blocks for the synthesis of organic molecules of higher complexity.^19^ Despite the surging applications of benzoxaboroles in various fields and handful attempts directed towards their achiral synthesis, we report herein, for the first time, the catalytic enantioselective synthesis of this class of compounds. In this reaction, the 2-formyl aryl boronic acids react *via* a Wittig reaction followed by an enantioselective oxa-Michael addition of a hydroxyl group attached to the boronic acid using chiral bifunctional organocatalysts (Scheme 1).

Our strategy is leveraged from the asymmetric oxa-Michael addition using bifunctional organocatalyst consisting of squaramide/thiourea moiety attached to a tertiary nitrogen on a chiral scaffold and the oxo-nucleophilicity of the hydroxy group of organoboronic acids, revealed by Falck *et al.* ^20^ We hypothesized to utilize an *in situ* generated *ortho*-boronic acid containing chalcones (II, Table 1) as the substrate wherein the asymmetric oxa-Michael reaction of hydroxy group of boronic acid is involved in the simultaneous coordination of the carbonyl with squaramide/thiourea (the pull) moiety and the tertiary nitrogen to boron (the push).

**Table 1:** Optimization of the reaction conditions^*a*^

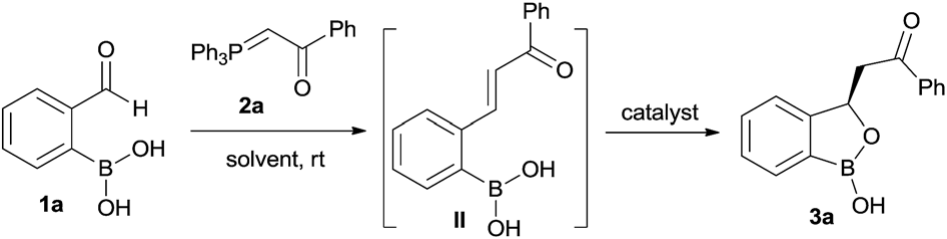				
Entry	Catalyst	Solvent	Yield^*b*^ (%)	(%) ee^*c*^
1	**C_1_**	CH_2_Cl_2_	12	56
2	**C_2_**	CH_2_Cl_2_	<5	ND
3	**C_3_**	CH_2_Cl_2_	26	70
4	**C_4_**	CH_2_Cl_2_	16	–62
5	**C_5_**	CH_2_Cl_2_	26	86
6	**C_6_**	CH_2_Cl_2_	53	90
7	**C_7_**	CH_2_Cl_2_	55	91
8	**C_7_**	Toluene	70	90
9	**C_7_**	PhCF_3_	85	90
10	**C_7_**	THF	15	80
11	**C_7_**	Chlorobenzene	86	91
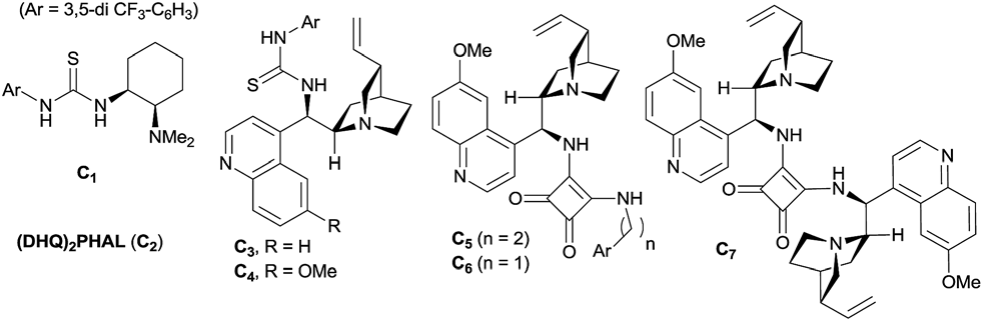				

^*a*^Reactions were performed on a 0.05 mmol scale of aldehyde **1a**, 1.5 equiv. of **2a**.

^*b*^Yield was calculated based on ^1^H NMR spectroscopy of the crude reaction mixture using diphenyl acetonitrile as the internal standard.

^*c*^Enantiomeric excess was determined by HPLC analysis on a chiral stationary phase after oxidative deborylation.

## Results and discussion

**Scheme 2:**
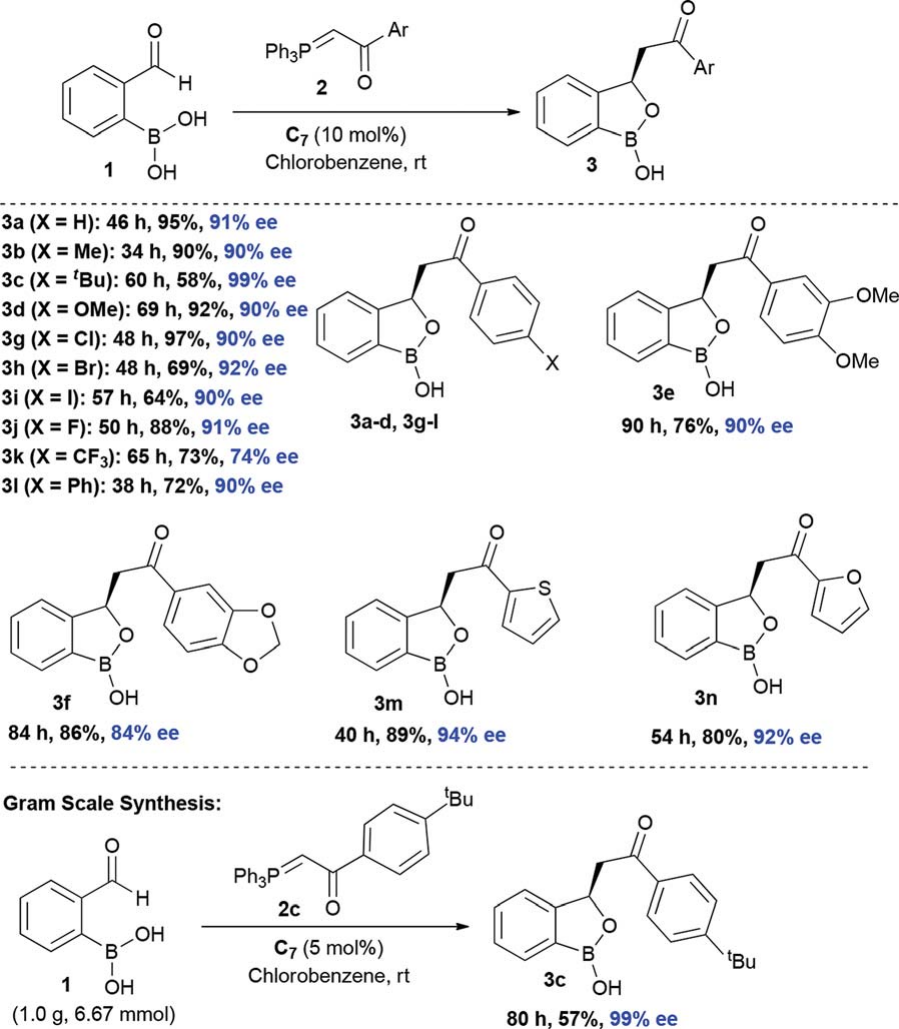
Exploration of the scope with Wittig olefines^a,b,c^. ^a^Reaction conditions: **1** (0.2 mmol), Wittig olefin **2** (0.3 mmol, 1.5 equiv.), **C_7_** (0.02 mmol, 10 mol%). ^b^Enantiomeric excess was determined by chiral HPLC analysis of the corresponding oxidative deborylation product. ^c^All are isolated yields.

**Fig. 2:**
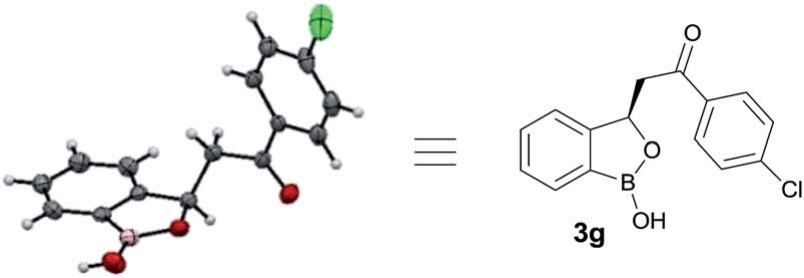
Crystal structure of **3g** (CCDC ; 1487136).

At the outset, we began our investigation with *ortho*-formyl phenyl boronic acid **1a** and benzoyl Wittig olefin **2a** aiming towards the synthesis of chiral benzoxaborole **3a** (Table 1). A variety of chiral amino-thiourea (**C_1_**) and cinchona alkaloid-derived catalysts (**C_2_–C_7_**) were surveyed in dichloromethane at room temperature (entries 1–7) (see ESI†). As shown in entry 7, catalyst **C_7_** was found to catalyze the reaction cleanly to furnish the benzoxaborole **3a** (55% NMR yield with 91% ee). A notable increase in yield was observed when the reaction was performed in toluene and trifluoro-toluene (entry 8 and 9, respectively). An immediate solvent study consoled that chlorobenzene was the optimal one, affording 91% ee with 86% yield (entry 11). Under these optimal reaction conditions, we examined the substrate scope of this reaction, and the results are shown in Scheme 2. The effect of substitution on the aryl moiety of Wittig-olefins was first examined. To our delight, electron-donating substituents such as Me– (**3b**), ^*t*^Bu– (**3c**) and MeO– (**3d–e**) underwent smooth cyclization, affording the desired products in good to excellent yields and with excellent enantioselectivities (58–95% yields, 84–99% ee). Similarly, electron-withdrawing substituents such as Cl– (**3g**), Br– (**3h**), I– (**3i**), F– (**3j**), F_3_C– (**3k**) and Ph– (**3l**), also furnished the desired products with high stereo-induction (64–97% yields, 74–92% ee). Hetero-aromatic groups such as 2-thiophenyl as well as 2-furyl were also well tolerated to provide the corresponding benzoxaboroles **3m** (89% yield, 94% ee) and **3n** (80% yield, 92% ee), respectively. To illustrate the practical utility of this methodology, a gram scale (1.0 g, 6.67 mmol) reaction was performed to provide the product **3c**. Interestingly, it was observed that even 5 mol% of catalyst was enough to complete the reaction without losing any enantioselectivity (57% yield, 99% ee). The absolute stereochemistry (*R*) of the product **3g** was determined by X-ray crystallography (Fig. 2) and the other compounds were assigned by analogy.

**Scheme 3:**
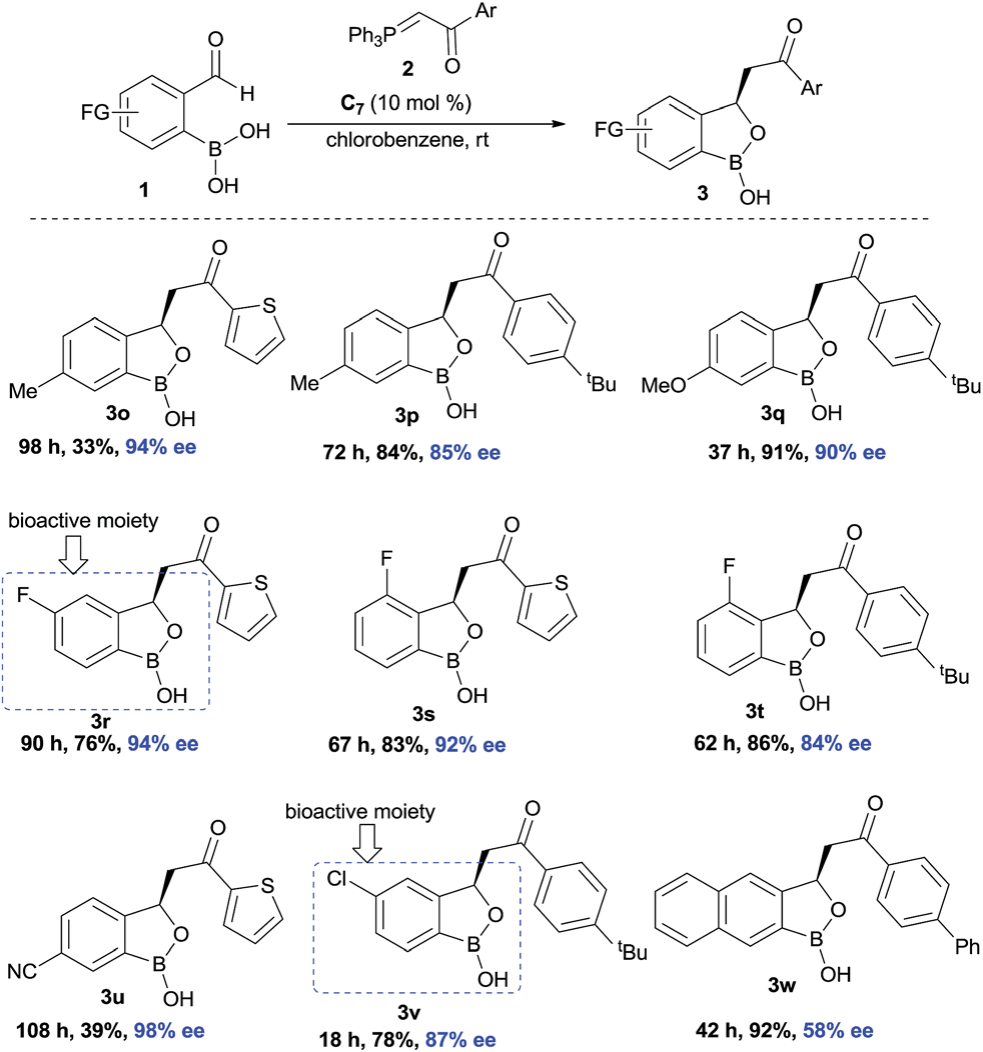
Exploration of the scope with *o*-formyl aryl boronic acids^a,b,c^. ^a^Reaction conditions: **1** (0.2 mmol), Wittig olefin **2** (0.3 mmol, 1.5 equiv.), **C_7_** (0.02 mmol, 10 mol%). ^b^Enantiomeric excess was determined by chiral HPLC analysis of the corresponding oxidative deborylation product. ^c^All are isolated yields.

Next, the substitution on the central aryl moiety was examined which was quite general (Scheme 3) concerning enantioselectivities as well as yields. Electron-donating substituents such as Me– (**3o–p**), MeO– (**3q**), as well as electron-withdrawing substituents such as F– (**3r–t**), CN– (**3u**) and Cl– (**3v**), worked smoothly, affording the desired benzoxaborole with excellent enantioselectivities. Instead of central phenyl moiety, naphthyl moiety (**3w**) remained less efficient regarding enantioselectivity. Notably, the F- and Cl-substituted (**3r** and **3v**) benzoxaborole moieties are known to have bioactivity as mentioned in Fig. 1.

**Scheme 4:**
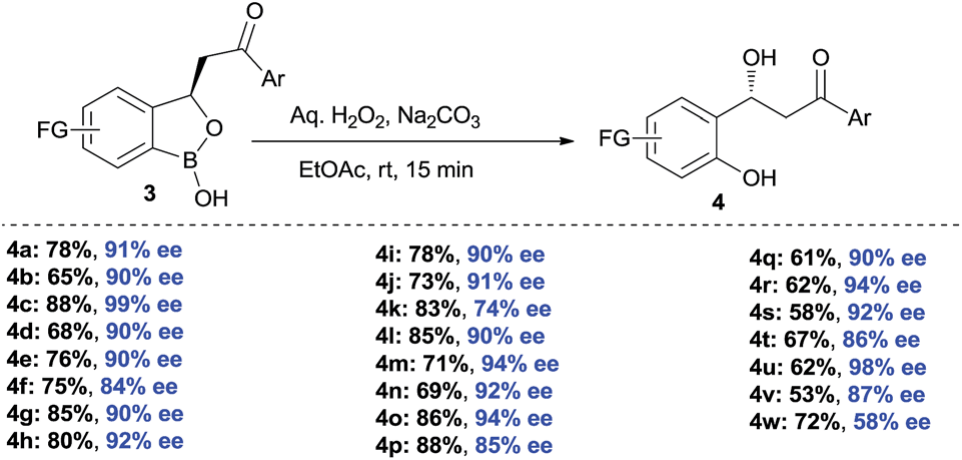
Synthesis of chiral β-hydroxy ketones^a,b,c^. ^a^Reaction conditions: **3** (0.1 mmol), Aq. H_2_O_2_ (0.1 mL), Aq. Na_2_CO_3_ (1 mL). ^b^Enantiomeric excess was determined by HPLC analysis on a chiral stationary phase. ^c^All are isolated yields.

The synthesis of chiral β-hydroxy carbonyls has remained an attractive area of research because of their prevalence in organic synthesis and medicinal chemistry.^21^ Chiral benzylic alcohol containing phenols were used as chiral precursor for asymmetric synthesis, ligand and chiral auxiliary in asymmetric catalysis.^22^ However, there are limited methods in literature for the synthesis of such chiral alcohols.^23^ Moreover, synthesis of chiral β-hydroxy ketones has remained elusive so far. Therefore, we also emphasized on the oxidative deborylation of the products **3** (Scheme 4). Interestingly, the corresponding chiral benzyl alcohol **4** was obtained smoothly after oxidative deborylation of **3** with high yields and excellent enantioselectivities. Thus, *via* this benzoxaborolane pathway, the synthesis of β-hydroxy ketones shows a considerable advantage with respect to enantioselectivity and substrate generality.

**Scheme 5:**
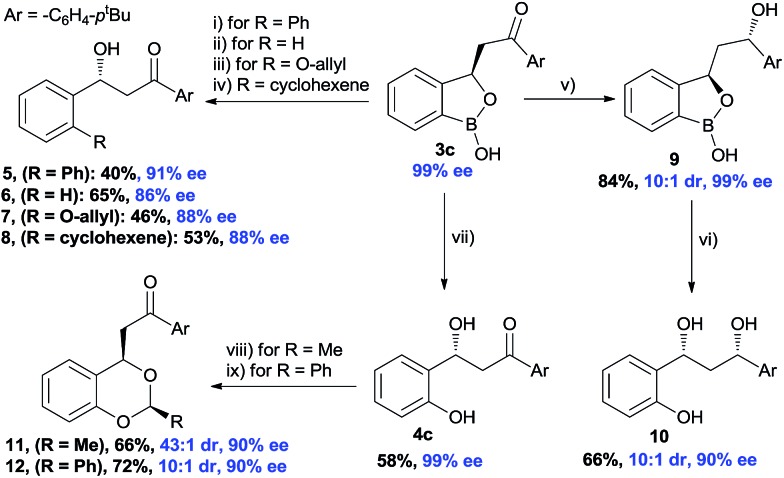
Functional group transformation of the product^a^. Reaction conditions: (i) PhBr (1.1 equiv.), Pd(PPh_3_)_4_ (2 mol%), K_2_CO_3_ (2 equiv.), dry dioxane, 80 °C. (ii) AgNO_3_ (10 mol%), Et_3_N (0.1 equiv.), EtOH : H_2_O (1 : 1), rt. (iii) Allyl alcohol, Cu(OAc)_2_ (2 equiv.), Et_3_N (4 equiv.), rt. (iv) Cyclohexenyl trifluoromethanesulfonate (1.2 equiv.), Pd(dppf)Cl_2_ (0.1 equiv.), DME, EtOH, Na_2_CO_3_ 80 °C. (v) NaBH_4_ (1.1 equiv.), MeOH, –5 °C to –10 °C. (vi) Pd(PPh_3_)_4_ (2 mol%), K_2_CO_3_ (20 mol%), 1,4-dioxane, 80 °C. (vii) Aq. H_2_O_2_, NaHCO_3_, EtOAc, rt. (viii) CH_3_CHO (1.2 equiv.), PTSA (20 mol%), CH_2_Cl_2_, rt. (ix) PhCH(OMe)_2_ (1.2 equiv.), PTSA (20 mol%), CH_2_Cl_2_, rt. ^a^All are isolated yields.

The synthetic potential of the benzoxaborolanes was briefly investigated using oxaborol **3** (Scheme 5). Subjecting **3c** to a Pd-catalyzed Suzuki reaction conditions using bromobenzene afforded product **5** in 40% yield and 91% ee. Similarly, Ag-catalyzed deborylation provided the compound **6** and Cu-catalyzed deborylation in the presence of allyl alcohol produced **7** with high ee. A palladium catalyzed olefination with alkenyl triflate furnished the desired **8** with 88% ee. A substantial stereo-induction was observed during the NaBH_4_ reduction of keto functionality of **3c**, an excellent diastereoselectivity (10 : 1) was achieved in the compound **9**. Further, oxidative deborylation of the product **9** produced chiral 1,3-diol^24^ **10** with high enantioselectivity and good diastereoselectivity. Finally, chiral benzylic alcohol **4c** was treated with aldehydes in the presence of acids which furnished the acetals (**11** and **12**) with excellent diastereoselectivity (43 : 1 and 10 : 1, respectively), albeit a small reduction of enantioselectivity.

To explain the observed absolute stereochemical outcome a bifunctional mechanism similar to those previously proposed for the squaramide/thiourea-catalyzed oxa-Michael reaction of enones may be invoked.^25^

## Conclusions

In summary, a sequential Wittig olefination followed by an enantioselective intramolecular oxa-Michael reaction of *ortho*-boronic acid containing chalcones have been developed using chiral bifunctional organocatalysts. This process provides the very first and promising approach for the synthesis of benzoxaboroles with excellent enantioselectivities and with a broad substrate scope. The resulting products were converted to the corresponding chiral β-hydroxy ketones without affecting the enantioselectivity.
