# Supplementary material for: Organocatalytic, enantioselective synthesis of benzoxaboroles via Wittig/oxa-Michael reaction Cascade of α-formyl boronic acids
Source: Chem Sci. 2017 Jan 30;8(4):3026–30. doi: 10.1039/c6sc04522g (PMC5380879; doi:10.1039/c6sc04522g)

Electronic Supplementary Material (ESI) for Chemical Science.
This journal is © The Royal Society of Chemistry 2017

Organocatalytic, Enantioselective Synthesis of Benzoxaboroles *via* Wittig / oxa-Michael Reaction Cascade of α-Formyl Boronic Acids.

Gurupada Hazra, Sanjay Maity, Sudipto Bhoumik, and Prasanta Ghorai*

Department of Chemistry, Indian Institute of Science Education and Research (IISER) Bhopal, Bhopal By-pass Road, Bhouri, Bhopal-462066, India.

E-mail: pghorai@iiserb.ac.in.

Supporting Information - I: *Experimental Procedures and Characterization*

Contents: **Pages**

General Experimental Procedures 4

Materials 4

Synthesis of benzoxaboroles: optimization of the reaction conditions: 5

Representative synthetic procedure for chiral benzoxaboroles 6

2-(1-Hydroxy-1,3-dihydrobenzo[c][1,2]oxaborol-3-yl)-1-phenylethan-1-one (**3a**) 6

2-(1-Hydroxy-1,3-dihydrobenzo[c][1,2]oxaborol-3-yl)-1-(*p*-tolyl)ethan-1-one (**3b**) 6

1-(4-(*tert*-Butyl)phenyl)-2-(1-hydroxy-1,3-dihydrobenzo[c][1,2]oxaborol-3-yl)ethan-1-one (**3c**) 7

2-(1-Hydroxy-1,3-dihydrobenzo[c][1,2]oxaborol-3-yl)-1-(4-methoxyphenyl)ethan-1-one (**3d**) 7

1-(3,4-Dimethoxyphenyl)2-(1-hydroxy-1,3-dihydrobenzo[c][1,2]oxaborol-3-yl)ethan-1-one (**3e**) 7

1-(Benzo[*d*][1,3]dioxol-5-yl)-2-(1-hydroxy-1,3-dihydrobenzo[c][1,2]oxaborol-3-yl)ethan-1-one (**3f**) 8

1-(4-Chlorophenyl)-2-(1-hydroxy-1,3-dihydrobenzo[c][1,2]oxaborol-3-yl)ethan-1-one (**3g**) 8

1-(4-Bromophenyl)-2-(1-hydroxy-1,3-dihydrobenzo[c][1,2]oxaborol-3-yl)ethan-1-one (**3h**) 8

2-(1-Hydroxy-1,3-dihydrobenzo[c][1,2]oxaborol-3-yl)-1-(4-iodophenyl)ethan-1-one (**3i**) 9

1-(4-Fluorophenyl)-2-(1-hydroxy-1,3-dihydrobenzo[c][1,2]oxaborol-3-yl)ethan-1-one (**3j**) 9

2-(1-Hydroxy-1,3-dihydrobenzo[c][1,2]oxaborol-3-yl)-1-(4-(trifluoromethyl)phenyl)ethan-1-one) (**3k**) 9

1-([1,1'-Biphenyl]-4-yl)-2-(1-hydroxy-1,3-dihydrobenzo[c][1,2]oxaborol-3-yl)ethan-1-one (**3l**) 10

2-(1-Hydroxy-1,3-dihydrobenzo[c][1,2]oxaborol-3-yl)-1-(thiophen-2-yl)ethan-1-one (**3m**) 10

1-(Furan-2-yl)-2-(1-hydroxy-1,3-dihydrobenzo[c][1,2]oxaborol-3-yl)ethan-1-one (**3n**) 10

2-(1-Hydroxy-6-methyl-1,3-dihydrobenzo[c][1,2]oxaborol-3-yl)-1-(thiophen-2-yl)ethan-1-one (**3o**) 11

1-(4-(*tert*-butyl)phenyl)-2-(1-hydroxy-6-methyl-1,3-dihydrobenzo[c][1,2]oxaborol-3-yl)ethan-1-one (**3p**) 11

1-(4-(tert-Butyl)phenyl)-2-(1-hydroxy-6-methoxy-1,3-dihydrobenzo[c][1,2]oxaborol-3-yl)ethan-1-one (**3q**)

2-(5-Fluoro-1-hydroxy-1,3-dihydrobenzo[c][1,2]oxaborol-3-yl)-1-(thiophen-2-yl)ethan-1-one (**3r**) 12

2-(4-Fluoro-1-hydroxy-1,3-dihydrobenzo[c][1,2]oxaborol-3-yl)-1-(thiophen-2-yl)ethan-1-one (**3s**) 12

1-(4-(*tert*-Butyl)phenyl)-2-(4-fluoro-1-hydroxy-1,3-dihydrobenzo[c][1,2]oxaborol-3-yl)ethan-1-one (**3t**) 12

1-Hydroxy-3-(2-oxo-2-(thiophen-2-yl)ethyl)-1,3-dihydrobenzo[c][1,2]oxaborole-6-carbonitrile (**3u**) 13
1-(4-(*tert*-Butyl)phenyl)-2-(5-chloro-1-hydroxy-1,3-dihydrobenzo[c][1,2]oxaborol-3-yl)ethan-1-one (**3v**) 13
1-([1,1'-Biphenyl]-4-yl)-2-(1-hydroxy-1,3-dihydronaphtho[2,3-c][1,2]oxaborol-3-yl)ethan-1-one (**3w**) 13
Representative synthetic procedure for β-hydroxy ketone 14
3-Hydroxy-3-(2-hydroxyphenyl)-1-phenylpropan-1-one (**4a**) 14
3-Hydroxy-3-(2-hydroxyphenyl)-1-(p-tolyl)propan-1-one (**4b**) 15
1-(4-(*tert*-Butyl)phenyl)-3-hydroxy-3-(2-hydroxyphenyl)propan-1-one (**4c**) 15
3-Hydroxy-3-(2-hydroxyphenyl)-1-(4-methoxyphenyl)propan-1-one (**4d**) 15
1-(3,4-Dimethoxyphenyl)-3-hydroxy-3-(2-hydroxyphenyl)propan-1-one (**4e**) 16
1-(Benzo[d][1,3]dioxol-5-yl)-3-hydroxy-3-(2-hydroxyphenyl)propan-1-one (**4f**) 16
1-(4-Chlorophenyl)-3-hydroxy-3-(2-hydroxyphenyl)propan-1-one (**4g**) 17
1-(4-Bromophenyl)-3-hydroxy-3-(2-hydroxyphenyl)propan-1-one (**4h**) 17
3-Hydroxy-3-(2-hydroxyphenyl)-1-(4-iodophenyl)propan-1-one (**4i**) 17
1-(4-Fluorophenyl)-3-hydroxy-3-(2-hydroxyphenyl)propan-1-one (**4j**) 18
3-Hydroxy-3-(2-hydroxyphenyl)-1-(4-(trifluoromethyl)phenyl)propan-1-one (**4k**) 18
1-([1,1'-Biphenyl]-4-yl)-3-hydroxy-3-(2-hydroxyphenyl)propan-1-oneone (**4l**) 19
3-Hydroxy-3-(2-hydroxyphenyl)-1-(thiophen-2-yl)propan-1-one (**4m**) 19
1-(Furan-2-yl)-3-hydroxy-3-(2-hydroxyphenyl)propan-1-one (**4n**) 19
3-Hydroxy-3-(2-hydroxy-4-methylphenyl)-1-(thiophen-2-yl)propan-1-one (**4o**) 20
1-(4-(*tert*-Butyl)phenyl)-3-hydroxy-3-(2-hydroxy-4-methylphenyl)propan-1-one (**4p**) 20
1-(4-(*tert*-Butyl)phenyl)-3-hydroxy-3-(2-hydroxy-4-methoxyphenyl)propan-1-one (**4q**) 21
3-(5-Fluoro-2-hydroxyphenyl)-3-hydroxy-1-(thiophen-2-yl)propan-1-one (**4r**) 21
3-(2-Fluoro-6-hydroxyphenyl)-3-hydroxy-1-(thiophen-2-yl)propan-1-one (**4s**) 21
1-(4-(*tert*-Butyl)phenyl)-3-(2-fluoro-6-hydroxyphenyl)-3-hydroxypropan-1-one (**4t**) 22
3-Hydroxy-4-(1-hydroxy-3-oxo-3-(thiophen-2-yl)propyl)benzonitrile (**4u**) 22
1-(4-(*tert*-Butyl)phenyl)-3-(5-chloro-2-hydroxyphenyl)-3-hydroxypropan-1-one (**4v**) 23
1-([1,1'-Biphenyl]-4-yl)-3-hydroxy-3-(3-hydroxynaphthalen-2-yl)propan-1-one (**4w**) 23
Procedure for deborylation-phenylation of compound **3c** ` 24
3-([1,1'-Biphenyl]-2-yl)-1-(4-(tert-butyl)phenyl)-3-hydroxypropan-1-one (**5**) 24
Procedure for deborylation-protonation of compound **3c** 24
1-(4-(*tert*-Butyl)phenyl)-3-hydroxy-3-phenylpropan-1-one (**6**) 25
Procedure for deborylation- (O-allylation) of compound **3c** 25
3-(2-(Allyloxy)phenyl)-1-(4-(tert-butyl)phenyl)-3-hydroxypropan-1-one (**7**) 25

Synthetic Procedure for olefination of **3c** 26

1-(4-(*tert*-Butyl)phenyl)-3-hydroxy-3-(2',3',4',5'-tetrahydro-[1,1'-biphenyl]-2-yl)propan-1-one (**8**): 26

Procedure for selective reduction of ketone of compound **3c** 26

3-(2-(4-(*tert*-Butyl)phenyl)-2-hydroxyethyl)benzo[c][1,2]oxaborol-1(3H)-ol (**9**) ` 27

Procedure for oxidative deborylation of compound **9** 27

1-(4-(*tert*-Butyl)phenyl)-3-(2-hydroxyphenyl)propane-1,3-diol (**10**) 27

Procedure for synthesis of methyl acetal of compound **4c** 28

1-(4-(*tert*-Butyl)phenyl)-2-(2-methyl-4H-benzo[d][1,3]dioxin-4-yl)ethan-1-one (**11**) 28

Procedure for synthesis of phenyl acetal of compound **4c** 28

1-(4-(*tert*-Butyl)phenyl)-2-(2-phenyl-4H-benzo[d][1,3]dioxin-4-yl)ethan-1-one (**12**) 29

NOE experiment of **9** 30

NOE experiment of **12** 31

References 31

Supporting Information - II: *Spectra and Chromatograms* II 1

I. NMR spectra of chiral benzoxaboroles II 2-24

II. NMR and HPLC spectra of β-hydroxy ketones: II 25-70

III. NMR and HPLC spectra of functionalised products: II 71-85

IV. XRD data for 3f II 86

General Experimental Procedures:

All reagents and solvents were used as supplied commercially. Analytical thin-layer chromatography (TLC) were performed on 0.2 mm coated Science silica gel (EM 60-F254) plates purchased from Merck, Germany. Visualization was accomplished with UV light (254 nm) and exposure to either ethanolic phosphomolybdic acid (PMA), anisaldehyde or KMnO4 solution, $CeSO_4$ + ammonium phosphomolybdate + 10% H_2SO4 followed by heating. Melting points are uncorrected. ^{1}H NMR spectra were acquired on a Bruker AVANCE (at 400 MHz, 500 MHz and 700 MHz) and chemical shifts are reported relative to the residual solvent peak. ^{13}C NMR spectra were acquired on Bruker AVANCE (at 100 MHz and 126 MHz) and chemical shifts are reported in ppm relative to the residual solvent peak. Unless noted, NMR spectra were acquired in $CDCl_3$; individual peaks are reported as: multiplicity (s = singlet, d = doublet, t = triplet, q = quartet, m = multiplet), integration, coupling constant in Hz. All IR spectra were obtained as neat films and selected absorbances are reported in cm^{-1}. High resolution data were acquired using Bruker Daltonics MicroTOF-Q-II Mass Spectrometer in MeOH as solvent or using Agilent GCQTOF Mass Spectrometer.

Materials: The organic base $\mathbf{C_5}$ and catalyst $\mathbf{C_8}$ were purchased form Sigma Aldrich and all squaramide and thiourea catalysts were prepared according to the reported procedure in ref. 1. The starting materials (**2a-u)** were also prepared according to the reported procedure in ref. 2. The starting material 2-formyl phenylboronic acid derivatives were also purchased from Sigma Aldrich.

For experimental data of (thiourea and squramide catalysts) see: ref. 1

For experimental data of (**2a-u**) see: Ref. 2

Synthesis of chiral benzoxaboroles: Optimization of the reaction conditions.

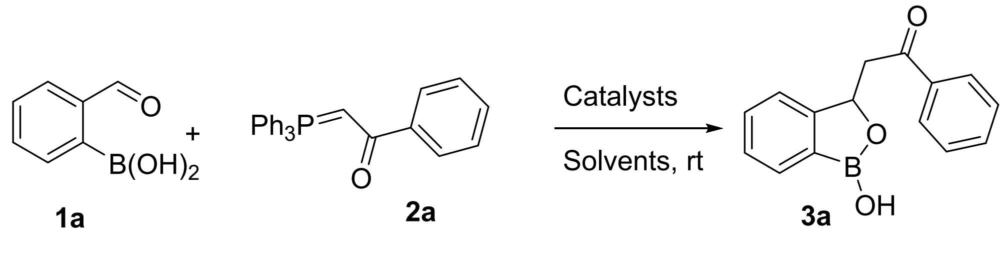

Entry	Solvent	Catalysts	X mol%	Time(h)	Wittig alkene (equiv.)	NMR Yield(%)	% ee
1.	Dichloromethane	C_1	10	36	1.5	26	70
2	Dichloromethane	C_2	10	36	1.5	16	-62
3	Dichloromethane	C_3	10	36	1.5	12	56
4	Dichloromethane	C_4	10	36	1.5	53	90
5	Dichloromethane	C_5	10	36	1.5	<5	ND
6	Dichloromethane	C_6	10	36	1.5	26	86
7	Dichloromethane	C_7	10	36	1.5	55	91
8	Dichloromethane	C_8	10	36	1.5	07	-19
9	Dichloromethane	C_9	10	36	1.5	ND	-16
10	Dichloromethane	C_{10}	10	36	1.5	ND	-42
11	Chloroform	C_7	10	36	1.5	61	90
12	Dichloroethane	C_7	10	36	1.5	65	89
13	Diethyl ether	C_7	10	36	1.5	25	85
14	1,2 dimethoxy ethane	C_7	10	36	1.5	24	87
15	Tetrahydrofuran	C_7	10	36	1.5	15	80
16	Toluene	C_7	10	36	1.5	71	90
17	Chlorobenzene	C_7	10	36	1.5	86	91
18	Carbon tetrachloride	C_7	10	36	1.5	75	89
19	Dimethylformamide	C_7	10	36	1.5	60	91
20	Ethyl acetate	C_7	10	36	1.5	63	87
17	P-Xylene	C_7	10	36	1.5	84	90
18	Mesitylene	C_7	10	36	1.5	ND	ND
19	Triflourotoluene	C_7	10	36	1.5	85	90
20	Trifluoroethanol	C_7	10	36	1.5	ND	ND
21	Chlorobenzene	C_7	5	80	1.5	80	91
22	Chlorobenzene	C_7	15	32	1.5	85	91

[a] Reaction were carried on a 0.04 mmol scale of aldehyde. [b] Yields were determined by ^{1}H NMR using diphenyl acetonitrile as an internal standard. [c] Determined by HPLC analysis on a chiral stationary phase. ND = Not Determined.

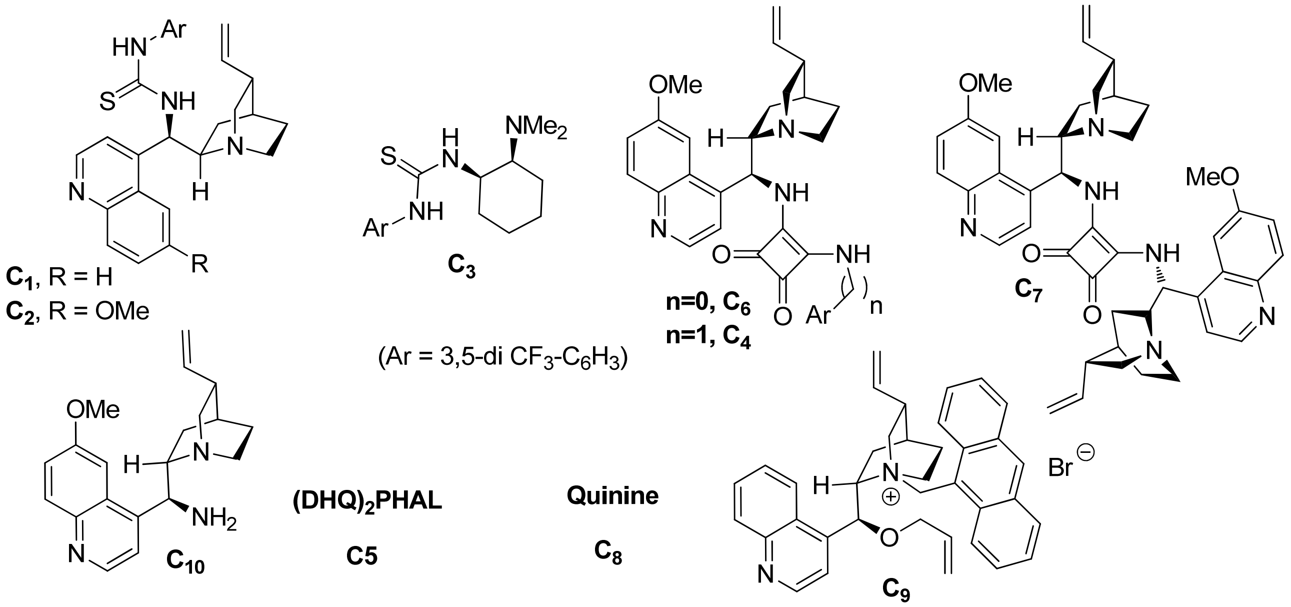

Representative synthetic procedure for chiral benzoxaboroles:

In a 5 mL round bottom flask 2-formyl boronic acid **1** (1 equiv., 0.2 mmol), catalyst **C_7** (0.1equiv. 10 mol %) and Wittig olefin **2** (1.5 equiv., 0.3mmol) were taken with 2 mL chlorobenzene. The whole reaction mixture was stirred at room temperature until all the 2-formyl boronic acid converted to the respective benzoxaboroles. After completion of reaction (monitored by TLC), the solvent was evaporated and purified by column chromatography on silica gel using a mixture of EtOAc/n-hexane as eluent. All racemic compounds were prepared by using 30 mol% Et_3N instead of catalyst. [The enantiomeric ratio was determined with respect to the corresponding alcohol which was obtained after oxidization of **3**.]

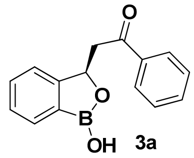

2-(1-Hydroxy-1,3-dihydrobenzo[c][1,2]oxaborol-3-yl)-1-phenylethan-1-one (**3a**): 47.9 mg, 95% yield; R_f = 0.20 (30:70 = EtOAc/n-Hexane); yellow liquid; **FT-IR** (neat): 3409, 2975, 2334, 1652, 1420, 1265, 740 cm^{-1}; **^{1}H NMR (400 MHz, $CDCl_3$)** δ, 7.96 (d, *J* = 7.3 Hz, 2H), 7.75 (d, *J* = 7.2 Hz, 1H), 7.56 (t, *J* = 7.4 Hz, 1H), 7.50 – 7.44 (m, 2H), 7.44 (d, *J* = 7.5 Hz, 1H), 7.37 (t, *J* = 7.6 Hz, 2H),5.92 (dd, *J* = 7.7, 5.0 Hz, 1H), 5.53 (s, 1H), 3.43 (dd, *J* = 17.0, 7.9 Hz, 1H), 3.35 (dd, *J* = 17.0, 4.9 Hz, 1H); **^{13}C NMR (101 MHz, $CDCl_3$)** δ, 197.5, 156.5, 136.8, 133.4 (2C), 131.3, 130.7, 128.7 (2C), 128.3 (2C), 127.7, 121.3, 77.7, 45.7; **HRMS (ESI, *m/z*):** calculated for $C_{15}H_{14}BO_3$ ($[M+H]^+$): 253.1036; found: 253.1033; $[\alpha]_D^{23}$ = + 22.666 (c = 0.075 , $CHCl_3$, 91% ee).

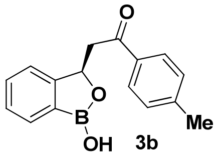

2-(1-Hydroxy-1,3-dihydrobenzo[c][1,2]oxaborol-3-yl)-1-(*p*-tolyl)ethan-1-one (3b): 47.8 mg, 90% yield; R_f = 0.23 (30:70 = EtOAc/n-Hexane); reddish semi-solid; **FT-IR** (neat): 3421, 2975, 2330, 1653, 1420, 1264, 744 cm^{-1}; **^{1}H NMR (400 MHz, $CDCl_3$)** δ, 7.86 (d, *J* = 8.2 Hz, 2H), 7.75 (d, *J* = 7.2 Hz, 1H), 7.46 (td, *J* = 7.5, 0.9 Hz, 1H), 7.37 (t, *J* = 7.6 Hz, 2H), 7.24 (d, *J* = 7.9 Hz, 2H), 5.91 (dd, *J* = 7.8, 4.9 Hz, 1H), 5.34 (s, 1H), 3.39 (dd, *J* = 16.9, 7.9 Hz, 1H), 3.31 (dd, *J* = 16.9, 4.9 Hz, 1H).2.39 (s, 3H); **^{13}C NMR (101 MHz, $CDCl_3$):** δ, 197.0, 156.7, 144.3, 134.4, 131.2, 130.7, 129.4 (2C), 129.3, 128.4 (2C), 127.7, 121.4, 77.8, 45.6, 21.7; **HRMS (ESI, *m/z*):** calculated for $C_{16}H_{15}BO_3Na$ ($[M+Na]^+$): 289.1012; found: 289.1009; $[\alpha]_D^{23}$ = + 5.095 (c = 0.210, $CHCl_3$, 90% ee).

1-(4-(tert-Butyl)phenyl)-2-(1-hydroxy-1,3-dihydrobenzo[c][1,2]oxaborol-3-yl)ethan-1-one (**3c**): 35.7 mg, 58% yield; $R_f = 0.52$ (50:50 = EtOAc/n-Hexane); brown solid; mp 123-125 °C; **FT-IR** (neat): 3414, 2971, 2335, 1651, 1425, 1265, 744 cm^{-1}; **^{1}H NMR (500 MHz, $CDCl_3$)** δ, 7.96 – 7.94 (m, 2H), 7.79 (d, *J* = 7.3 Hz, 1H), 7.51 (d, *J* = 8.6 Hz, 3H), 7.42 (dd, *J* = 10.5, 7.5 Hz, 2H), 5.96 (dd, *J* = 8.0, 4.8 Hz, 1H), 5.09 (s, 1H), 3.45 (dd, *J* = 16.9, 8.1 Hz, 1H), 3.36 (dd, *J* = 16.9, 4.7 Hz, 1H), 1.37 (s, 9H); **^{13}C NMR (126 MHz, $CDCl_3$)** δ, 197.0, 157.3, 156.7, 134.3, 131.3, 130.7, 128.3 (2C), 127.7 (2C), 125.6 (2C), 121.4, 77.9, 45.6, 35.2, 31.0 (3C); **HRMS (ESI, *m/z*):** calculated for $C_{19}H_{22}BO_3$ ($[M+H]^+$): 309.1662; found: 309.1660; $[\alpha]_D^{23}$ = +42.857 (c = 0.175, $CHCl_3$, 99% ee).

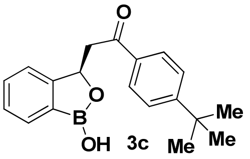

2-(1-Hydroxy-1,3-dihydrobenzo[c][1,2]oxaborol-3-yl)-1-(4-methoxyphenyl)ethan-1-one (**3d**): 51.8 mg, 92% yield; $R_f = 0.10$ (30:70 = EtOAc/n-Hexane); brown semi-solid ; **FT-IR** (neat): 3411, 2970, 2334, 1646, 1421, 1265, 745 cm^{-1}; **^{1}H NMR (400 MHz, $CDCl_3$)** δ, 7.94 (d, *J* = 8.9 Hz, 2H), 7.74 (d, *J* = 7.2 Hz, 1H), 7.46 (dd, *J* = 11.2, 3.7 Hz, 2H), 7.37 (t, *J* = 7.4 Hz, 1H), 6.91 (d, *J* = 8.9 Hz, 1H), 5.91 (dd, *J* = 7.9, 4.8 Hz, 1H), 5.32 (s, 1H), 3.85 (s, 3H), 3.37 (dd, *J* = 16.8, 8.0 Hz, 1H), 3.28 (dd, *J* = 16.7, 4.8 Hz, 1H).; **^{13}C NMR (101 MHz, $CDCl_3$)** δ, 195.9, 163.8, 156.7, 131.2, 130.6 (3C), 130.0, 127.7, 121.4, 113.8 (3C), 77.9, 55.5, 45.3; **HRMS (ESI, *m/z*):** calculated for $C_{16}H_{16}BO_4$ ($[M+H]^+$): 283.1141; found: 283.1139; $[\alpha]_D^{23}$ = +10.264 (c = 0.530, $CHCl_3$, 90% ee).

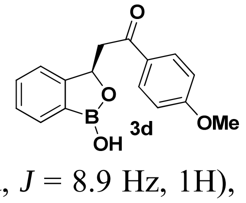

1-(3,4-Dimethoxyphenyl)2-(1-hydroxy-1,3-dihydrobenzo[c][1,2]oxaborol-3-yl)ethan-1-one (**3e**): 47.4 mg, 76% yield; $R_f = 0.10$ (30:70 = EtOAc/n-Hexane); brown oil; **FT-IR** (neat): 3401, 2361, 2334, 1645, 1265, 741 cm^{-1}; **^{1}H** **^{1}H NMR (400 MHz, $CDCl_3$)** δ, 7.74 (d, *J* = 7.2 Hz, 1H), 7.57 (d, *J* = 1.7 Hz, 1H), 7.54 (dd, *J* = 8.4, 1.9 Hz, 1H), 7.48 (t, *J* = 7.2 Hz, 1H), 7.38 (t, *J* = 7.4 Hz, 1H), 6.85 (d, *J* = 8.4 Hz, 1H), 5.91 (dd, *J* = 8.0, 4.7 Hz, 1H), 5.14 (s, 1H), 3.92 (s, 6 H), 3.39 (dd, *J* = 16.7, 8.1 Hz, 1H), 3.29 (dd, *J* = 16.7, 4.7 Hz, 1H); **^{13}C NMR (101 MHz, $CDCl_3$)** δ, 195.9, 156.7, 153.6, 149.1, 137.5, 131.3, 130.7, 130.3, 127.7, 123.1, 121.4, 110.3, 110.0, 78.0, 56.1, 56.0, 45.2; **HRMS (ESI, *m/z*):** calculated for $C_{17}H_{18}BO_5$ ($[M+H]^+$): 313.1247; found: 313.1245; $[\alpha]_D^{23}$ = + 8.222 (c = 0.900, $CHCl_3$, 90% ee).

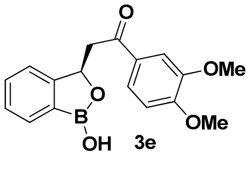

1-(Benzo[*d*][1,3]dioxol-5-yl)-2-(1-hydroxy-1,3-dihydrobenzo[c][1,2]oxaborol-3-yl)ethan-1-one (**3f**): 50.9 mg, 86 % yield; R_f = 0.18 (30:70 = EtOAc/n-Hexane); yellow semi-solid ; **FT-IR** (neat): 3422, 2364, 2331, 1657, 1261, 747 cm^{-1}; **^{1}H NMR (500 MHz, CDCl$_3$)** δ, 7.74 (d, *J* = 7.2 Hz, 1H), 7.51 (dd, *J* = 8.1, 1.2 Hz, 1H), 7.45 (dd, *J* = 7.9, 6.1 Hz, 2H), 7.35 (dd, *J* = 10.7, 7.5 Hz, 2H), 6.80 (d, *J* = 8.2 Hz, 1H), 6.07 (s, 2H), 5.92 (dd, *J* = 7.6, 5.0 Hz, 1H), 5.15 (s, 1H), 3.33 (dd, *J* = 16.8, 7.9 Hz, 1H), 3.26 (dd, *J* = 16.8, 4.8 Hz, 1H); **^{13}C NMR (126 MHz, CDCl$_3$)** δ, 195.5, 156.5, 152.0, 148.3, 131.8, 131.2, 130.7, 127.7(2C), 124.8, 121.3, 108.0, 107.9, 101.9, 77.9, 45.4; **HRMS (ESI, *m/z*):** calculated for $C_{16}H_{14}BO_5$([M+H]$^+$): 297.0934; found: 297.0932; $[\alpha]_D^{23}$ = +13.504 (c = 0.605, CHCl$_3$, 84% ee).

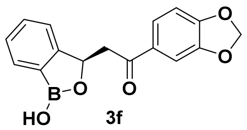

1-(4-Chlorophenyl)-2-(1-hydroxy-1,3-dihydrobenzo[c][1,2]oxaborol-3-yl)ethan-1-one (**3g**): 55.6 mg, 97% yield; R_f = 0.12 (30:70 = EtOAc/n-Hexane); yellow solid; mp 100 °C; **FT-IR** (neat): 3420, 2971, 2334, 1658, 1420, 1265, 744 cm^{-1}; **^{1}H NMR (400 MHz, CDCl$_3$)** δ, 7.89 (d, *J* = 8.6 Hz, 2H), 7.74 (d, *J* = 7.2 Hz, 1H), 7.48 (t, *J* = 7.2 Hz, 1H), 7.42 (d, *J* = 8.5 Hz, 2H), 7.39 – 7.32 (m, 2H), 5.88 (dd, *J* = 7.6, 4.9 Hz, 1H), 5.28 (s, 1H), 3.38 (dd, *J* = 17.0, 7.9 Hz, 1H), 3.31 (dd, *J* = 17.0, 4.8 Hz, 1H); **^{13}C NMR (101 MHz, CDCl$_3$)** δ, 196.2, 156.3, 140.0, 135.2, 131.3, 130.7, 129.7 (2C), 129.0 (2C), 127.8 (2C), 121.3, 77.7, 45.6; **HRMS (ESI, *m/z*):** calculated for $C_{15}H_{13}BClO_3$ ([M+H]$^+$): 287.0646; found: 287.0643; $[\alpha]_D^{23}$ = +10.405 (c = 0.185, CHCl$_3$, 90% ee).

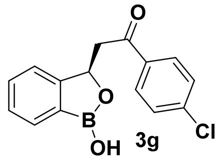

1-(4-Bromophenyl)-2-(1-hydroxy-1,3-dihydrobenzo[c][1,2]oxaborol-3-yl)ethan-1-one (**3h**): 45.7 mg, 69% yield; R_f = 0.18 (30:70 = EtOAc/n-Hexane); light orange solid; mp 113-115 °C; **FT-IR** (neat): 3411, 2972, 2330, 1656, 1420, 1265, 746 cm^{-1}; **^{1}H NMR (500 MHz, CDCl$_3$)** δ, 7.86 (d, *J* = 8.5 Hz, 2H), 7.78 (d, *J* = 7.3 Hz, 1H), 7.64 (d, *J* = 8.5 Hz, 2H), 7.52 (t, *J* = 7.5 Hz, 1H), 7.43 (t, *J* = 7.3 Hz, 1H), 7.39 (d, *J* = 7.7 Hz, 1H), 5.93 (dd, *J* = 8.0, 4.7 Hz, 1H), 5.09 (s, 1H), 3.41 (dd, *J* = 16.9, 8.0 Hz, 1H), 3.35 (dd, *J* = 16.9, 4.7 Hz, 1H); **^{13}C NMR (126 MHz, CDCl$_3$)** δ, 196.4, 156.4, 135.6, 132.0 (2C), 131.4, 130.7, 129.8 (2C), 128.7, 127.8 (2C), 121.3, 77.6, 45.6; **HRMS (ESI, *m/z*):** calculated for $C_{15}H_{13}BBrO_3$ ([M+H]$^+$): 331.0141; found: 331.0138; $[\alpha]_D^{23}$ = +16.852 (c = 0.305, CHCl$_3$, 92% ee).

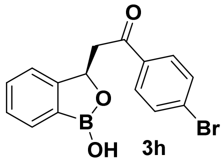

2-(1-Hydroxy-1,3-dihydrobenzo[c][1,2]oxaborol-3-yl)-1-(4-iodophenyl)ethan-1-one (3i): 48.4 mg, 64% yield; R_f= 0.19 (30:70 = EtOAc/n-Hexane); orange solid; mp 59-61 °C; **FT-IR** (neat): 3421, 2976, 2338, 1650, 1420, 1265, 744 cm^{-1}; **^{1}H NMR (500 MHz, $CDCl_3$)** δ, 7.86 (d, *J* = 8.5 Hz, 1H), 7.78 (d, *J* = 7.3 Hz, 1H), 7.70 (d, *J* = 8.5 Hz, 1H), 7.52 (td, *J* = 7.5, 1.1 Hz, 1H), 7.42 (t, *J* = 7.3 Hz, 1H), 7.39 (d, *J* = 7.6 Hz, 1H), 5.92 (dd, *J* = 8.0, 4.7 Hz, 1H), 5.07 (s, 1H), 3.40 (dd, *J* = 16.9, 8.0 Hz, 1H), 3.34 (dd, *J* = 16.9, 4.7 Hz, 1H); **^{13}C NMR (126 MHz, $CDCl_3$)** δ, 196.73, 156.4, 138.0, 136.1, 131.4, 130.7, 129.7(3C), 127.8(2C), 121.3, 101.6, 77.6, 45.5; **HRMS (ESI, *m/z*):** calculated for $C_{15}H_{12}BIO_3Na$ ($[M+Na]^+$): 400.9822; found: 400.9819; $[\alpha]_D^{23}$ = +15.834 (c = 0.485, $CHCl_3$, 90% ee).

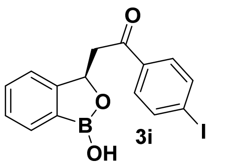

1-(4-Fluorophenyl)-2-(1-hydroxy-1,3-dihydrobenzo[c][1,2]oxaborol-3-yl)ethan-1-one (3j): 48 mg, 88% yield; R_f = 0.25 (30:70 = EtOAc/n-Hexane); white semi-solid; **FT-IR** (neat): 3420, 2977, 2334, 1662, 1420, 1265, 742 cm^{-1}; **^{1}H NMR (500 MHz, $CDCl_3$)** δ, 8.03 (dd, *J* = 8.7, 5.4 Hz, 2H), 7.78 (d, *J* = 7.2 Hz, 1H), 7.50 (t, *J* = 7.1 Hz, 1H), 7.41 (dd, *J* = 13.0, 6.7 Hz, 2H), 7.15 (t, *J* = 8.6 Hz, 2H), 5.93 (dd, *J* = 7.6, 4.9 Hz, 1H), 5.75 (s, 1H), 3.43 (dd, *J* = 16.9, 7.9 Hz, 1H), 3.36 (dd, *J* = 16.9, 4.8 Hz, 1H); **^{13}C NMR (126 MHz, $CDCl_3$)** δ, 195.9, 165.9 (d, J = 255.5 Hz), 156.4, 133.3 (d, J = 2.7 Hz),, 131.3, 131.0 (d, J = 9.4 Hz, 2C), 130.7, 127.8, 121.3, 115.8 (d, J = 21.9 Hz, 2C), 115.1, 77.7, 45.6; **HRMS (ESI, *m/z*):** calculated for $C_{15}H_{12}BFO_3Na$ ($[M+Na]^+$): 293.0762; found: 293.0758; $[\alpha]_D^{23}$ = +1.354 (c = 0.480, $CHCl_3$, 91% ee).

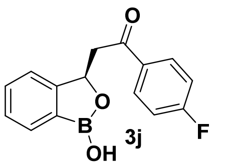

2-(1-Hydroxy-1,3-dihydrobenzo[c][1,2]oxaborol-3-yl)-1-(4-(trifluoromethyl)phenyl)ethan-1-one) (3k): 46.7 mg, 73 % yield; R_f = 0.24 (30:70 = EtOAc/n-Hexane); brown solid; mp 99°C ; **FT-IR** (neat): 3401, 2977, 2334, 1664, 1420, 1264, 742 cm^{-1}. **^{1}H NMR (500 MHz, $CDCl_3$)** δ, 8.10 (d, *J* = 8.1 Hz, 2H), 7.79 (d, *J* = 7.3 Hz, 1H), 7.76 (d, *J* = 8.2 Hz, 2H), 7.53 (td, *J* = 7.5, 1.1 Hz, 1H), 7.43 (t, *J* = 7.3 Hz, 1H), 7.40 (dd, *J* = 7.7, 0.7 Hz, 1H), 5.95 (dd, *J* = 7.8, 4.8 Hz, 1H), 5.34 (s, 1H), 3.47 (dd, *J* = 17.1, 7.9 Hz, 1H), 3.42 (dd, *J* = 17.0, 4.8 Hz, 1H); **^{13}C NMR (126 MHz, $CDCl_3$)** δ, 196.5, 156.0, 139.4, 134.9, 131.4, 130.8, 129.7, 128.6 (2C), 127.9, 125.7 (qt, *J* =16Hz), 124.3 (qt, *J* = 274.4Hz), 122.2, 121.2, 77.5, 45.9; **HRMS**

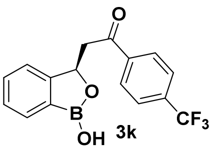

(ESI, *m/z*): calculated for $C_{16}H_{13}BF_3O_3$ ($[M+H]^+$): 321.0910; found: 321.0907; $[\alpha]_D^{23}$ = +15.942 (c = 0.190, $CHCl_3$, 74% ee).

1-([1,1'-Biphenyl]-4-yl)-2-(1-hydroxy-1,3-dihydrobenzo[c][1,2]oxaborol-3-yl)ethan-1-one (3l): 47 mg, 72% yield; R_f = 0.62 (50:50 = EtOAc/n-Hexane); white solid; mp 135-137 °C; **FT-IR** (neat): 3362, 2362, 2334, 1653, 750 cm^{-1}; **^{1}H NMR (400 MHz, $CDCl_3$)** δ, 8.03 (d, *J* = 8.3 Hz, 2H), 7.77 (d, *J* = 7.3 Hz, 1H), 7.66 (d, *J* = 8.3 Hz, 2H), 7.60 (d, *J* = 7.3 Hz, 2H), 7.46 (dd, *J* = 14.1, 7.4 Hz, 3H), 7.39 (t, *J* = 6.6 Hz, 3H), 5.94 (dd, *J* = 7.8, 4.9 Hz, 1H), 5.33 (s, 1H), 3.45 (dd, *J* = 16.9, 8.0 Hz, 1H), 3.37 (dd, *J* = 16.9, 4.8 Hz, 1H; **^{13}C NMR (101 MHz, $CDCl_3$)** δ, 197.0, 156.6, 146.1, 139.8, 135.6, 131.3, 130.7, 129.0 (3C), 128.9 (2C), 128.3 (2C), 127.7, 127.3 (3C), 121.4, 77.8, 45.7; **HRMS (ESI, *m/z*):** calculated for $C_{21}H_{18}BO_3$ ($[M+H]^+$): 329.1349; found: 329.1347; $[\alpha]_D^{23}$ = +0.202 (c = 0.635, $CHCl_3$, 90% ee).

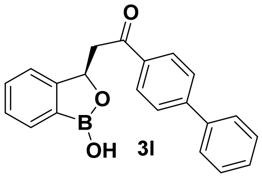

3l

2-(1-Hydroxy-1,3-dihydrobenzo[c][1,2]oxaborol-3-yl)-1-(thiophen-2-yl)ethan-1-one (3m): 45.9 mg, 89% yield; R_f = 0.12 (30:70 = EtOAc/n-Hexane); yellow solid; mp 126 °C; **FT-IR** (neat): 3397, 2365, 2345, 1637, 1262, 745 cm^{-1}; **^{1}H NMR (500 MHz, $CDCl_3$)** δ, 7.79 (d, *J* = 7.3 Hz, 1H), 7.72 (dd, *J* = 3.8, 1.0 Hz, 1H), 7.70 (dd, *J* = 4.9, 1.0 Hz, 1H), 7.52 (td, *J* = 7.5, 1.1 Hz, 1H), 7.43 (d, *J* = 7.3 Hz, 1H), 7.41 – 7.38 (m, 1H), 7.15 (dd, *J* = 4.9, 3.9 Hz, 1H), 5.91 (dd, *J* = 8.0, 4.9 Hz, 1H), 5.13 (s, 1H), 3.40 – 3.34 (m, 1H), 3.32 (dd, *J* = 16.2, 4.9 Hz, 1H); **^{13}C NMR (126 MHz, $CDCl_3$)** δ, 190.1, 156.3, 144.2, 134.4, 132.6, 131.3, 130.7, 128.2, 128.2, 127.8, 121.3, 77.8, 46.4; **HRMS (ESI, *m/z*):** calculated for $C_{13}H_{12}BO_3S$ ($[M+H]^+$): 259.0600; found: 259.0597; $[\alpha]_D^{23}$ = +2.612 (c = 0.122, $CHCl_3$, 94% ee).

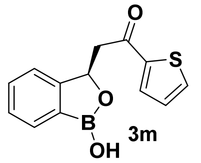

3m

1-(Furan-2-yl)-2-(1-hydroxy-1,3-dihydrobenzo[c][1,2]oxaborol-3-yl)ethan-1-one (3n): 38.7 mg, 80% yield; R_f= 0.20 (30:70 = EtOAc/n-Hexane); light yellow liquid ; **FT-IR** (neat): 3395, 2365, 2344, 1642, 1267, 747 cm^{-1}; **^{1}H NMR (500 MHz, $CDCl_3$)** δ, 7.78 (d, *J* = 7.3 Hz, 1H), 7.63 – 7.60 (m, 1H), 7.51 (td, *J* = 7.5, 1.1 Hz, 1H), 7.41 (t, *J* = 7.3 Hz, 1H), 7.38 (dd, *J* = 7.7, 0.6 Hz, 1H), 7.26 (d, *J* = 3.6 Hz, 1H), 6.57 (dd, *J* = 3.6, 1.7 Hz, 1H), 5.89 (dd, *J* = 7.7, 5.4 Hz, 1H), 5.19 (s, 1H), 3.30 (dd, *J* = 14.8, 6.3 Hz, 1H), 3.25 (dd, *J* = 14.7, 3.8 Hz, 1H); **^{13}C NMR (126 MHz, $CDCl_3$)** δ, 186.3, 156.3, 152.7, 146.8, 131.3, 130.7, 128.3, 127.7, 121.3, 117.9, 112.5,

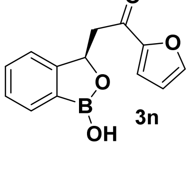

3n

77.5, 45.5; **HRMS (ESI, *m/z*):** calculated for $C_{13}H_{12}BO_4$ ($[M+H]^+$): 243.0828; found: 243.0826; $[\alpha]_D^{23}$ = + 0.881 (c = 0.465, $CHCl_3$, 92% ee).

2-(1-Hydroxy-6-methyl-1,3-dihydrobenzo[c][1,2]oxaborol-3-yl)-1-(thiophen-2-yl)ethan-1-one (**3o**): 17.9 mg, 33 % yield; R_f = 0.50 (50:50 = EtOAc/n-Hexane); brown semi-solid; **FT-IR** (neat): 3398, 2363, 2345, 1636, 1265, 744 cm^{-1}; **^{1}H NMR (400 MHz, $CDCl_3$)** δ, 7.66 (dd, *J* = 7.1, 4.4 Hz, 2H), 7.54 (s, 1H), 7.29 (d, *J* = 7.8 Hz, 1H), 7.24 (d, *J* = 7.8 Hz, 1H), 7.11 (dd, *J* = 4.8, 4.0 Hz, 1H), 5.83 (dd, *J* = 7.6, 5.3 Hz, 1H), 4.92 (s, 1H), 3.30 (dd, *J* = 16.1, 7.8 Hz, 1H), 3.25 (dd, *J* = 16.1, 5.0 Hz, 1H), 2.39 (s, 3H); **^{13}C NMR (101 MHz, $CDCl_3$)** δ, 190.2, 153.6, 144.3, 137.4, 134.3 (2C), 132.6, 132.4, 130.9, 128.2, 121.1, 77.7, 46.5, 21.2; **HRMS (ESI, *m/z*):** calculated for $C_{14}H_{13}BO_3SNa$ ($[M+Na]^+$): 295.0576; found: 295.0573; $[\alpha]_D^{23}$ = +41.111 (c = 0.180, $CHCl_3$, 94% ee).

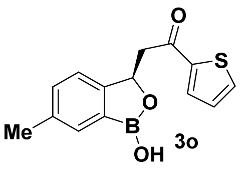

1-(4-(*tert*-Butyl)phenyl)-2-(1-hydroxy-6-methyl-1,3-dihydrobenzo[c][1,2]oxaborol-3-yl)ethan-1-one (**3p**): 54 mg, 84% yield; R_f = 0.30 (30:70 = EtOAc/n-Hexane); yellow liquid; **FT-IR** (neat): 3411, 2975, 2334, 1651, 1420, 1265, 746 cm^{-1}; **^{1}H NMR (400 MHz, $CDCl_3$)** δ, 7.90 (d, *J* = 8.5 Hz, 2H), 7.55 (s, 1H), 7.46 (d, *J* = 8.5 Hz, 2H), 7.29 (d, *J* = 7.7 Hz, 1H), 7.26 – 7.23 (m, 1H), 5.88 (dd, *J* = 7.7, 5.0 Hz, 1H), 5.23 (s, 1H), 3.39 (dd, *J* = 16.9, 7.9 Hz, 1H), 3.29 (dd, *J* = 16.9, 4.9 Hz, 1H), 2.39 (s, 3H), 1.32 (s, 9H); **^{13}C NMR (101 MHz, $CDCl_3$)** δ, 197.1, 157.2, 154.1, 137.3, 134.4, 132.3 (2C), 130.9, 128.3 (2C), 125.6 (2C), 121.1, 77.7, 45.7, 35.2, 31.0 (3C), 21.3; **HRMS (ESI, *m/z*):** calculated for $C_{20}H_{24}BO_3$ ($[M+H]^+$): 323.1818; found: 323.1817; $[\alpha]_D^{24}$ = +9.402 (c = 0.230, $CHCl_3$, 85% ee).

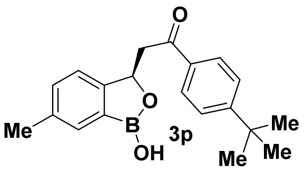

1-(4-(*tert*-Butyl)phenyl)-2-(1-hydroxy-6-methoxy-1,3-dihydrobenzo[c][1,2]oxaborol-3-yl)ethan-1-one (**3q**): 61.5 mg, 91% yield; R_f = 0.32 (30:70 = EtOAc/n-Hexane); white semi-solid; **FT-IR** (neat): 3411, 2975, 2334, 1652, 1420, 1260,1167, 746 cm^{-1}; **^{1}H NMR (400 MHz, $CDCl_3$)** δ, 7.90 (d, *J* = 8.5 Hz, 2H), 7.64 (d, *J* = 8.2 Hz, 1H), 7.46 (d, *J* = 8.5 Hz, 2H), 6.92 (dd, *J* = 8.2, 1.9 Hz, 1H), 6.86 (s, 1H), 5.85 (dd, *J* = 7.7, 5.0 Hz, 1H), 5.08 (s, 1H), 3.81 (s, 3H), 3.42 (dd, *J* = 16.9, 7.9 Hz, 1H), 3.29 (dd, *J* = 16.9, 4.9 Hz, 1H), 1.32 (s, 9H); **^{13}C NMR (101 MHz, $CDCl_3$)** δ, 197.2, 162.6, 159.2, 157.2, 134.4, 134.3, 132.0, 128.3 (2C), 125.6 (2C), 114.9,

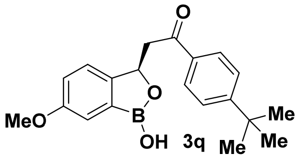

106.2, 77.4, 55.3, 45.7, 35.1, 31.0 (3C); **HRMS (ESI, *m/z*):** calculated for $C_{20}H_{24}BO_4$ ($[M+H]^+$): 339.1767; found: 339.1766; $[\alpha]_D^{24}$ = +33.472 (c = 0.090, $CHCl_3$, 90% ee).

2-(5-Fluoro-1-hydroxy-1,3-dihydrobenzo[c][1,2]oxaborol-3-yl)-1-(thiophen-2-yl)ethan-1-one (**3r**): 41.9 mg, 76% yield; R_f = 0.36 (50:50 = EtOAc/n-Hexane); white solid; mp 111 °C ; **FT-IR** (neat): 3402, 2355, 2331, 1648, 1265, 746 cm^{-1}; **^{1}H NMR (400 MHz, $CDCl_3$)** δ, 7.74 – 7.70 (m, 1H), 7.69 – 7.67 (m, 1H), 7.66 (s, 1H), 7.12 (dd, *J* = 4.7, 4.0 Hz, 1H), 7.06 (d, *J* = 8.9 Hz, 2H), 5.82 (dd, *J* = 7.8, 5.1 Hz, 1H), 5.18 (s, 1H), 3.35 (dd, *J* = 16.5, 7.9 Hz, 1H), 3.25 (dd, *J* = 16.5, 5.0 Hz, 1H); **^{13}C NMR (101 MHz, $CDCl_3$)** δ, 189.8, 165.3 (d, *J* = 250.8 Hz), 159.0 (d, *J* = 8.4 Hz), 144.0, 134.5, 132.7, 132.6, 128.3, 128.2, 115.7 (d, *J* = 22.0 Hz), 108.8 (d, *J* = 22.5 Hz).77.2, 46.0; **HRMS (ESI, *m/z*):** calculated for $C_{13}H_{11}BFO_3S$ ($[M+H]^+$): 277.0506; found: 277.0503; $[\alpha]_D^{23}$ = +1.884 (c = 0.610, $CHCl_3$, 94% ee).

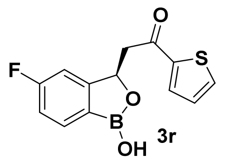

3r

2-(4-Fluoro-1-hydroxy-1,3-dihydrobenzo[c][1,2]oxaborol-3-yl)-1-(thiophen-2-yl)ethan-1-one (**3s**): 46 mg, 83% yield; R_f = 0.47 (50:50 = EtOAc/n-Hexane); yellow solid; mp 82 °C; **FT-IR** (neat): 3414, 2359, 2338, 1648, 1255, 744 cm^{-1}; **^{1}H NMR (500 MHz, $CDCl_3$)** δ, 7.76 – 7.72 (m, 1H), 7.70 (dd, *J* = 4.9, 0.7 Hz, 1H), 7.57 (d, *J* = 7.1 Hz, 1H), 7.43 (td, *J* = 7.6, 4.5 Hz, 1H), 7.20 (d, *J* = 9.2 Hz, 1H), 7.16 (dd, *J* = 8.3, 3.6 Hz, 1H), 6.02 (dd, *J* = 9.7, 1.7 Hz, 1H), 5.06 (s, 1H), 3.66 (dd, *J* = 16.2, 2.4 Hz, 1H), 3.20 (dd, *J* = 16.2, 9.8 Hz, 1H); **^{13}C NMR (126 MHz, $CDCl_3$)** δ, 189.5, 159.2 (d, J = 256.1 Hz), 156.2, 144.2, 141.2, 134.3, 132.5, 130.4 (d, *J* = 5.8 Hz), 128.2, 126.6 (d, *J* = 3.9 Hz), 117.9 (d, *J* = 19.9 Hz), 75.6(d, J = 2.9 Hz), 44.4; **HRMS (ESI, *m/z*):** calculated for $C_{13}H_{11}BFO_3S$ ($[M+H]^+$): 277.0506; found: 277.0503 $[\alpha]_D^{25}$ = +560.170 (c = 0.235, $CHCl_3$, 92% ee).

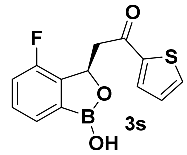

3s

1-(4-(*tert*-Butyl)phenyl)-2-(4-fluoro-1-hydroxy-1,3-dihydrobenzo[c][1,2]oxaborol-3-yl)ethan-1-one (**3t**): 56 mg, 86% yield; R_f = 0.69 (50:50 = EtOAc/n-Hexane); colourless liquid; **FT-IR** (neat): 3418, 2978, 2334, 1659, 1420, 1265, 745 cm^{-1}; **^{1}H NMR (400 MHz, $CDCl_3$)** δ, 7.90 (d, *J* = 8.5 Hz, 2H), 7.54 (d, *J* = 7.1 Hz, 1H), 7.46 (d, *J* = 8.5 Hz, 2H), 7.37 (dd, *J* = 11.9, 7.5 Hz, 1H), 7.17 – 7.10 (m, 1H), 6.01 (d, *J* = 9.2 Hz, 1H), 5.59 (s, 1H), 3.66 (dd, *J* = 16.8, 2.1 Hz, 1H), 3.21 (dd, *J* = 16.8, 9.8 Hz, 1H), 1.32 (s, 9H); **^{13}C NMR (101 MHz, $CDCl_3$)** δ, 196.5, 157.2,

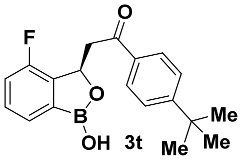

3t

157.1 (d, J = 249.3 Hz), 141.6, 141.4, 134.2, 130.3 (d, J = 5.6 Hz), 128.3 (2C), 126.7 (d, J = 3.4 Hz), 125.6 (2C), 117.8 (d, J = 19.6 Hz), 75.5 (d, J = 2.1 Hz), 43.6, 35.1, 31.0 (3C); **HRMS (ESI, *m/z*):** calculated for $C_{19}H_{21}BFO_3$ ($[M+H]^+$): 327.1568; found: 327.1566; $[\alpha]_D^{24}$ = +51.766 (c = 1.285, $CHCl_3$, 84% ee).

1-Hydroxy-3-(2-oxo-2-(thiophen-2-yl)ethyl)-1,3-dihydrobenzo[c][1,2]oxaborole-6-carbonitrile (**3u**): 22 mg, 39% yield; R_f = 0.42 (50:50 = EtOAc/n-Hexane); yellow liquid ; **FT-IR** (neat): 3412, 2359, 2334, 1648, 1265, 747 cm^{-1}; **^{1}H NMR (500 MHz, $CDCl_3$)** δ, 8.09 (s, 1H), 8.78 (d, J = 7.9 Hz, 1H), 7.72 (dd, J = 7.5, 1.4 Hz, 2H), 7.53 (d, J = 2.9 Hz, 1H), 7.17 (t, 1H), 5.95 (dd, J = 7.3, 5.4 Hz, 1H).), 5.36 (s, 1H), 3.44 (dd, J = 16.4, 7.8 Hz, 1H), 3.32 (dd, J = 16.3, 4.9 Hz, 1H); **^{13}C NMR (126 MHz, $CDCl_3$)** δ, 189.3, 160.5, 143.8, 135.0,134.9, 134.7, 134.6, 132.7, 128.4, 122.5, 118.8, 112.1, 77.8, 45.6; **HRMS (ESI, *m/z*):** calculated for $C_{14}H_{11}BNO_3S$ ($[M+H]^+$): 284.0552; found: 284.0550; $[\alpha]_D^{25}$ = +1.037 (c = 0.675, $CHCl_3$, 98% ee).

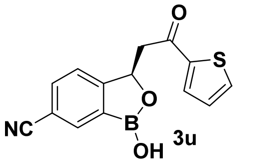

1-(4-(*tert*-Butyl)phenyl)-2-(5-chloro-1-hydroxy-1,3-dihydrobenzo[c][1,2]oxaborol-3-yl)ethan-1-one (**3v**): 53.4 mg, 78% yield; R_f = 0.27 (30:70 = EtOAc/n-Hexane); yellow semi-solid; **FT-IR** (neat): 3409, 2977, 2334, 1658, 1420, 1265, 742 cm^{-1}; **^{1}H NMR (400 MHz, $CDCl_3$)** δ, 7.89 (d, J = 8.4 Hz, 2H), 7.66 (d, J = 7.8 Hz, 1H), 7.46 (d, J = 8.4 Hz, 2H), 7.38 – 7.32 (m, 2H), 5.88 (dd, J = 7.3, 5.3 Hz, 1H), 5.50 (s, 1H), 3.42 (dd, J = 17.2, 7.7 Hz, 1H), 3.30 (dd, J = 17.2, 5.0 Hz, 1H), 1.32 (s, 9H); **^{13}C NMR (101 MHz, $CDCl_3$)** δ, 196.7, 158.4, 157.4, 137.8, 134.1, 131.9, 128.3, 128.2 (3C), 125.7 (2C), 121.9, 77.3, 45.3, 35.2, 31.0 (3C); **HRMS (ESI, *m/z*):** calculated for $C_{19}H_{21}BClO_3$ ($[M+H]^+$): 343.1272; found: 343.1270; $[\alpha]_D^{24}$ = +27.936 (c = 0.420, $CHCl_3$, 87% ee).

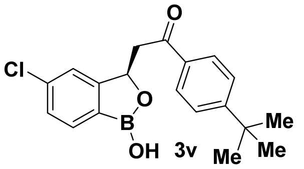

1-([1,1'-Biphenyl]-4-yl)-2-(1-hydroxy-1,3-dihydronaphtho[2,3-c][1,2]oxaborol-3-yl)ethan-1-one (**3w**): 69.5 mg, 92% yield; R_f = 0.58 (50:50 = EtOAc/n-Hexane); brown solid; mp 161-163 °C; **FT-IR** (neat): 3375, 2923, 2364, 1653, 1189, 763 cm^{-1}; **^{1}H NMR (400 MHz, $CDCl_3$)** δ, 8.38 (d, J = 8.1 Hz, 1H), 8.05 (d, J = 8.4 Hz, 2H), 7.96 (d, J = 8.4 Hz, 1H), 7.89 (d, J = 8.1 Hz, 1H), 7.67 (d, J = 8.4 Hz, 2H), 7.62 – 7.56 (m, 3H), 7.55 – 7.49 (m, 1H), 7.45 (t, J =

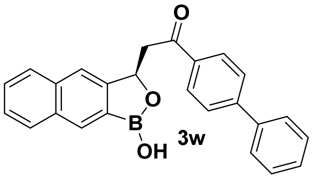

7.8 Hz, 3H), 7.39 (dd, *J* = 8.3, 6.2 Hz, 1H), 6.04 (t, *J* = 6.4 Hz, 1H), 5.41 (s, 1H), 3.50 – 3.44 (m, 1H), 3.44 (dd, *J* = 12.9, 11.2 Hz, 1H); **^{13}C NMR (101 MHz, $CDCl_3$)** δ, 197.0, 156.8, 146.1, 139.7, 135.6, 134.5, 132.8, 132.4, 129.0 (5C), 128.3, 128.3, 127.3 (4C), 127.2, 126.1 (2C), 119.3, 77.8, 45.4; **HRMS (ESI, *m/z*):** calculated for $C_{25}H_{20}BO_3$ ($[M+H]^+$): 379.1505; found: 379.1504; $[\alpha]_D^{24}$ = +0.094 (c = 1.060, $CHCl_3$, 58% ee).

Representative synthetic procedure for β-hydroxy ketone:

In a 5 ml round bottom flask, benzoxaborole **3** (0.04 mmol to 0.15 mmol, 1equiv) were taken followed by addition of 2 ml ethyl acetate as a solvent and stirring it. Then in this flask saturated aqueous Na_2CO_3 solution (0.5 mL to 1mL) was taken followed by addition of 31% aqueous H_2O_2 (0.02 to 0.1 mL) and stirring the reaction mixture upto15 to 20 minutes under room temperature. After completion of the starting benzoxaboroles **3**, reaction was quenched with $Na_2S_2O_3$ solution followed by $NaHCO_3$ solution (1:5) and stirring up to 5-10 minutes. The organic layer was extracted with EtOAc (10 mL x 3) and dried over anhydrous Na_2SO_4 and the residue was purified by flash column chromatography on silica gel using EtOAc / hexanes as an eluent to give the corresponding product **4**.
The enantiomeric ratio was determined by HPLC analysis using a chiral column and a mixture of n-Hexane/2-propanol as eluent (flow rate 1.0 mL/min, λ = 254 nm). The retention time (t_R) for each enantiomer of the products are given along with the experimental data.

3-Hydroxy-3-(2-hydroxyphenyl)-1-phenylpropan-1-one (**4a**): 18 mg, 78% yield; R_f = 0.46 (30:70 = EtOAc/n-Hexane); Yellowish liquid; **FT-IR** (neat): 3396, 2924, 2334, 2360, 1652, 1265, 752 cm^{-1}; **^{1}H NMR (400 MHz, $CDCl_3$)** δ, 8.31 (s, 1H), 7.98 – 7.92 (m, 2H), 7.60 (t, *J* = 7.4 Hz, 1H), 7.47 (t, *J* = 7.7 Hz, 2H), 7.23 – 7.17 (m, 1H), 7.01 (dd, *J* = 7.5, 1.3 Hz, 1H), 6.94 – 6.89 (m, 1H), 6.85 (td, *J* = 7.5, 1.0 Hz, 1H), 5.48 (dd, *J* = 9.9, 2.6 Hz, 1H), 4.61 (s, 1H), 3.57 (dd, *J* = 18.3, 9.9 Hz, 2H), 3.42 (dd, *J* = 18.3, 2.7 Hz, 2H); **^{13}C NMR (101 MHz, $CDCl_3$)** δ, 201.0, 155.9, 136.1, 134.1, 129.3, 128.8 (2C), 128.2 (2C), 126.7, 125.8, 120.0, 117.6, 71.8, 45.1; **HRMS (ESI, *m/z*):** calculated for $C_{15}H_{14}O_3Na$ ($[M+Na]^+$): 265.0841; found: 265.0835; $[\alpha]_D^{26}$ = +34.058 (c = 0.690, $CHCl_3$, 91% ee).

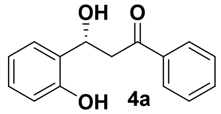

The enantiomeric ratio was determined by HPLC analysis using a Daicel Chiralpak OZ-3 column (Hexane/2-propanol = 85:15, flow rate 1.0 mL/min, λ = 254 nm), t_R = 11.7 min (major), t_R = 15.2 min (minor).

3-Hydroxy-3-(2-hydroxyphenyl)-1-(p-tolyl)propan-1-one (**4b**): 19 mg, 65% yield; R_f = 0.40 (30:70 = EtOAc/n-Hexane); yellow semi-solid; **FT-IR** (neat): 3401, 2929, 2332, 2360, 1650, 1261, 751 cm^{-1}; ^{1}**H NMR (400 MHz, CDCl$_3$)** δ, 8.38 (s, 1H), 7.84 (d, *J* = 8.2 Hz, 2H), 7.25 (d, *J* = 7.9 Hz, 2H), 7.21 – 7.15 (m, 1H), 7.01 (dd, *J* = 7.5, 1.3 Hz, 1H), 6.91 (d, *J* = 8.1 Hz, 1H), 6.84 (td, *J* = 7.5, 0.9 Hz, 1H), 5.46 (d, *J* = 9.6 Hz, 1H), 4.73 (s, 1H), 3.52 (dd, *J* = 18.2, 9.9 Hz, 1H), 3.38 (dd, *J* = 18.2, 2.8 Hz, 1H), 2.41 (s, 3H); ^{13}**C NMR (101 MHz, CDCl$_3$)** δ, 200.7, 155.9, 145.1, 133.7, 129.5 (2C), 129.2, 128.4 (2C), 126.7, 125.9, 120.0, 117.6, 71.8, 44.9, 21.7; **HRMS (ESI, *m/z*):** calculated for $C_{16}H_{16}O_3Na$ ($[M+Na]^+$): 279.0997; found: 279.0992; $[\alpha]_D^{25}$ = +10.968 (c = 0.155, $CHCl_3$, 90% ee). The enantiomeric ratio was determined by HPLC analysis using a Daicel Chiralpak OZ-3 column (Hexane/2-propanol = 85:15, flow rate 1.0 mL/min, λ = 254 nm), t_R = 12.9 min (major), t_R = 16.5 min (minor).

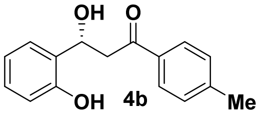

1-(4-(*tert*-Butyl)phenyl)-3-hydroxy-3-(2-hydroxyphenyl)propan-1-one (**4c**): 38.6 mg, 88% yield; R_f = 0.44 (30:70 = EtOAc/n-Hexane); yellow semi-solid; **FT-IR** (neat): 3415, 2361, 2335, 1652, 748 cm^{-1}; ^{1}**H NMR (400 MHz, CDCl$_3$)** δ, 8.41 (s, 1H), 7.89 (d, *J* = 8.5 Hz, 2H), 7.47 (d, *J* = 8.5 Hz, 2H), 7.22 – 7.15 (m, 1H), 7.02 (dd, *J* = 7.5, 1.2 Hz, 1H), 6.94 – 6.90 (m, 1H), 6.85 (td, *J* = 7.5, 0.9 Hz, 1H), 5.48 (dd, *J* = 9.7, 2.4 Hz, 1H), 4.75 (s, 1H), 3.54 (dd, *J* = 18.1, 9.8 Hz, 1H), 3.40 (dd, *J* = 18.1, 2.8 Hz, 1H), 1.33 (s, 9H); ^{13}**C NMR (101 MHz, CDCl$_3$)** δ, 200.7, 158.1, 155.9, 133.6, 129.2, 128.3 (2C), 126.7, 125.9, 125.8 (2C), 120.0, 117.6, 71.8, 44.9, 35.3, 31.0 (3C); **HRMS (ESI, *m/z*):** calculated for $C_{19}H_{22}O_3Na$ ($[M+Na]^+$): 321.1467; found: 321.1461; $[\alpha]_D^{24}$ = +46.829 (c = 0.205, $CHCl_3$, 99% ee). The enantiomeric ratio was determined by HPLC analysis using a Daicel Chiralpak OD-3 column (Hexane/2-propanol = 95:05, flow rate 1.0 mL/min, λ = 254 nm), t_R = 25.9 min (major).

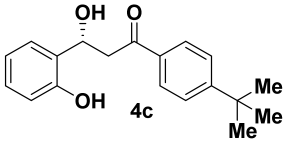

3-Hydroxy-3-(2-hydroxyphenyl)-1-(4-methoxyphenyl)propan-1-one (**4d**): 7.5 mg, 68% yield; R_f = 0.20 (30:70 = EtOAc/n-Hexane); Light yellow oil; **FT-IR** (neat): 3397, 2361, 2338, 1603, 745 cm^{-1}; ^{1}**H NMR (500 MHz,**

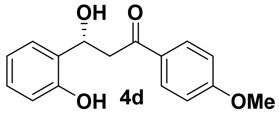

$CDCl_3$) δ, 8.46 (s, 1H), 7.98 – 7.95 (m, 2H), 7.23 (td, *J* = 8.2, 1.6 Hz, 1H), 7.04 (dd, *J* = 7.6, 1.5 Hz, 1H), 6.97 (d, *J* = 2.0 Hz, 1H), 6.97 – 6.93 (m, 2H), 6.89 (td, *J* = 7.5, 1.1 Hz, 1H), 5.49 (d, *J* = 9.9 Hz, 1H), 4.86 (d, *J* = 1.7 Hz, 1H), 3.91 (s, 3H), 3.52 (dd, *J* = 18.1, 10.0 Hz, 1H), 3.41 (dd, *J* = 18.1, 2.7 Hz, 1H; **^{13}C NMR (126 MHz, $CDCl_3$)** δ, 199.6, 164.3, 156.0, 130.6 (2C), 129.2, 129.2, 126.7, 125.8, 119.9, 117.6, 114.0 (2C), 72.0, 55.6, 44.5; **HRMS (ESI, *m/z*):** calculated for $C_{16}H_{16}O_4Na$ ($[M+Na]^+$): 295.0946; found: 295.0941; $[\alpha]_D^{25}$ = +21.515 (c = 0.165, $CHCl_3$, 90% ee).The enantiomeric ratio was determined by HPLC analysis using a Daicel Chiralpak OZ-3 column (Hexane/2-propanol = 85:15, flow rate 1.0 mL/min, λ = 254 nm), t_R = 24.5 min (major), t_R = 29.1 min (minor).

1-(3,4-Dimethoxyphenyl)-3-hydroxy-3-(2-hydroxyphenyl)propan-1-one (**4e**): 11 mg, 76% yield; R_f = 0.18 (30:70 = EtOAc/n-Hexane); brown solid; mp 87-89 °C; **FT-IR** (neat): 3406, 3059, 2338, 1615, 1260, 745 cm^{-1}; **^{1}H NMR (400 MHz, $CDCl_3$)** δ, 8.38 (s, 1H), 7.55 (dd, *J* = 8.4, 1.9 Hz, 1H), 7.49 (d, *J* = 1.9 Hz, 1H), 7.22 – 7.17 (m, 1H), 7.00 (dd, *J* = 7.5, 1.3 Hz, 1H), 6.91 (d, *J* = 7.6 Hz, 1H), 6.88 – 6.82 (m, 2H), 5.45 (d, *J* = 9.6 Hz, 1H), 4.76 (s, 1H), 3.93 (s, 3H), 3.92 (s, 3H), 3.50 (dd, *J* = 18.1, 9.9 Hz, 1H), 3.38 (dd, *J* = 18.1, 2.8 Hz, 1H); **^{13}C NMR (101 MHz, $CDCl_3$)** δ, 199.6, 156.0, 154.2, 149.2, 145.7, 129.3, 129.2, 126.7, 125.7, 123.3, 120.0, 117.6, 110.0, 72.1, 56.2, 56.1, 44.5; **HRMS (ESI, *m/z*):** calculated for $C_{17}H_{18}O_5Na$ ($[M+Na]^+$): 325.1052; found: 325.1046; $[\alpha]_D^{24}$ = +38.898 (c = 0.390, $CHCl_3$, 90% ee). The enantiomeric ratio was determined by HPLC analysis using a Daicel Chiralpak OZ-3 column (Hexane/2-propanol = 75:25, flow rate 1.0 mL/min, λ = 254 nm), t_R = 16.4 min (major), t_R = 24.9 min (minor).

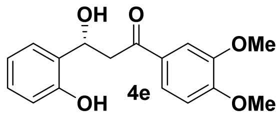

1-(Benzo[d][1,3]dioxol-5-yl)-3-hydroxy-3-(2-hydroxyphenyl)propan-1-one (**4f**): 11 mg, 75 % yield; R_f = 0.30 (30:70 = EtOAc/n-Hexane); Yellow solid; mp 109-111°C; **FT-IR** (neat): 3401,3059,2338,1618, 1265,744 cm^{-1}; **^{1}H NMR (400 MHz, $CDCl_3$)** δ, 8.34 (s, 1H), 7.52 (dd, *J* = 8.2, 1.4 Hz, 1H), 7.41 (d, *J* = 1.4 Hz, 1H), 7.22 – 7.14 (m, 1H), 6.99 (dd, *J* = 7.5, 1.1 Hz, 1H), 6.89 (d, *J* = 8.1 Hz, 1H), 6.83 (t, *J* = 8.0 Hz, 2H), 6.04 (s, 2H), 5.43 (dd, *J* = 9.8, 2.3 Hz, 1H), 4.70 (s, 1H), 3.46 (dd, *J* = 18.1, 9.9 Hz, 1H), 3.32 (dd, *J* = 18.1, 2.6 Hz, 1H); **^{13}C NMR (101 MHz, $CDCl_3$)** δ, 199.0, 155.9, 152.6, 148.4, 131.0, 129.2, 126.7, 125.7, 125.0, 120.0, 117.6, 108.1, 107.7, 102.1, 71.9, 44.7; **HRMS (ESI, *m/z*):** calculated for $C_{16}H_{14}O_5Na$ ($[M+Na]^+$):

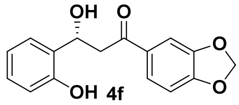

309.0739; found: 309.0733; $[\alpha]_D^{23}$ = +168.895(c = 0.190, $CHCl_3$, 84% ee). The enantiomeric ratio was determined by HPLC analysis using a Daicel Chiralpak OZ-3 column (Hexane/ 2-propanol = 85:15, flow rate 1.0 mL/min, λ = 254 nm), t_R = 25.8 min (major), t_R = 37.3 min (minor).

1-(4-Chlorophenyl)-3-hydroxy-3-(2-hydroxyphenyl)propan-1-one (**4g**): 14 mg, 85% yield; R_f = 0.46 (30:70 = EtOAc/n-Hexane); yellow semi-solid; **FT-IR** (neat): 3421, 2362, 2338, 1652, 747 cm^{-1}; **^{1}H NMR (500 MHz, CDCl$_3$)** δ, 8.24 (s, 1H), 7.87 (dd, *J* = 8.6, 2.4 Hz, 2H), 7.68 (dd, *J* = 8.5, 2.5 Hz, 2H), 7.26 – 7.19 (m, 1H), 7.04 (dd, *J* = 7.6, 1.4 Hz, 1H), 6.94 (d, *J* = 8.2 Hz, 1H), 6.89 (t, *J* = 7.4 Hz, 1H), 5.50 (dd, *J* = 9.9, 2.2 Hz, 1H), 4.50 (s, 1H), 3.56 (dd, *J* = 18.3, 9.9 Hz, 1H), 3.38 (dt, *J* = 18.2, 2.7 Hz, 1H; **δ^{13}C NMR (126 MHz, CDCl$_3$)** δ, 199.6, 155.7, 140.6, 134.5, 129.6(2C), 129.4, 129.2(2C), 126.7, 125.6, 120.1, 117.6, 71.6, 45.1; **HRMS (ESI, *m/z*):** calculated for $C_{15}H_{13}ClO_3Na$ ($[M+Na]^+$): 299.0451; found: 299.0445; $[\alpha]_D^{25}$ = +36.374 (c = 0.535, $CHCl_3$, 90% ee). The enantiomeric ratio was determined by HPLC analysis using a Daicel Chiralpak OZ-3 column (Hexane/2-propanol = 85:15, flow rate 1.0 mL/min, λ = 254 nm), t_R = 10.2 min (major), t_R = 13.9 min (minor).

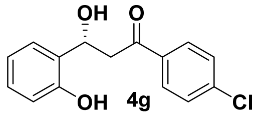

1-(4-Bromophenyl)-3-hydroxy-3-(2-hydroxyphenyl)propan-1-one (**4h**): 20 mg, 80% yield; R_f = (30:70 = EtOAc/n-Hexane); whitish solid; mp 89-91 °C; **FT-IR** (neat): 3398, 2928, 2334, 2360, 1655, 1262, 750 cm^{-1}; **^{1}H NMR (500 MHz, CDCl$_3$)** δ, 8.24 (s, 1H), 7.86 – 7.82 (m, 2H), 7.68 – 7.63 (m, 2H), 7.24 (td, *J* = 8.2, 1.7 Hz, 1H), 7.05 (dd, *J* = 7.6, 1.6 Hz, 1H), 6.95 (dd, *J* = 8.2, 1.0 Hz, 1H), 6.89 (td, *J* = 7.4, 1.1 Hz, 1H), 5.51 (dt, *J* = 9.9, 2.4 Hz, 1H), 4.50 (d, *J* = 2.4 Hz, 1H), 3.58 (dd, *J* = 18.3, 9.9 Hz, 1H), 3.40 (dd, *J* = 18.3, 2.7 Hz, 1H); **^{13}C NMR (126 MHz, CDCl$_3$)** δ, 199.8, 155.8, 134.8, 132.2 (2C), 129.7 (2C), 129.4, 129.4, 126.7, 125.6, 120.1, 117.7, 71.6, 45.0; **HRMS (ESI, *m/z*):** calculated for $C_{15}H_{13}BrO_3Na$ ($[M+Na]^+$): 342.9946; found: 342.9940; $[\alpha]_D^{25}$ = +31.600 (c = 1.000, $CHCl_3$, 92% ee). The enantiomeric ratio was determined by HPLC analysis using a Daicel Chiralpak OZ-3 column (Hexane/2-propanol = 90:10, flow rate 1.0 mL/min, λ = 254 nm), t_R = 18.2 min (major), t_R = 24.6 min (minor).

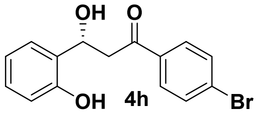

3-Hydroxy-3-(2-hydroxyphenyl)-1-(4-iodophenyl)propan-1-one (**4i**): 12 mg, 78% yield; R_f = 0.44 (30:70 = EtOAC/n-Hexane); yellow solid; mp

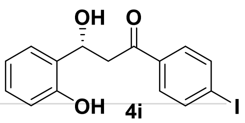

108-110 °C; **FT-IR** (neat): 3401, 2924, 2334, 2358, 1651, 1265, 752, 465 cm^{-1}; **^{1}H NMR (500 MHz, $CDCl_3$)** δ, 8.24 (s, 1H), 7.87 (dd, *J* = 8.6, 2.4 Hz, 2H), 7.68 (dd, *J* = 8.5, 2.5 Hz, 2H), 7.26 – 7.19 (m, 1H), 7.04 (dd, *J* = 7.6, 1.4 Hz, 1H), 6.94 (d, *J* = 8.2 Hz, 1H), 6.89 (t, *J* = 7.4 Hz, 1H), 5.50 (dd, *J* = 9.9, 2.2 Hz, 1H), 4.50 (s, 1H), 3.56 (dd, *J* = 18.3, 9.9 Hz, 1H), 3.38 (dt, *J* = 18.2, 2.7 Hz, 1H); **^{13}C NMR (126 MHz, $CDCl_3$)** δ, 200.2, 155.8, 138.2(2C), 135.4, 129.5(2C), 129.4, 126.7, 125.6, 120.1, 117.6, 102.4, 71.6, 45.0; **HRMS (ESI, *m/z*):** calculated for $C_{15}H_{13}IO_3Na$ ($[M+Na]^+$): 390.9807; found: 390.9802; $[\alpha]_D^{25}$ = +15.382 (c = 0.275, $CHCl_3$, 90% ee). The enantiomeric ratio was determined by HPLC analysis using a Daicel Chiralpak OZ-3 column (Hexane/2-propanol = 90:10, flow rate 1.0 mL/min, λ = 254 nm), t_R = 20.3 min (major), t_R = 26.5 min (minor).

1-(4-Fluorophenyl)-3-hydroxy-3-(2-hydroxyphenyl)propan-1-one (**4j**): 10.5 mg, 73% yield; R_f = 0.44 (30:70 = EtOAc/n-Hexane); Light yellow semi-solid; **FT-IR** (neat): 3422, 2923,1652,1454,750 cm^{-1};**^{1}H NMR (500 MHz, $CDCl_3$)** δ, 8.28 (s, 1H), 8.08 – 7.96 (m, 2H), 7.27 – 7.21 (m, 1H), 7.18 (t, *J* = 8.6 Hz, 2H), 7.05 (dd, *J* = 7.6, 1.6 Hz, 1H), 6.95 (dd, *J* = 8.2, 1.0 Hz, 1H), 6.89 (td, *J* = 7.5, 1.1 Hz, 1H), 5.51 (dd, *J* = 9.9, 2.5 Hz, 1H), 4.57 (s, 1H), 3.58 (dd, *J* = 18.2, 10.0 Hz, 1H), 3.42 (dd, *J* = 18.2, 2.7 Hz, 1H); **^{13}C NMR (126 MHz, $CDCl_3$)** δ, 199.3, 166.3 (d, *J* = 256.6 Hz), 155.9, 132.6 (d, *J* = 3.1 Hz), 131.0, 130.9, 129.3, 126.7, 125.6, 120.1, 117.7, 116.1, 116.0, 71.8, 45.0.; **HRMS (ESI, *m/z*):** calculated for $C_{15}H_{13}FO_3Na$ ($[M+Na]^+$): 283.0747; found: 283.0741; $[\alpha]_D^{25}$ = +42.245 (c = 0.570, $CHCl_3$, 91% ee).

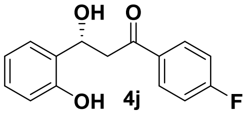

4j

The enantiomeric ratio was determined by HPLC analysis using a Daicel Chiralpak OZ-3 column (Hexane/2-propanol = 85:15, flow rate 1.0 mL/min, λ = 254 nm), t_R = 9.7 min (major), t_R = 13.3 min (minor).

3-Hydroxy-3-(2-hydroxyphenyl)-1-(4-(trifluoromethyl)phenyl)propan-1-one (**4k**): 17.5 mg, 83% yield; R_f = 0.46 (30:70 = EtOAc/n-Hexane); yellowish solid; mp 81-83 °C; **FT-IR** (neat): 3405, 2924, 2336, 2361, 1667, 1261, 755 cm^{-1}; **^{1}H NMR (500 MHz, $CDCl_3$)** δ, 8.14 (s, 1H), 8.09 (d, *J* = 8.1 Hz, 2H), 7.77 (d, *J* = 8.2 Hz, 2H), 7.24 (td, *J* = 8.2, 1.6 Hz, 1H), 7.06 (dd, *J* = 7.6, 1.5 Hz, 1H), 6.95 (dd, *J* = 8.1, 0.9 Hz, 1H), 6.90 (td, *J* = 7.5, 1.1 Hz, 1H), 5.54 (dt, *J* = 9.7, 2.2 Hz, 1H), 4.37 (d, *J* = 2.4 Hz, 1H), 3.65 (dd, *J* = 18.3, 9.8 Hz, 1H), 3.45 (dd, *J* = 18.3, 2.7 Hz, 1H).**^{13}C NMR (126 MHz, $CDCl_3$)** δ, 199.7, 155.7, 138.7, 135.6 (qt, *J* = 65.6 Hz), 130.0,

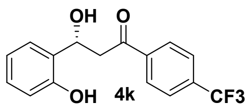

4k

129.4, 128.6, 126.7, 126.1, 125.9 (qt, J = 7.4 Hz), 125.4 (qt J = 7.4 Hz), 123.4 (qt, J = 272.8 Hz) 120.2, 117.6, 71.5, 45.5; **HRMS (ESI, *m/z*):** calculated for $C_{16}H_{13}F_3O_3Na$ ($[M+Na]^+$): 333.0714; found: 307.0709; $[\alpha]_D^{25}$ = +14.788 (c = 0.825, $CHCl_3$, 74% ee). The enantiomeric ratio was determined by HPLC analysis using a Daicel Chiralpak IC-3 column (Hexane/ 2-propanol = 95:05, flow rate 1.0 mL/min, λ = 254 nm), t_R = 19.5 min (minor), t_R = 23.4 min (major).

1-([1,1'-Biphenyl]-4-yl)-3-hydroxy-3-(2-hydroxyphenyl)propan-1-oneone (**4l**): 27 mg, 85% yield; R_f = 0.38 (30:70 = EtOAc/n-Hexane); white solid; mp 122-124 °C ; **FT-IR** (neat): 3364, 2923, 2359, 1651, 1263, 736 cm^{-1}; **^{1}H NMR (500 MHz, CDCl$_3$)** δ, 8.39 (s, 1H), 8.06 (d, J = 8.5 Hz, 2H), 7.73 (d, J = 8.5 Hz, 2H), 7.66 (dd, J = 5.3, 3.3 Hz, 2H), 7.53 – 7.48 (m, 2H), 7.45 (ddd, J = 7.3, 3.7, 1.2 Hz, 1H), 7.25 (td, J = 8.2, 1.6 Hz, 1H), 7.08 (dd, J = 7.6, 1.4 Hz, 1H), 6.97 (dd, J = 8.2, 0.9 Hz, 1H), 6.91 (td, J = 7.5, 1.1 Hz, 1H), 5.55 (dt, J = 9.9, 2.2 Hz, 1H), 4.72 (d, J = 2.2 Hz, 1H), 3.64 (dd, J = 18.2, 10.0 Hz, 1H), 3.49 (dd, J = 18.2, 2.6 Hz, 1H); **^{13}C NMR (126 MHz, CDCl$_3$)** δ, 200.6, 156.0, 146.8, 139.5, 134.8, 129.3, 129.0 (2C), 128.9 (2C), 128.5, 127.4 (2C), 127.3 (2C), 126.7, 125.7, 120.0, 117.7, 71.9, 45.0; **HRMS (ESI, *m/z*):** calculated for $C_{21}H_{18}O_3Na$ ($[M+Na]^+$): 341.1154; found: 341.1148; $[\alpha]_D^{24}$ = +37.191 (c = 0.890, $CHCl_3$, 90% ee).

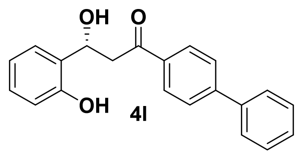

The enantiomeric ratio was determined by HPLC analysis using a Daicel Chiralpak IC-3 column (Hexane/2-propanol = 90:10, flow rate 1.0 mL/min, λ = 254 nm), t_R = 31.4 min (major), t_R = 39.8 min (minor).

3-Hydroxy-3-(2-hydroxyphenyl)-1-(thiophen-2-yl)propan-1-one (**4m**): 11 mg, 71% yield; R_f = 0.32 (30:70 = EtOAc/n-Hexane); Yellow stiky liquid; mp 69-71 °C; **FT-IR** (neat): 3397, 2361, 2338, 1651, 1265, 746 cm^{-1}; **^{1}H NMR (400 MHz, CDCl$_3$)** δ, 8.28 (s, 1H), 7.71 (t, J = 4.4 Hz, 2H), 7.22 – 7.17 (m, 1H), 7.13 (dd, J = 4.8, 4.0 Hz, 1H), 7.01 (dd, J = 7.5, 1.3 Hz, 1H), 6.90 (d, J = 8.1 Hz, 1H), 6.85 (td, J = 7.5, 0.9 Hz, 1H), 5.46 (dd, J = 10.0, 2.1 Hz, 1H), 4.60 (s, 1H), 3.51 (dd, J = 17.8, 10.0 Hz, 1H), 3.35 (dd, J = 17.8, 2.7 Hz, 1H); **^{13}C NMR (101 MHz, CDCl$_3$)** δ, 193.5, 155.9, 143.2, 135.1, 133.2, 129.3, 128.5, 126.7, 125.5, 120.0, 117.7, 71.9, 45.4; **HRMS (ESI, *m/z*):** calculated for $C_{13}H_{12}O_3SNa$ ($[M+ Na]^+$): 271.0405; found: 271.0399; $[\alpha]_D^{25}$ = +40.606 (c = 0.660, $CHCl_3$, 94% ee). The enantiomeric ratio was determined by HPLC analysis using a

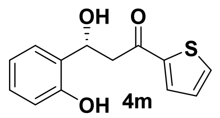

Daicel Chiralpak OZ-3 column (Hexane/2-propanol = 85:15, flow rate 1.0 mL/min, λ = 254 nm), t_R = 13.0 min (major), t_R = 16.9 min (minor).

1-(Furan-2-yl)-3-hydroxy-3-(2-hydroxyphenyl)propan-1-one (**4n**): 6.6 mg, 69% yield; R_f = 0.19 (30:70 = EtOAc/n-Hexane); light green semi-solid; **FT-IR** (neat): 3371, 2923, 2848, 2334, 1653,757 cm^{-1}; ^{1}H **NMR (400 MHz, $CDCl_3$)** δ, 8.26 (s, 1H), 7.60 (d, *J* = 0.9 Hz, 1H), 7.25 (d, *J* = 3.8 Hz, 1H), 7.22 – 7.14 (m, 1H), 7.00 (dd, *J* = 7.5, 1.3 Hz, 1H), 6.89 (d, *J* = 8.1 Hz, 1H), 6.84 (dd, *J* = 10.7, 4.1 Hz, 1H), 6.56 (dd, *J* = 3.6, 1.6 Hz, 1H), 5.45 (dd, *J* = 9.9, 2.6 Hz, 1H), 4.51 (s, 1H), 3.42 (dd, *J* = 17.9, 10.0 Hz, 1H), 3.29 (dd, *J* = 17.9, 2.9 Hz, 1H); **^{13}C NMR (101 MHz, $CDCl_3$)** δ, 189.3, 155.9, 152.0, 147.4, 129.3, 126.7, 125.5, 120.0, 118.6, 117.6, 112.7, 71.7, 44.5; **HRMS (ESI, *m/z*):** calculated for $C_{13}H_{12}O_4Na$ ($[M+Na]^+$): 255.0633; found: 255.0628; $[\alpha]_D^{25}$ = +22.214 (c = 0.140, $CHCl_3$, 92% ee).

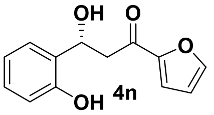

The enantiomeric ratio was determined by HPLC analysis using a Daicel Chiralpak OZ-3 column (Hexane/2-propanol = 85:15, flow rate 1.0 mL/min, λ = 254 nm), t_R = 17.9 min (major), t_R = 20.6 min (minor).

3-Hydroxy-3-(2-hydroxy-4-methylphenyl)-1-(thiophen-2-yl)propan-1-one (**4o**): 7.5 mg, 86% yield; R_f = 0.38 (30:70 = EtOAc/n-Hexane); Light yellow solid; mp 88-90 °C; **FT-IR** (neat): 3389, 2363, 2337, 1649, 1258, 747 cm^{-1}; **^{1}H NMR (400 MHz, $CDCl_3$)** δ, 8.19 (s, 1H), 7.70 (dd, *J* = 4.1, 3.2 Hz, 2H), 7.13 (dd, *J* = 4.8, 4.0 Hz, 1H), 6.88 (d, *J* = 7.7 Hz, 1H), 6.73 (s, 1H), 6.66 (d, *J* = 7.7 Hz, 1H), 5.43 (d, *J* = 9.8 Hz, 1H), 4.54 (s, 1H), 3.49 (dd, *J* = 17.8, 10.0 Hz, 1H), 3.33 (dd, *J* = 17.8, 2.7 Hz, 1H), 2.29 (s, 3H); **^{13}C NMR (101 MHz, $CDCl_3$)** δ, 193.6, 155.8, 143.2, 139.5, 135.0, 133.1, 128.5, 126.5, 122.6, 120.8, 118.2, 71.8, 45.6, 21.1; **HRMS (ESI, *m/z*):** calculated for $C_{14}H_{14}O_3SNa$ ($[M+Na]^+$): 285.0562; found: 285.0556; $[\alpha]_D^{23}$ = +25.318 (c = 0.055, $CHCl_3$, 94% ee). The enantiomeric ratio was determined by HPLC analysis using a Daicel Chiralpak OZ-3 column (Hexane/2-propanol = 85:15, flow rate 1.0 mL/min, λ = 254 nm), t_R = 12.6 min (major), t_R = 15.9 min (minor).

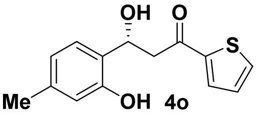

1-(4-(*tert*-Butyl)phenyl)-3-hydroxy-3-(2-hydroxy-4-methylphenyl)propan-1-one (**4p**): 13 mg, 88% yield; R_f = 0.58 (30:70 = EtOAc/n-Hexane); Light yellow liquid; **FT-IR** (neat): 3420,

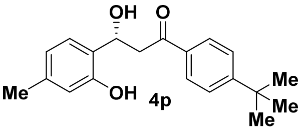

2359, 2334, 1650, 747 cm^{-1}; **^{1}H NMR (400 MHz, CDCl$_3$)** δ, 8.29 (s, 1H), 7.88 (d, *J* = 8.5 Hz, 2H), 7.47 (d, *J* = 8.5 Hz, 2H), 6.88 (d, *J* = 7.7 Hz, 1H), 6.74 (s, 1H), 6.66 (d, *J* = 7.7 Hz, 1H), 5.43 (d, *J* = 9.7 Hz, 1H), 4.66 (d, *J* = 1.9 Hz, 1H), 3.51 (dd, *J* = 18.2, 9.8 Hz, 1H), 3.39 (dd, *J* = 18.2, 2.8 Hz, 1H), 2.29 (s, 3H), 1.33 (s, 9H); **^{13}C NMR (101 MHz, CDCl$_3$)** δ, 200.8, 158.1, 155.8, 139.4, 133.6, 128.2 (2C), 126.5, 125.8 (2C), 122.8, 120.7, 118.2, 71.8, 44.9, 35.2, 31.0 (3C), 21.1; **HRMS (ESI, *m/z*):** calculated for $C_{20}H_{24}O_3Na([M+Na]^+)$: 335.1623; found: 335.1618; $[\alpha]_D^{24}$ = +2.222(c = 0.045, CHCl$_3$, 85% ee). The enantiomeric ratio was determined by HPLC analysis using a Daicel Chiralpak OD-3 column (Hexane/ 2-propanol = 80:20, flow rate 1.0 mL/min, λ = 254 nm), t_R = 6.0 min (minor), t_R = 14.8 min (major).

1-(4-(*tert*-Butyl)phenyl)-3-hydroxy-3-(2-hydroxy-4-methoxyphenyl)propan-1-one (**4q**): 20 mg, 61 % yield; R_f = 0.40 (30:70 = EtOAc/n-Hexane); colourless liquid ; **FT-IR** (neat): 3398 , 2338, 1650 , 1147, 746 cm^{-1}; **^{1}H NMR (500 MHz, CDCl$_3$)** δ, 7.92 (d, *J* = 8.6 Hz, 2H), 7.51 (d, *J* = 8.6 Hz, 2H), 6.88 (d, *J* = 8.8 Hz, 1H), 6.79 (dd, *J* = 8.8, 3.0 Hz, 1H), 6.63 (d, *J* = 3.0 Hz, 1H), 5.45 (dd, *J* = 9.7, 2.6 Hz, 1H), 4.64 (s, 1H), 3.77 (s, 3H), 3.57 (dd, *J* = 18.1, 9.8 Hz, 1H), 3.45 (dd, *J* = 18.1, 2.8 Hz, 1H), 1.37 (s, 9H). **^{13}C NMR (126 MHz, CDCl$_3$)** δ, 200.7, 158.1, 153.1, 149.6, 133.6, 128.3 (2C), 126.6, 125.8 (2C), 118.2, 114.2, 112.3, 71.5, 55.8, 44.8, 35.3, 31.0 (3C); **HRMS (ESI, *m/z*):** calculated for $C_{20}H_{24}O_4Na$ ($[M+Na]^+$): 351.1572; found: 351.1567; $[\alpha]_D^{25}$ = +46.520 (c = 1.010, CHCl$_3$, 90% ee). The enantiomeric ratio was determined by HPLC analysis using a Daicel Chiralpak OD-3 column (Hexane/ 2-propanol = 80:20, flow rate 1.0 mL/min, λ = 254 nm), t_R = 9.1 min (minor), t_R = 14.9 min (major).

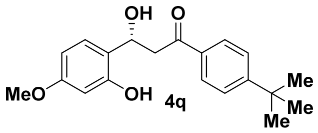

3-(5-Fluoro-2-hydroxyphenyl)-3-hydroxy-1-(thiophen-2-yl)propan-1-one (**4r**): 12 mg, 62% yield; R_f = 0.64 (30:70 = EtOAc/n-Hexane); yellowish oil; **FT-IR** (neat): 3398, 2365, 2338, 1653, 1261, 748 cm^{-1}; **^{1}H NMR (500 MHz, CDCl$_3$)** δ, 8.04 (s, 1H), 7.77 (ddd, *J* = 6.0, 4.4, 1.1 Hz, 2H), 7.19 (dd, *J* = 4.9, 3.9 Hz, 1H), 6.96 – 6.91 (m, 1H), 6.88 (dd, *J* = 8.9, 4.8 Hz,1H), 6.79 (dd, *J* = 8.8, 3.0 Hz, 1H), 5.45 (dd, *J* = 9.9, 2.8 Hz, 1H), 4.65 (s, 1H), 3.53 (dd, *J* = 17.8, 9.9 Hz, 1H), 3.40 (dd, *J* = 17.8, 2.9 Hz, 1H); **^{13}C NMR (126 MHz, CDCl$_3$)** δ, 193.2, 156.5 (d, *J* = 237.9 Hz), 151.7 (d, *J* = 2.1 Hz), 143.0, 135.3, 133.3, 128.5, 126.5 (d, *J* = 6.6 Hz), 118.5(d, *J* = 7.9 Hz), 115.6 (d, *J* = 22.8 Hz), 113.1 (d, *J* = 23.9 Hz), 71.2 (d, *J* = 1.4 Hz), 45.1; **HRMS (ESI, *m/z*):** calculated for $C_{13}H_{11}FO_3SNa$ ($[M+Na]^+$): 289.0311; found: 289.0305; $[\alpha]_D^{23}$ = +36.000 (c =

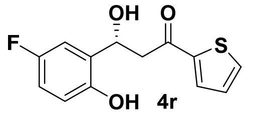

0.050, $CHCl_3$, 94% ee). The enantiomeric ratio was determined by HPLC analysis using a Daicel Chiralpak IE-3 column (Hexane/2-propanol = 85:15, flow rate 1.0 mL/min, λ = 254 nm), t_R = 17.5 min (minor), t_R = 19.7 min (major).

3-(2-Fluoro-6-hydroxyphenyl)-3-hydroxy-1-(thiophen-2-yl)propan-1-one (**4s**): 13 mg, 58% yield; R_f = 0.41 (30:70 = EtOAc/n-Hexane); colourless liquid; **FT-IR** (neat): 3399, 2360, 2338, 1651, 1264, 746 cm^{-1}; **^{1}H NMR (400 MHz, $CDCl_3$)** δ, 8.97 (s, 1H), 7.75 – 7.71 (m, 2H), 7.16 – 7.10 (m, 2H), 6.69 (d, *J* = 8.3 Hz, 1H), 6.60 – 6.52 (m, 1H), 5.82 (dd, *J* = 12.2, 5.9 Hz, 1H), 4.93 (s, 1H), 3.41 (dd, *J* = 12.9, 11.9 Hz, 1H), 3.37 (dd, *J* = 19.8, 7.7 Hz, 1H); **^{13}C NMR (101 MHz, $CDCl_3$)** δ, 193.5, 159.3 (d, *J* = 244.1 Hz), 157.8 (d, *J* = 5.8 Hz), 143.0, 135.3, 133.4, 129.5 (d, *J* = 10.9 Hz), 128.5, 113.5 (d, *J* = 3.0 Hz), 113.0 (d, *J* = 15.9 Hz), 106.5 (d, *J* = 22.1 Hz), 66.5 (d, *J* = 6.2 Hz), 44.6; **HRMS (ESI, *m/z*)**: calculated for $C_{13}H_{11}FO_3SNa$ ($[M+Na]^+$): 289.0311; found: 289.0305; $[\alpha]_D^{23}$ = +68.342 (c = 0.175, $CHCl_3$, 92% ee).The enantiomeric ratio was determined by HPLC analysis using a Daicel Chiralpak OD-3 column (Hexane/2-propanol = 95:05, flow rate 1.0 mL/min, λ = 254 nm), t_R = 21.3 min (minor), t_R = 26.6 min (major).

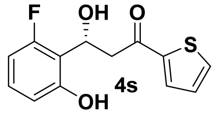

1-(4-(*tert*-Butyl)phenyl)-3-(2-fluoro-6-hydroxyphenyl)-3-hydroxypropan-1-one (**4t**): 21 mg, 67 % yield; R_f = 0.50 (30:70 = EtOAc/n-Hexane); colourless oil; **FT-IR (neat):** 3406, 2366, 1644, 1233, 749 cm^{-1}; **^{1}H NMR (500 MHz, $CDCl_3$)** δ, 9.05 (s, 1H), 8.00 – 7.87 (m, 2H), 7.55 – 7.50 (m, 2H), 7.17 (td, *J* = 8.3, 6.7 Hz, 1H), 6.73 (d, *J* = 8.3 Hz, 1H), 6.66 – 6.57 (m, 1H), 5.87 (d, *J* = 9.9 Hz, 1H), 5.07 (s, 1H), 3.50 (dd, *J* = 18.1, 2.8 Hz, 1H), 3.44 (dd, *J* = 18.1, 9.8 Hz, 1H), 1.37 (s, 9H).**^{13}C NMR (126 MHz, $CDCl_3$)** δ, 200.8, 159.4(d, *J* = 244.0 Hz), 158.3, 157.8 (d, *J* = 5.8 Hz), 133.4, 129.4 (d, *J* = 10.9 Hz), 128.3 (2C), 125.8 (2C), 113.4 (d, *J* = 29.0 Hz), 113.2 (d, *J* = 15.9 Hz), 106.44 (d, *J* = 22.0 Hz), 66.5 (d, *J* = 6.0 Hz), 43.6, 35.1, 31.0 (3C); **HRMS (ESI, *m/z*):** calculated for $C_{19}H_{21}FO_3Na$ ($[M+Na]^+$): 339.1383; found: 339.1367; $[\alpha]_D^{24}$ = +42.580 (c = 0.730, $CHCl_3$, 84% ee). The enantiomeric ratio was determined by HPLC analysis using a Daicel Chiralpak OD-3 column (Hexane/ 2-propanol = 90:05, flow rate 1.0 mL/min, λ = 254 nm), t_R = 7.9 min (minor), t_R = 20.9 min (major).

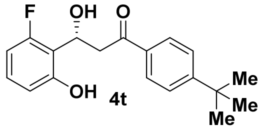

3-Hydroxy-4-(1-hydroxy-3-oxo-3-(thiophen-2-yl)propyl)benzonitrile (**4u**): 6 mg, 62 % yield; R_f = 0.20 (30:70 = EtOAc/n-Hexane); Light yellow liquid; **FT-IR** (neat): 3395, 2348, 2328, 2207, 1651, 1265, 748 cm^{-1}; **^{1}H NMR (500 MHz, CDCl$_3$)** δ, 8.77 (s, 1H), 7.77 (dt, *J* = 3.6, 1.1 Hz, 2H), 7.22 – 7.20 (m, 1H), 7.20 – 7.17 (m, 2H), 7.14 (d, *J* = 7.8 Hz, 1H), 5.55 (dt, *J* = 9.9, 2.4 Hz, 1H), 4.83 (d, *J* = 2.2 Hz, 1H), 3.50 (dd, *J* = 17.8, 10.0 Hz, 1H), 3.38 (dd, *J* = 17.8, 2.8 Hz, 1H); **^{13}C NMR (126 MHz, CDCl$_3$)** δ, 192.8, 156.4, 142.8, 135.6, 133.4, 130.6, 128.6, 127.6, 123.7, 121.2, 118.4, 112.9, 71.7, 44.9; **HRMS (ESI, *m/z*):** calculated for $C_{14}H_{11}NO_3SNa$ ([M+Na]$^+$): 296.0357; found: 296.0352; $[\alpha]_D^{24}$ = +28.785 (c = 0.140, CHCl$_3$, 98% ee). The enantiomeric ratio was determined by HPLC analysis using a Daicel Chiralpak OJ column (Hexane/ 2-propanol = 85:15, flow rate 1.0 mL/min, λ = 254 nm), t_R = 21.1 min (minor), t_R = 26.4 min (major).

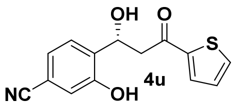

1-(4-(*tert*-Butyl)phenyl)-3-(5-chloro-2-hydroxyphenyl)-3-hydroxypropan-1-one (**4v**): 12 mg, 53% yield; R_f = 0.37 (30:70 = EtOAc/n-Hexane); white solid; mp 118-120 °C ; **FT-IR** (neat): 3401, 2356, 1648, 1263, 748 cm^{-1} ; **^{1}H NMR (400 MHz, CDCl$_3$)** δ, 8.38 (s, 1H), 7.88 (d, *J* = 8.5 Hz, 2H), 7.48 (d, *J* = 8.5 Hz, 2H), 7.14 (dd, *J* = 8.7, 2.5 Hz, 1H), 6.99 (d, *J* = 2.5 Hz, 1H), 6.83 (d, *J* = 8.7 Hz, 1H), 5.41 (d, *J* = 8.1 Hz, 1H), 4.75 (d, *J* = 1.1 Hz, 1H), 3.48 (dd, *J* = 18.2, 9.4 Hz, 1H), 3.40 (dd, *J* = 18.2, 3.3 Hz, 1H), 1.33 (s, 9H); **^{13}C NMR (101 MHz, CDCl$_3$)** δ, 200.5, 158.3, 154.6, 133.4, 129.0, 128.3(2C), 127.2, 126.5, 125.8(2C), 124.6, 119.0, 71.3, 44.6, 35.3, 31.0(3C); **HRMS (ESI, *m/z*):** calculated for $C_{19}H_{22}ClO_3$ ([M+H]$^+$): 333.1257; found: 333.1252; $[\alpha]_D^{25}$ = +88.057 (c = 0.650, CHCl$_3$, 87% ee). The enantiomeric ratio was determined by HPLC analysis using a Daicel Chiralpak OD-3 column (Hexane/ 2-propanol = 80:20, flow rate 1.0 mL/min, λ = 254 nm), t_R = 5.7 min (minor), t_R = 14.4 min (major).

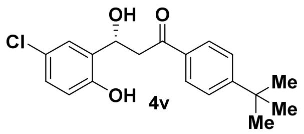

1-([1,1'-Biphenyl]-4-yl)-3-hydroxy-3-(3-hydroxynaphthalen-2-yl)propan-1-one (**4w**): 9 mg, 72% yield; R_f = 0.46 (30:70 = EtOAc/n-Hexane); Light yellow semi-solid, **FT-IR** (neat): 3404, 2335, 2357, 1660, 1263, 748 cm^{-1}; **^{1}H NMR (400 MHz, CDCl$_3$)** δ, 9.24 (s, 1H), 8.36 – 8.24 (m, 1H), 8.02 (d, *J* = 8.4 Hz, 2H), 7.79 – 7.72 (m, 1H), 7.68 (d, *J* = 8.4 Hz, 2H), 7.63 – 7.54 (m, 2H), 7.50 – 7.43 (m, 4H), 7.41 (d, *J* = 7.2 Hz, 1H), 7.36 (d, *J* = 8.4 Hz, 1H), 7.08 (d, *J* = 8.4 Hz, 1H), 5.65 (dd, *J* = 7.5, 2.1 Hz, 1H), 4.80 (d, *J* = 1.6 Hz, 1H), 3.63 (dd, *J* = 18.3, 9.7 Hz, 1H), 3.52 (dd, *J* = 18.3, 2.8 Hz, 1H). **^{13}C NMR (101 MHz, CDCl$_3$)** δ, 200.7, 151.7, 146.8,

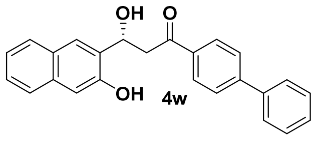

139.5, 134.7, 134.0, 129.0(2C), 128.9(2C), 128.5, 127.4(2C), 127.3(2C), 127.3, 126.5, 125.8, 125.3, 124.3, 122.2, 119.5, 118.2, 72.5, 45.3; **HRMS (ESI, *m/z*):** calculated for $C_{25}H_{20}O_3Na$ ($[M+Na]^+$): 391.1310; found: 391.1305; $[\alpha]_D^{24}$ = -25.100 (c = 0.440, $CHCl_3$, 58% ee). The enantiomeric ratio was determined by HPLC analysis using a Daicel Chiralpak OD-3 column (Hexane/ 2-propanol = 60:40, flow rate 1.0 mL/min, λ = 254 nm), t_R = 18.2 min (minor), t_R = 30.2 min (major).

Procedure for deborylation-phenylation of compound 3c:

To a solution of **3c** (1equiv. 0.1 mmol) and bromobenzene (1.1equiv. o.11 mmol) in dry 1,4-dioxane was taken and degassing the reaction mixture with N_2 gas up to 1 hour. Under N_2 atmosphere $Pd(PPh_3)_4$ (0.02equiv.) and K_2CO_3 (2 equiv.) was then added. The reaction mixture was heated at 80°C .After completion of substrate **3c** (monitored by TLC), the reaction mixture was pass through a plug of silica gel and purified by column chromatography by using hexane/EtOAc as eluent. The obtained product **5** was characterised as follows.

3-([1,1'-Biphenyl]-2-yl)-1-(4-(tert-butyl)phenyl)-3-hydroxypropan-1-one (**5**): 14.3 mg, 40% yield; R_f = 0.45 (30:70 = EtOAc/n-Hexane); white solid; mp 79-81 °C; **FT-IR** (neat): 3398, 2927, 1651, 1263, 1121, 1033, 746 cm^{-1}; **^{1}H NMR (400 MHz, CDCl$_3$)** δ, 7.70 (d, *J* = 8.5 Hz, 3H), 7.42 (ddd, *J* = 18.4, 11.1, 6.3 Hz, 6H), 7.35 – 7.31 (m, 3H), 7.23 (dd, *J* = 7.6, 1.1 Hz, 1H), 5.43 (dt, *J* = 9.4, 2.5 Hz, 1H), 3.62 (d, *J* = 2.7 Hz, 1H), 3.21 (dd, *J* = 17.3, 2.7 Hz, 1H), 3.12 (dd, *J* = 17.4, 9.4 Hz, 1H), 1.31 (s, 9H); **^{13}C NMR (101 MHz, CDCl$_3$)** δ,

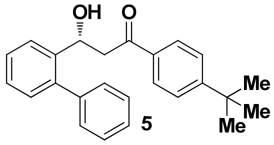

5

199.7, 157.4, 140.8, 140.3, 140.2, 133.8, 130.1, 129.2 (2C), 128.4 (2C), 128.1 (2C), 128.0, 127.4, 127.2, 126.1, 125.6 (2C), 66.7, 46.3, 35.2, 31.0 (3C); **HRMS (ESI, *m/z*):** calculated for $C_{25}H_{26}O_2Na$ ($[M+Na]^+$): 381.1831; found: 381.1825; $[\alpha]_D^{25}$ = +85.854 (c = 0.205, $CHCl_3$, 91% ee). The enantiomeric ratio was determined by HPLC analysis using a Daicel Chiralpak OD-3 column (Hexane/2-propanol = 90:10, flow rate 1.0 mL/min, λ = 254 nm), t_R = 16.1 min (minor), t_R = 19.4 min (major).

Procedure for deborylation-protonation of compound 3c:

In a 2 mL round bottom flask substrate **3c** (1 euqiv., 0.14 mmol) and $AgNO_3$ (0.1equiv., 0.014 mmol) in 2 mL (EtOH: H_2O = 1:1) as a solvent and Et_3N (0.1equiv., 0.014) was taken, followed by stirring the reaction mixture up to 1 hour. Then organic layer extracted with EtOAc (3x10 mL) and brine water also added during extraction and the residue obtained purified by column chromatography by hexane/EtOAc as eluent. The obtained product **6** was characterised as follows.

Caution: On keeping the reaction in longer time, there is a possibility of decrease of % ee of the product due to racemization

1-(4-(*tert*-Butyl)phenyl)-3-hydroxy-3-phenylpropan-1-one (**6**) : 26 mg, 65% yield; R_f = 0.58 (30:70 = EtOAc/n-Hexane); white solid; mp 47-49 °C; **FT-IR** (neat): 3401, 2362, 2332, 1656, 747 cm^{-1}; **^{1}H NMR (400 MHz, $CDCl_3$)** δ, 7.88 (d, *J* = 8.5 Hz, 2H), 7.46 (d, *J* = 8.5 Hz, 2H), 7.43 (d, *J* = 7.3 Hz, 2H), 7.37 (t, *J* = 7.5 Hz, 2H), 7.29 (t, *J* = 7.2 Hz, 1H), 5.35 – 5.30 (m, 1H), 3.65 (d, *J* = 2.9 Hz, 1H), 3.37 (dd, *J* = 16.2, 2.7 Hz, 1H), 3.31 (dd, *J* = 16.2, 6.4 Hz, 1H), 1.33 (s, 9H); **^{13}C NMR (101 MHz, $CDCl_3$)** δ, 200.0, 157.6, 143.0, 134.0, 128.6 (2C), 128.2 (2C), 127.6, 125.8 (2C), 125.7 (2C), 70.1, 47.2, 35.2, 31.0 (3C); **HRMS (ESI, *m/z*):** calculated for $C_{19}H_{22}O_2Na$ ($[M+Na]^+$): 305.1517; found: 305.1512; $[\alpha]_D^{23}$ = +36.222 (c = 0.090, $CHCl_3$, 86% ee). The enantiomeric ratio was determined by HPLC analysis using a Daicel Chiralpak OZ-3 column (Hexane/2-propanol = 90:10, flow rate 1.0 mL/min, λ = 254 nm), t_R = 12.5 min (major), t_R = 14.3 min (minor).

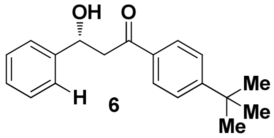

6

Procedure for deborylation- (O-allylation) of compound 3c:

In a 5 mL round bottom flask substrate **3c** (1 euqiv., 0.084 mmol) and $Cu(OAc)_2$ (2 equiv., 0.168 mmol) in 1 mL allyl alcohol as a solvent and Et_3N (4 equiv., 0.336 mmol) was taken, followed by stirring the reaction mixture up to 9 hour under room temperature. Then organic layer extracted with EtOAc (3x10 mL) and brine water also added during extraction and the residue obtained purified by column chromatography by hexane/EtOAc as eluent. The obtained product **7** was characterised as follows.

Caution: On keeping the reaction in longer time, there is a possibility of decrease of % ee of the product due to racemization.

3-(2-(Allyloxy)phenyl)-1-(4-(tert-butyl)phenyl)-3-hydroxypropan-1-one (**7**): 13 mg, 46% yield; R_f = 0.6 (30:70 = EtOAC/n-Hexane); colourless liquid ; **FT-IR** (neat): 3398, 2970, 1653, 1541, 1291, 888, 745 cm^{-1}; **^{1}H NMR (400 MHz, $CDCl_3$)** δ, 7.90 (d, *J* = 8.5 Hz, 2H), 7.56 (dd, *J* = 7.5, 1.2 Hz, 1H), 7.46 (d, *J* = 8.5 Hz, 2H), 7.23 (td, *J* = 8.1, 1.7 Hz, 1H), 7.01 (t, *J* = 7.3 Hz, 1H), 6.86 (d, *J* = 8.1 Hz, 1H), 6.02 (ddt, *J* = 17.1, 10.4, 5.1 Hz, 1H), 5.68 – 5.58 (m, 1H), 5.37 (dd, *J* = 17.3, 1.5 Hz, 1H), 5.24 (dd, *J* = 10.5, 1.3 Hz, 1H), 4.65 – 4.53 (m, 2H), 3.71 (d, *J* = 4.0 Hz, 1H), 3.54 (dd, *J* = 17.2, 2.7 Hz, 1H), 3.19 (dd, *J* = 17.2, 9.3 Hz, 1H), 1.33 (s, 9H); **^{13}C NMR (101 MHz, $CDCl_3$)** δ, 200.4, 157.3, 154.7, 134.2, 133.1, 131.6, 128.2(3C), 126.6, 125.6(2C), 121.1, 117.5, 111.5, 68.8, 65.7, 45.6, 35.2, 31.0 (3C); **HRMS (ESI, *m/z*):** calculated for $C_{22}H_{26}O_3Na$ ($[M+Na]^+$): 361.1780; found: 361.1774; $[\alpha]_D^{25}$ = +18.571 (c = 0.035, $CHCl_3$, 88% ee). The enantiomeric ratio was determined by HPLC analysis using a Daicel Chiralpak OD-3 column (Hexane/ 2-propanol = 95:05, flow rate 1.0 mL/min, λ = 254 nm), t_R = 21.1 min (major), t_R = 26.7min (minor).

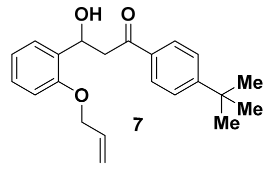

Synthetic Procedure for olefination of 3c[8]:

To a stirred solution of the cyclohexene triflate (1.2 equiv, 0.06 mmol) and $Pd(dppf)Cl_2$ (0.1 equiv, 0.005 mmol) in dimethoxyethane (1.5 mL), was added a solution of the benzoxaborol **3c** (1 equiv, 0.05 mmol) dissolved in a minimal amount of EtOH (0.2 mL), followed by a solution of Na_2CO_3 (0.03 mL, 0.06 mmol, 2 M in H_2O). The resulting mixture was heated at reflux for 1 h, then cooled to rt and filtered through a plug of celite with EtOAc. The filtrate was concentrated and purified by flash column chromatography using hexane/EtOAc as eluent and the product **8** was characterized as follows.

1-(4-(*tert*-Butyl)phenyl)-3-hydroxy-3-(2',3',4',5'-tetrahydro-[1,1'-biphenyl]-2-yl)propan-1-one (**8**): 10 mg, 53% yield; R_f = 0.42 (20:80 = EtOAc/n-Hexane); Colourless sticky liquid; **FT-IR** (neat): 3423, 2970, 2320, 1632, 1423, 1054, 735 cm^{-1}; **^{1}H NMR (400 MHz, $CDCl_3$)** δ, 7.88 (d, *J* = 8.5 Hz, 2H), 7.62 (d, *J* = 7.6 Hz, 1H), 7.47 (d, *J* = 8.5 Hz, 2H), 7.31 (td, *J* = 7.5, 1.2 Hz, 1H), 7.26 – 7.20 (m, 1H), 7.08 (dd, *J* = 7.5, 1.1 Hz, 1H), 5.56 (dd, *J* = 3.2, 1.8 Hz, 1H), 5.49 (dt, *J* = 8.3, 3.1 Hz, 1H), 3.49 (d, *J* = 2.6 Hz, 1H), 3.28 (s, 1H), 3.28 (d, *J* = 11.3 Hz, 1H), 2.34 – 2.20 (m, 1H), 2.19 – 2.03 (m, 3H), 1.71 (dd, *J* = 11.8, 5.9 Hz, 2H), 1.66 – 1.60 (m, 2H), 1.33 (s, 9H); **^{13}C NMR (101 MHz, $CDCl_3$)** δ, 200.0, 157.5, 142.8, 139.9, 138.0, 134.1, 128.7, 128.2 (2C),

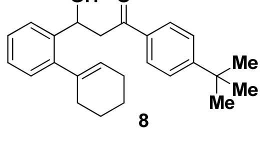

127.4, 127.2, 126.8, 125.9, 125.7 (2C), 66.8, 47.2, 35.2, 31.3, 31.1 (3C), 25.3, 23.0, 21.9; **HRMS (ESI, *m/z*):** calculated for $C_{25}H_{30}O_2Na$ ($[M+Na]^+$): 385.2138; found: 385.2137; $[\alpha]_D^{22}$ = 67.711 (c = 0.465, $CHCl_3$, 88% ee).

The enantiomeric ratio was determined by HPLC analysis using a Daicel Chiralpak OD-3 column (Hexane/2-propanol = 95:5, flow rate 1.0 mL/min, λ = 254 nm), t_R = 15.9 min (minor), t_R = 13.4 min (major).

Synthetic procedure for selective reduction of compound 3c:

To a solution of **3c** (1equiv., 0.1 mmol) in dry MeOH was taken and stirring the reaction mixture at -5°C to -10°C, then $NaBH_4$ (1.1equiv., 0.11 mmol) was added. After 15 minutes the reaction mixture passed through over silica gel and send for ^1H NMR to record the dr. The reaction mixture was purified on column chromatography using hexane/EtOAc as an eluent and the obtained product **9** was characterized as follows.

3-(2-(4-(*tert*-Butyl)phenyl)-2-hydroxyethyl)benzo[c][1,2]oxaborol-1(3H)-ol (**9**): 36.9 mg, 84% yield; R_f = 0.21 (50:50 = EtOAc/n-Hexane); Off-white solid; mp 172-174 °C; **FT-IR (neat):** 3409, 2975, 2334, 1420, 1265, 1033, 745 cm^{-1}; **^1H NMR (400 MHz, $CDCl_3$)** δ, 7.67 (dd, *J* = 7.2, 3.5 Hz, 3H), 7.46 (d, *J* = 8.2 Hz, 3H), 7.42 – 7.32 (m, 2H), 7.27 (d, *J* = 7.3 Hz, 1H), 7.16 (d, *J* = 7.5 Hz, 1H), 6.22 (dd, *J* = 11.5, 3.6 Hz, 1H), 4.73 (d, *J* = 10.4 Hz, 1H), 2.63 – 2.54 (m, 1H), 2.13 (td, *J* = 12.8, 3.6 Hz, 1H), 1.35 (s, 9H); **^{13}C NMR (101 MHz, $CDCl_3$)** δ, 156.9, 150.6, 139.5, 130.5, 130.3, 127.1, 126.9 (2C), 125.5, 125.3 (2C), 120.6, 78.4, 77.3, 77.0, 76.7, 74.4, 53.4, 45.7, 34.6, 31.4 (3C); **HRMS (ESI, *m/z*):** calculated for $C_{19}H_{23}BO_3Na$ ($[M+Na]^+$): 333.1638; found: 333.1636; $[\alpha]_D^{22}$ = -20.286 (c = 0.350, $CHCl_3$, 99% ee). The diastereomeric ratio (10:1) was calculated from ^1H NMR of the crude reaction mixture.

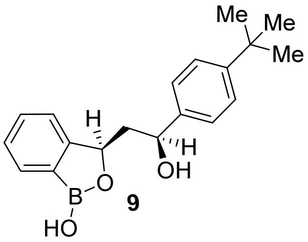

Procedure for oxidative deborylation of compound 9:

To a solution of compound **9** (1 equiv., 0.1 mmol) and in dry 1,4-dioxane was taken and degassing the reaction mixture with N_2 gas up to 1 hour, then under N_2 atmosphere $Pd(PPh_3)_4$ (0.02 equiv.) and K_2CO_3 (0.2 equiv.) was added. The reaction mixture was heated at 80°C .After completion of substrate **9** (monitored by TLC), the reaction mixture was passed

through a plug of silica gel and purified by column chromatography by using hexane/EtOAc as eluent. The obtained product **10** was characterised as follows:

1-(4-(*tert*-Butyl)phenyl)-3-(2-hydroxyphenyl)propane-1,3-diol (**10**): 20 mg, 66% yield; R_f = 0.40 (40:60 = EtOAc/n-Hexane); colourless oil; **FT-IR** (neat): 3404, 2361, 2335, 1121, 1036, 748 cm^{-1}; **^{1}H NMR (400 MHz, $CDCl_3$)** δ, 8.66 (s, 1H), 7.36 (d, *J* = 8.3 Hz, 2H), 7.28 (d, *J* = 8.3 Hz, 2H), 7.17 – 7.08 (m, 1H), 6.95 (dd, *J* = 7.5, 1.2 Hz, 1H), 6.84 (d, *J* = 8.0 Hz, 1H), 6.78 (t, *J* = 7.4 Hz, 1H), 5.25 (dd, *J* = 10.3, 1.9 Hz, 1H), 5.05 (d, *J* = 10.9 Hz, 1H), 4.96 (s, 1H), 2.50 – 2.39 (m, 2H), 1.96 (dd, *J* = 14.9, 1.5 Hz, 1H), 1.29 (s, 9H); **^{13}C NMR (101 MHz, $CDCl_3$)** δ, 155.9, 151.4, 140.4, 128.8, 126.8, 126.6, 125.7 (2C), 125.4 (2C), 119.7, 117.3, 76.9, 75.9, 45.4, 34.6, 31.3 (3C); **HRMS (ESI, *m/z*):** calculated for $C_{19}H_{24}O_3Na$ ($[M+Na]^+$): 323.1623; found: 323.1618; $[\alpha]_D^{24}$ = -1.728 (c = 0.600, $CHCl_3$, 90% ee).

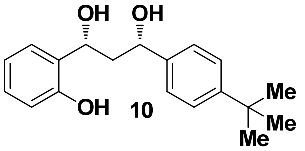

The enantiomeric ratio was determined by HPLC analysis using a Daicel Chiralpak OZ-3 column (Hexane/2-propanol = 90:10, flow rate 1.0 mL/min, λ = 254 nm), t_R = 23.7 min (minor), t_R = 29.4 min (major).

Procedure for synthesis of methyl acetal of compound 4c:

In a 2 mL round bottom flask substrate **4c** was taken (1 euqiv., 0.034 mmol) and acetaldehyde (1.2 equiv., 0.04 mmol) in 1 mL CH_2Cl_2 and then p-TSA (0.2 equiv., 0.007 mmol) was added and stirred the reaction mixture up to 30 minutes and then reaction mixture passed through over silica gel, send for ^{1}H NMR to record the dr. The reaction mixture purified on column chromatography using hexane/EtOAc as an eluent and the obtained product was characterized as follows.

Caution: On keeping the reaction in longer time there is a possibility of decrease of % ee in the product due to racemization.

1-(4-(*tert*-Butyl)phenyl)-2-(2-methyl-4H-benzo[d][1,3]dioxin-4-yl)ethan-1-one (**11**): 7 mg, 66% yield; R_f = 0.68 (10:90 = EtOAc/n-Hexane); colourless semi-solid; **FT-IR** (neat): 3029, 2930, 1651, 1584, 1280, 747 cm^{-1}; **^{1}H NMR (400 MHz, $CDCl_3$)** δ, 7.94 (d, *J* = 8.5 Hz, 2H), 7.48 (d, *J* = 8.5 Hz, 2H), 7.16 (dd, *J* = 11.4, 4.1 Hz, 1H), 7.01 (d, *J* = 7.6 Hz, 1H), 6.91 (dd, *J* = 11.7, 4.3 Hz, 1H), 6.85 (d, *J* = 8.2 Hz, 1H), 5.75 (dd, *J* = 7.9, 4.0 Hz, 1H), 5.26 (q, *J*

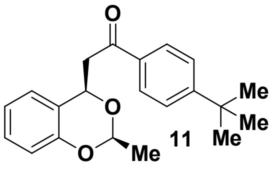

= 5.1 Hz, 1H), 3.60 (dd, J = 16.7, 8.0 Hz, 1H), 3.25 (dd, J = 16.7, 4.0 Hz, 1H), 1.47 (d, J = 5.1 Hz, 3H), 1.33 (s, 9H); **^{13}C NMR (101 MHz, $CDCl_3$)** δ, 197.2, 157.2, 153.3, 134.5, 128.3 (2C), 128.3, 125.6 (2C), 124.7, 124.4, 121.2, 116.9, 96.8, 72.3, 44.8, 35.2, 31.1 (3C), 20.7; **HRMS (ESI, *m/z*):** calculated for $C_{21}H_{25}O_3$ ($[M+H]^+$): 325.1803; found: 325.1798; $[\alpha]_D^{25}$ = +59.178 (c = 0.365, $CHCl_3$, 90% ee). The diastereomeric ratio (43:1) was calculated from ^{1}H NMR of the crude reaction mixture.The enantiomeric ratio was determined by HPLC analysis using a Daicel Chiralpak OD-3 column (Hexane/2-propanol = 90:10, flow rate 1.0 mL/min, λ = 254 nm), t_R = 5.3 min (major), t_R = 7.9 min (minor).

Procedure for synthesis of phenyl acetal of compound 4c:

In a 2 mL round bottom flask substrate **4c** was taken (1 euqiv., 0.05 mmol) and benzaldehyde dimethyl acetal (1.2 equiv., 0.06 mmol) in 1 mL CH_2Cl_2 and then p-TSA (0.2 equiv., 0.01 mmol) was added and stirred the reaction mixture up to 2 hours. After completion (monitored by TLC) reaction mixture passed through over silica gel, send for ^{1}H NMR to record the dr. Then the reaction mixture was purified on column chromatography using hexane/EtOAc as an eluent and the obtained product was characterized as follows.

Caution: On keeping the reaction in longer time there is a possibility of decrease of % ee in the product due to racemization.

1-(4-(*tert*-Butyl)phenyl)-2-(2-phenyl-4H-benzo[d][1,3]dioxin-4-yl)ethan-1-one (**12**): 14 mg, 72% yield; R_f= 0.44 (10:90 = EtOAc/n-Hexane); light yellow solid; mp 102-104 °C; **FT-IR** (neat)**:** 3033, 2941, 1651, 1584, 1282, 746 cm^{-1}; **^{1}H NMR (400 MHz, $CDCl_3$)** δ, 7.95 (d, J = 8.5 Hz, 2H), 7.52 (dd, J = 6.6, 2.8 Hz, 2H), 7.47 (d, J = 8.5 Hz, 2H), 7.36 (dd, J = 4.9, 1.6 Hz, 3H), 7.21 (t, J = 7.7 Hz, 1H), 7.09 (d, J = 7.7 Hz, 1H), 6.97 (t, J = 7.2 Hz, 2H), 6.07 (s, 1H), 5.97 (dd, J = 7.5, 4.3 Hz, 1H), 3.73 (dd, J = 16.6, 7.7 Hz, 1H), 3.35 (dd, J = 16.7, 4.3 Hz, 1H), 1.33 (s, 9H); **^{13}C NMR (101 MHz, $CDCl_3$)** δ, 197.1, 157.2, 153.4, 137.0, 134.5, 129.3, 128.4, 128.4 (2C), 128.3 (2C), 126.4 (2C), 125.6 (2C), 124.8, 124.6, 121.6, 117.3, 98.8, 73.3, 44.9, 35.2, 31.0 (3C); **HRMS (ESI, *m/z*):** calculated for $C_{26}H_{26}O_3Na$ ($[M+Na]^+$): 409.1780; found: 409.1774; $[\alpha]_D^{23}$ = +35.322 (c = 0.155, $CHCl_3$, 90% ee). The diastereomeric ratio (>10:1) was calculated from ^{1}H NMR of the crude reaction mixture.The enantiomeric ratio was determined by HPLC analysis using a Daicel Chiralpak OD-3 column (Hexane/2-propanol = 97:3, flow rate 1.0 mL/min, λ = 254 nm), t_R = 8.8 min (major), t_R = 19.6 min (minor).

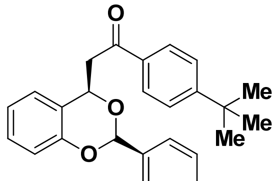

12

NOE Experiment of **9**

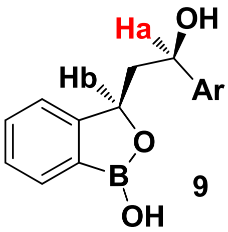

9

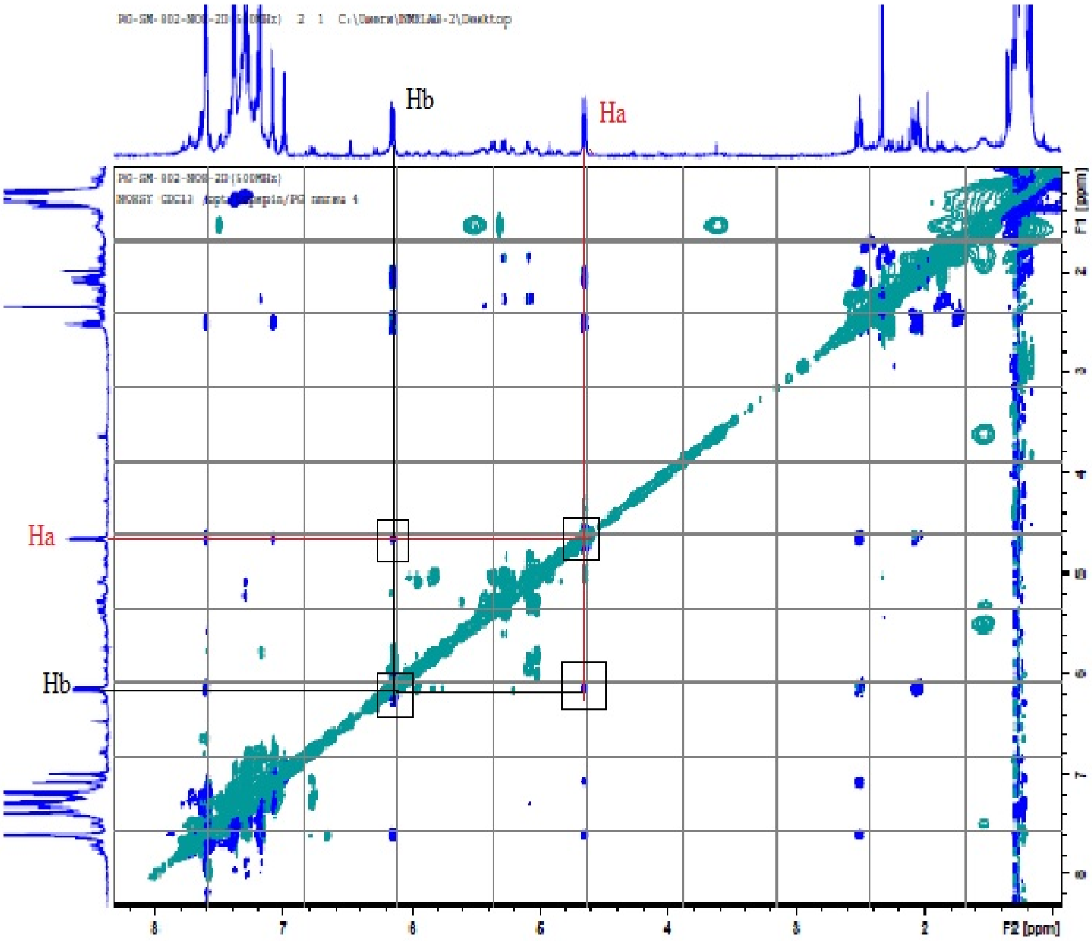

NOE experiment of **12**

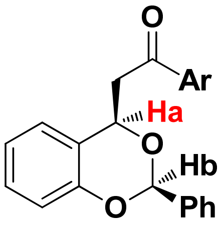

12

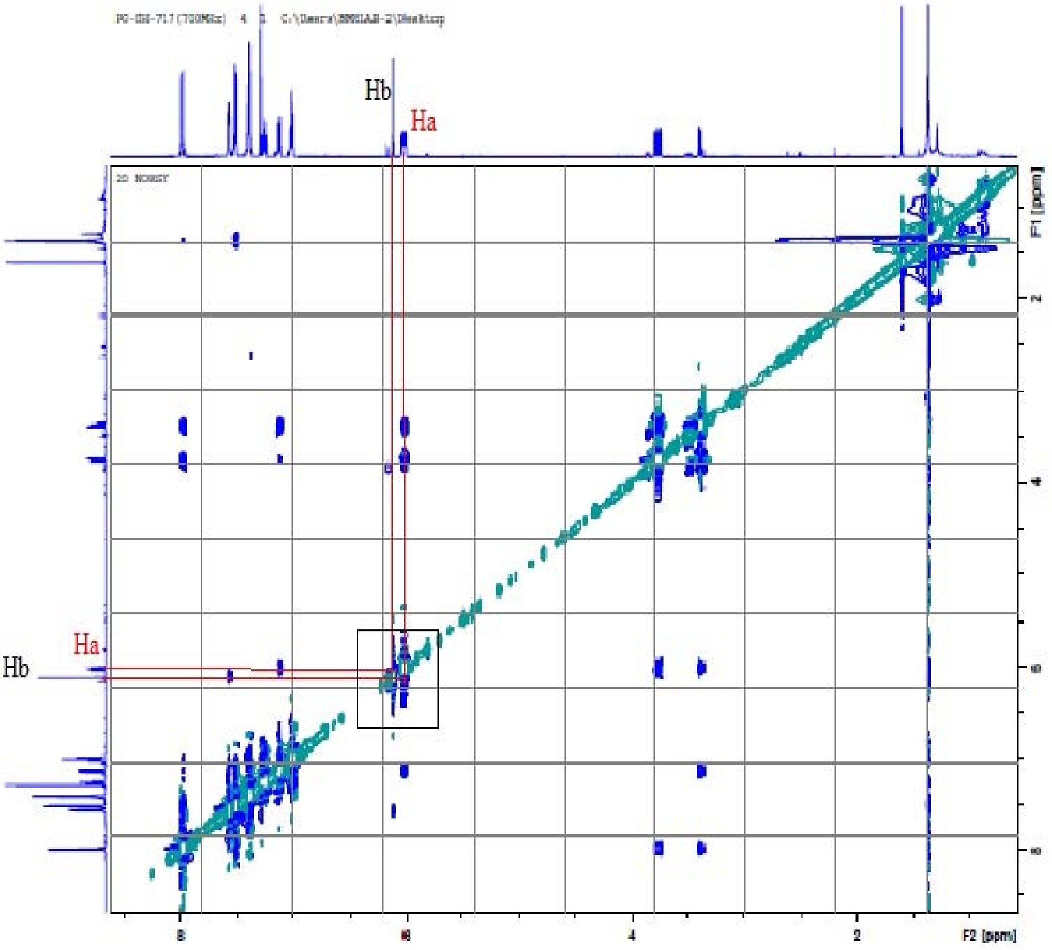

References:

1.a) Lee, J. W.; Ryu, T. H.; Oh, J. S.; Bae, H. Y.; Jang, H. B.; Song, C. E. *Chem. Commun.*, **2009**, 7224. b) Molleti, N.; Singh, V. K.; *Org. Biomol. Chem.*, **2015**, *13*, 5243.

2) Oswald, C. L.; Peterson, J. A.; H. W. *Org. Lett.*, **2009**, *11*, 4504.

3) Li, D. R.; Murugan, A.; and J. R. Falck**,** J. R. *J. Am. Chem. Soc*. **2008**, *130*, 46.

4) Guo, L-N.; Duan, X-H.; Hu, J.; Bi, H-P.; Liu, X-Y.; Liang, Y-M. *Eur. J. Org. Chem.* **2008**, 1418.

5) Liu, C.; Li, X.; Wu, Y. *RSC Adv*. **2015**, *5*, 15354.

6) Ramachary, D. B.; Sakthidevi, R. *Chem. Eur. J*. **2009**, *15*, 4516.

7) Quach, T. D.; Batey, R. A. *Org. Lett.*, **2003**, *5*, 1381.

8) Cho, I.; Meimetis, L.; Belding, L.; Katz, M. J.; Dudding, T.; Britton, R. *Beilstein J. Org. Chem*. **2011**, *7*, 1315.

Supporting Information - II: *Spectra and Chromatograms*

Organocatalytic, Enantioselective Synthesis of Benzoxaboroles *via* Wittig / oxa-Michael Reaction Cascade of α-Formyl Boronic Acids.

Gurupada Hazra, Sanjay Maity, Sudipto Bhoumik, and Prasanta Ghorai*

Department of Chemistry, Indian Institute of Science Education and Research (IISER) Bhopal, Bhopal By-pass Road, Bhouri, Bhopal-462066, India.

E-mail: pghorai@iiserb.ac.in.

Contents: **Pages**

I.	NMR spectra of chiral benzoxaboroles	2-24
II.	NMR and HPLC spectra of β-hydroxy ketones:	25-70
III.	NMR and HPLC spectra of functionalised products:	71-85
IV.	XRD data for **3f**:	86

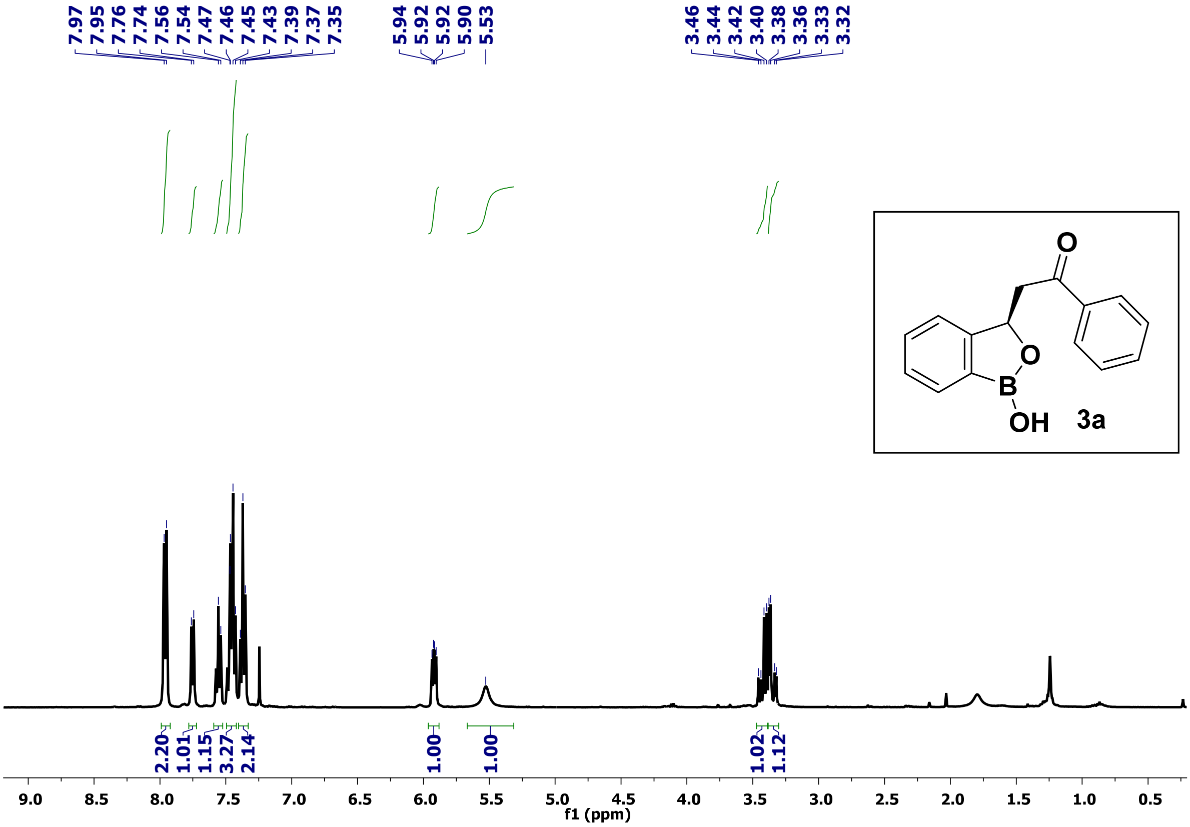
7.97 7.95 7.76 7.74 7.56 7.54 7.47 7.46 7.45 7.43 7.39 7.37 7.35
5.94 5.92 5.92 5.90 5.53
3.46 3.44 3.42 3.40 3.38 3.36 3.33 3.32
O
O
B
OH
3a
2.20 1.01 1.15 3.27 2.14 1.00 1.00 1.02 1.12
9.0 8.5 8.0 7.5 7.0 6.5 6.0 5.5 5.0 4.5 4.0 3.5 3.0 2.5 2.0 1.5 1.0 0.5
f1 (ppm)

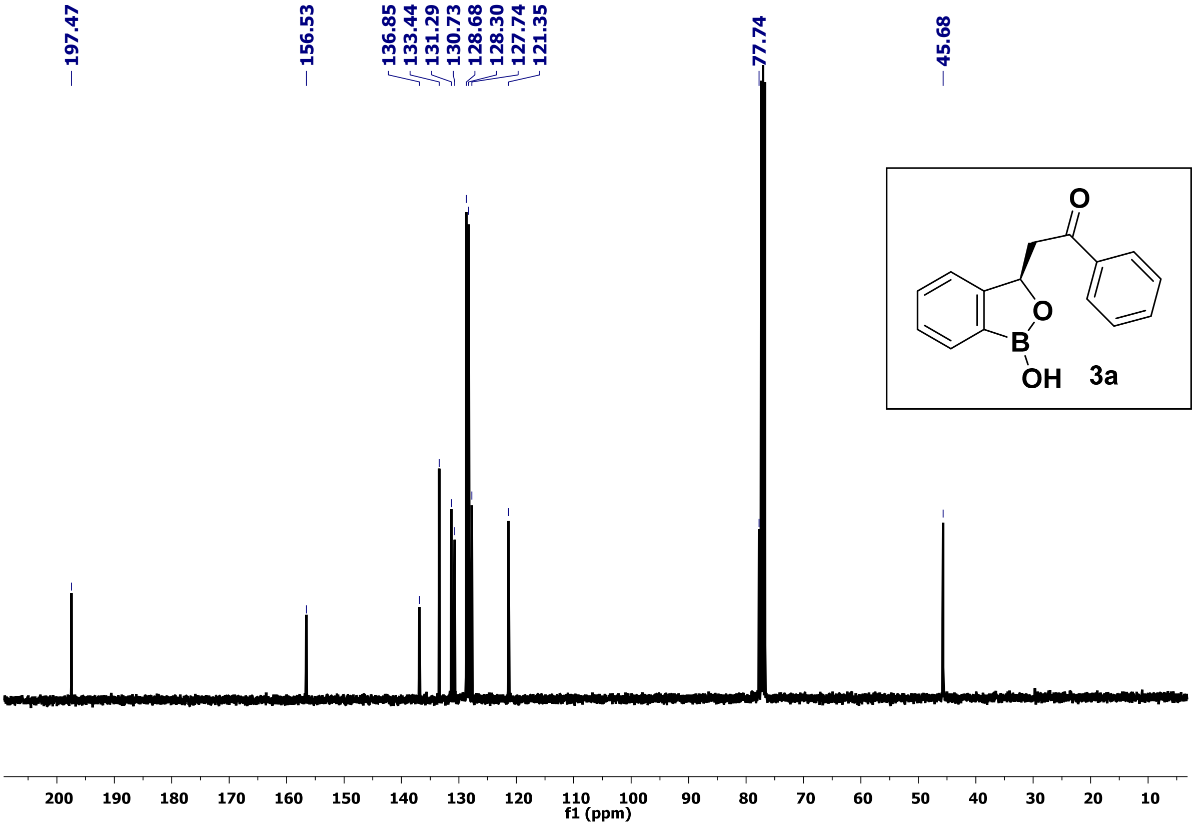
197.47
156.53
136.85 133.44 131.29 130.73 128.68 128.30 127.74 121.35
77.74
45.68
O
O
B
OH
3a
200 190 180 170 160 150 140 130 120 110 100 90 80 70 60 50 40 30 20 10
f1 (ppm)

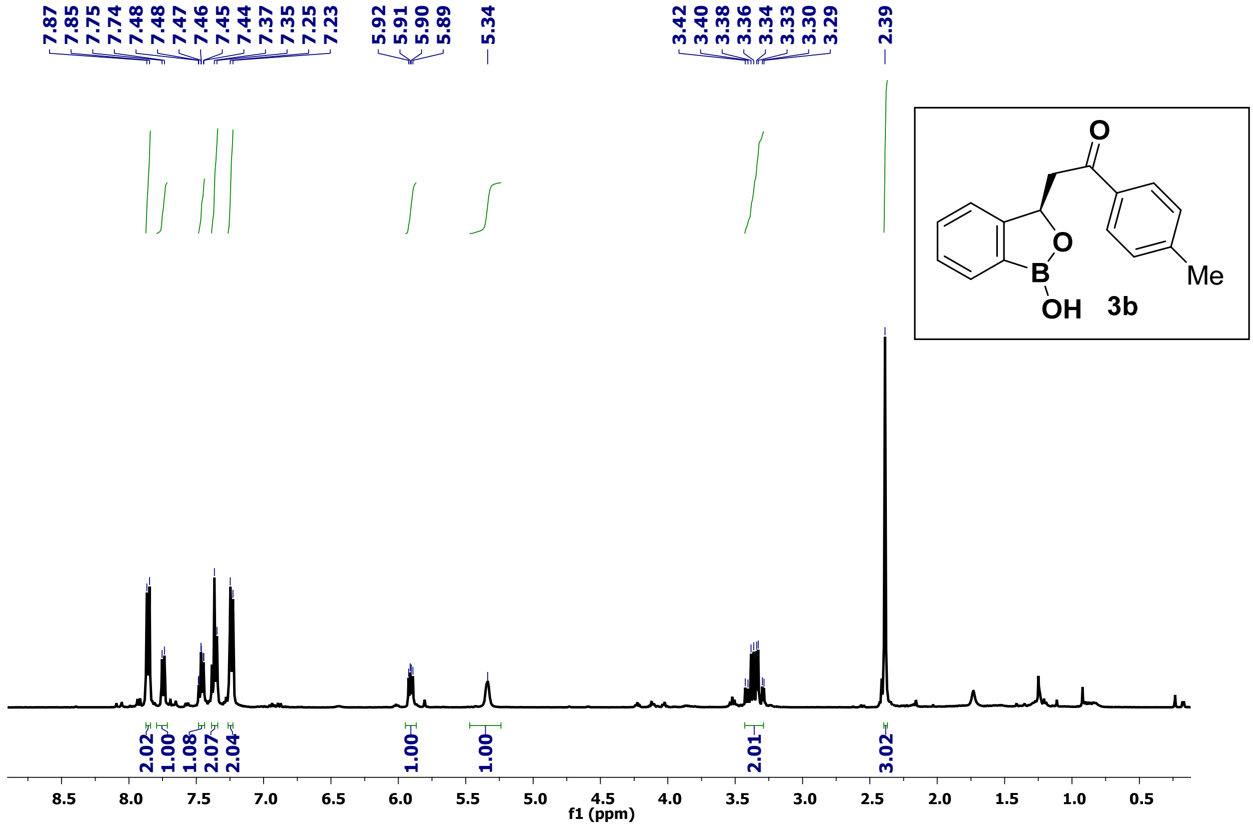
7.87 7.85 7.75 7.74 7.48 7.48 7.47 7.46 7.45 7.44 7.37 7.35 7.25 7.23
5.92 5.91 5.90 5.89
5.34
3.42 3.40 3.38 3.36 3.34 3.33 3.30 3.29
2.39
2.02 1.00 1.08 2.07 2.04 1.00 1.00 2.01 3.02
f1 (ppm)
O
O
B
OH
Me
3b

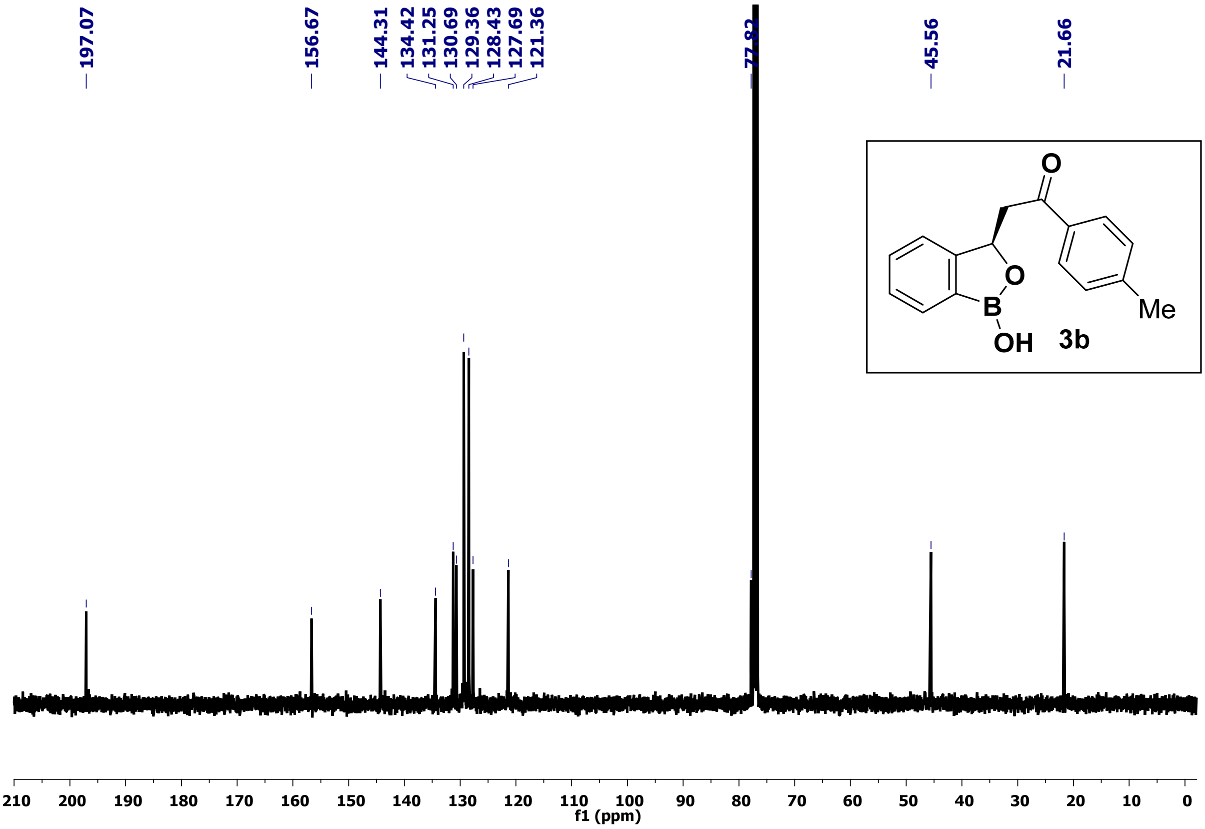
197.07
156.67
144.31
134.42 131.25 130.69 129.36 128.43 127.69 121.36
45.56
21.66
f1 (ppm)
O
O
B
OH
Me
3b

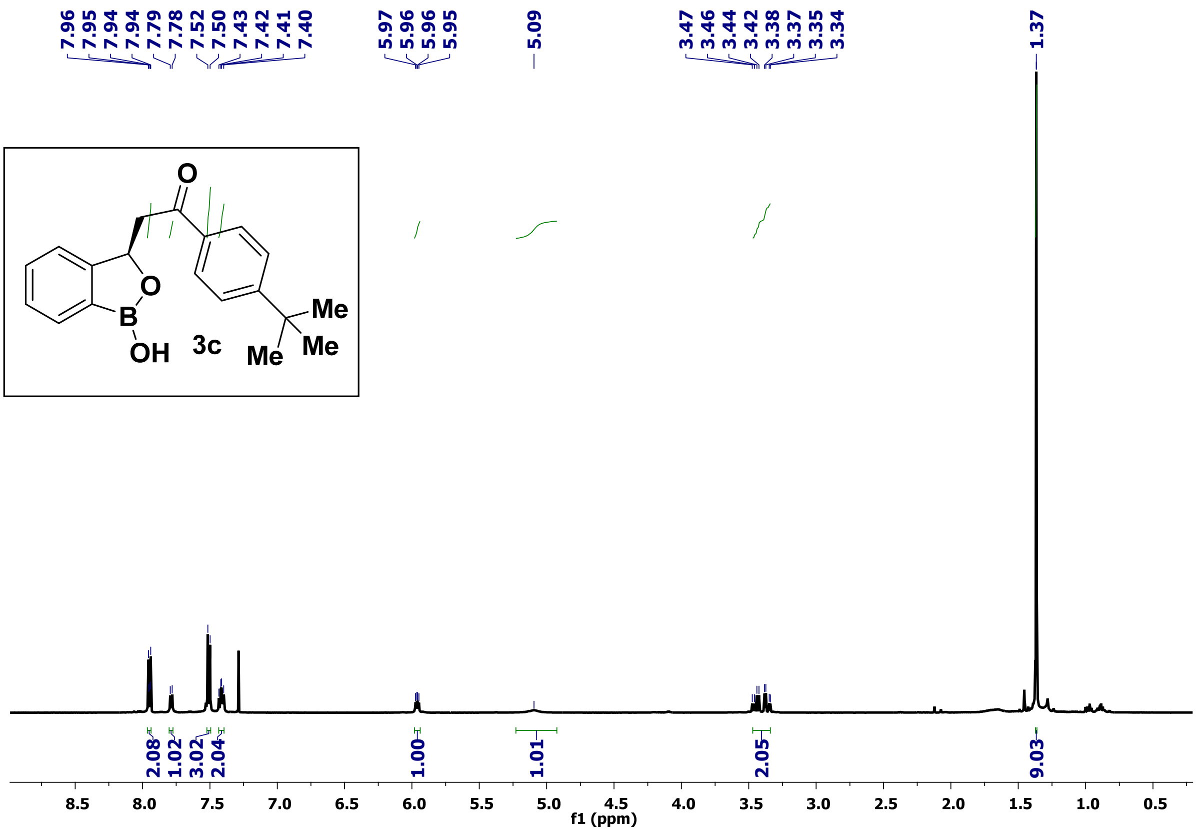
7.96 7.95 7.94 7.94 7.79 7.78 7.52 7.50 7.43 7.42 7.41 7.40
5.97 5.96 5.96 5.95
5.09
3.47 3.46 3.44 3.42 3.38 3.37 3.35 3.34
1.37
O
O
B
OH
3c
Me
Me
Me
2.08 1.02 3.02 2.04 1.00 1.01 2.05 9.03
f1 (ppm)

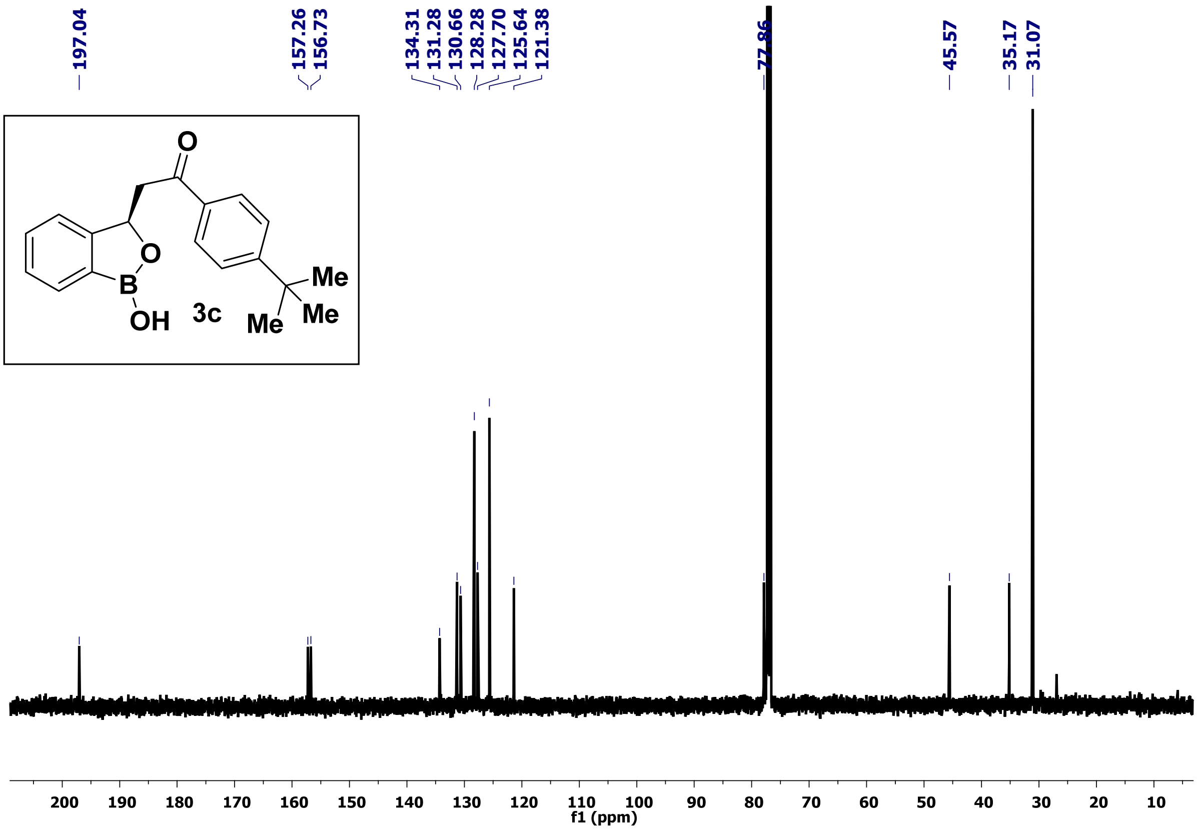
197.04
157.26 156.73
134.31 131.28 130.66 128.28 127.70 125.64 121.38
77.86
45.57
35.17 31.07
O
O
B
OH
3c
Me
Me
Me
f1 (ppm)

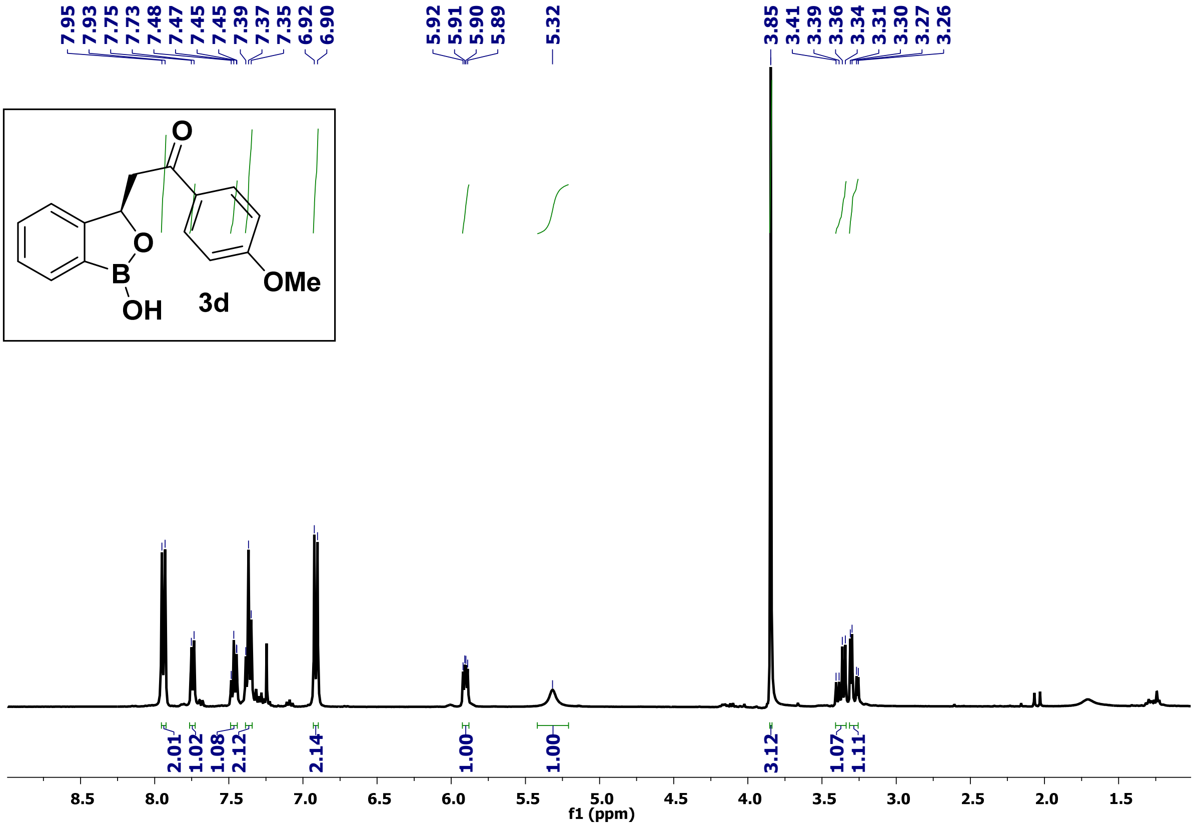
7.95 7.93 7.75 7.73 7.48 7.47 7.45 7.45 7.39 7.37 7.35 6.92 6.90
5.92 5.91 5.90 5.89
5.32
3.85 3.41 3.39 3.36 3.34 3.31 3.30 3.27 3.26
O
O
B
OH
3d
OMe
2.01 1.02 1.08 2.12 2.14 1.00 1.00 3.12 1.07 1.11
8.5 8.0 7.5 7.0 6.5 6.0 5.5 5.0 4.5 4.0 3.5 3.0 2.5 2.0 1.5
f1 (ppm)

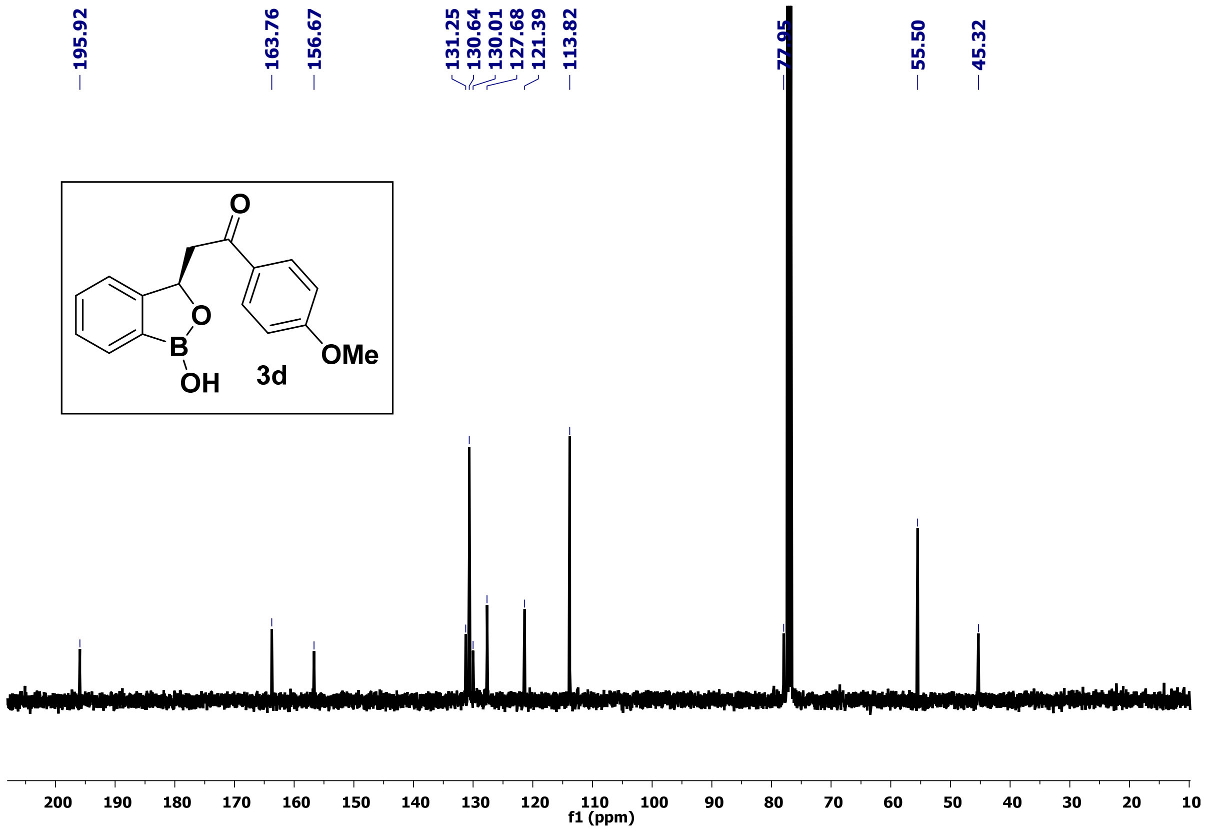
195.92
163.76
156.67
131.25 130.64 130.01 127.68 121.39
113.82
77.95
55.50
45.32
O
O
B
OH
3d
OMe
200 190 180 170 160 150 140 130 120 110 100 90 80 70 60 50 40 30 20 10
f1 (ppm)

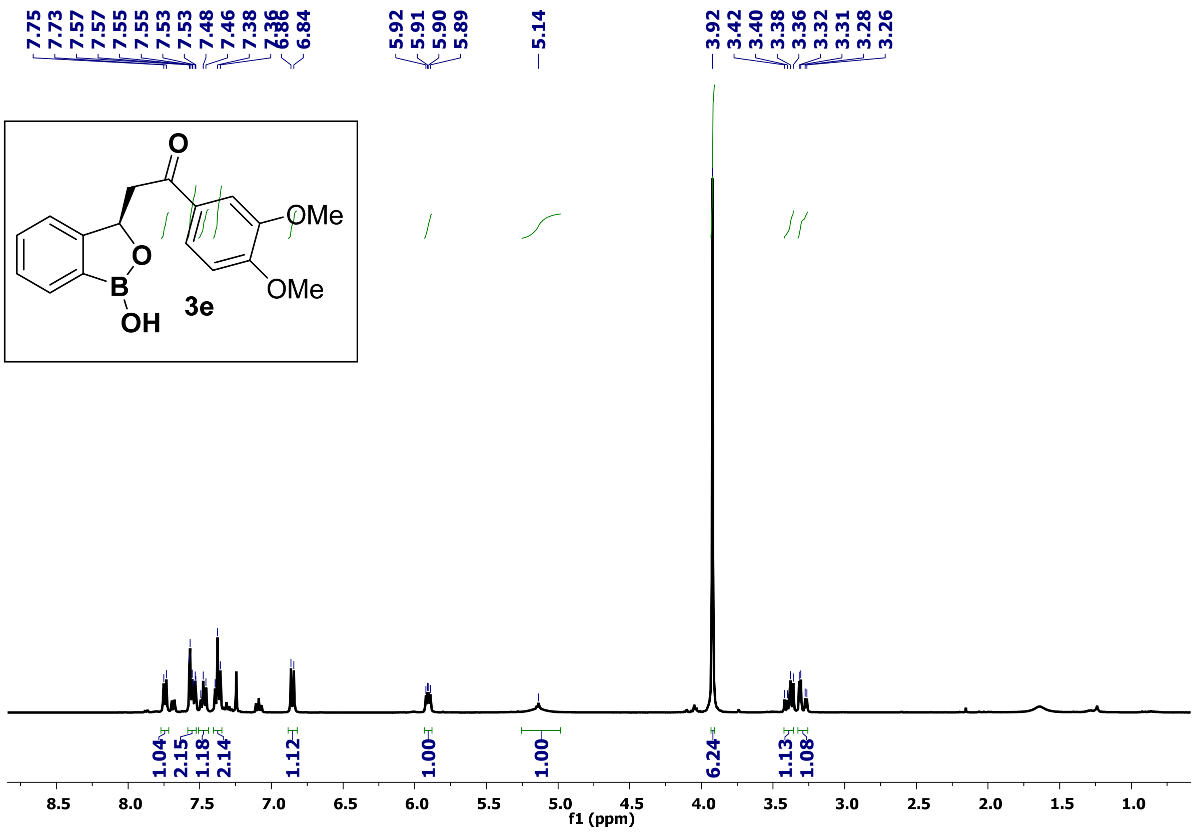
7.75 7.73 7.57 7.57 7.55 7.55 7.53 7.53 7.48 7.46 7.38 7.36 6.86 6.84
5.92 5.91 5.90 5.89
5.14
3.92 3.42 3.40 3.38 3.36 3.32 3.31 3.28 3.26
O
OMe
O
B
OMe
OH
3e
1.04 2.15 1.18 2.14 1.12 1.00 1.00 6.24 1.13 1.08
f1 (ppm)

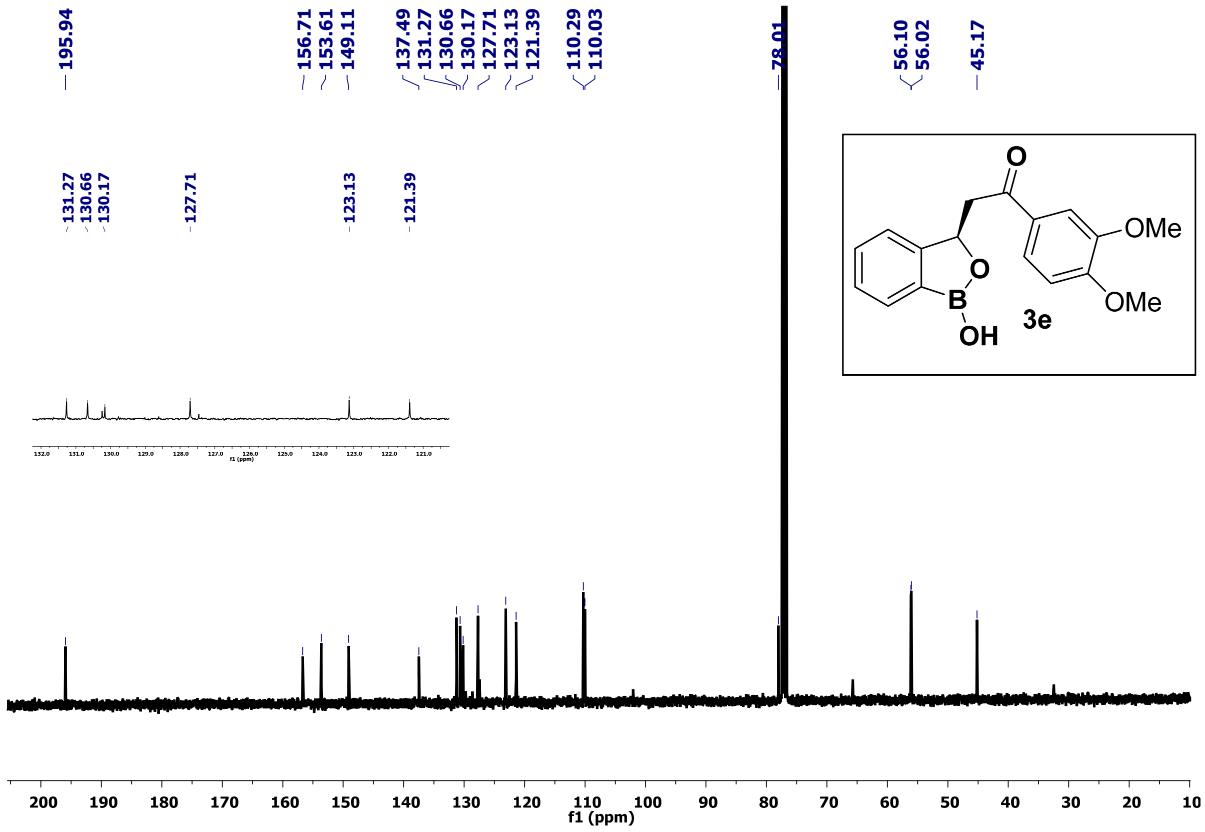
195.94
156.71 153.61 149.11
137.49 131.27 130.66 130.17 127.71 123.13 121.39
110.29 110.03
78.01
56.10 56.02
45.17
131.27 130.66 130.17
127.71
123.13
121.39
O
OMe
O
B
OMe
OH
3e
f1 (ppm)

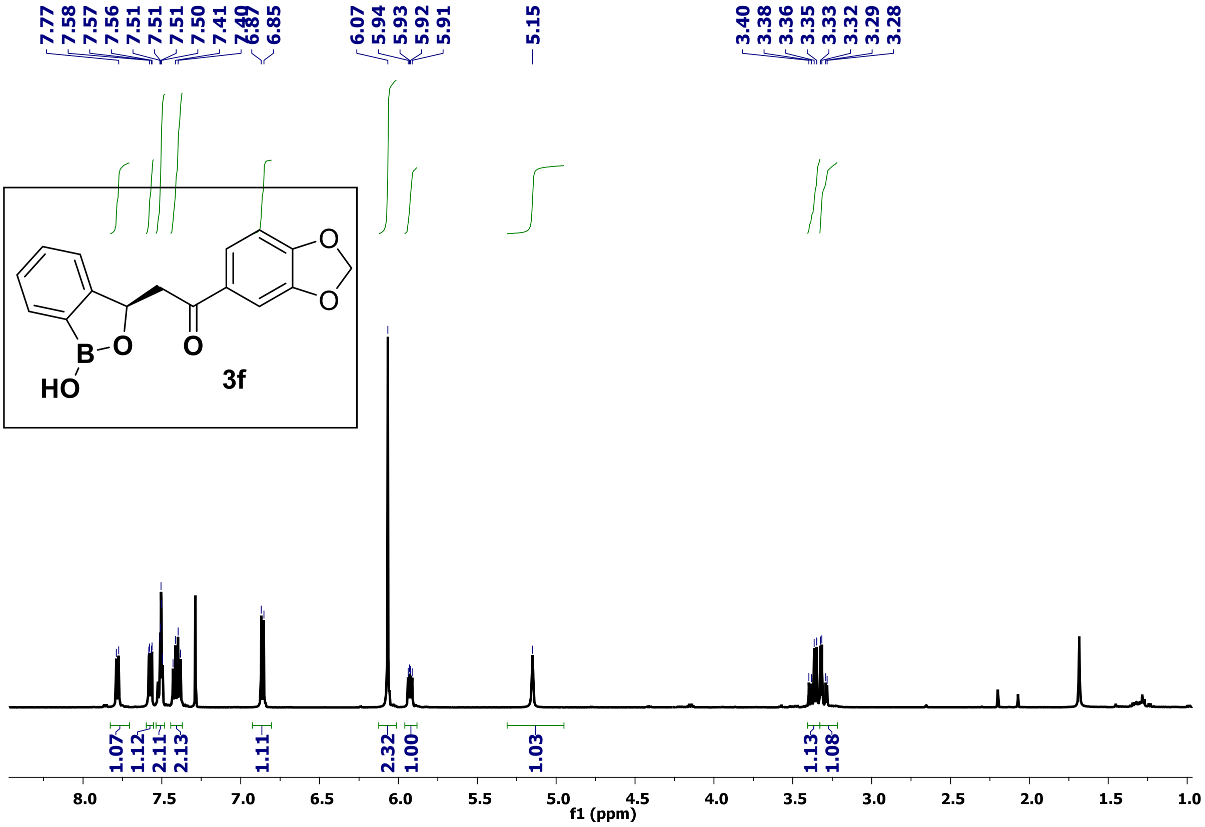
7.77 7.58 7.57 7.56 7.51 7.51 7.51 7.50 7.41 7.40 6.87 6.85
6.07 5.94 5.93 5.92 5.91
5.15
3.40 3.38 3.36 3.35 3.33 3.32 3.29 3.28
B
O
HO
O
O
O
3f
1.07 1.12 2.11 2.13 1.11 2.32 1.00 1.03 1.13 1.08
8.0 7.5 7.0 6.5 6.0 5.5 5.0 4.5 4.0 3.5 3.0 2.5 2.0 1.5 1.0
f1 (ppm)

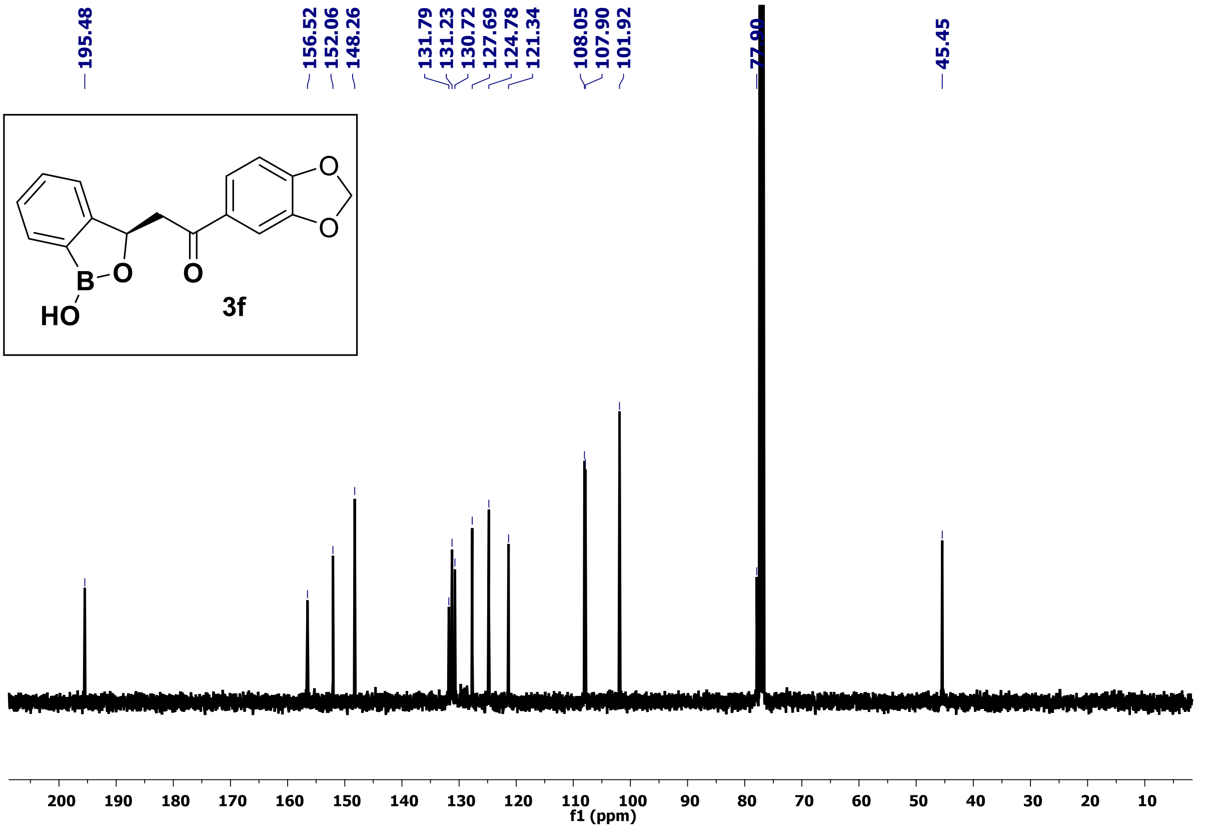
195.48
156.52 152.06 148.26
131.79 131.23 130.72 127.69 124.78 121.34
108.05 107.90 101.92
77.00
45.45
B
O
HO
O
O
O
3f
200 190 180 170 160 150 140 130 120 110 100 90 80 70 60 50 40 30 20 10
f1 (ppm)

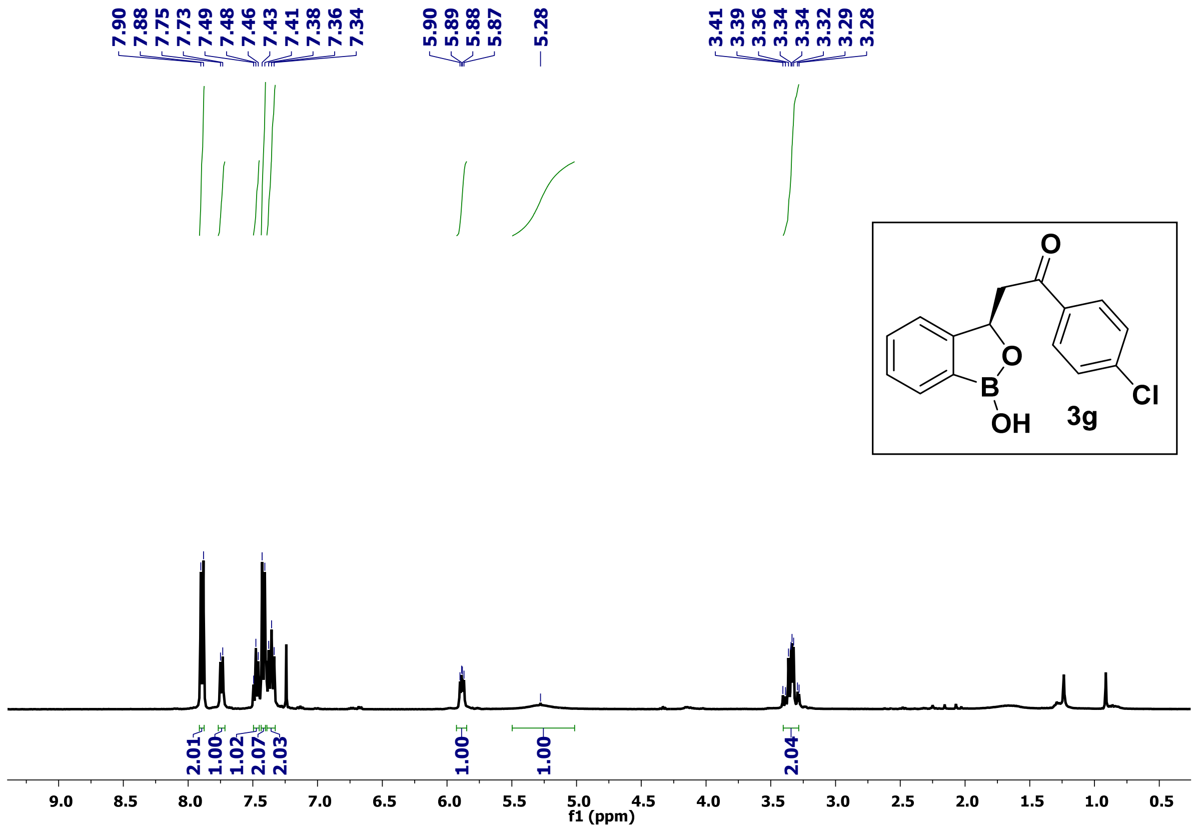
7.90 7.88 7.75 7.73 7.49 7.48 7.46 7.43 7.41 7.38 7.36 7.34
5.90 5.89 5.88 5.87
5.28
3.41 3.39 3.36 3.34 3.34 3.32 3.29 3.28
O
O
B
OH
3g
Cl
2.01 1.00 1.02 2.07 2.03
1.00
1.00
2.04
9.0 8.5 8.0 7.5 7.0 6.5 6.0 5.5 5.0 4.5 4.0 3.5 3.0 2.5 2.0 1.5 1.0 0.5
f1 (ppm)

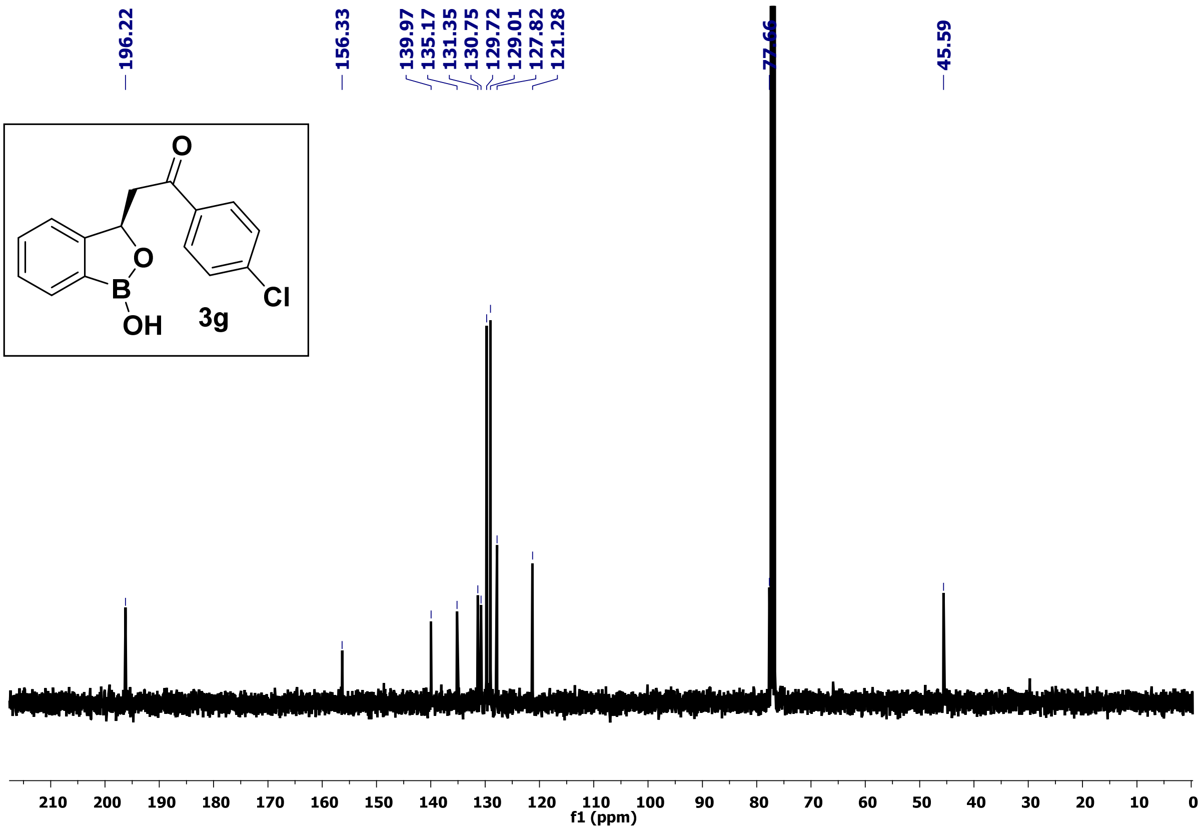
196.22
156.33
139.97 135.17 131.35 130.75 129.72 129.01 127.82 121.28
77.66
45.59
O
O
B
OH
3g
Cl
210 200 190 180 170 160 150 140 130 120 110 100 90 80 70 60 50 40 30 20 10 0
f1 (ppm)

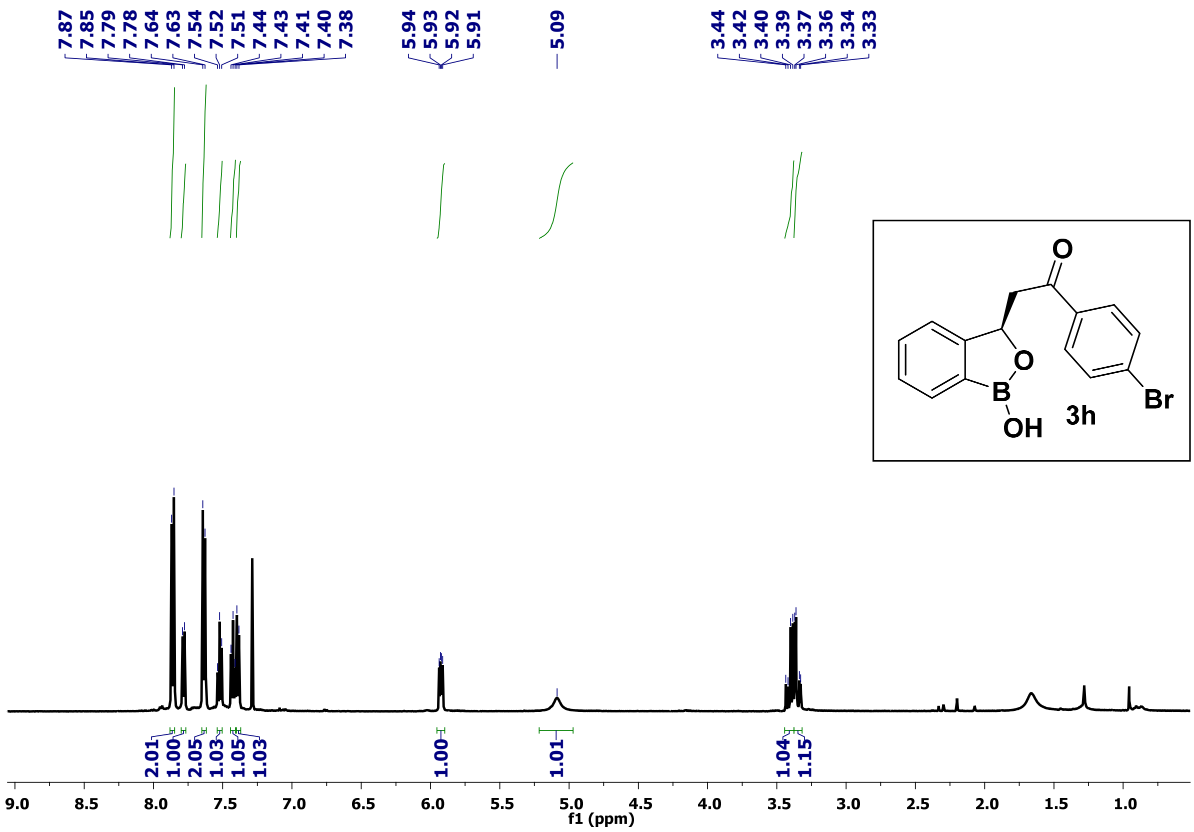
7.87 7.85 7.79 7.78 7.64 7.63 7.54 7.52 7.51 7.44 7.43 7.41 7.40 7.38
5.94 5.93 5.92 5.91
5.09
3.44 3.42 3.40 3.39 3.37 3.36 3.34 3.33
O
O
B
OH
3h
Br
2.01 1.00 2.05 1.03 1.05 1.03
1.00
1.01
1.04 1.15
9.0 8.5 8.0 7.5 7.0 6.5 6.0 5.5 5.0 4.5 4.0 3.5 3.0 2.5 2.0 1.5 1.0
f1 (ppm)

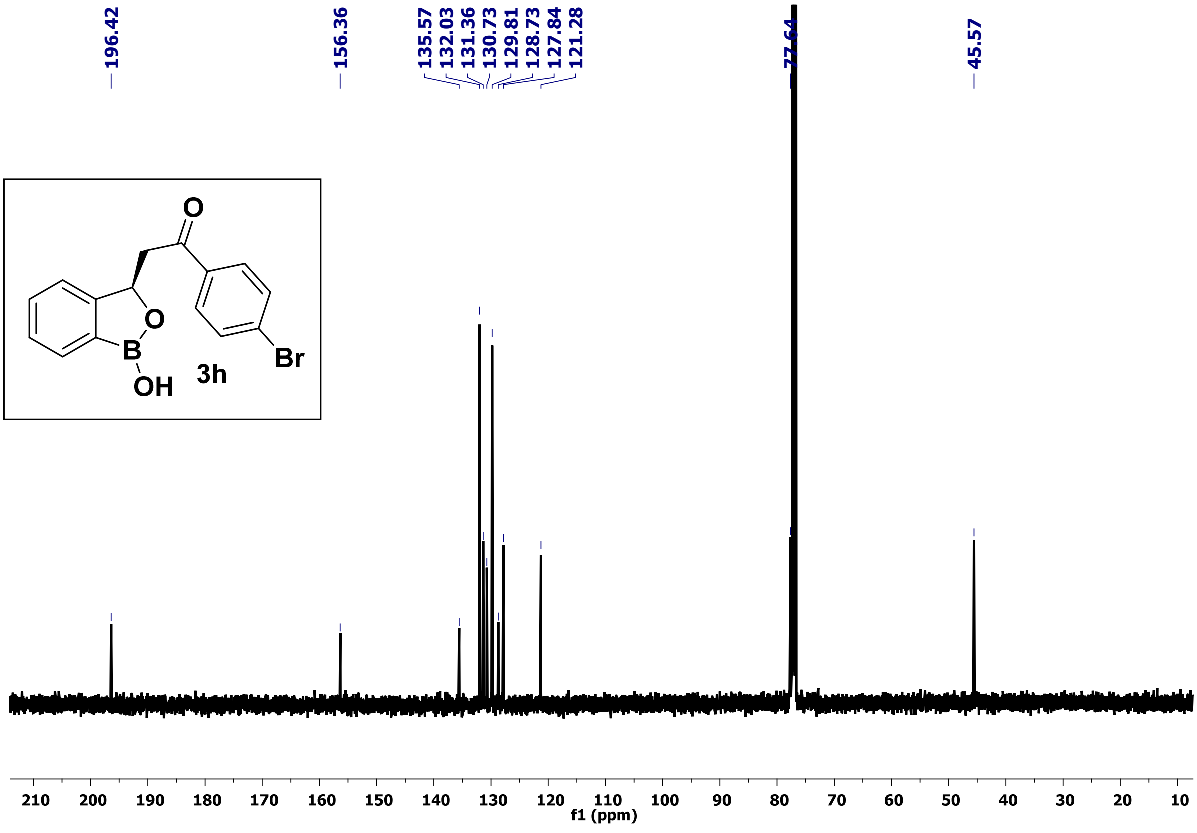
196.42
156.36
135.57 132.03 131.36 130.73 129.81 128.73 127.84 121.28
77.64
45.57
O
O
B
OH
3h
Br
210 200 190 180 170 160 150 140 130 120 110 100 90 80 70 60 50 40 30 20 10
f1 (ppm)

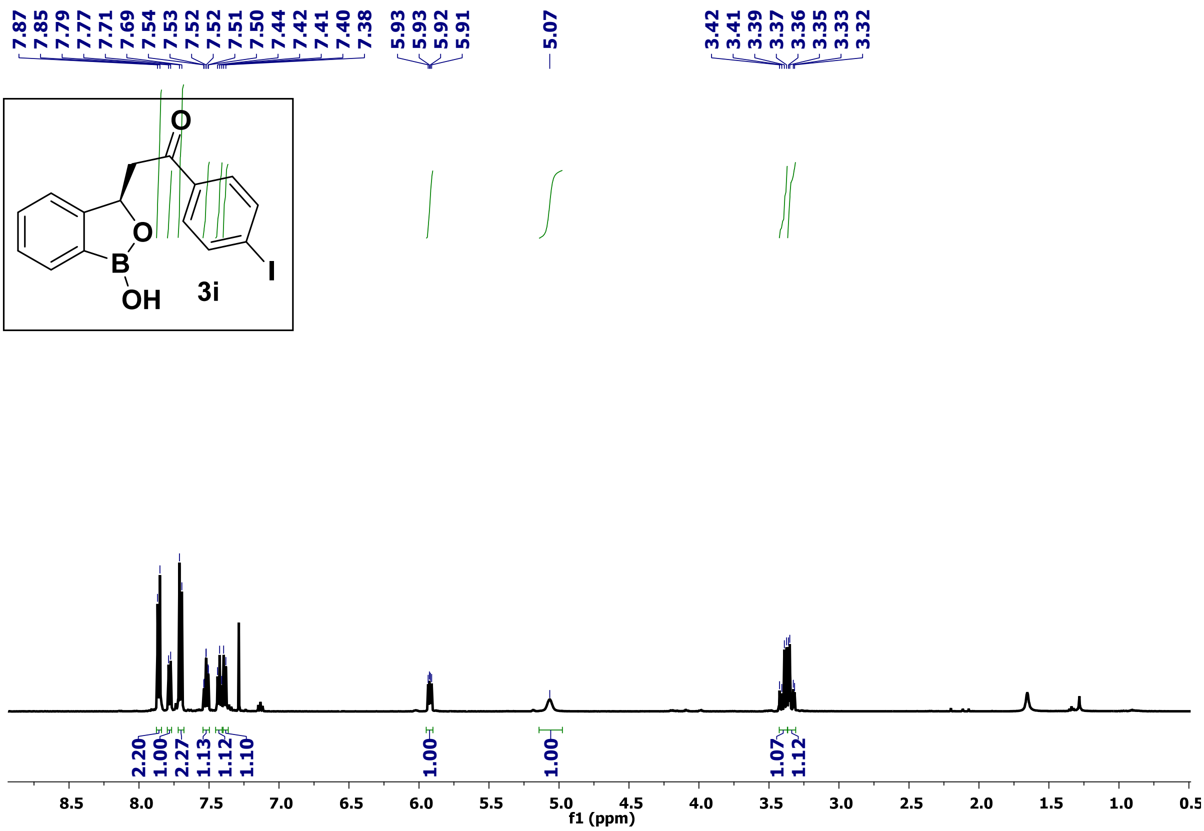
7.87
7.85
7.79
7.77
7.71
7.69
7.54
7.53
7.52
7.52
7.51
7.50
7.44
7.42
7.41
7.40
7.38
5.93
5.93
5.92
5.91
5.07
3.42
3.41
3.39
3.37
3.36
3.35
3.33
3.32
O
O
B
OH
3i
I
2.20
1.00
2.27
1.13
1.12
1.10
1.00
1.00
1.07
1.12
8.5
8.0
7.5
7.0
6.5
6.0
5.5
5.0
4.5
4.0
3.5
3.0
2.5
2.0
1.5
1.0
0.5
f1 (ppm)

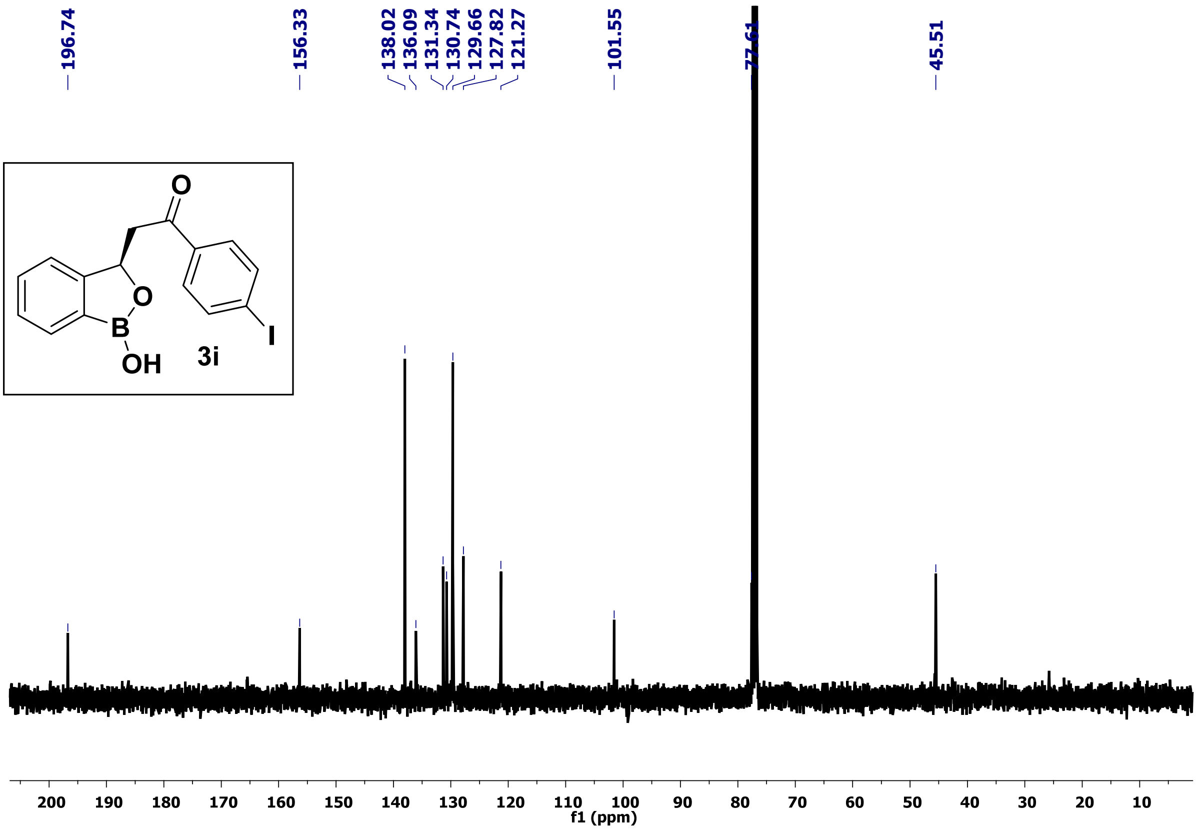
196.74
156.33
138.02
136.09
131.34
130.74
129.66
127.82
121.27
101.55
77.61
45.51
O
O
B
OH
3i
I
200
190
180
170
160
150
140
130
120
110
100
90
80
70
60
50
40
30
20
10
f1 (ppm)

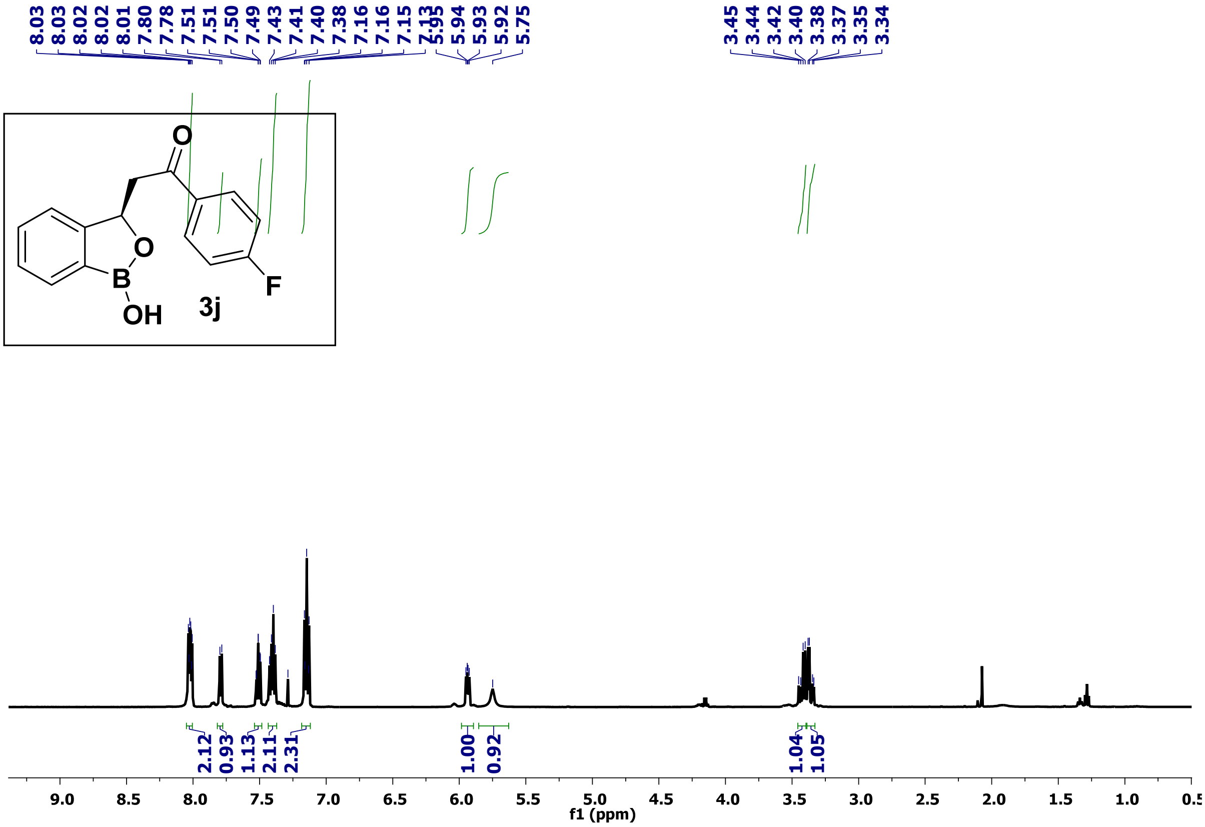
8.03
8.03
8.02
8.02
8.01
7.80
7.78
7.51
7.51
7.50
7.49
7.43
7.41
7.40
7.38
7.16
7.16
7.15
7.13
5.95
5.94
5.93
5.92
5.75
3.45
3.44
3.42
3.40
3.38
3.37
3.35
3.34
O
O
B
OH
F
3j
2.12
0.93
1.13
2.11
2.31
1.00
0.92
1.04
1.05
9.0 8.5 8.0 7.5 7.0 6.5 6.0 5.5 5.0 4.5 4.0 3.5 3.0 2.5 2.0 1.5 1.0
f1 (ppm)

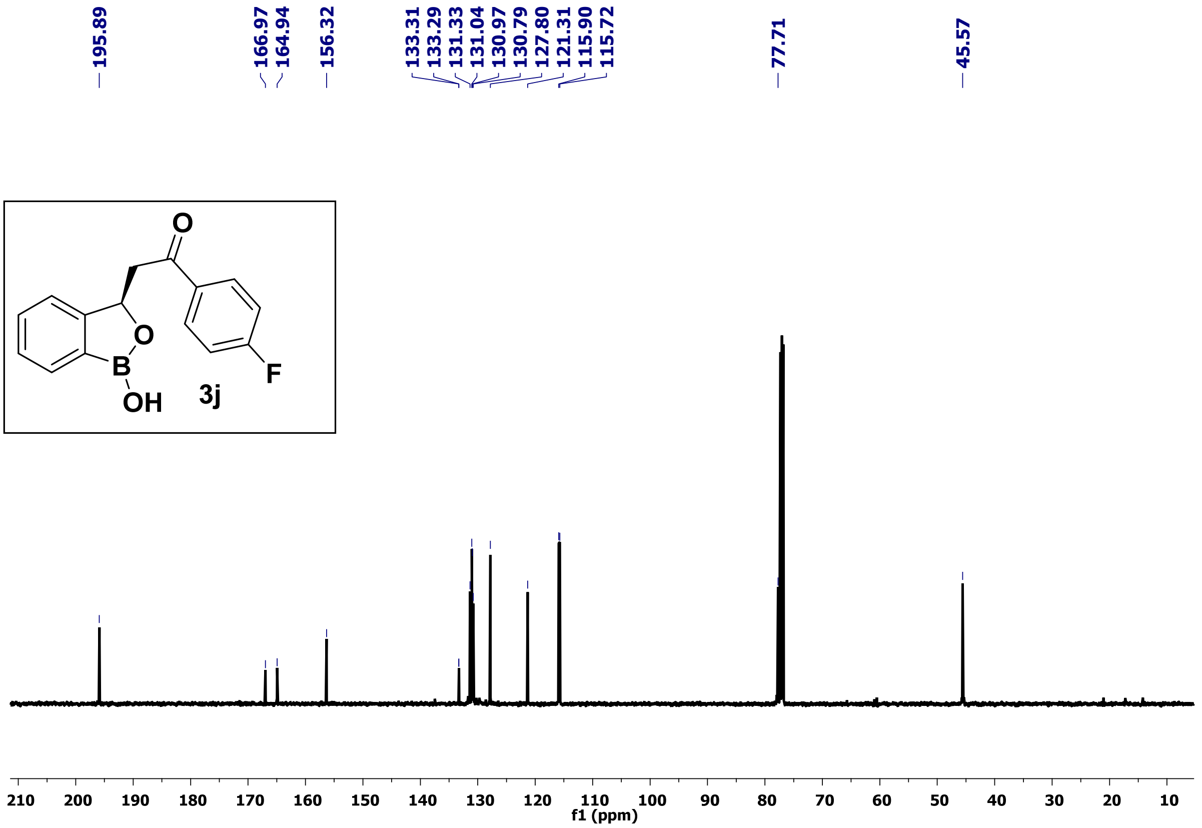
195.89
166.97
164.94
156.32
133.31
133.29
131.33
131.04
130.97
130.79
127.80
121.31
115.90
115.72
77.71
45.57
O
O
B
OH
F
3j
210 200 190 180 170 160 150 140 130 120 110 100 90 80 70 60 50 40 30 20 10
f1 (ppm)

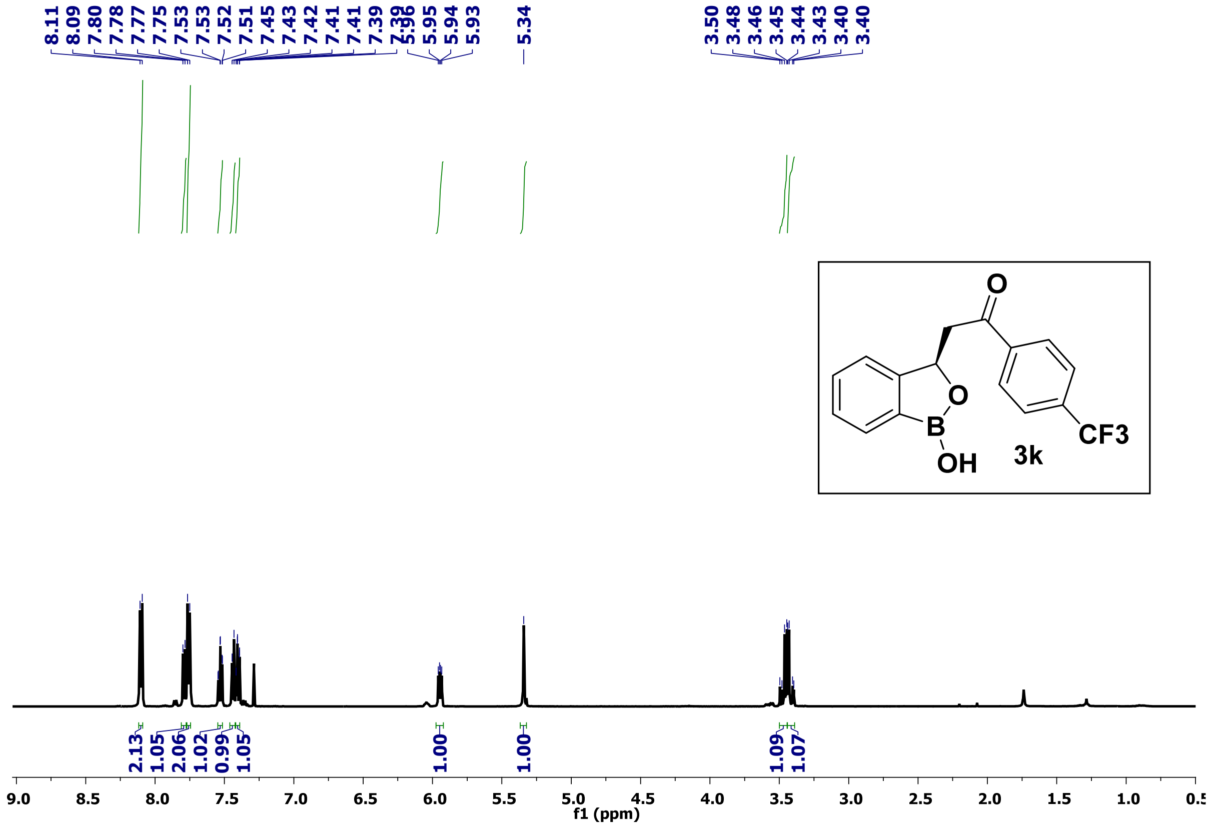
8.11 8.09 7.80 7.78 7.77 7.75 7.53 7.53 7.52 7.51 7.45 7.43 7.42 7.41 7.41 7.39 7.39 5.96 5.95 5.94 5.93
5.34
3.50 3.48 3.46 3.45 3.44 3.43 3.40 3.40
O
O
B
OH
3k
CF3
2.13 1.05 2.06 1.02 0.99 1.05
1.00
1.00
1.09 1.07
f1 (ppm)

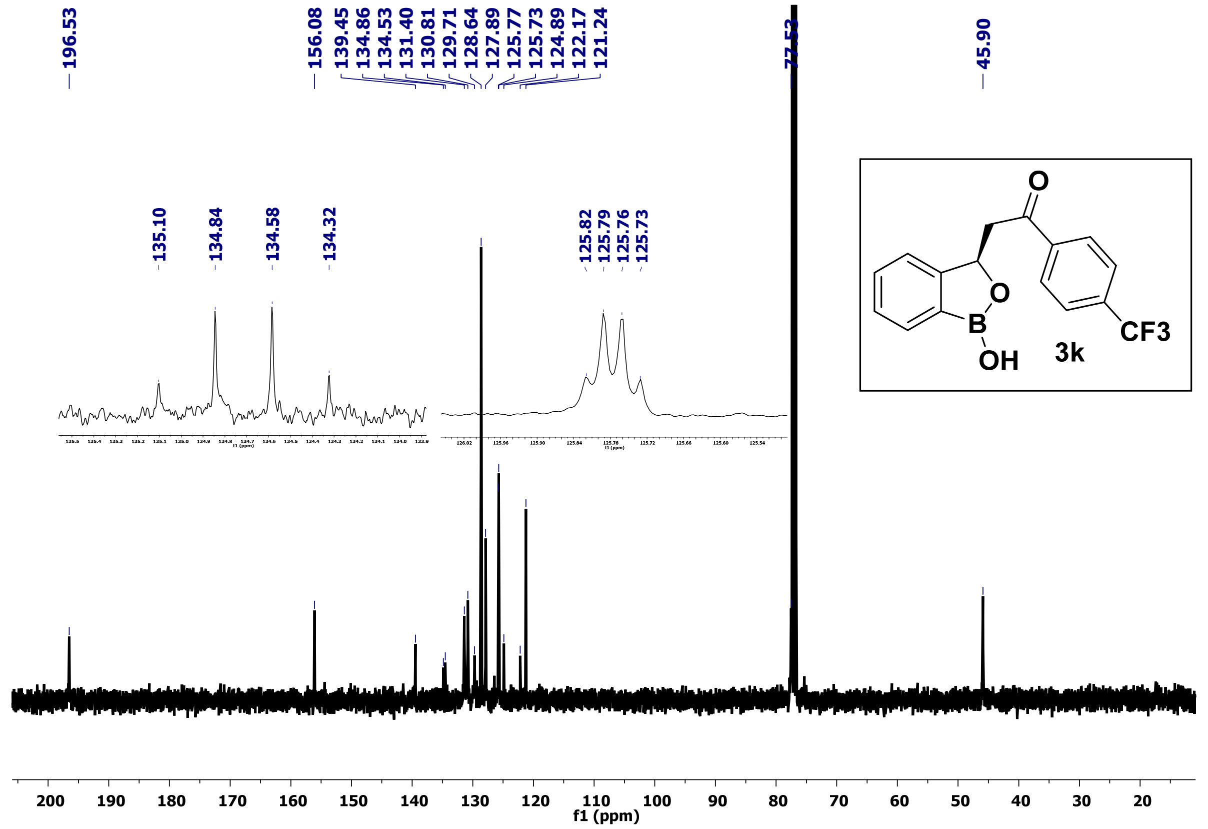
196.53
156.08 139.45 134.86 134.53 131.40 130.81 129.71 128.64 127.89 125.77 125.73 124.89 122.17 121.24
77.53
45.90
135.10 134.84 134.58 134.32
125.82 125.79 125.76 125.73
O
O
B
OH
3k
CF3
f1 (ppm)

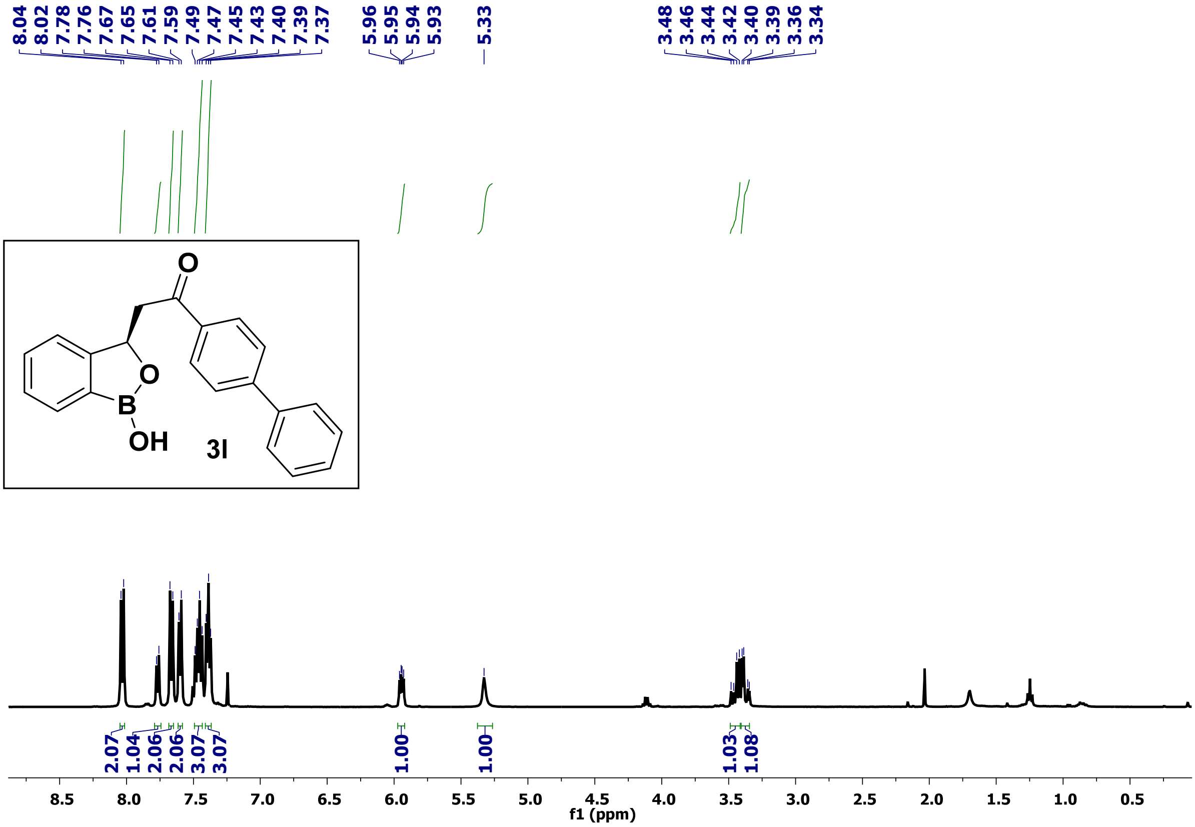
8.04 8.02 7.78 7.76 7.67 7.65 7.61 7.59 7.49 7.47 7.45 7.43 7.40 7.39 7.37
5.96 5.95 5.94 5.93
5.33
3.48 3.46 3.44 3.42 3.40 3.39 3.36 3.34
O
O
B
OH
3l
2.07 1.04 2.06 3.07 3.07
1.00
1.00
1.03 1.08
8.5 8.0 7.5 7.0 6.5 6.0 5.5 5.0 4.5 4.0 3.5 3.0 2.5 2.0 1.5 1.0 0.5
f1 (ppm)

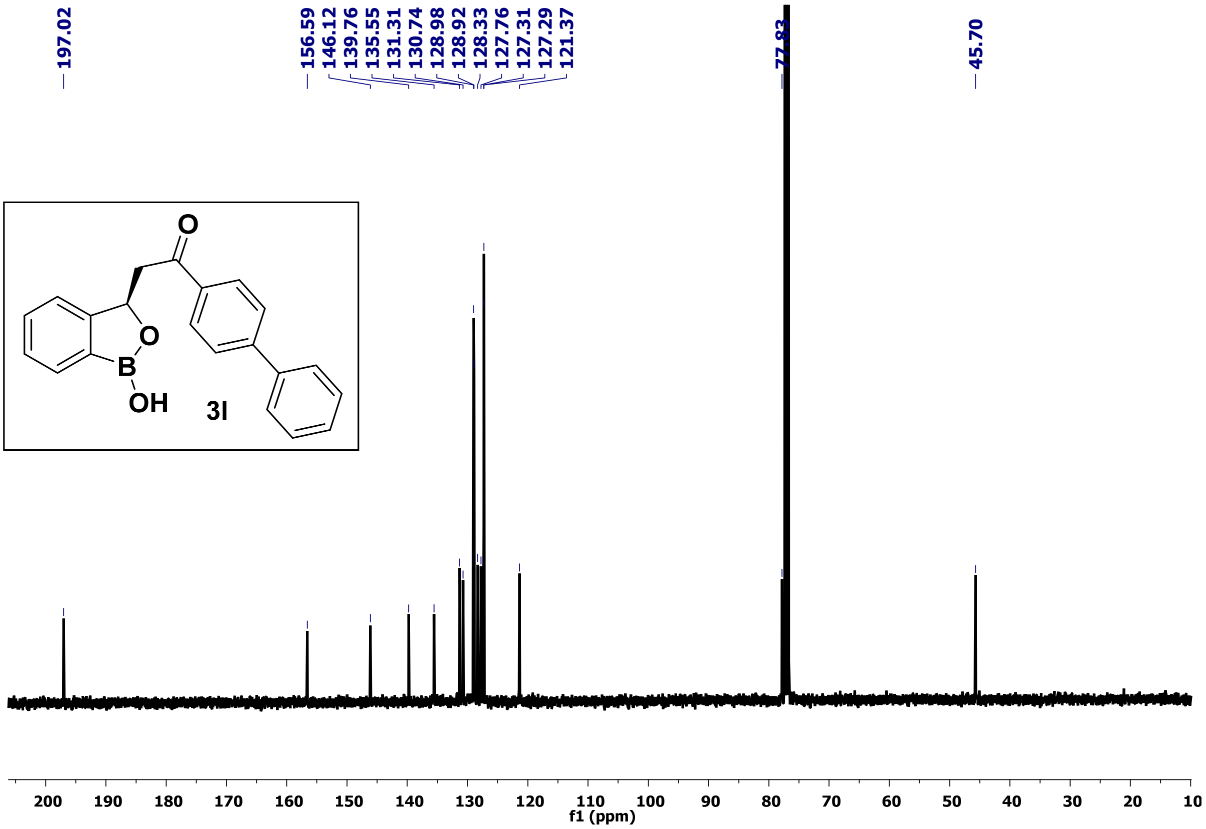
197.02
156.59 146.12 139.76 135.55 131.31 130.74 128.98 128.92 128.33 127.76 127.31 127.29 121.37
77.83
45.70
O
O
B
OH
3l
200 190 180 170 160 150 140 130 120 110 100 90 80 70 60 50 40 30 20 10
f1 (ppm)

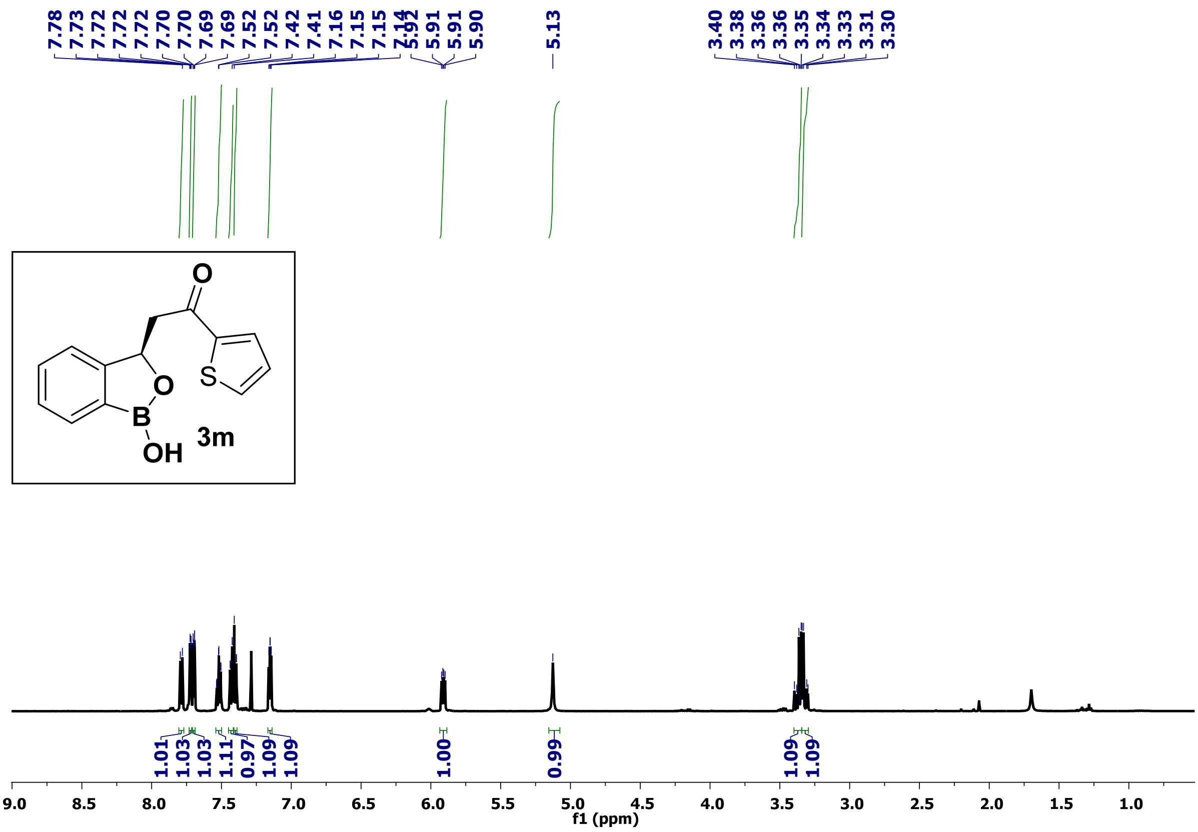
7.78
7.73
7.72
7.72
7.72
7.70
7.70
7.69
7.69
7.52
7.52
7.42
7.41
7.16
7.15
7.14
5.92
5.91
5.91
5.90
5.13
3.40
3.38
3.36
3.35
3.34
3.33
3.31
3.30
O
S
O
B
OH
3m
1.01
1.03
1.03
1.11
0.97
1.09
1.09
1.00
0.99
1.09
1.09
f1 (ppm)

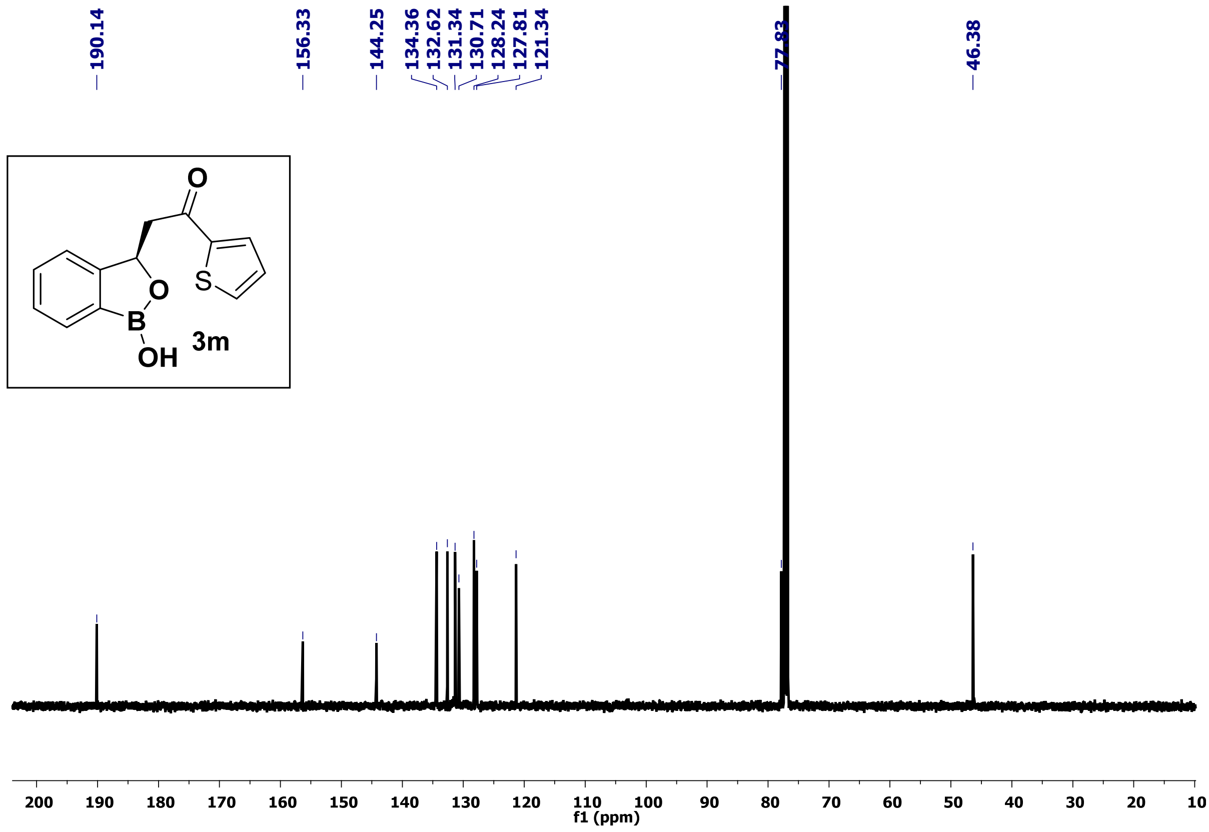
190.14
156.33
144.25
134.36
132.62
131.34
130.71
128.24
127.81
121.34
77.83
46.38
O
S
O
B
OH
3m
f1 (ppm)

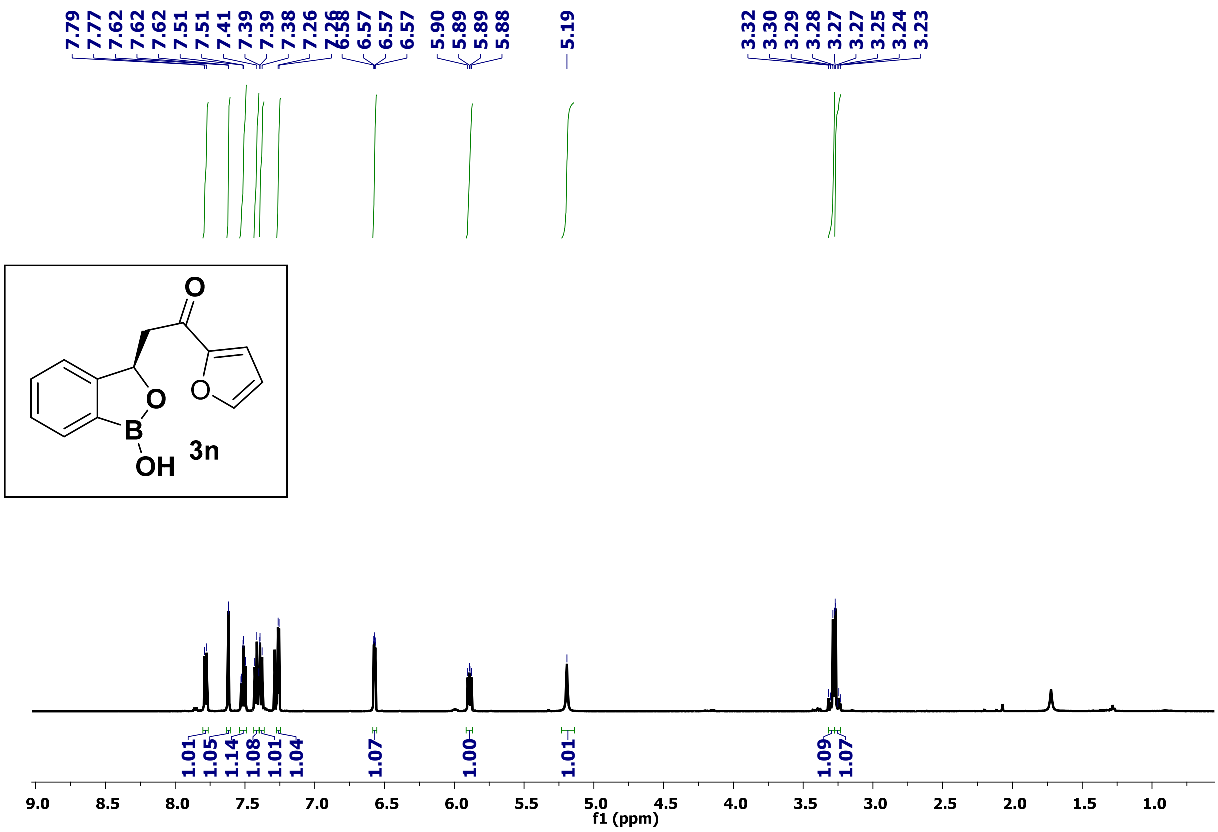
7.79 7.77 7.62 7.62 7.62 7.51 7.51 7.41 7.39 7.39 7.38 7.26 7.26 6.58 6.57 6.57 6.57 5.90 5.89 5.89 5.88
5.19
3.32 3.30 3.29 3.28 3.27 3.27 3.25 3.24 3.23
O
O
O
B
OH
3n
1.01 1.05 1.14 1.08 1.01 1.04 1.07 1.00 1.01 1.09 1.07
9.0 8.5 8.0 7.5 7.0 6.5 6.0 5.5 5.0 4.5 4.0 3.5 3.0 2.5 2.0 1.5 1.0
f1 (ppm)

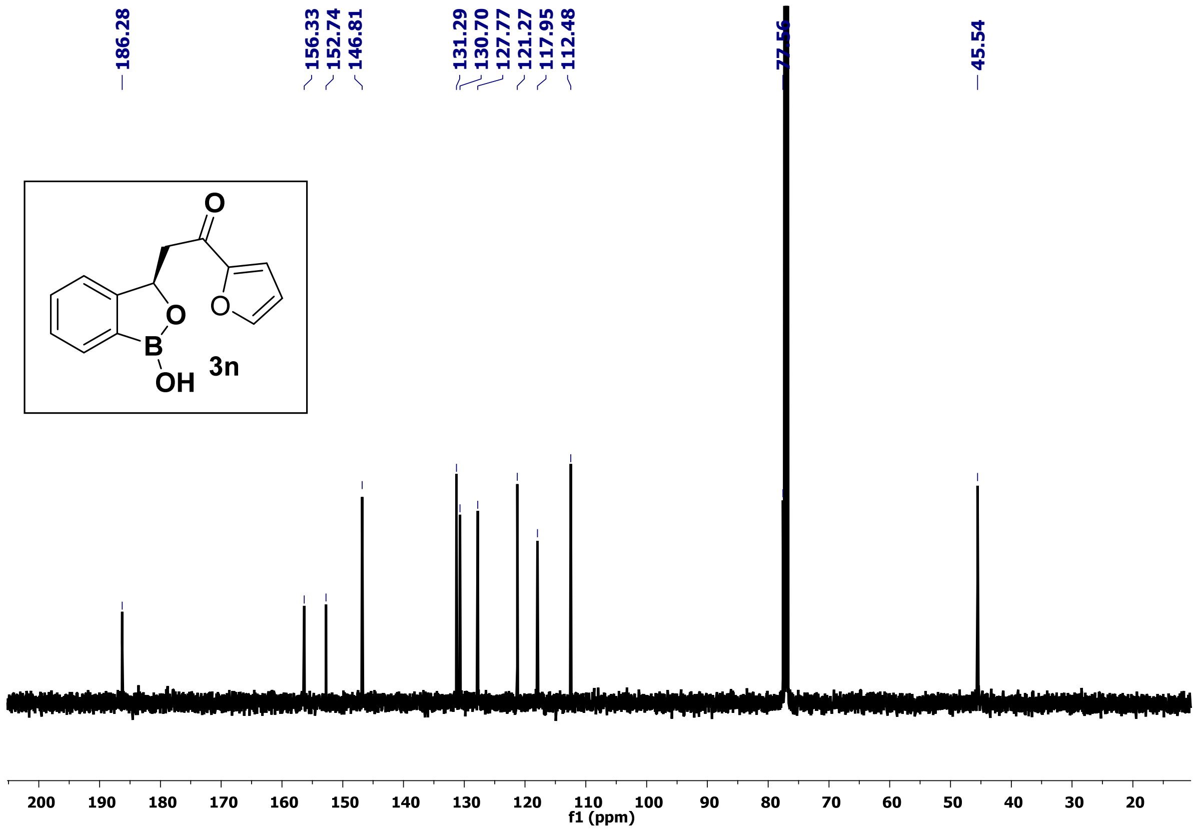
186.28
156.33 152.74 146.81
131.29 130.70 127.77 121.27 117.95 112.48
77.56
45.54
O
O
O
B
OH
3n
200 190 180 170 160 150 140 130 120 110 100 90 80 70 60 50 40 30 20
f1 (ppm)

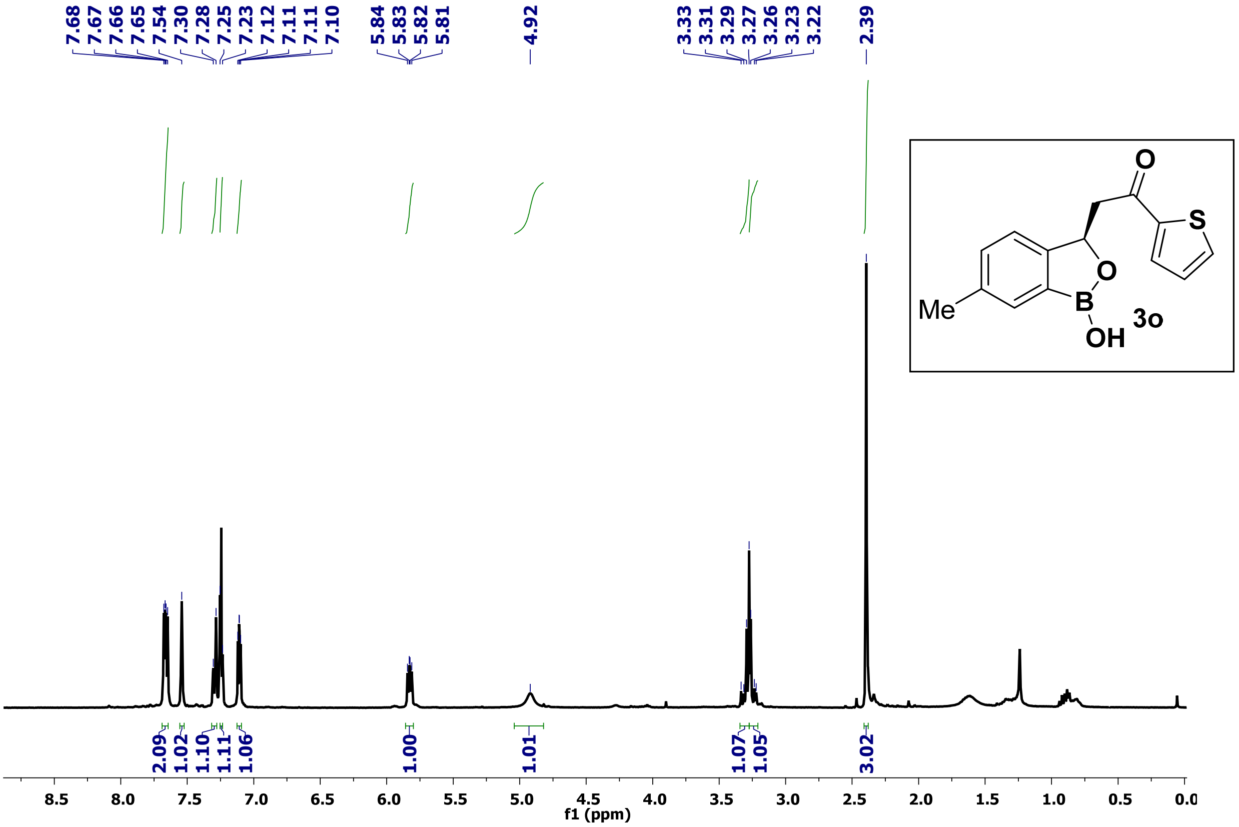
7.68
7.67
7.66
7.65
7.54
7.30
7.28
7.25
7.23
7.12
7.11
7.11
7.10
5.84
5.83
5.82
5.81
4.92
3.33
3.31
3.29
3.27
3.26
3.23
3.22
2.39
O
S
O
Me
B
OH
3o
2.09
1.02
1.10
1.11
1.06
1.00
1.01
1.07
1.05
3.02
f1 (ppm)

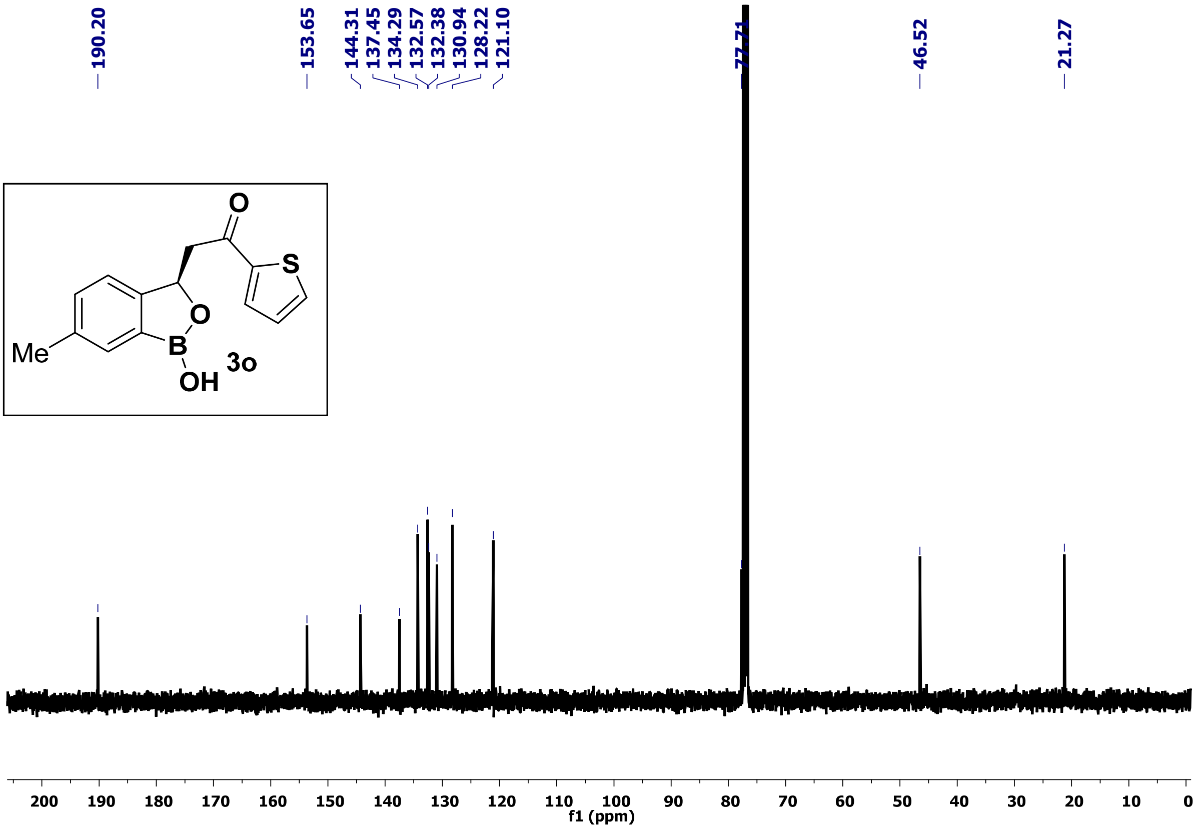
190.20
153.65
144.31
137.45
134.29
132.57
132.38
130.94
128.22
121.10
77.71
46.52
21.27
O
S
O
Me
B
OH
3o
f1 (ppm)

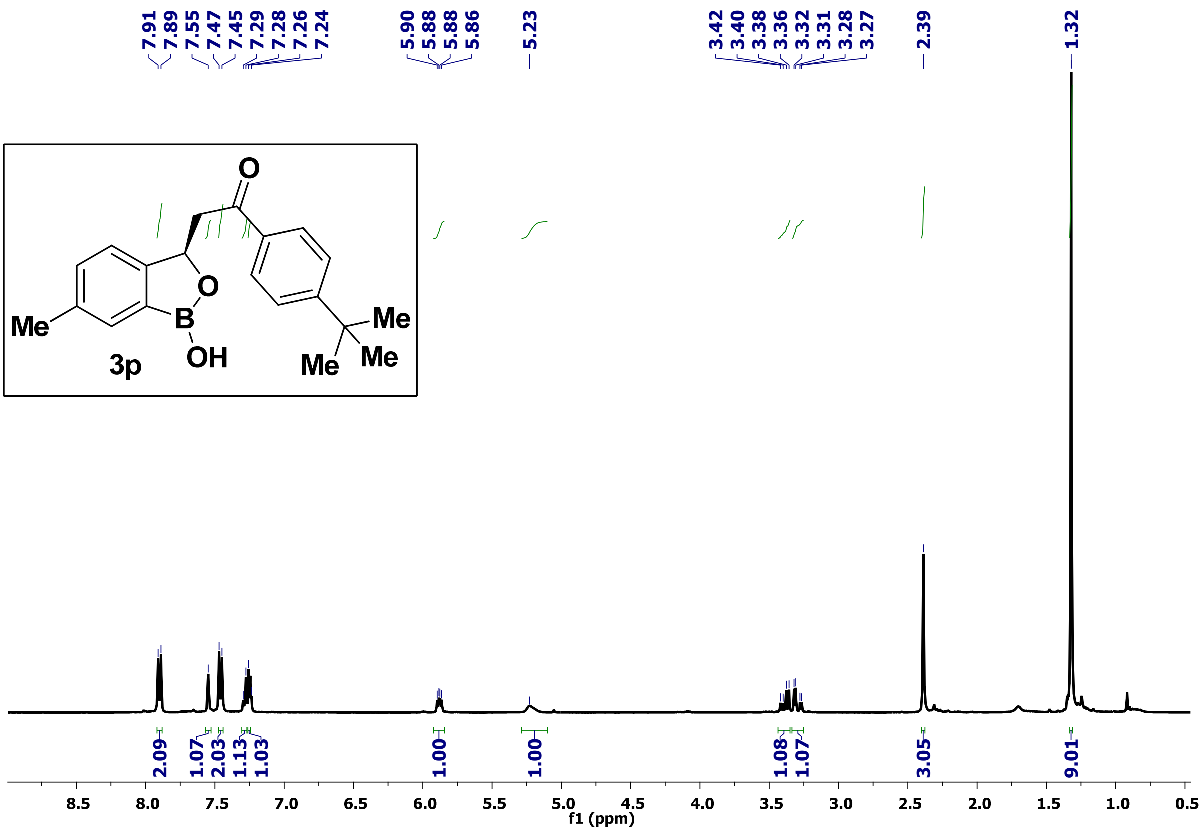
7.91 7.89 7.55 7.47 7.45 7.29 7.28 7.26 7.24
5.90 5.88 5.88 5.86
5.23
3.42 3.40 3.38 3.36 3.32 3.31 3.28 3.27
2.39
1.32
O
O
B
OH
Me
3p
Me
Me
Me
2.09 1.07 2.03 1.13 1.03
1.00
1.00
1.08 1.07
3.05
9.01
8.5 8.0 7.5 7.0 6.5 6.0 5.5 5.0 4.5 4.0 3.5 3.0 2.5 2.0 1.5 1.0 0.5
f1 (ppm)

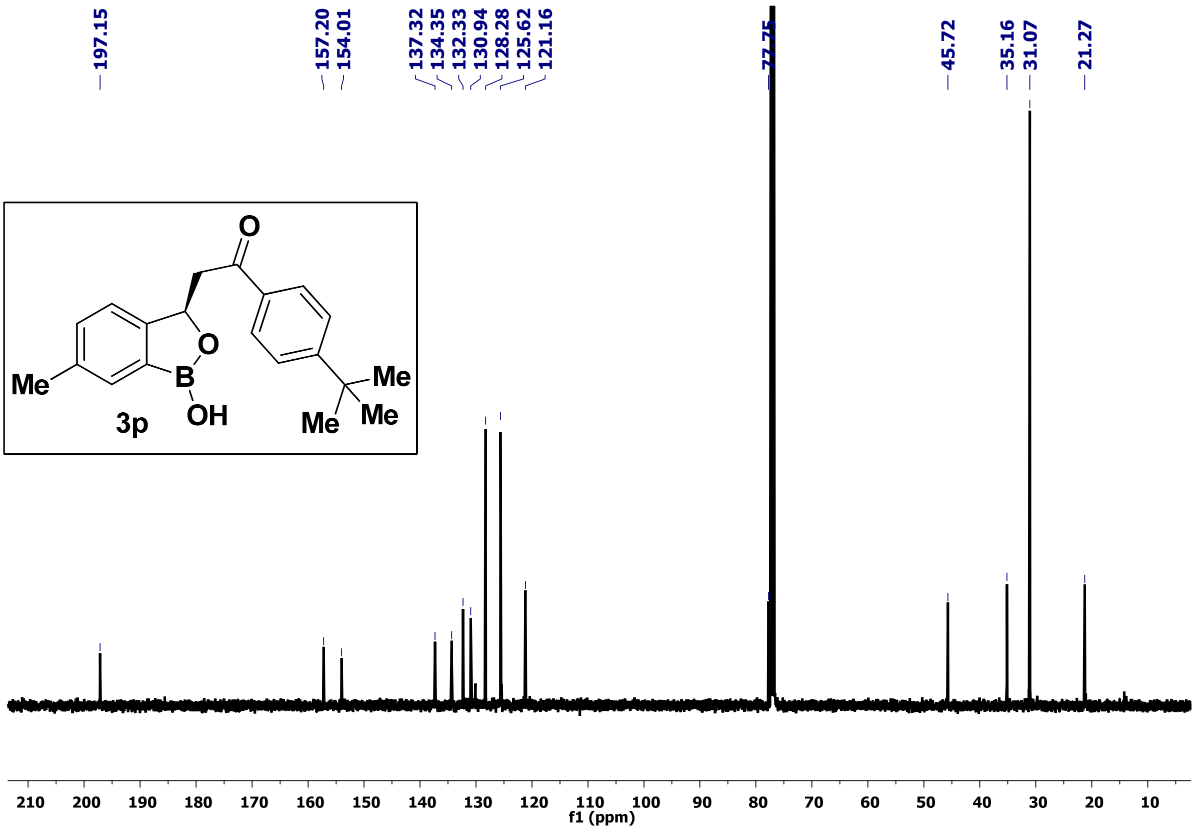
197.15
157.20 154.01
137.32 134.35 132.33 130.94 128.28 125.62 121.16
77.75
45.72
35.16 31.07
21.27
O
O
B
OH
Me
3p
Me
Me
Me
210 200 190 180 170 160 150 140 130 120 110 100 90 80 70 60 50 40 30 20 10
f1 (ppm)

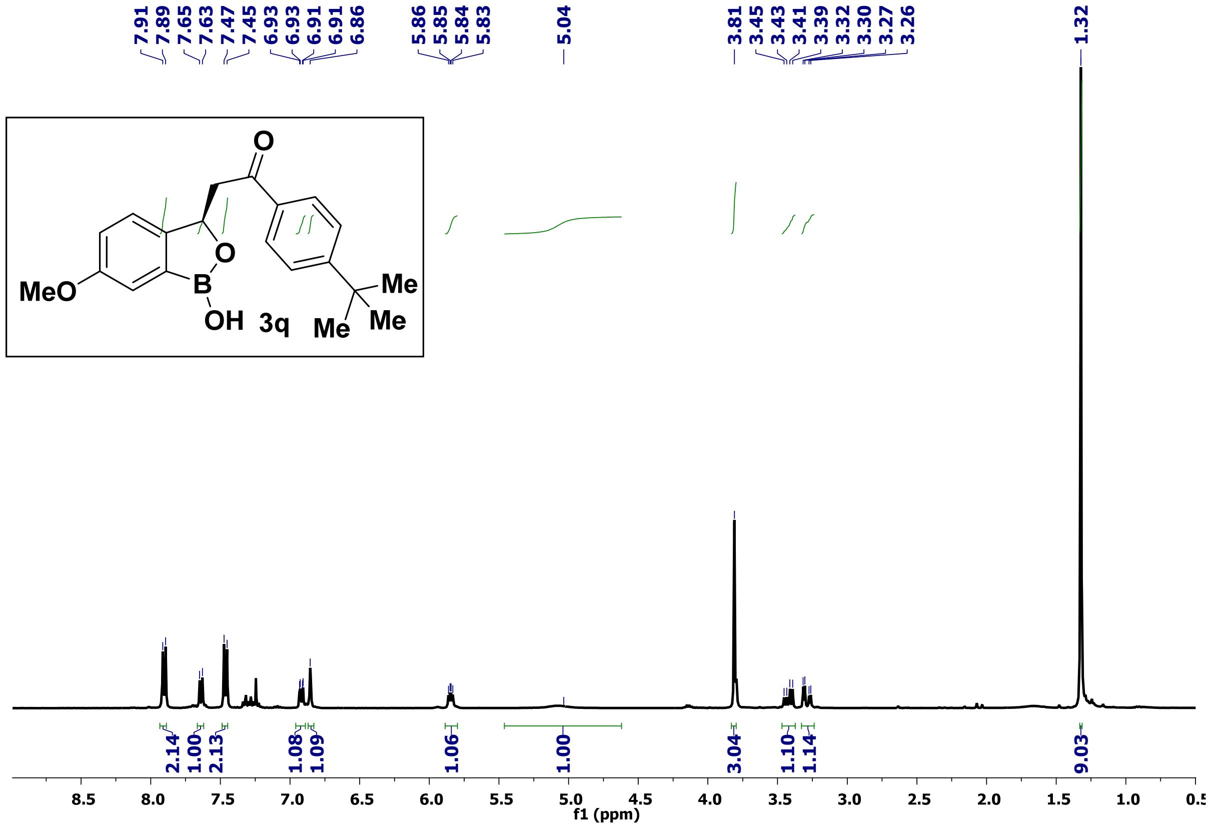
7.91 7.89 7.65 7.63 7.47 7.45 6.93 6.93 6.91 6.91 6.86
5.86 5.85 5.84 5.83
5.04
3.81 3.45 3.43 3.41 3.39 3.32 3.30 3.27 3.26
1.32
O
MeO
B
O
OH
3q
Me
Me
Me
2.14 1.00 2.13 1.08 1.09 1.06 1.00 3.04 1.10 1.14 9.03
f1 (ppm)

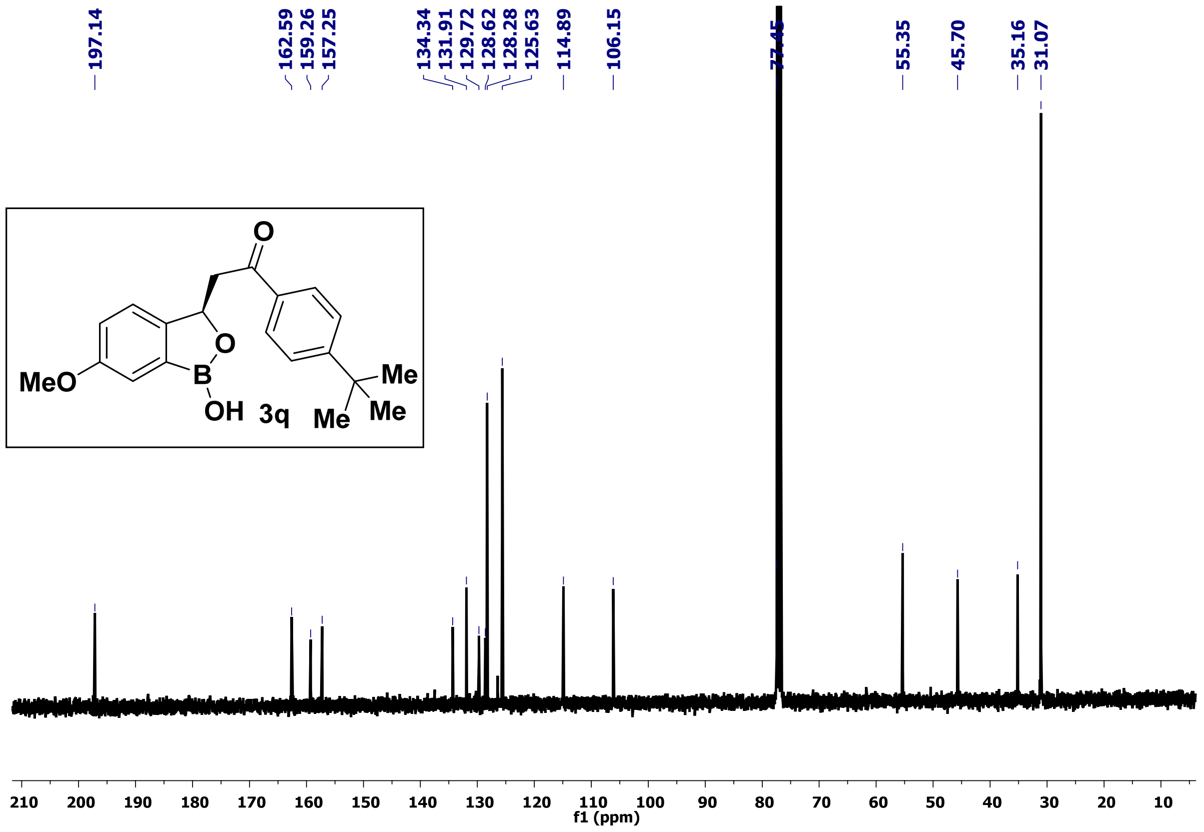
197.14
162.59 159.26 157.25
134.34 131.91 129.72 128.62 128.28 125.63
114.89
106.15
77.45
55.35
45.70
35.16 31.07
O
MeO
B
O
OH
3q
Me
Me
Me
f1 (ppm)

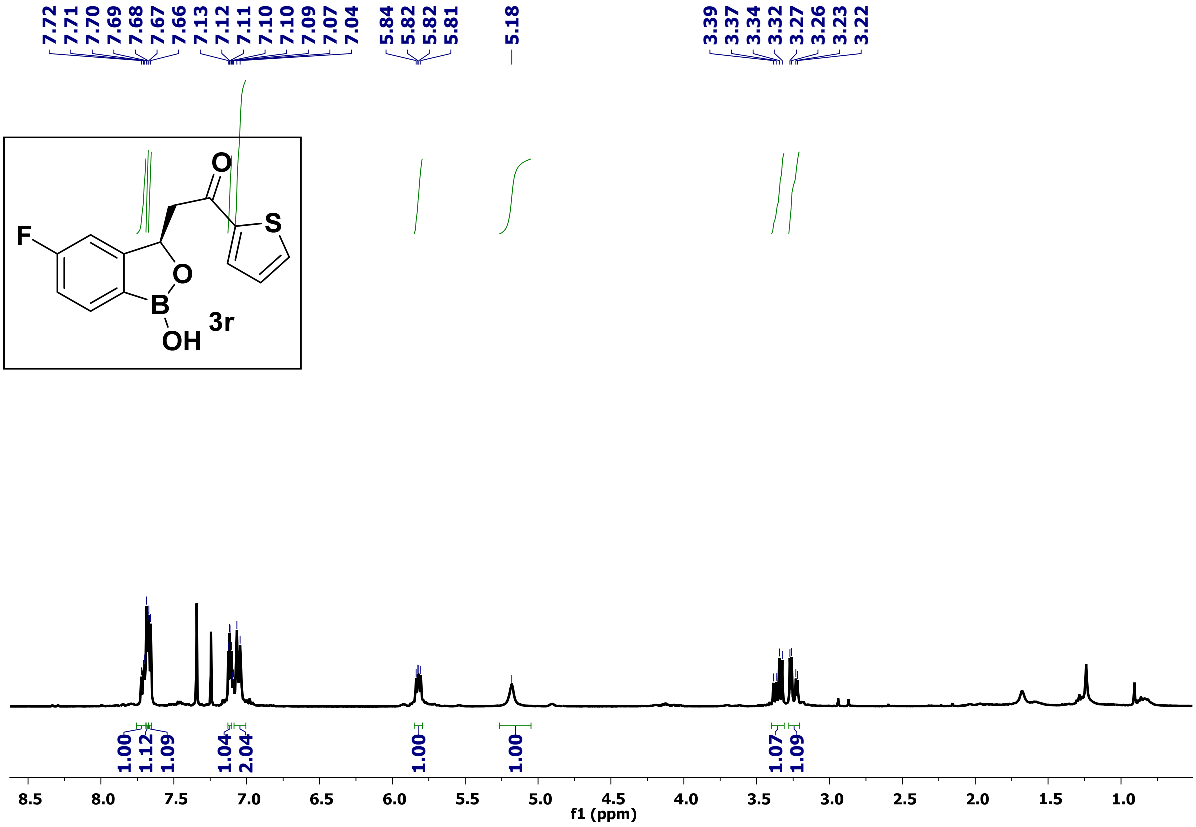
7.72 7.71 7.70 7.69 7.68 7.67 7.66 7.13 7.12 7.11 7.10 7.10 7.09 7.07 7.04
5.84 5.82 5.82 5.81
5.18
3.39 3.37 3.34 3.32 3.27 3.26 3.23 3.22
O
S
F
O
B
OH
3r
1.00 1.12 1.09 1.04 2.04 1.00 1.00 1.07 1.09
8.5 8.0 7.5 7.0 6.5 6.0 5.5 5.0 4.5 4.0 3.5 3.0 2.5 2.0 1.5 1.0
f1 (ppm)

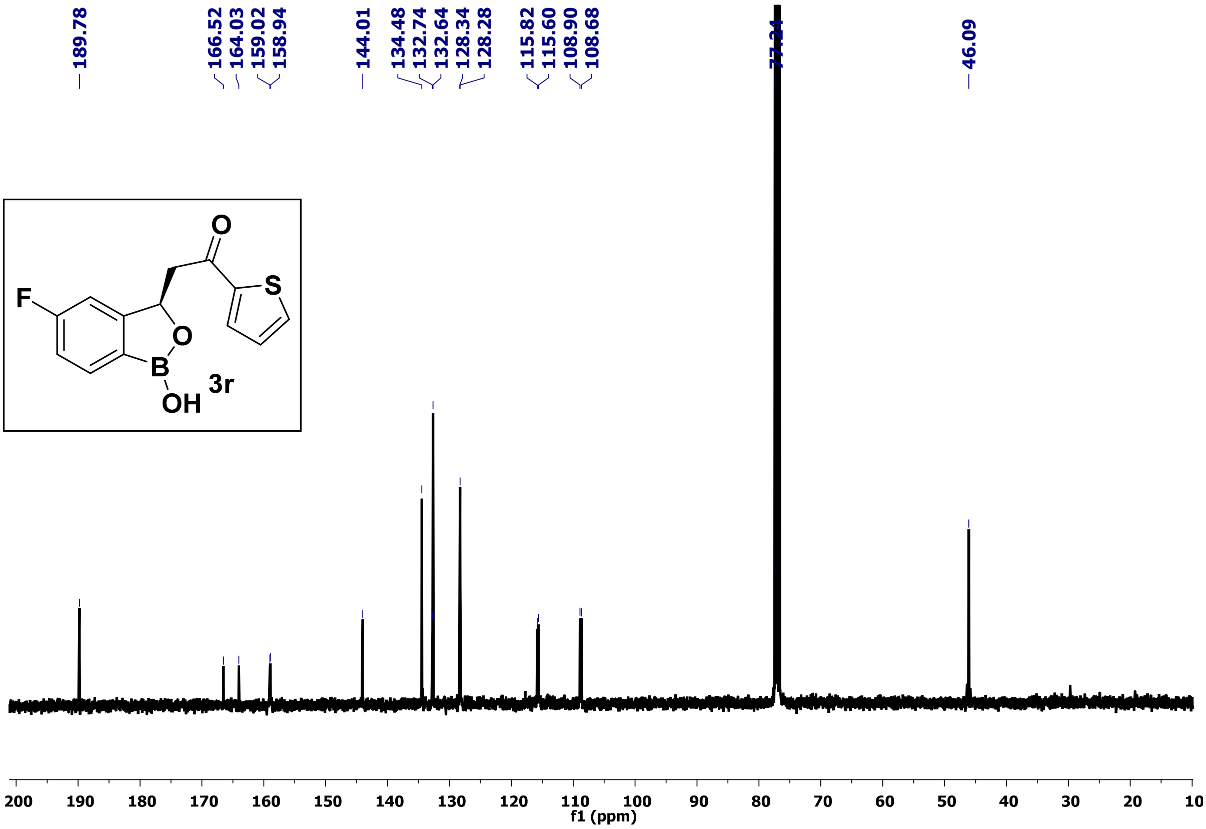
189.78
166.52 164.03 159.02 158.94
144.01
134.48 132.74 132.64 128.34 128.28
115.82 115.60 108.90 108.68
77.24
46.09
O
S
F
O
B
OH
3r
200 190 180 170 160 150 140 130 120 110 100 90 80 70 60 50 40 30 20 10
f1 (ppm)

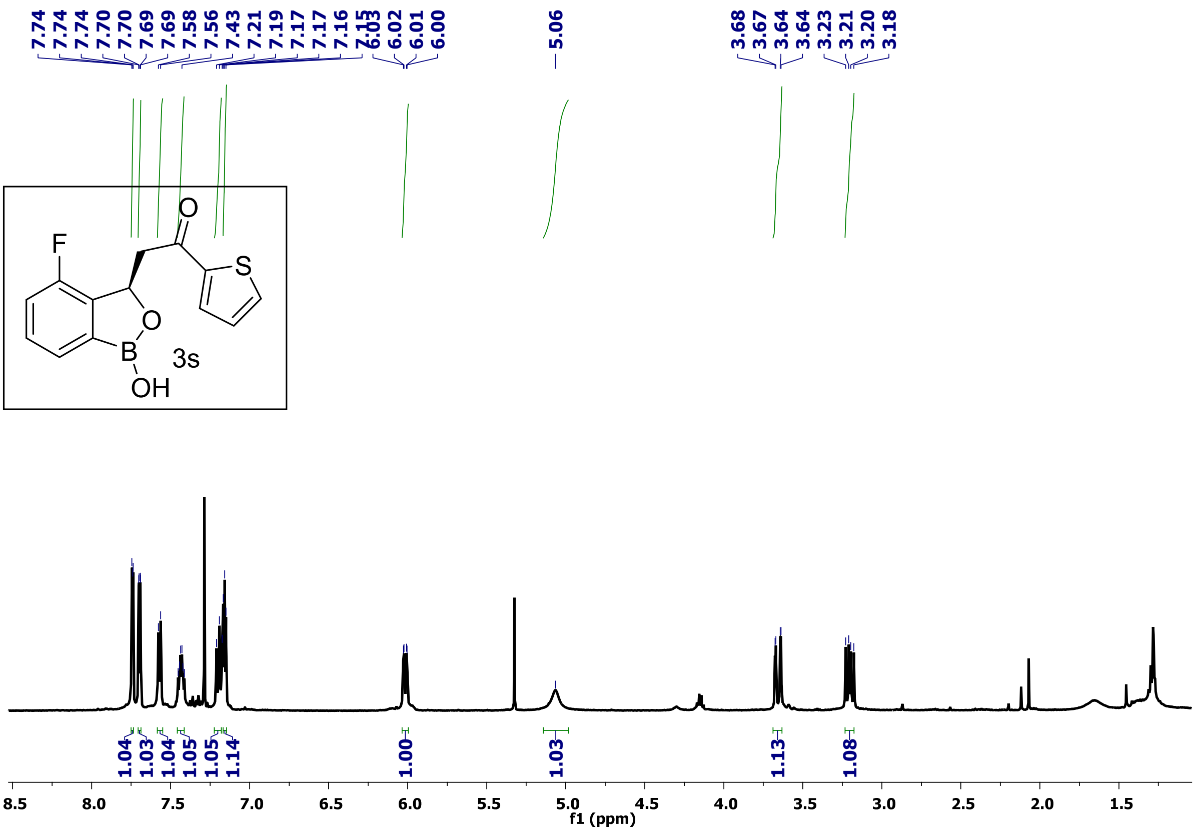
7.74
7.74
7.74
7.70
7.69
7.69
7.58
7.56
7.43
7.21
7.19
7.17
7.17
7.16
7.15
6.03
6.02
6.01
6.00
5.06
3.68
3.67
3.64
3.64
3.23
3.21
3.20
3.18
F
O
S
O
B
3s
OH
1.04
1.03
1.04
1.05
1.05
1.14
1.00
1.03
1.13
1.08
8.5
8.0
7.5
7.0
6.5
6.0
5.5
5.0
4.5
4.0
3.5
3.0
2.5
2.0
1.5
f1 (ppm)

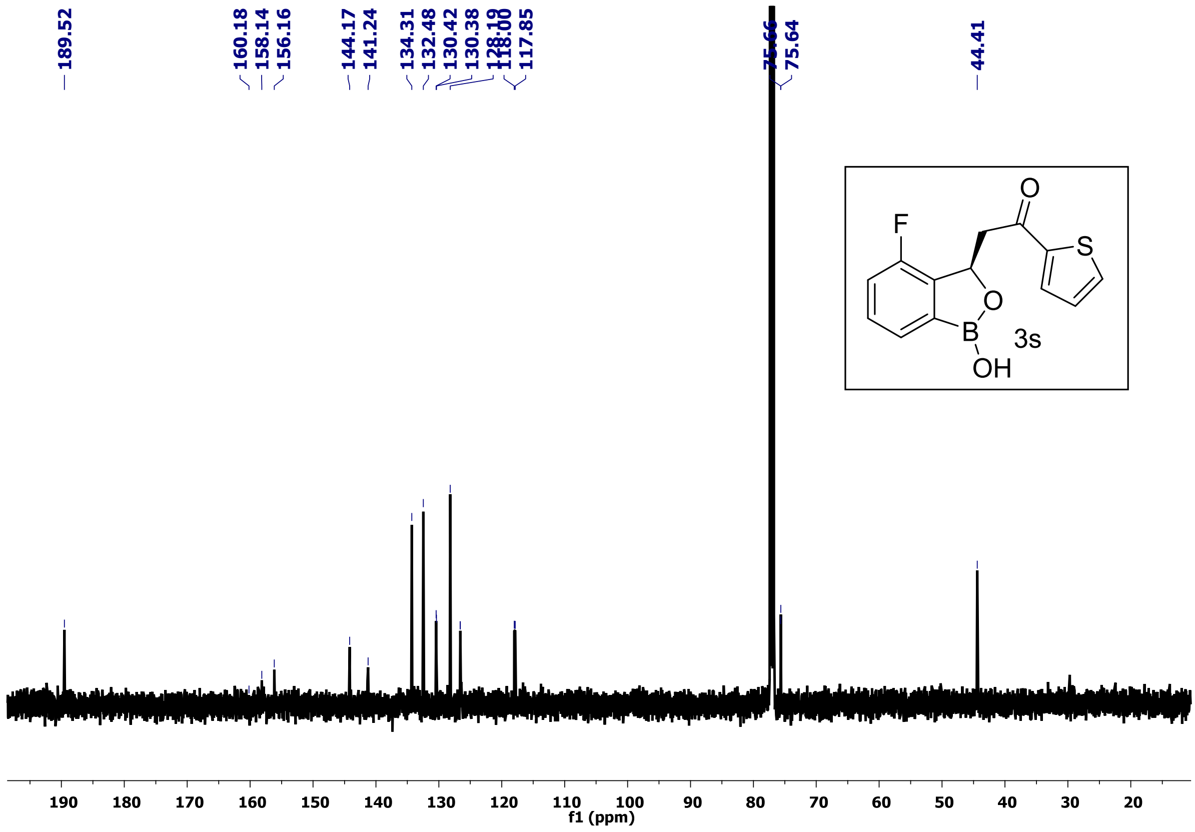
189.52
160.18
158.14
156.16
144.17
141.24
134.31
132.48
130.42
130.38
128.19
118.00
117.85
75.66
75.64
44.41
F
O
S
O
B
3s
OH
190
180
170
160
150
140
130
120
110
100
90
80
70
60
50
40
30
20
f1 (ppm)

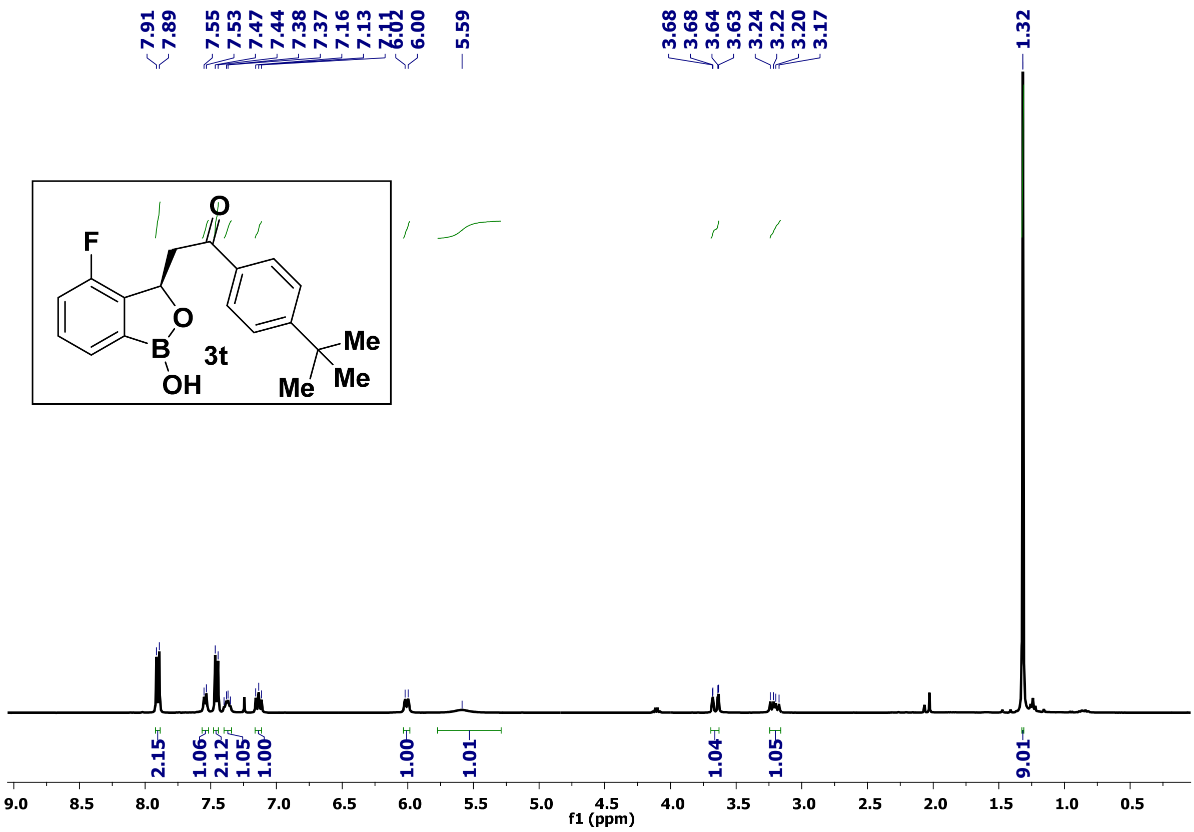
7.91 7.89 7.55 7.53 7.47 7.44 7.38 7.37 7.16 7.13 7.11 6.02 6.00 5.59 3.68 3.68 3.64 3.63 3.24 3.22 3.20 3.17 1.32
F
O
B
OH
3t
Me
Me
Me
2.15 1.06 2.12 1.05 1.00 1.00 1.01 1.04 1.05 9.01
9.0 8.5 8.0 7.5 7.0 6.5 6.0 5.5 5.0 4.5 4.0 3.5 3.0 2.5 2.0 1.5 1.0 0.5
f1 (ppm)

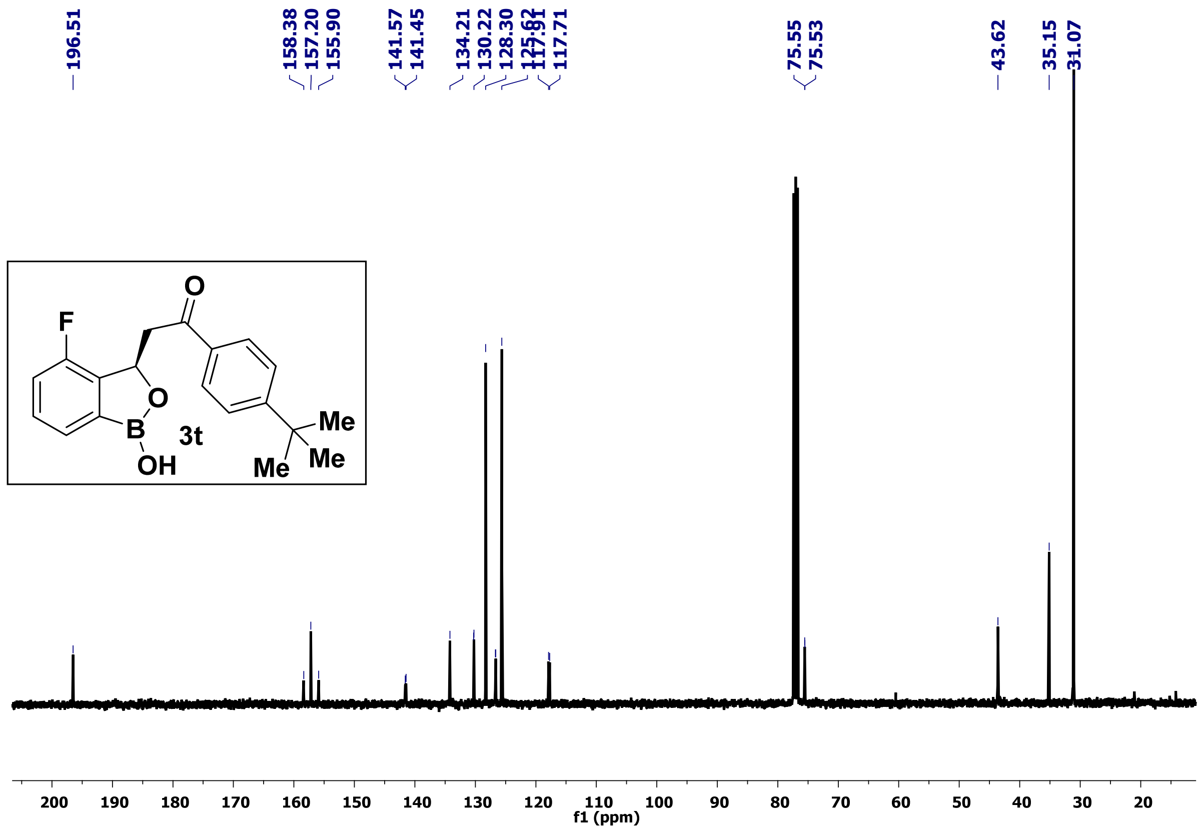
196.51 158.38 157.20 155.90 141.57 141.45 134.21 130.22 128.30 117.71 75.55 75.53 43.62 35.15 31.07
F
O
B
OH
3t
Me
Me
Me
200 190 180 170 160 150 140 130 120 110 100 90 80 70 60 50 40 30 20
f1 (ppm)

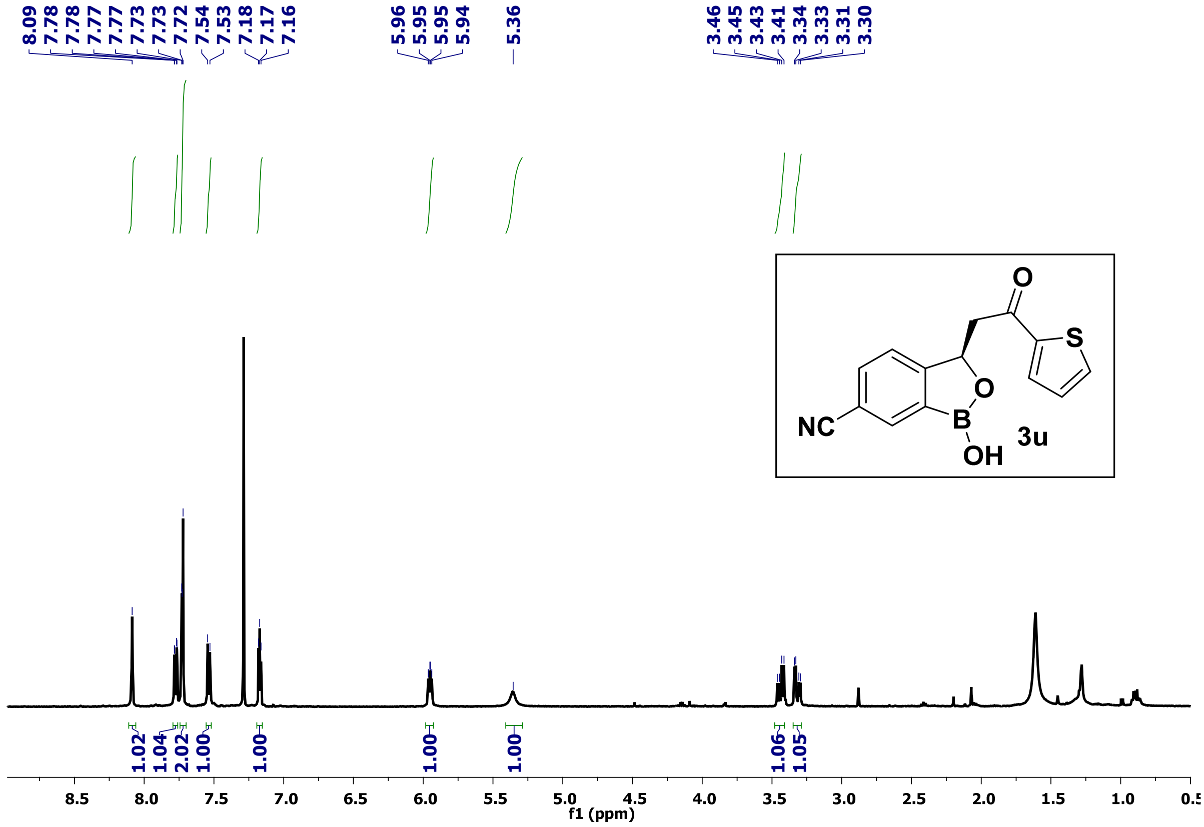
8.09 7.78 7.78 7.77 7.77 7.73 7.73 7.72 7.54 7.53 7.18 7.17 7.16
5.96 5.95 5.95 5.94
5.36
3.46 3.45 3.43 3.41 3.34 3.33 3.31 3.30
O
S
O
B
NC
OH
3u
1.02
1.04
2.02
1.00
1.00
1.00
1.00
1.06
1.05
f1 (ppm)

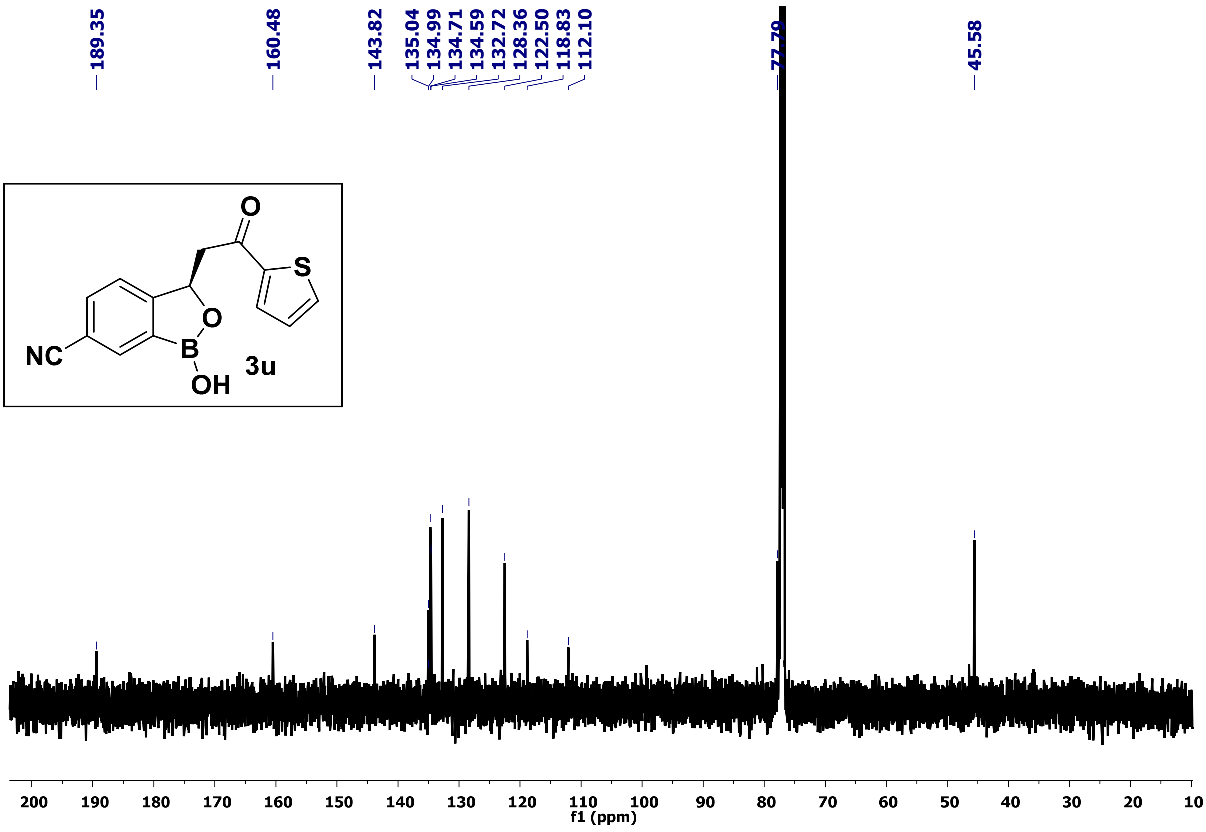
189.35
160.48
143.82
135.04 134.99 134.71 134.59 132.72 128.36 122.50 118.83 112.10
45.58
O
S
O
B
NC
OH
3u
f1 (ppm)

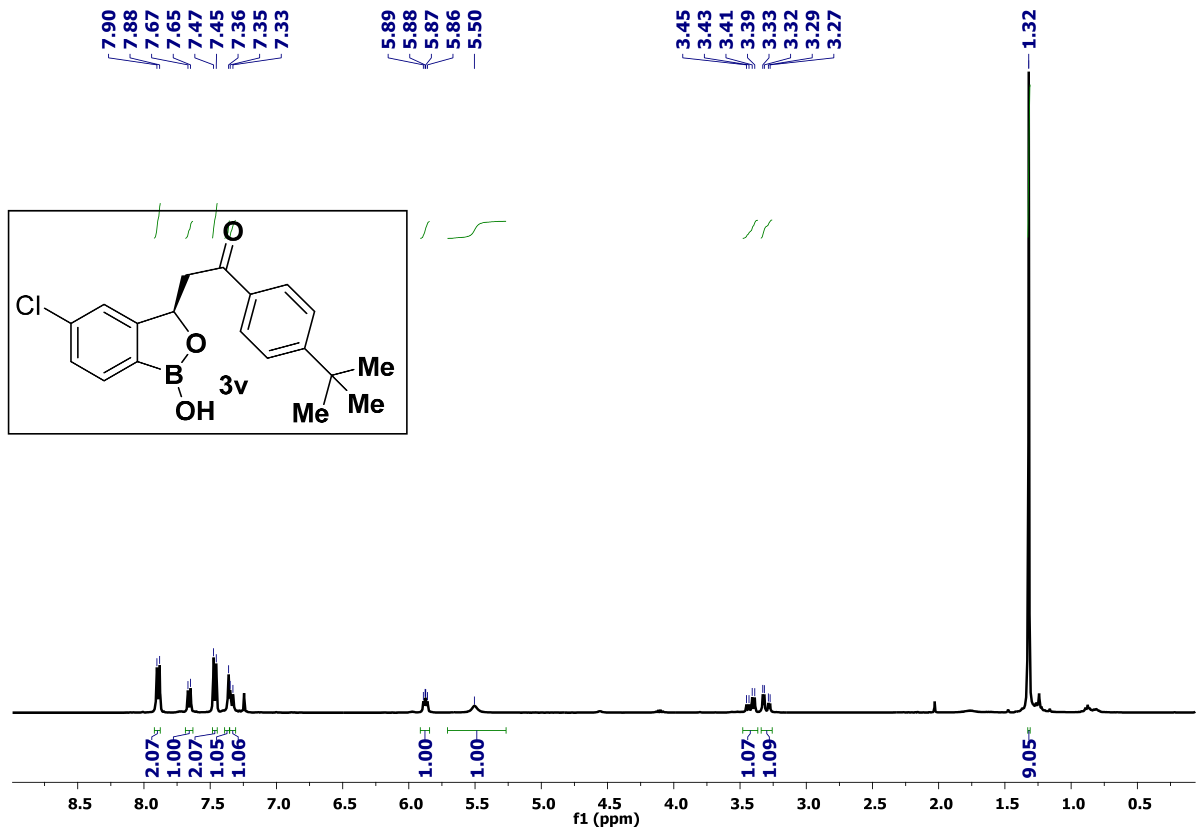
7.90
7.88
7.67
7.65
7.47
7.45
7.36
7.35
7.33
5.89
5.88
5.87
5.86
5.50
3.45
3.43
3.41
3.39
3.33
3.32
3.29
3.27
1.32
Cl
O
O
B
OH
3v
Me
Me
Me
2.07
1.00
2.07
1.05
1.06
1.00
1.00
1.07
1.09
9.05
8.5
8.0
7.5
7.0
6.5
6.0
5.5
5.0
4.5
4.0
3.5
3.0
2.5
2.0
1.5
1.0
0.5
f1 (ppm)

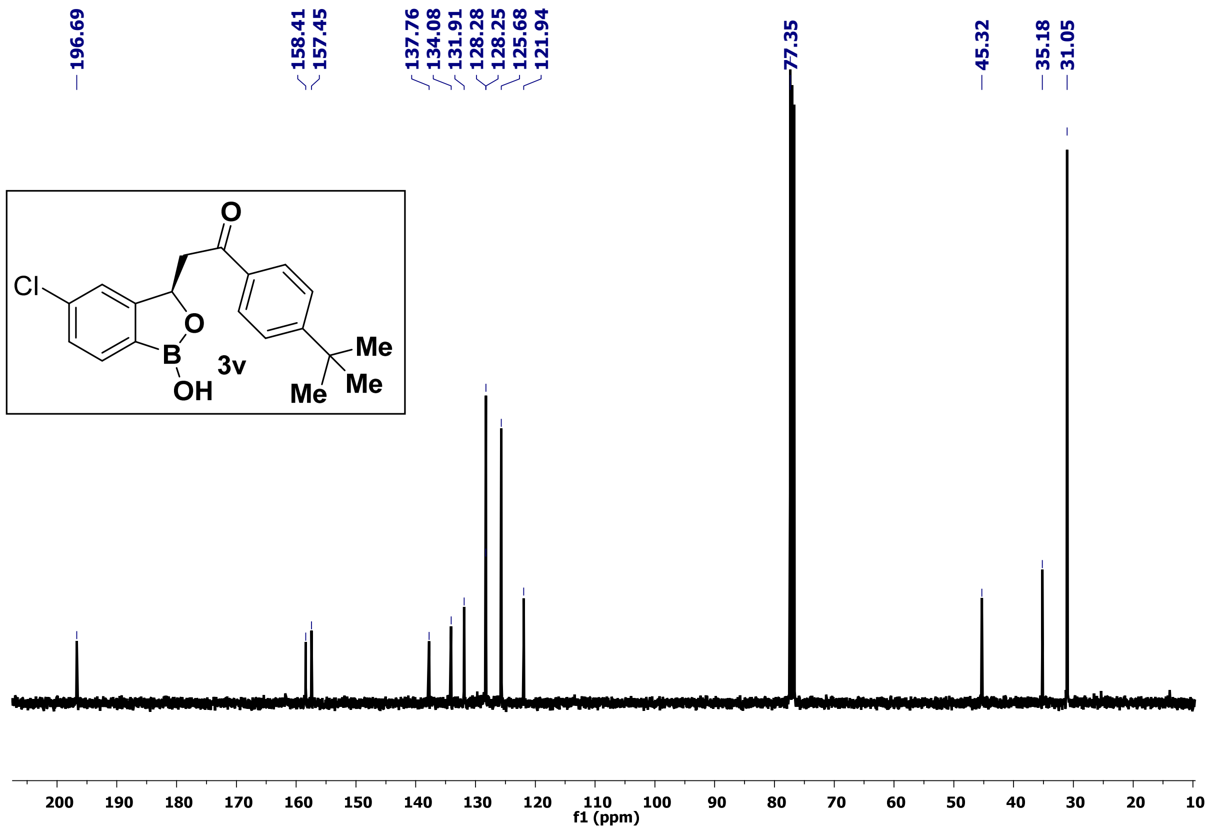
196.69
158.41
157.45
137.76
134.08
131.91
128.28
128.25
125.68
121.94
77.35
45.32
35.18
31.05
Cl
O
O
B
OH
3v
Me
Me
Me
200
190
180
170
160
150
140
130
120
110
100
90
80
70
60
50
40
30
20
10
f1 (ppm)

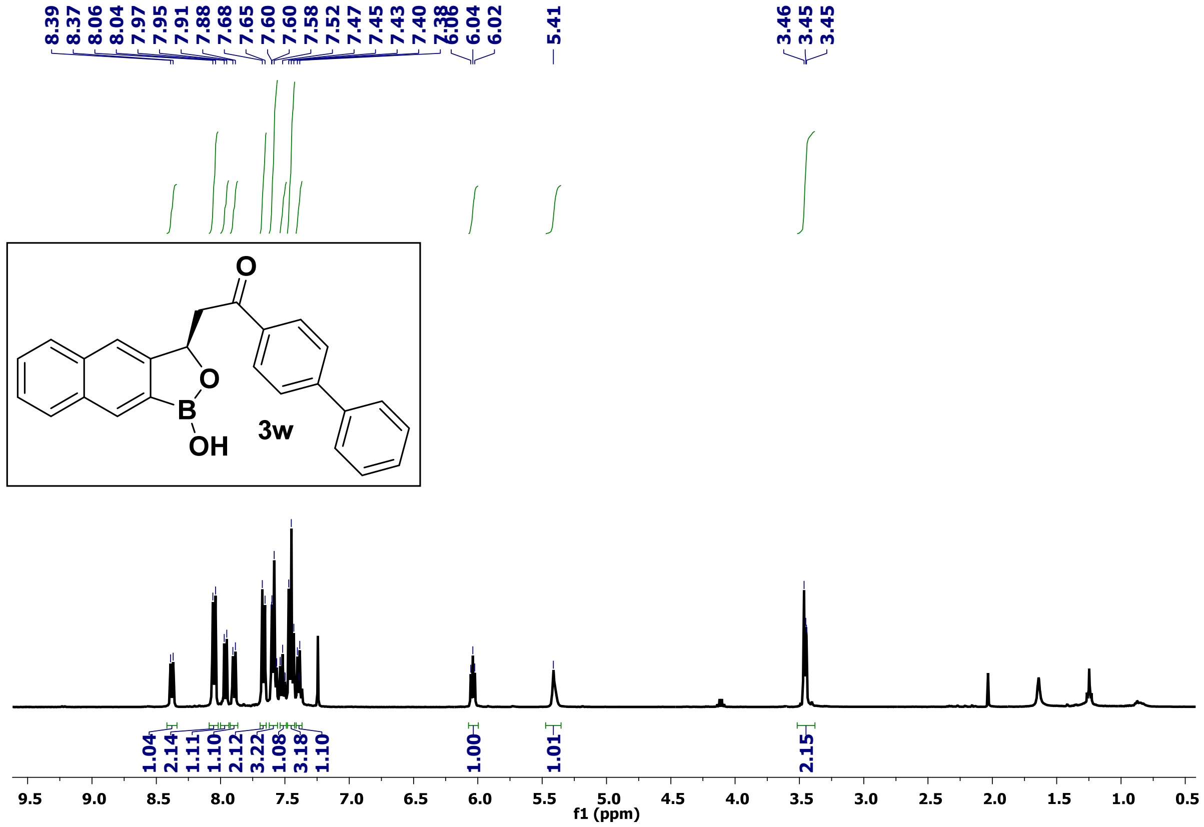
8.39 8.37 8.06 8.04 7.97 7.95 7.91 7.88 7.68 7.65 7.60 7.60 7.58 7.52 7.47 7.45 7.43 7.40 7.38 6.06 6.04 6.02
5.41
3.46 3.45 3.45
O
O
B
OH
3w
1.04 2.14 1.11 1.10 2.12 3.22 1.08 3.18 1.10
1.00
1.01
2.15
9.5 9.0 8.5 8.0 7.5 7.0 6.5 6.0 5.5 5.0 4.5 4.0 3.5 3.0 2.5 2.0 1.5 1.0 0.5
f1 (ppm)

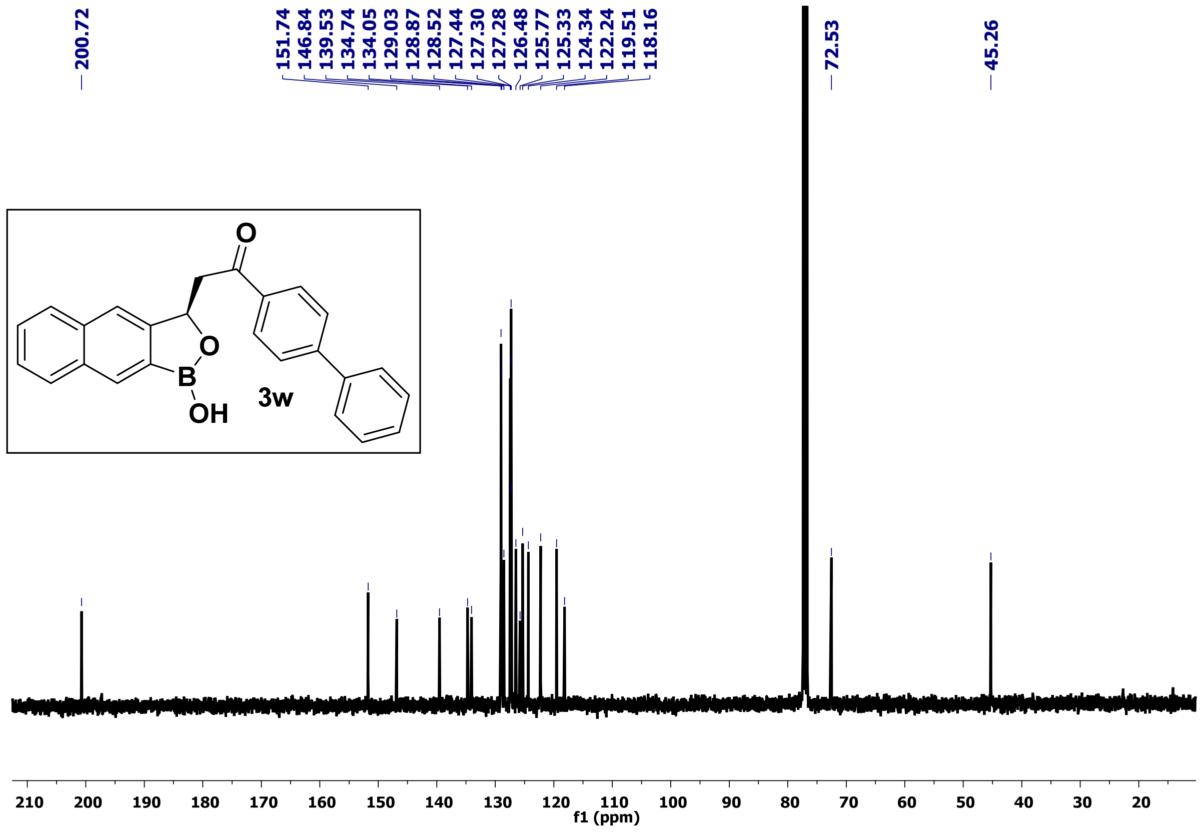
200.72
151.74 146.84 139.53 134.74 134.05 129.03 128.87 128.52 127.44 127.30 127.28 126.48 125.77 125.33 124.34 122.24 119.51 118.16
72.53
45.26
O
O
B
OH
3w
210 200 190 180 170 160 150 140 130 120 110 100 90 80 70 60 50 40 30 20
f1 (ppm)

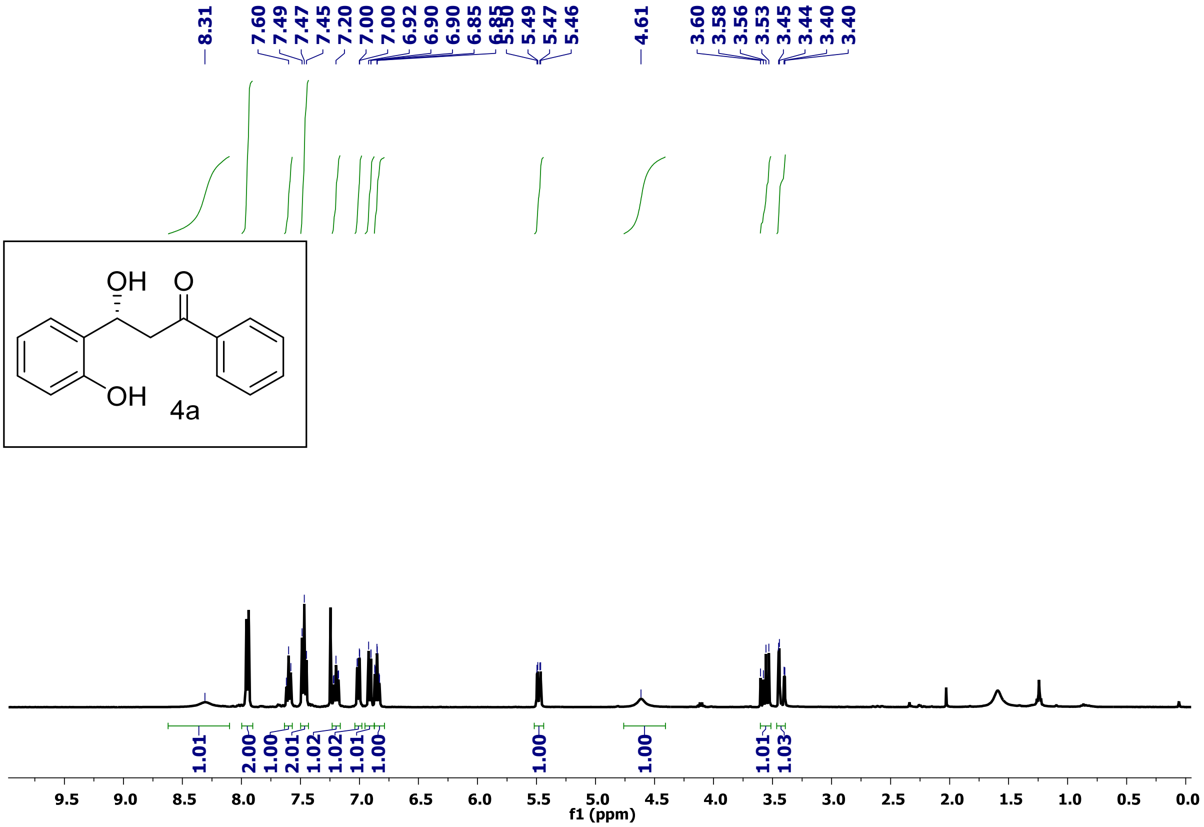
8.31
7.60 7.49 7.47 7.45 7.20 7.00 7.00 6.92 6.90 6.85 6.85 5.50 5.49 5.47 5.46
4.61
3.60 3.58 3.56 3.53 3.45 3.44 3.40 3.40
OH O
OH 4a
1.01 2.00 1.00 2.01 1.02 1.02 1.01 1.00 1.00 1.00 1.01 1.03
9.5 9.0 8.5 8.0 7.5 7.0 6.5 6.0 5.5 5.0 4.5 4.0 3.5 3.0 2.5 2.0 1.5 1.0 0.5 0.0
f1 (ppm)

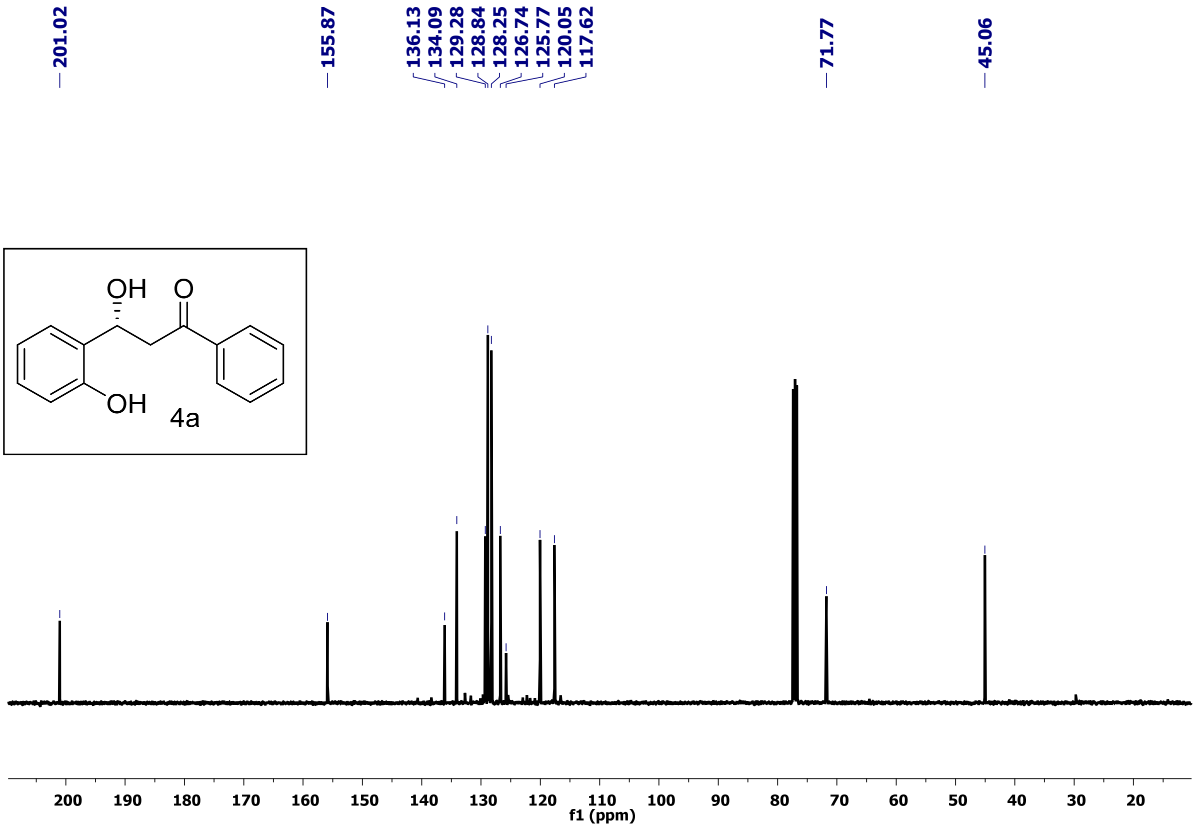
201.02
155.87
136.13 134.09 129.28 128.84 128.25 126.74 125.77 120.05 117.62
71.77
45.06
OH O
OH 4a
200 190 180 170 160 150 140 130 120 110 100 90 80 70 60 50 40 30 20
f1 (ppm)

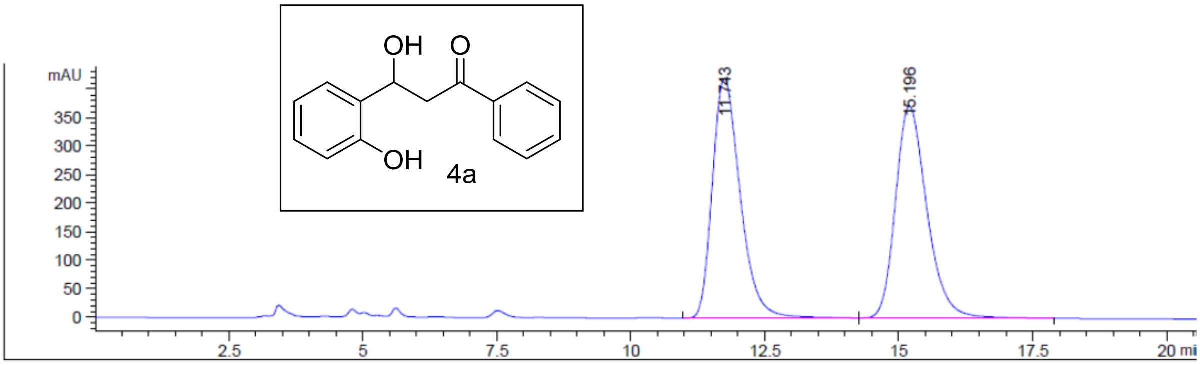

Signal 1: DAD1 A, Sig=254,4 Ref=360,100

Peak #	RetTime [min]	Type	Width [min]	Area [mAU*s]	Height [mAU]	Area %
1	11.743	BB	0.5590	1.52432e4	420.42453	50.0446
2	15.196	BB	0.6267	1.52160e4	372.35693	49.9554
Totals :				3.04593e4	792.78146	

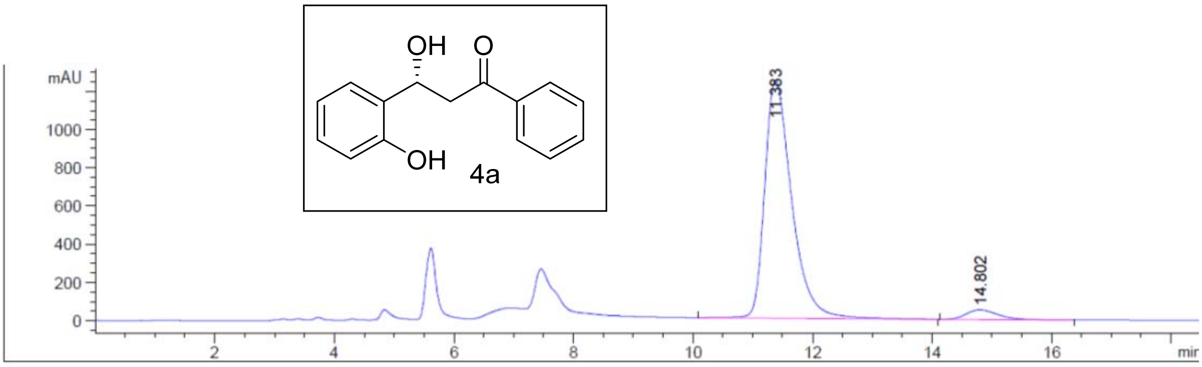

Signal 1: DAD1 A, Sig=254,4 Ref=360,100

Peak #	RetTime [min]	Type	Width [min]	Area [mAU*s]	Height [mAU]	Area %
1	11.383	BB	0.4789	3.91062e4	1243.36951	95.4962
2	14.802	BB	0.5548	1844.33655	50.64859	4.5038
Totals :				4.09505e4	1294.01810	

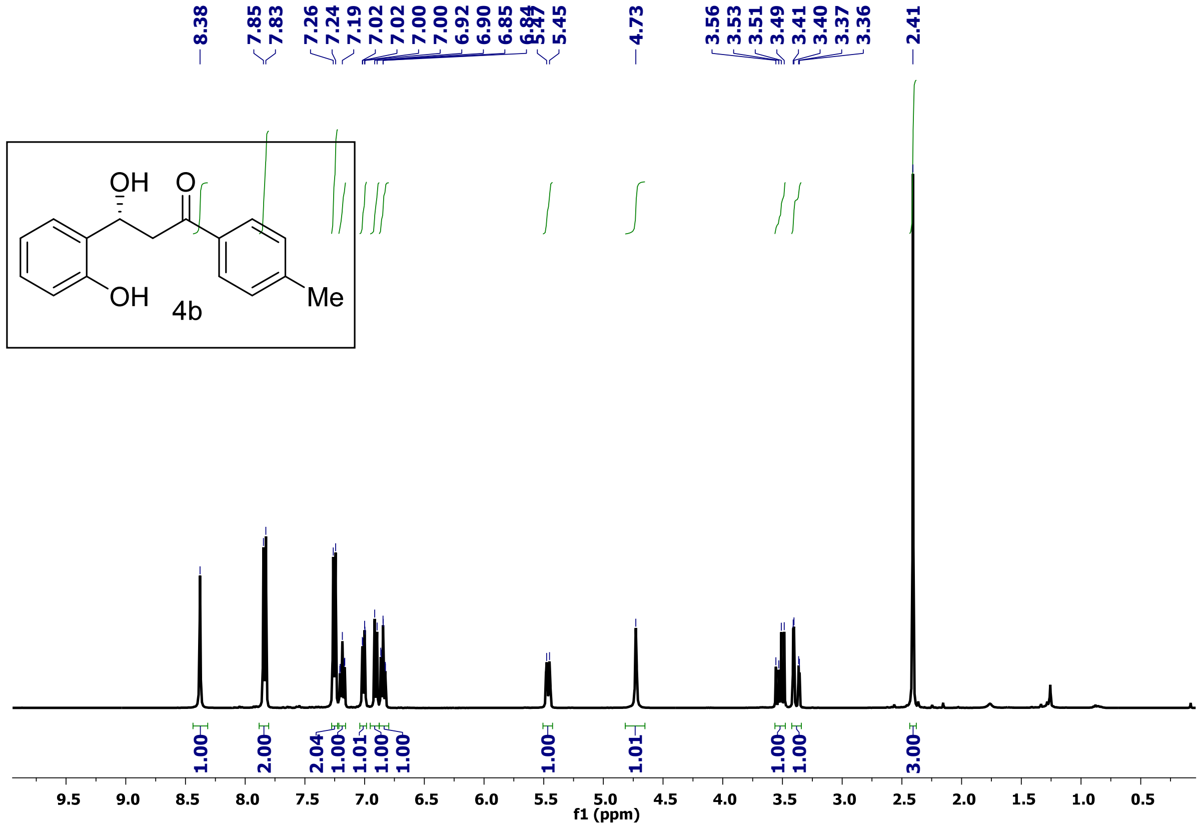
8.38
7.85
7.83
7.26
7.24
7.19
7.02
7.02
7.00
7.00
6.92
6.90
6.85
6.84
5.47
5.45
4.73
3.56
3.53
3.51
3.49
3.41
3.40
3.37
3.36
2.41
OH
O
OH
4b
Me
1.00
2.00
2.04
1.00
1.01
1.00
1.00
1.00
1.01
1.00
1.00
3.00
f1 (ppm)

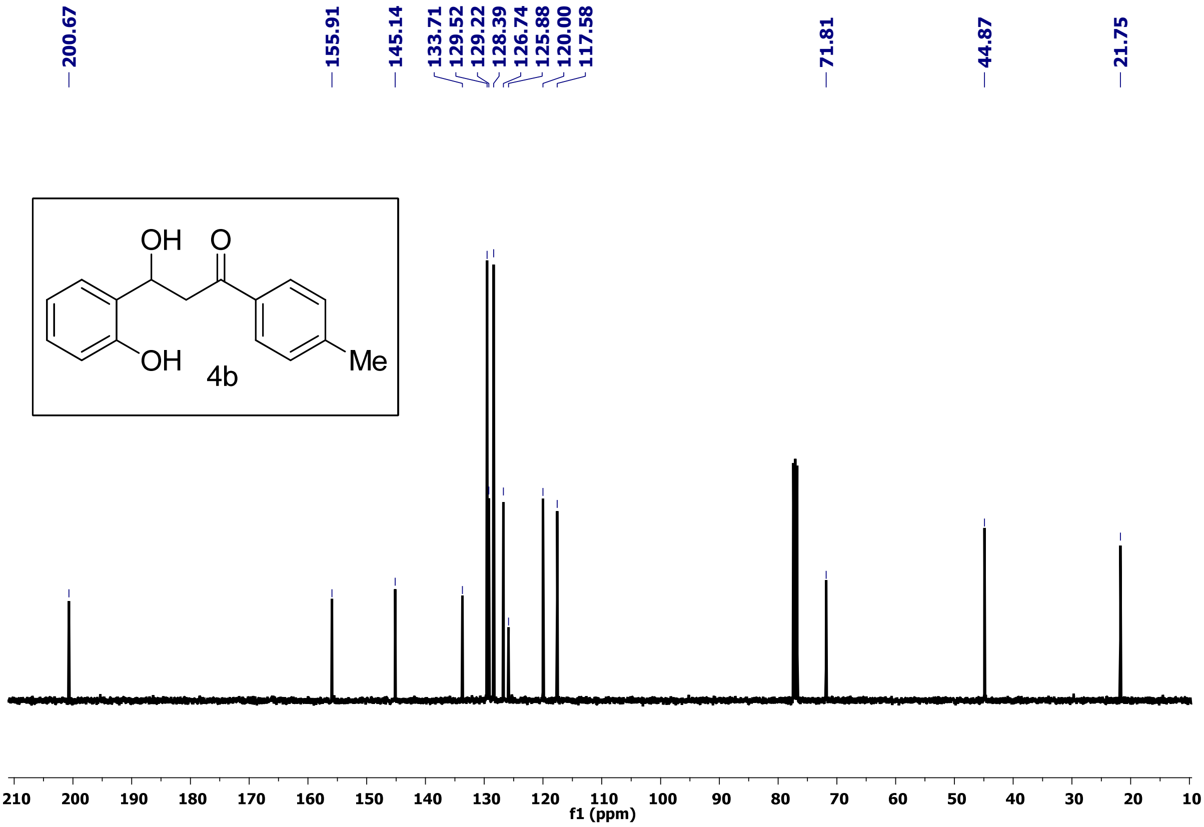
200.67
155.91
145.14
133.71
129.52
129.22
128.39
126.74
125.88
120.00
117.58
71.81
44.87
21.75
OH
O
OH
4b
Me
f1 (ppm)

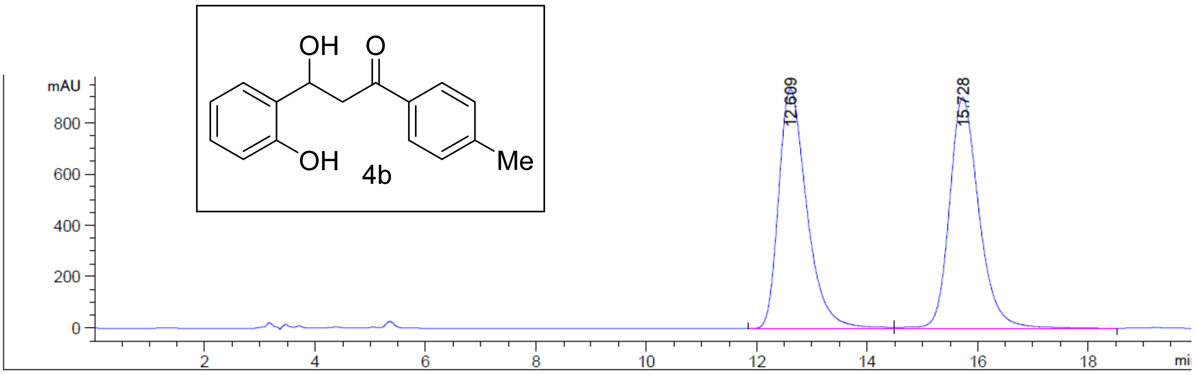

Signal 1: DAD1 A, Sig=254,4 Ref=360,100

Peak #	RetTime [min]	Type	Width [min]	Area [mAU*s]	Height [mAU]	Area %
1	12.609	BV	0.5418	3.30884e4	932.96667	49.7142
2	15.728	VV	0.5662	3.34688e4	899.32349	50.2858

Totals : 6.65572e4 1832.29016

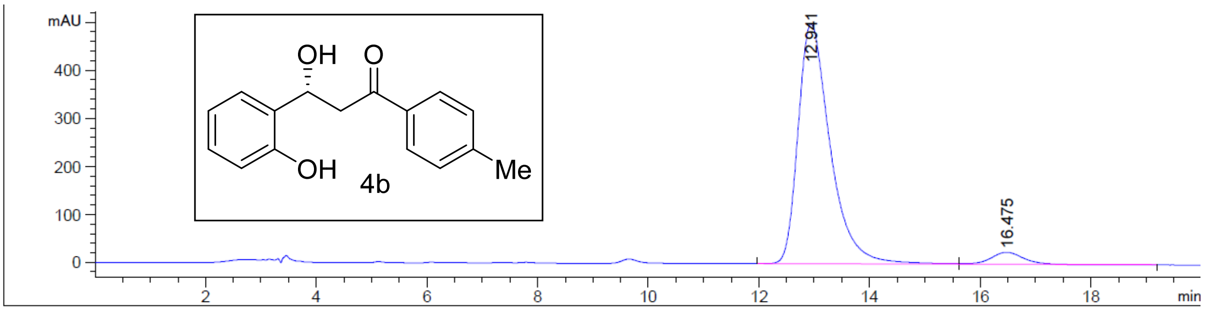

Signal 1: DAD1 A, Sig=254,4 Ref=360,100

Peak #	RetTime [min]	Type	Width [min]	Area [mAU*s]	Height [mAU]	Area %
1	12.941	VV	0.6134	2.01967e4	499.86285	94.9571
2	16.475	VV	0.6335	1072.58093	25.35507	5.0429

Totals : 2.12693e4 525.21792

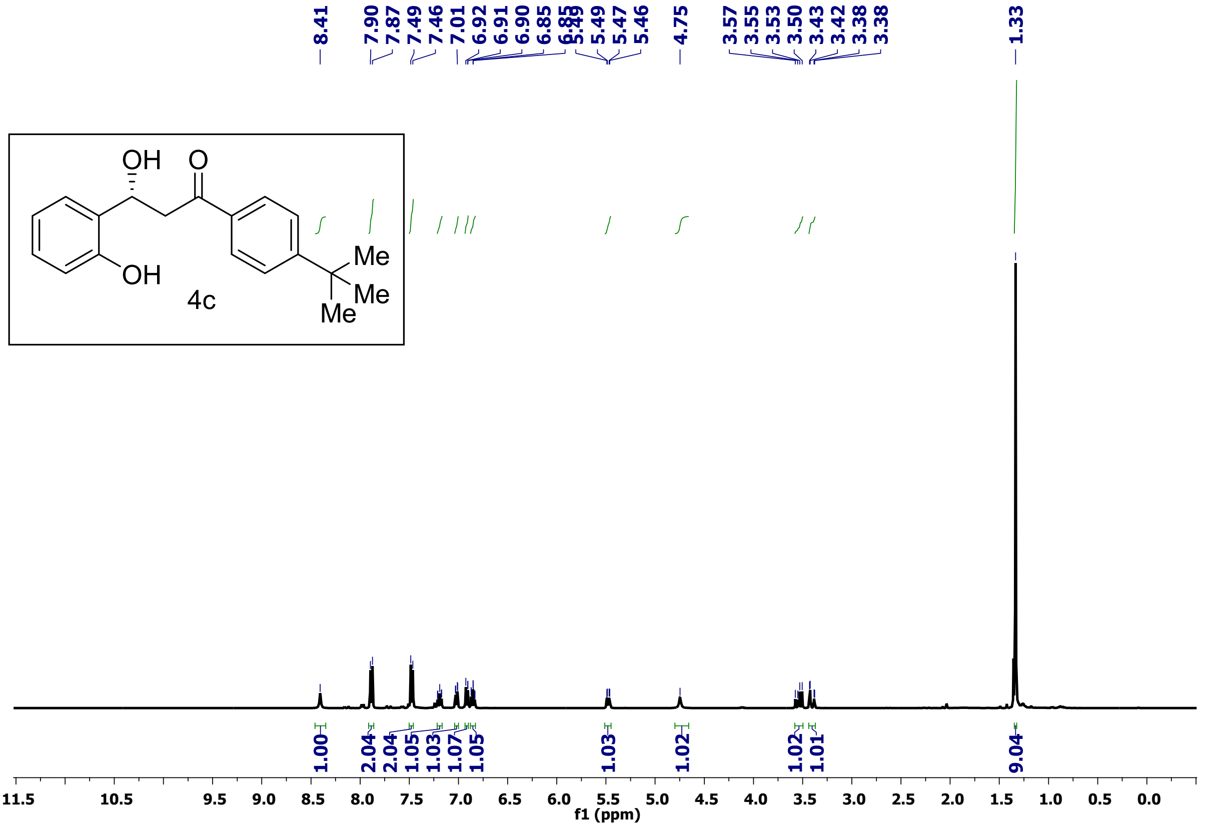
8.41
7.90
7.87
7.49
7.46
7.01
6.92
6.91
6.90
6.85
6.85
5.49
5.49
5.47
5.46
4.75
3.57
3.55
3.53
3.50
3.43
3.42
3.38
3.38
1.33
OH
O
OH
4c
Me
Me
Me
1.00
2.04
2.04
1.05
1.03
1.07
1.05
1.03
1.02
1.02
1.01
9.04
f1 (ppm)

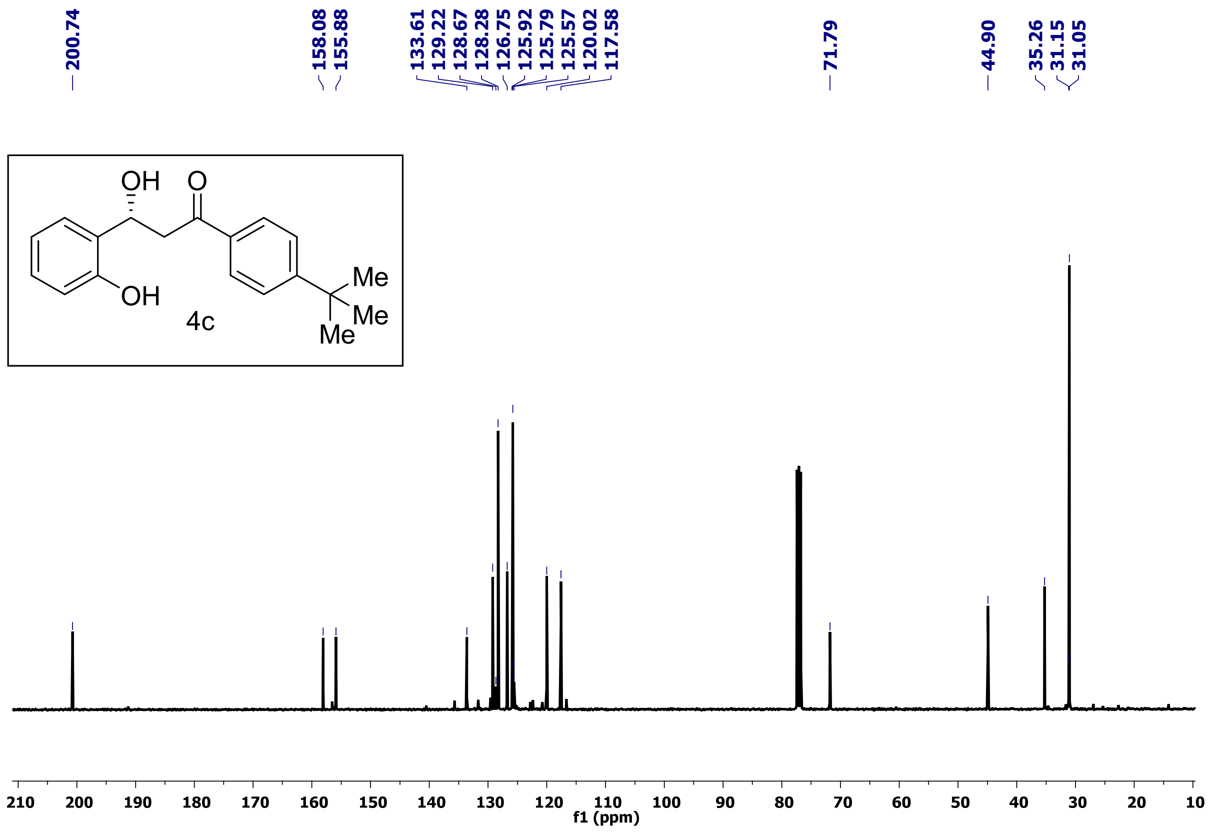
200.74
158.08
155.88
133.61
129.22
128.67
128.28
126.75
125.92
125.79
125.57
120.02
117.58
71.79
44.90
35.26
31.15
31.05
OH
O
OH
4c
Me
Me
Me
f1 (ppm)

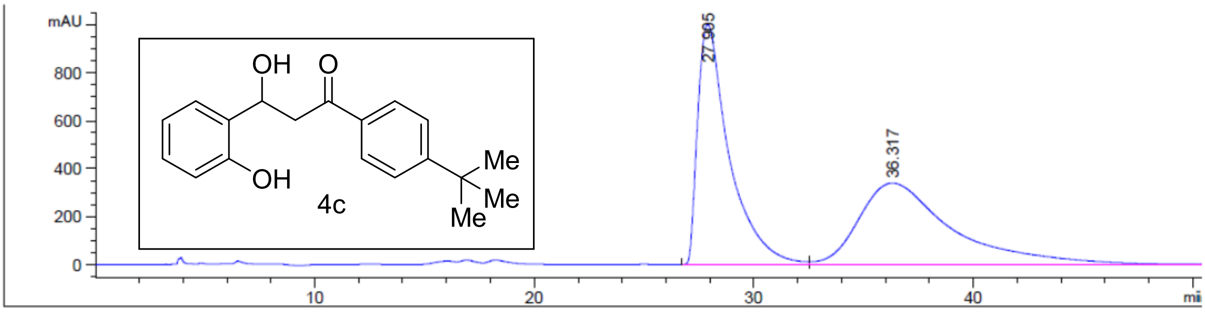

Signal 1: DAD1 A, Sig=254,4 Ref=360,100

Peak #	RetTime [min]	Type	Width [min]	Area [mAU*s]	Height [mAU]	Area %
1	27.905	BV	1.4801	9.99995e4	1000.47137	50.0970
2	36.317	VBA	4.2597	9.96123e4	339.24200	49.9030
Totals :				1.99612e5	1339.71338	

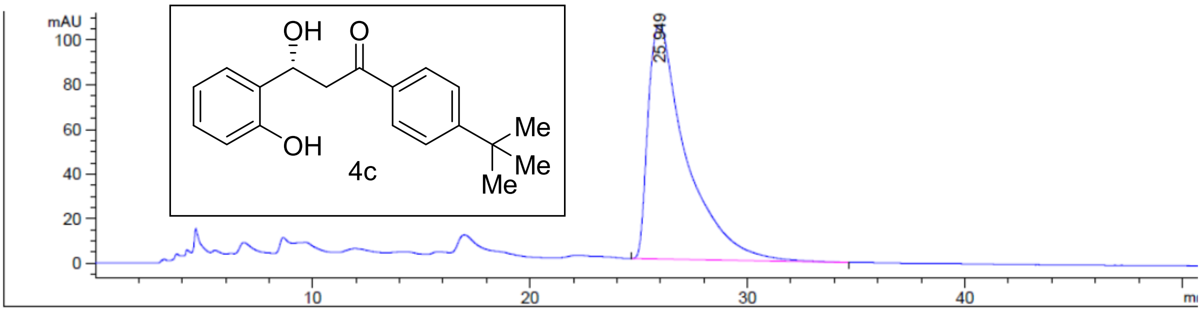

Signal 1: DAD1 A, Sig=254,4 Ref=360,100

Peak #	RetTime [min]	Type	Width [min]	Area [mAU*s]	Height [mAU]	Area %
1	25.949	BB	1.7646	1.28199e4	104.99127	100.0000
Totals :				1.28199e4	104.99127	

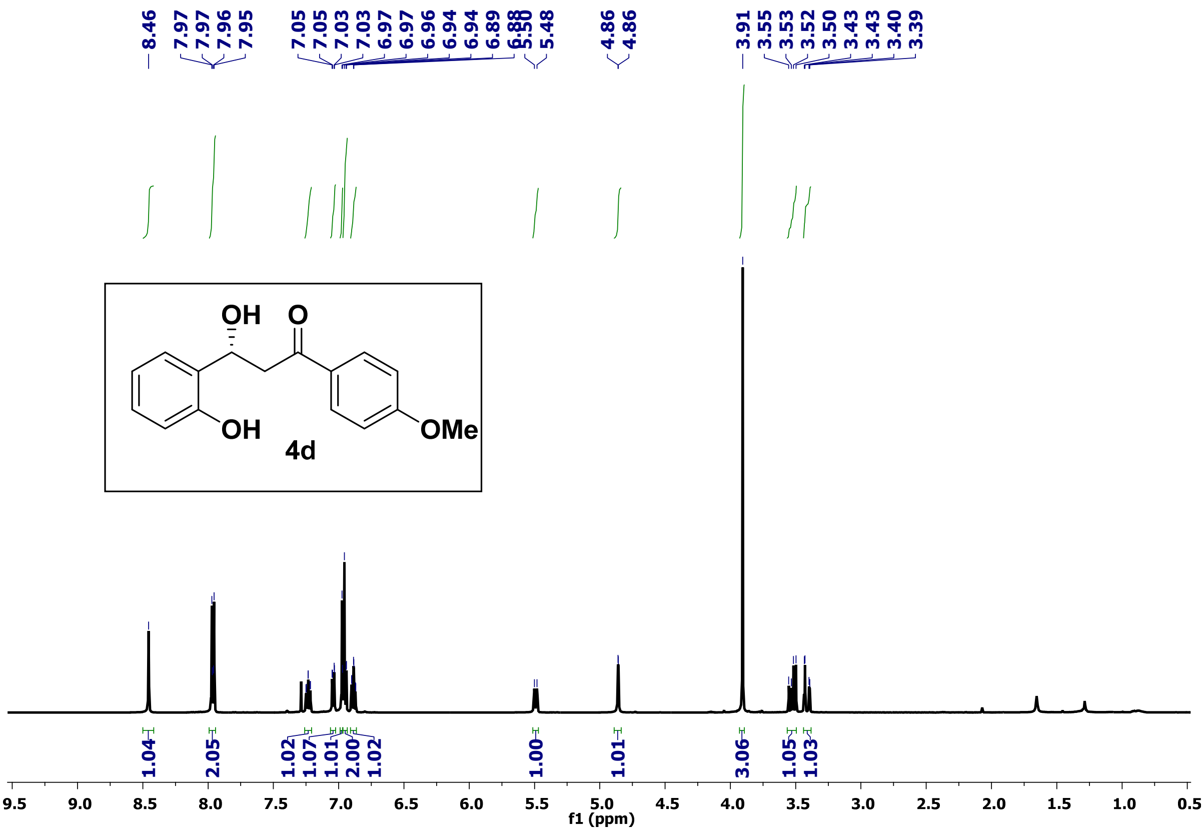
8.46 7.97 7.97 7.96 7.95 7.05 7.05 7.03 7.03 6.97 6.97 6.96 6.94 6.94 6.89 6.88 5.50 5.48 4.86 4.86 3.91 3.55 3.53 3.52 3.50 3.43 3.43 3.40 3.39
OH O
OH
OMe
4d
1.04 2.05 1.02 1.07 1.01 2.00 1.02 1.00 1.01 3.06 1.05 1.03
9.5 9.0 8.5 8.0 7.5 7.0 6.5 6.0 5.5 5.0 4.5 4.0 3.5 3.0 2.5 2.0 1.5 1.0 0.5
f1 (ppm)

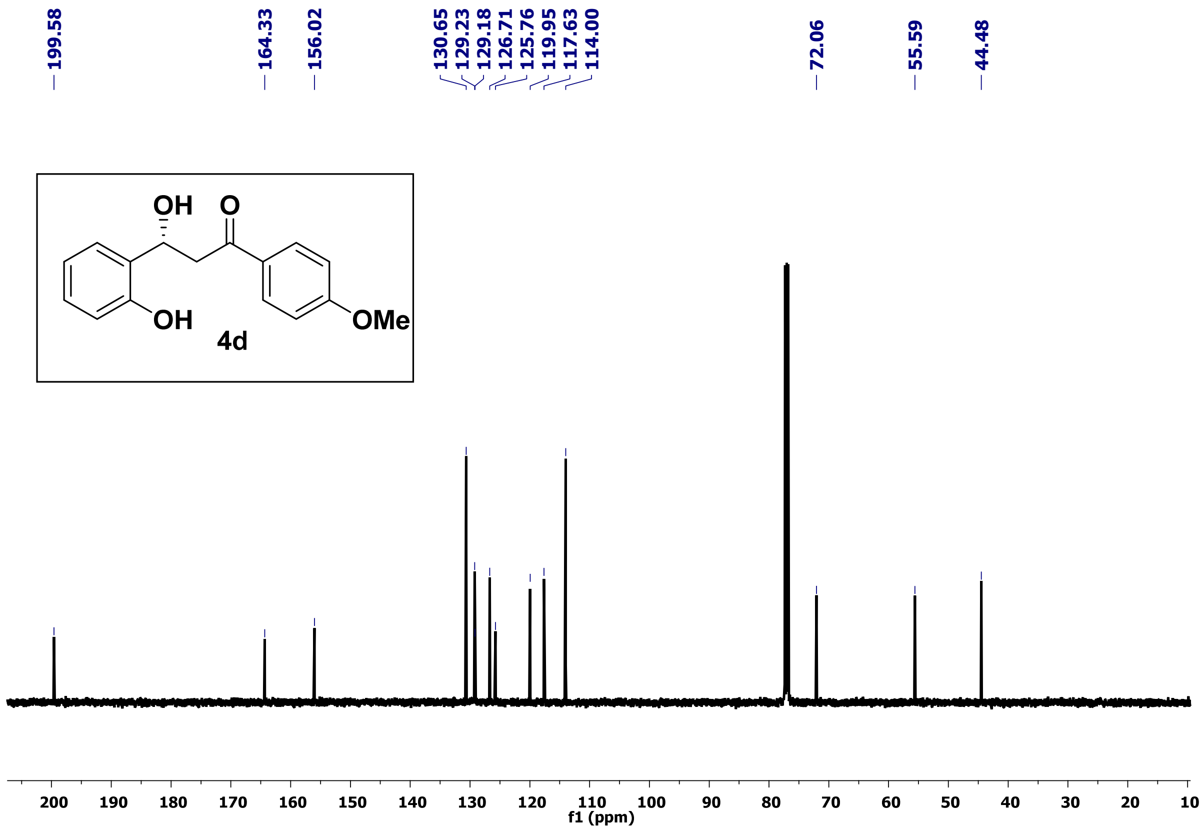
199.58 164.33 156.02 130.65 129.23 129.18 126.71 125.76 119.95 117.63 114.00 72.06 55.59 44.48
OH O
OH
OMe
4d
200 190 180 170 160 150 140 130 120 110 100 90 80 70 60 50 40 30 20 10
f1 (ppm)

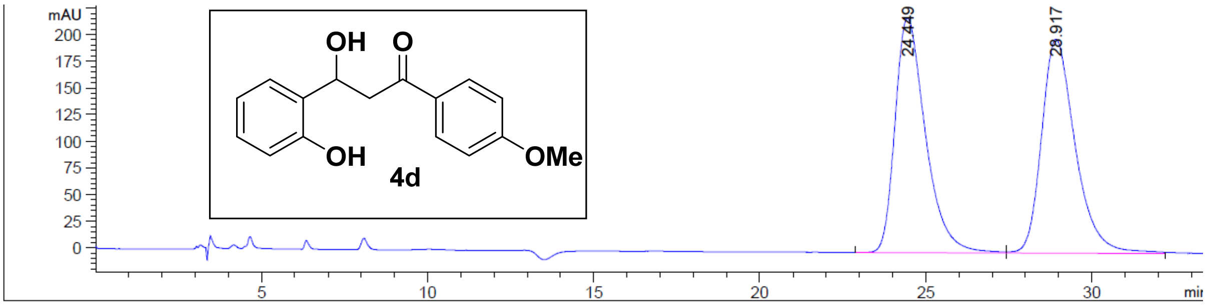

Signal 1: DAD1 A, Sig=254,4 Ref=360,100

Peak #	RetTime [min]	Type	Width [min]	Area [mAU*s]	Height [mAU]	Area %
1	24.449	VV	0.9662	1.40336e4	219.98921	49.8004
2	28.917	VV	1.0647	1.41461e4	200.59843	50.1996

Totals : 2.81796e4 420.58765

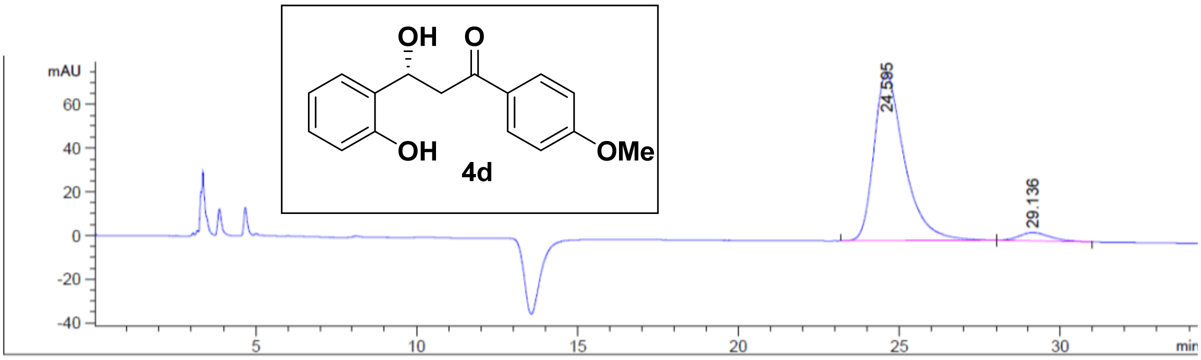

Signal 1: DAD1 A, Sig=254,4 Ref=360,100

Peak #	RetTime [min]	Type	Width [min]	Area [mAU*s]	Height [mAU]	Area %
1	24.595	BB	0.9901	5016.47412	76.19472	95.1934
2	29.136	BV	0.8405	253.29939	3.74803	4.8066

Totals : 5269.77351 79.94274

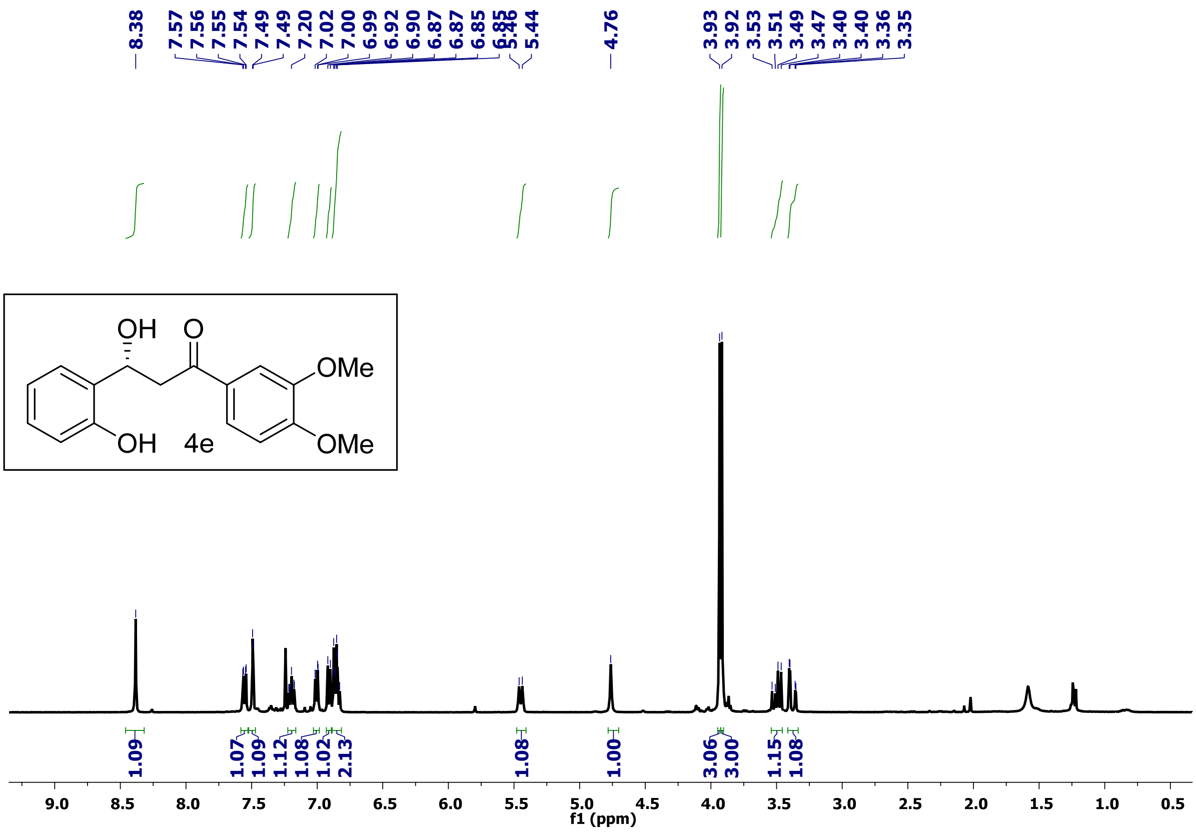
8.38
7.57
7.56
7.55
7.54
7.49
7.49
7.20
7.02
7.00
6.99
6.92
6.90
6.87
6.85
6.85
5.46
5.44
4.76
3.93
3.92
3.53
3.51
3.49
3.47
3.40
3.36
3.35
OH
O
OMe
OH
4e
OMe
1.09
1.07
1.09
1.12
1.08
1.02
2.13
1.08
1.00
3.06
3.00
1.15
1.08
9.0
8.5
8.0
7.5
7.0
6.5
6.0
5.5
5.0
4.5
4.0
3.5
3.0
2.5
2.0
1.5
1.0
0.5
f1 (ppm)

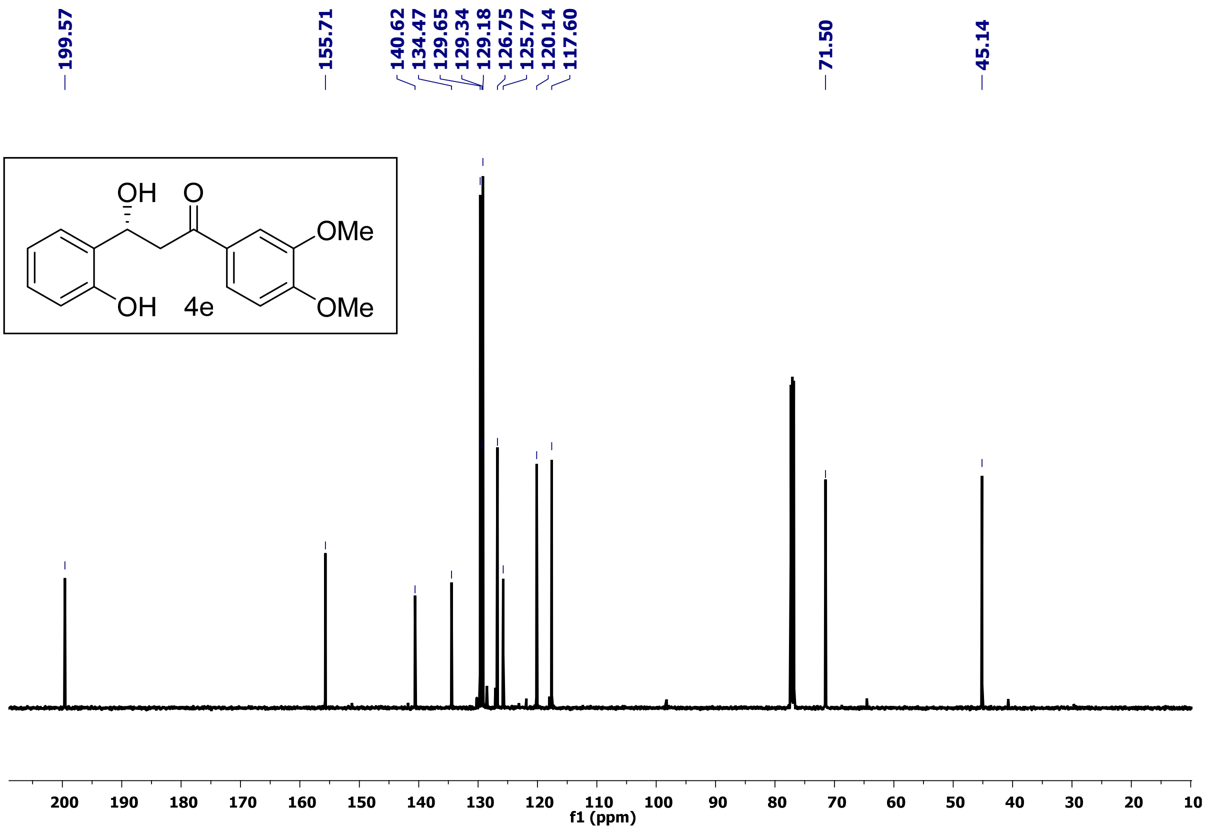
199.57
155.71
140.62
134.47
129.65
129.34
129.18
126.75
125.77
120.14
117.60
71.50
45.14
OH
O
OMe
OH
4e
OMe
200
190
180
170
160
150
140
130
120
110
100
90
80
70
60
50
40
30
20
10
f1 (ppm)

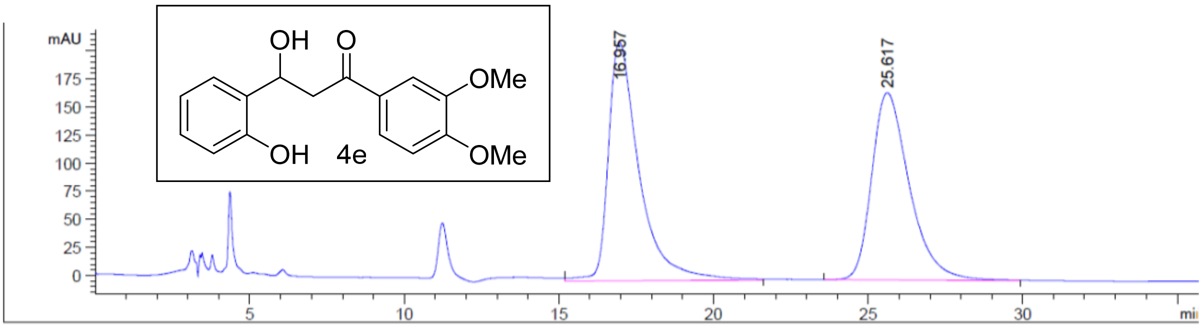

Signal 1: DAD1 A, Sig=254,4 Ref=360,100

Peak #	RetTime [min]	Type	Width [min]	Area [mAU*s]	Height [mAU]	Area %
1	16.957	VV	1.0281	1.48293e4	212.50467	51.2242
2	25.617	VV	1.2933	1.41205e4	166.52133	48.7758

Totals : 2.89499e4 379.02600

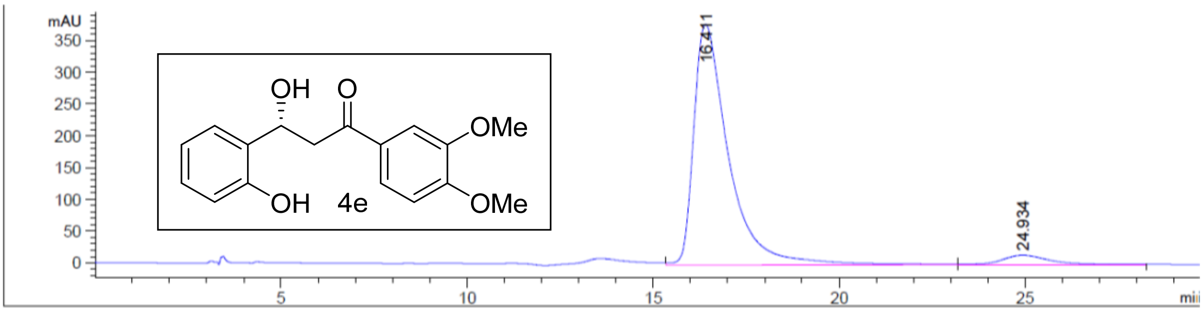

Signal 1: DAD1 A, Sig=254,4 Ref=360,100

Peak #	RetTime [min]	Type	Width [min]	Area [mAU*s]	Height [mAU]	Area %
1	16.411	VV	0.9753	2.49376e4	379.15503	94.7825
2	24.934	VV	1.3415	1372.74792	15.08690	5.2175

Totals : 2.63103e4 394.24193

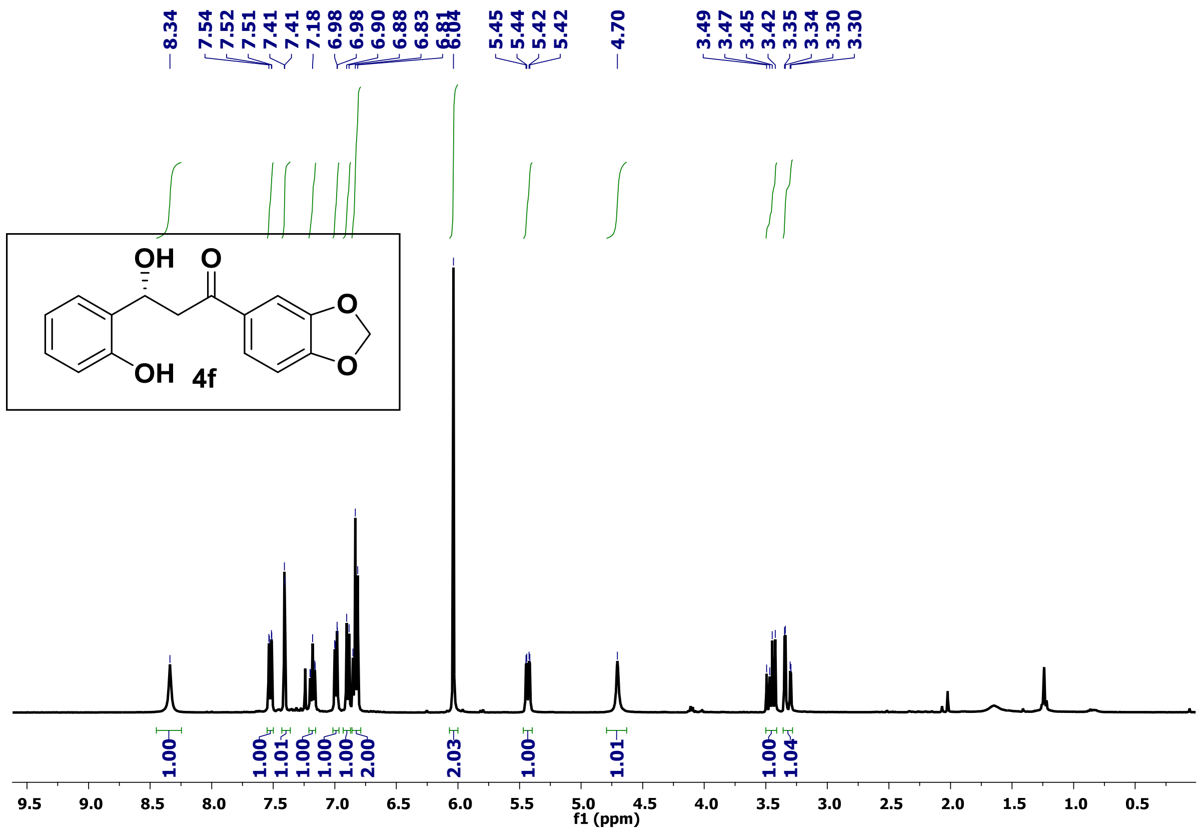
8.34
7.54
7.52
7.51
7.41
7.41
7.18
6.98
6.98
6.90
6.88
6.83
6.81
6.04
5.45
5.44
5.42
5.42
4.70
3.49
3.47
3.45
3.42
3.35
3.34
3.30
3.30
OH
O
O
O
OH
4f
1.00
1.00
1.01
1.00
1.00
1.00
2.00
2.03
1.00
1.01
1.00
1.04
f1 (ppm)

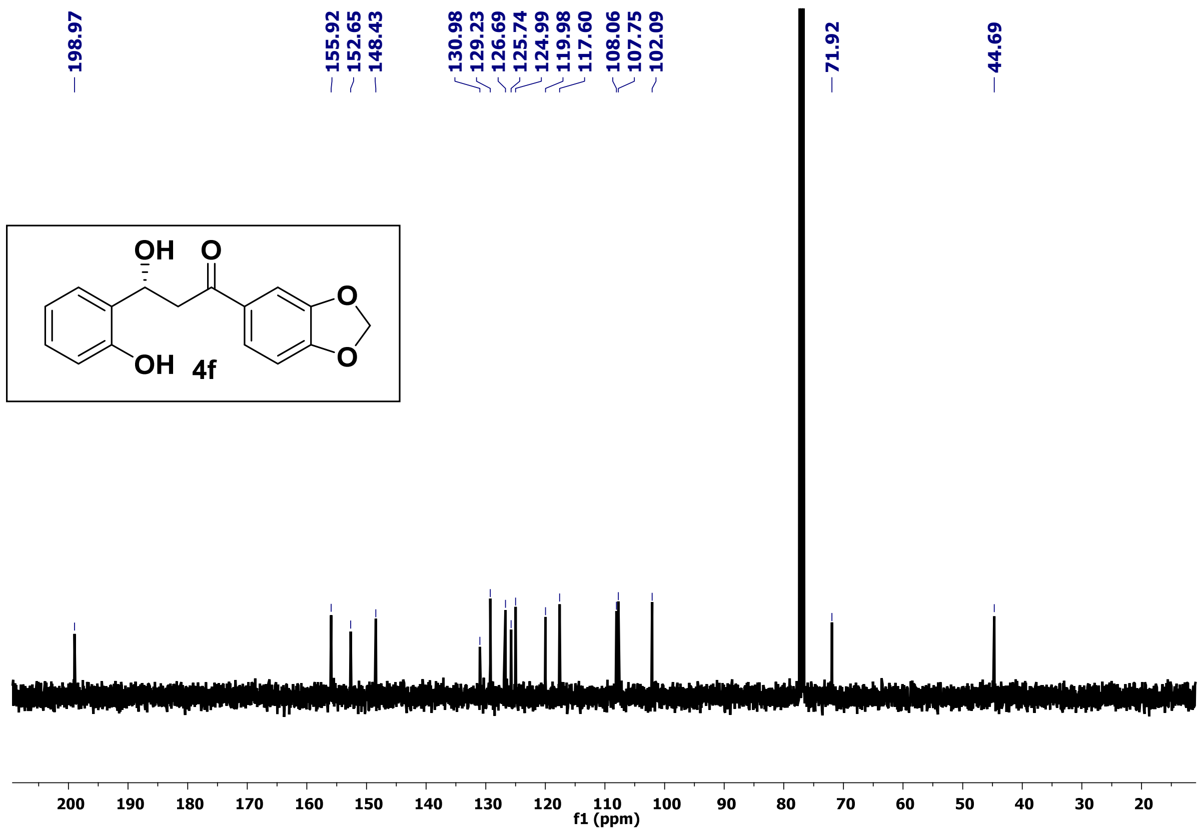
198.97
155.92
152.65
148.43
130.98
129.23
126.69
125.74
124.99
119.98
117.60
108.06
107.75
102.09
71.92
44.69
OH
O
O
O
OH
4f
f1 (ppm)

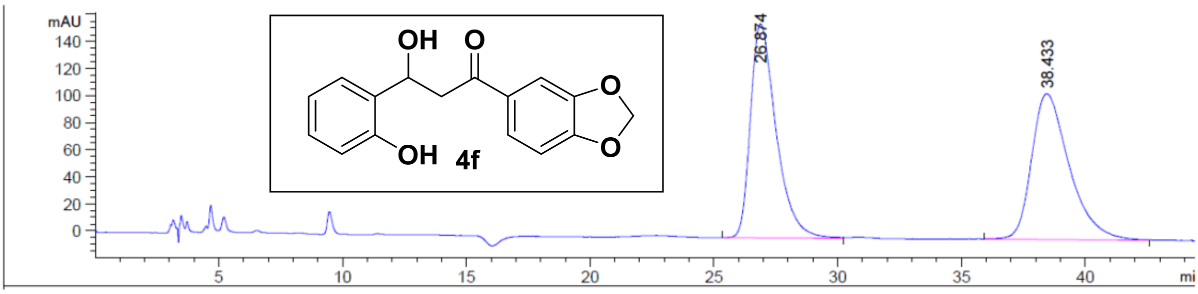

Signal 1: DAD1 A, Sig=254,4 Ref=360,100

Peak #	RetTime [min]	Type	Width [min]	Area [mAU*s]	Height [mAU]	Area %
1	26.874	VV	1.1007	1.14313e4	158.26610	50.0268
2	38.433	VV	1.5878	1.14190e4	107.98108	49.9732

Totals : 2.28503e4 266.24718

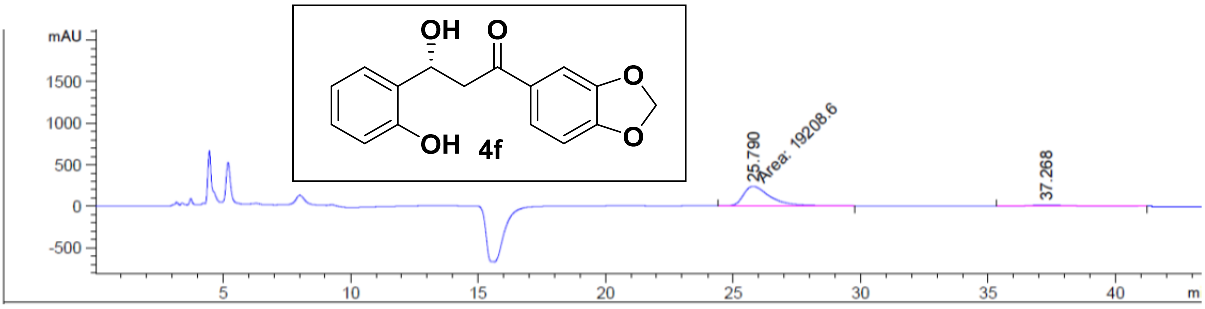

Signal 1: DAD1 A, Sig=254,4 Ref=360,100

Peak #	RetTime [min]	Type	Width [min]	Area [mAU*s]	Height [mAU]	Area %
1	25.790	MM	1.3210	1.92086e4	242.35229	91.9819
2	37.268	BV	1.4954	1674.41479	15.81250	8.0181

Totals : 2.08830e4 258.16479

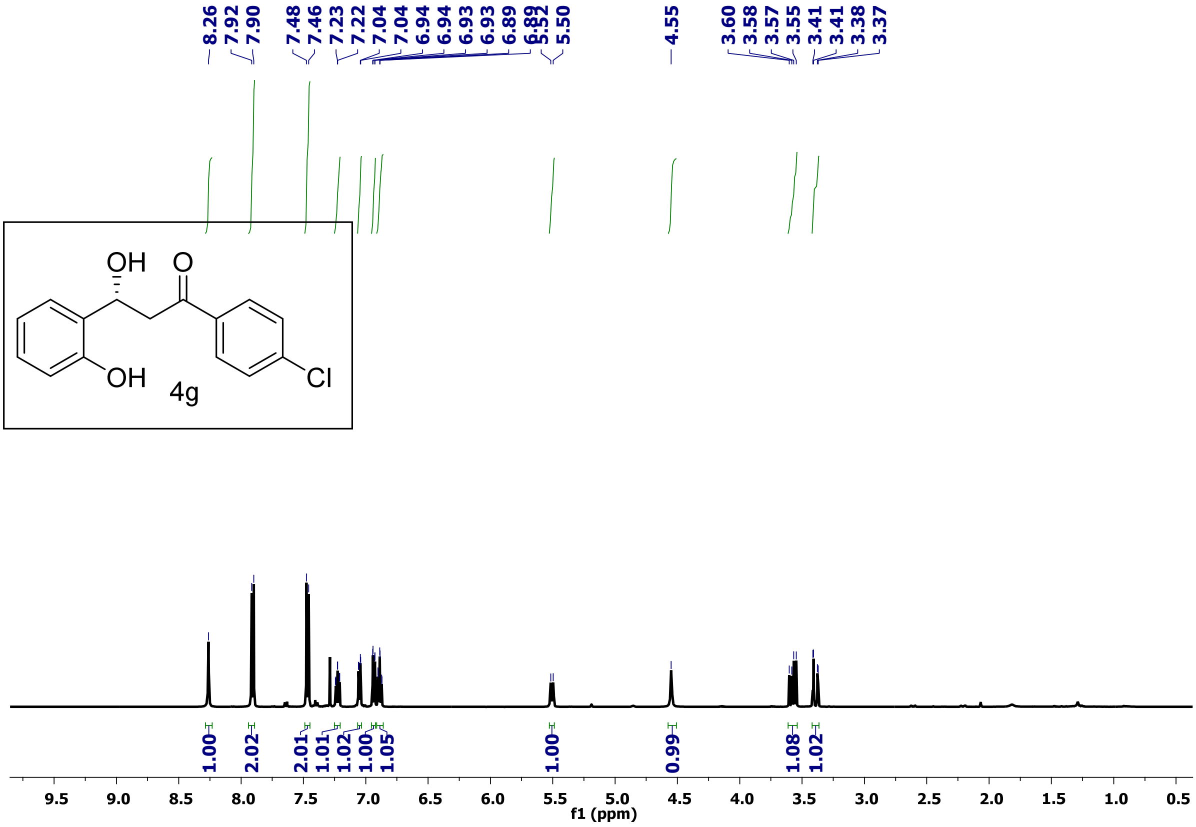
8.26
7.92
7.90
7.48
7.46
7.23
7.22
7.04
7.04
6.94
6.94
6.93
6.93
6.89
6.89
5.52
5.50
4.55
3.60
3.58
3.57
3.55
3.41
3.41
3.38
3.37
OH
O
OH
4g
Cl
1.00
2.02
2.01
1.01
1.02
1.00
1.05
1.00
0.99
1.08
1.02
f1 (ppm)

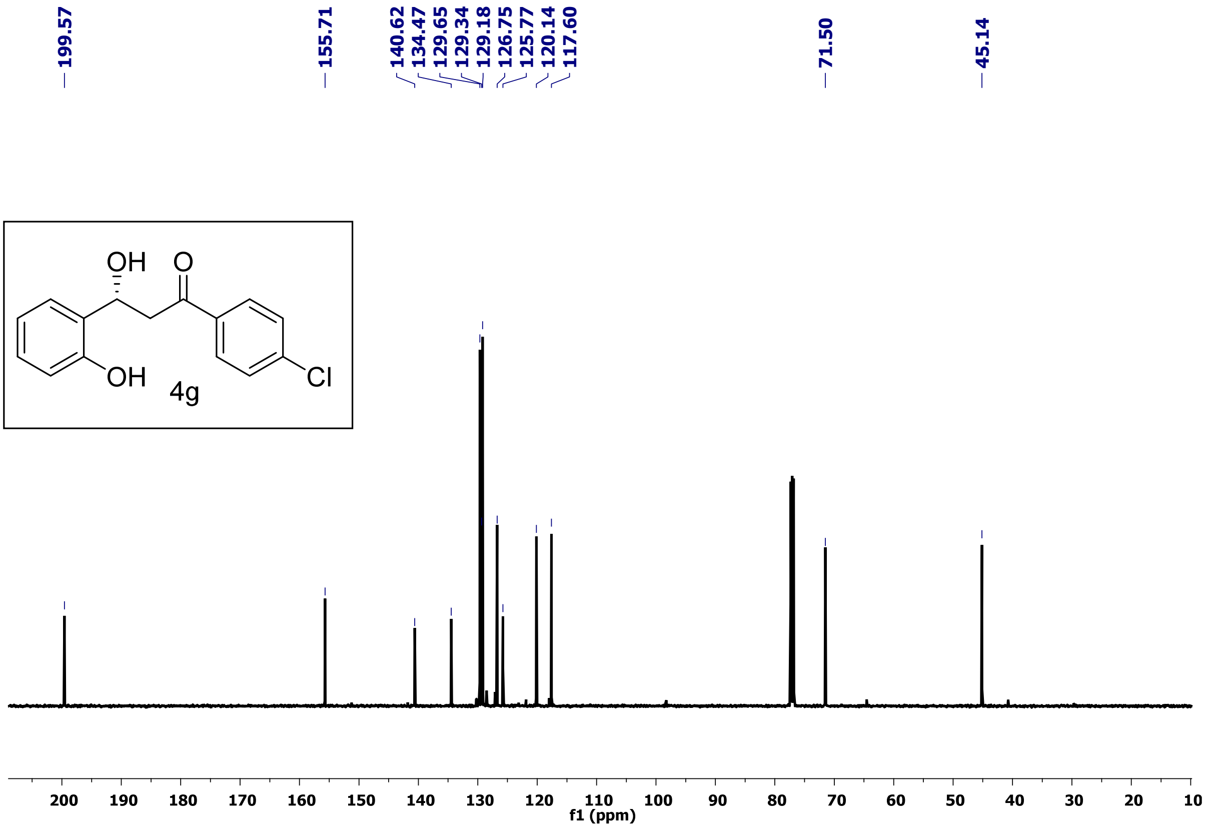
199.57
155.71
140.62
134.47
129.65
129.34
129.18
126.75
125.77
120.14
117.60
71.50
45.14
OH
O
OH
4g
Cl
f1 (ppm)

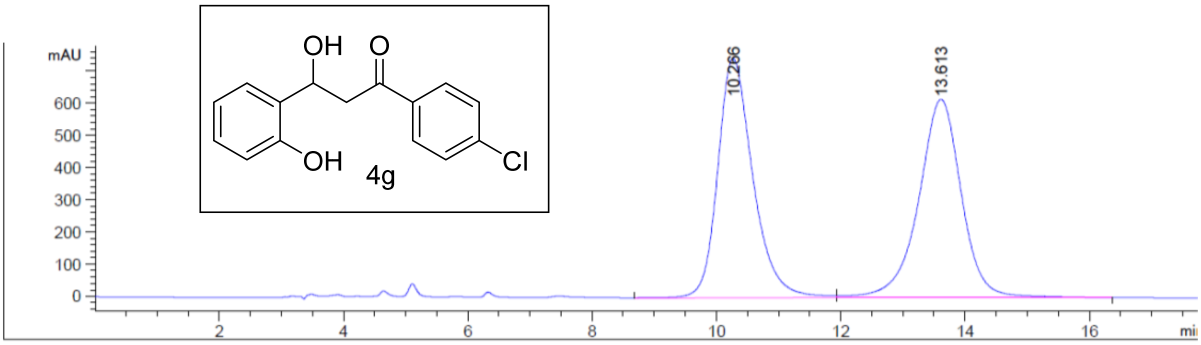

Signal 1: DAD1 A, Sig=254,4 Ref=360,100

Peak #	RetTime [min]	Type	Width [min]	Area [mAU*s]	Height [mAU]	Area %
1	10.266	BV	0.5960	2.93157e4	743.55902	49.7819
2	13.613	VB	0.7223	2.95725e4	615.76117	50.2181

Totals : 5.88882e4 1359.32019

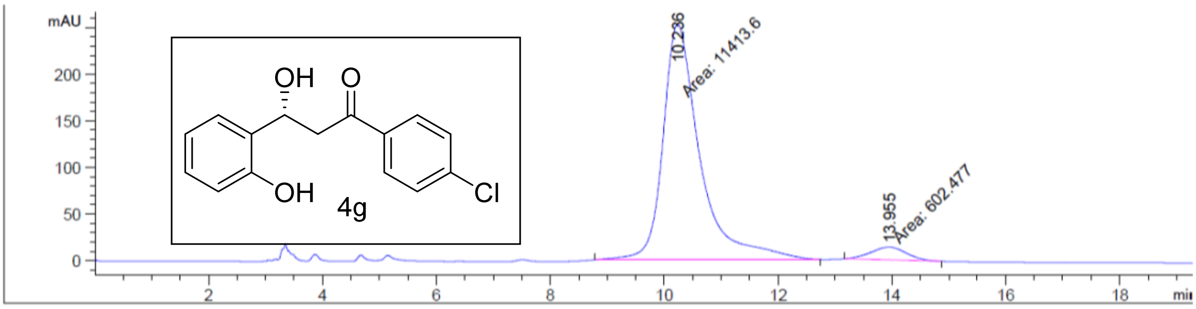

Signal 1: DAD1 A, Sig=254,4 Ref=360,100

Peak #	RetTime [min]	Type	Width [min]	Area [mAU*s]	Height [mAU]	Area %
1	10.236	MM	0.7536	1.14136e4	252.43982	94.9861
2	13.955	MM	0.7321	602.47717	13.71593	5.0139

Totals : 1.20161e4 266.15575

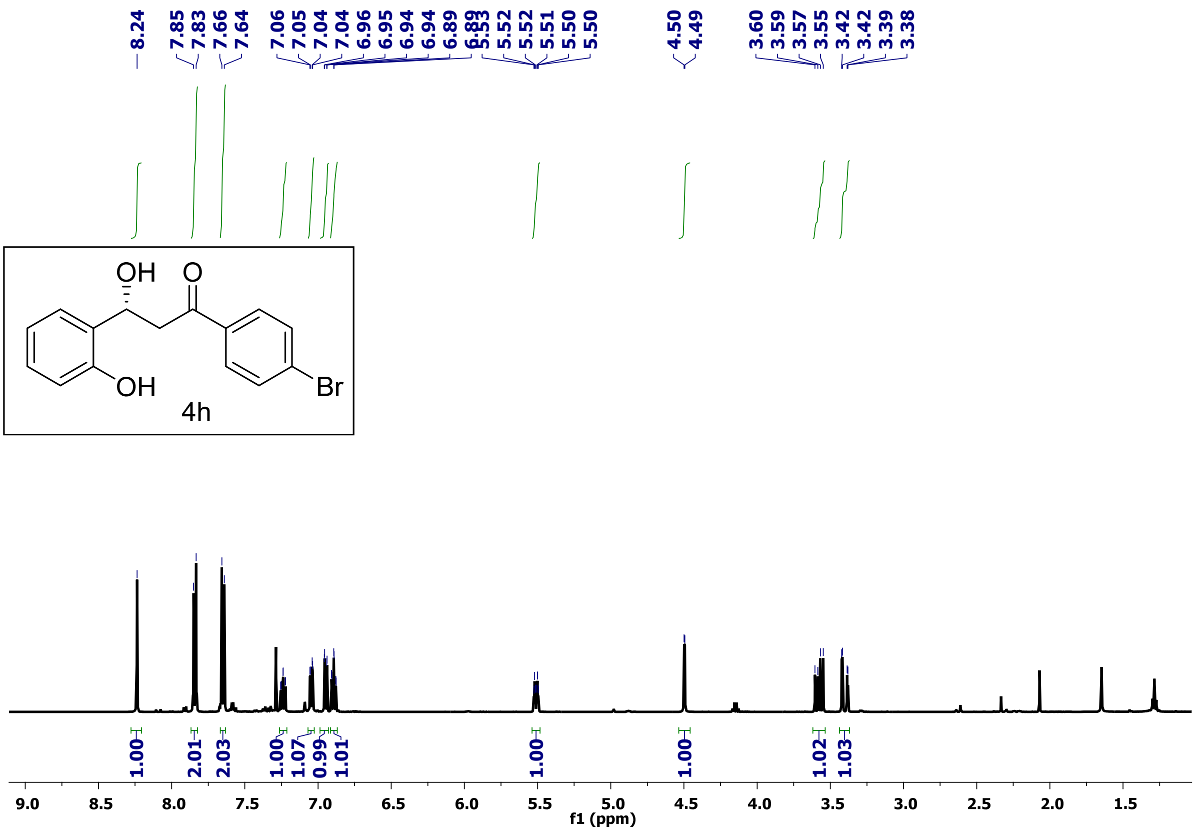
8.24
7.85
7.83
7.66
7.64
7.06
7.05
7.04
7.04
6.96
6.95
6.94
6.94
6.89
6.89
5.53
5.52
5.52
5.51
5.50
5.50
4.50
4.49
3.60
3.59
3.57
3.55
3.42
3.42
3.39
3.38
OH
O
OH
Br
4h
1.00
2.01
2.03
1.00
1.07
0.99
1.01
1.00
1.00
1.02
1.03
9.0 8.5 8.0 7.5 7.0 6.5 6.0 5.5 5.0 4.5 4.0 3.5 3.0 2.5 2.0 1.5
f1 (ppm)

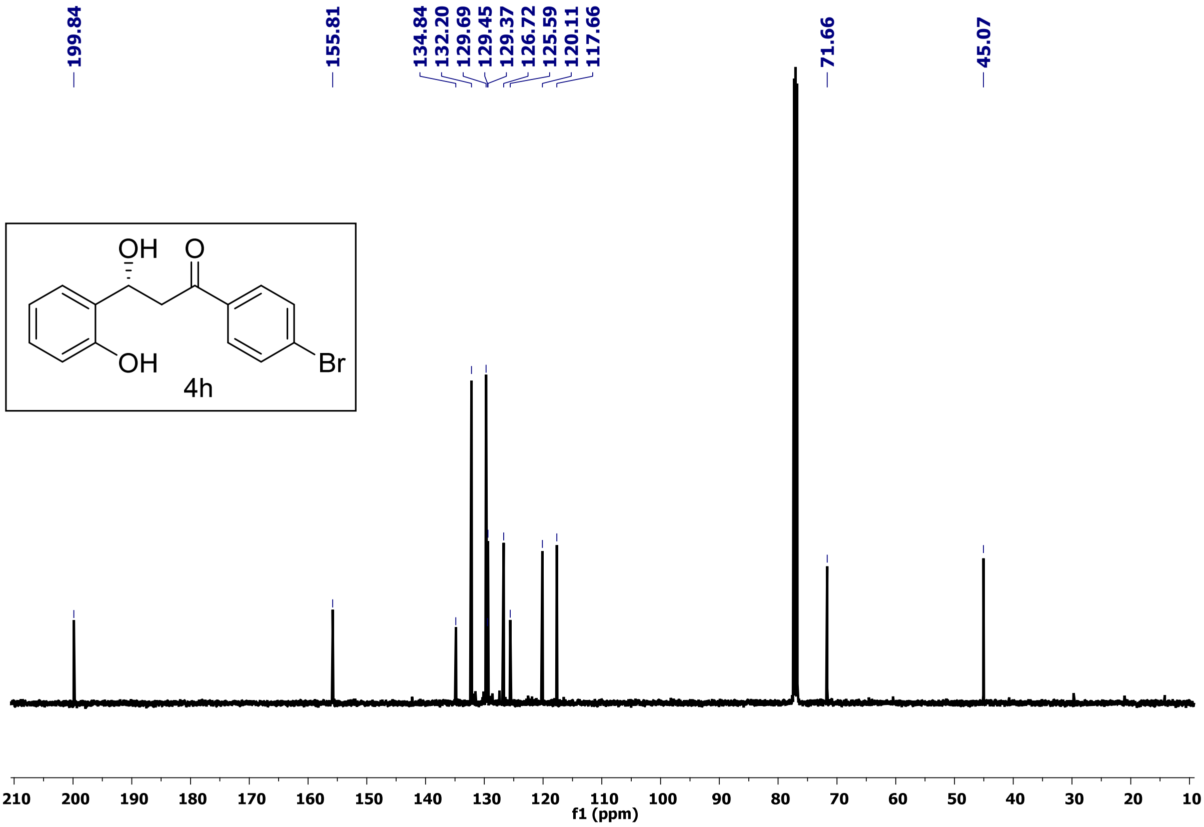
199.84
155.81
134.84
132.20
129.69
129.45
129.37
126.72
125.59
120.11
117.66
71.66
45.07
OH
O
OH
Br
4h
210 200 190 180 170 160 150 140 130 120 110 100 90 80 70 60 50 40 30 20 10
f1 (ppm)

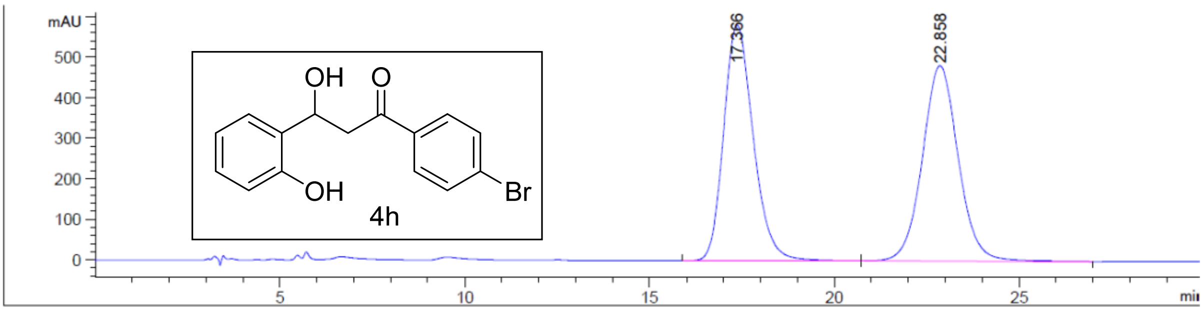

Signal 1: DAD1 A, Sig=254,4 Ref=360,100

Peak #	RetTime [min]	Type	Width [min]	Area [mAU*s]	Height [mAU]	Area %
1	17.366	VB	0.8587	3.24484e4	581.78070	49.9161
2	22.858	BV	1.0369	3.25574e4	481.46838	50.0839

Totals : 6.50058e4 1063.24908

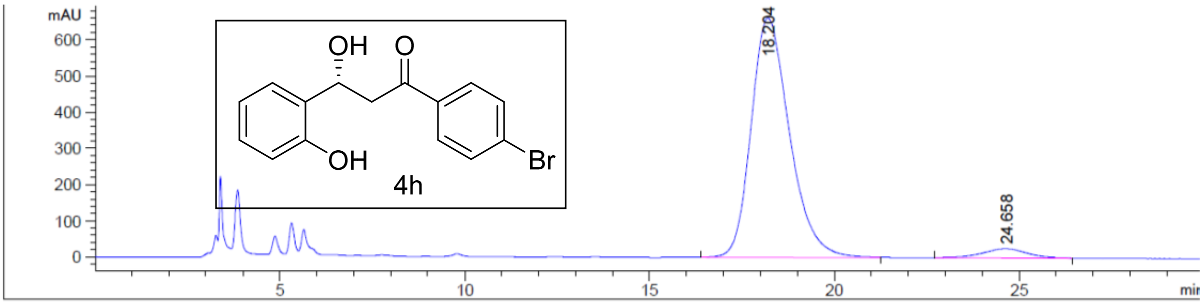

Signal 1: DAD1 A, Sig=254,4 Ref=360,100

Peak #	RetTime [min]	Type	Width [min]	Area [mAU*s]	Height [mAU]	Area %
1	18.204	VV	1.1123	4.78457e4	661.17096	95.7414
2	24.658	VV	1.0240	2128.18311	25.33521	4.2586

Totals : 4.99738e4 686.50617

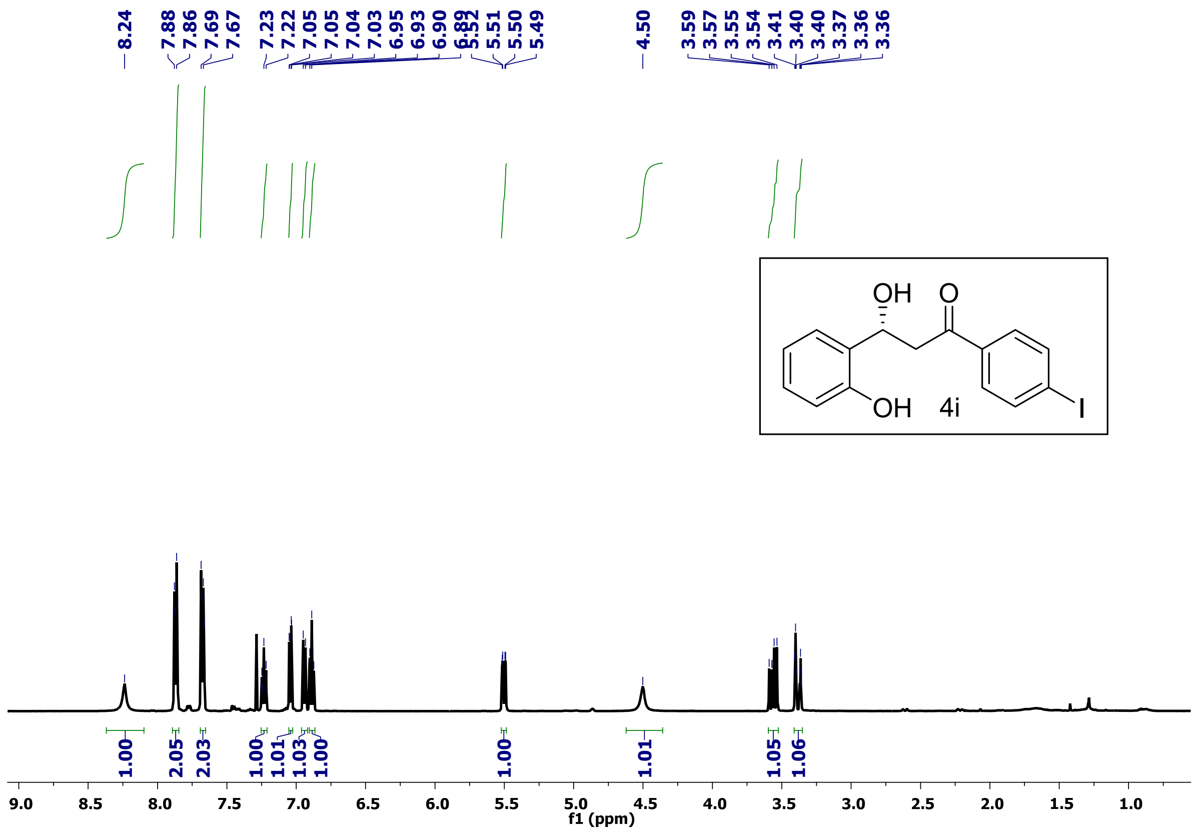
8.24
7.88
7.86
7.69
7.67
7.23
7.22
7.05
7.05
7.04
7.03
6.95
6.93
6.90
6.89
5.52
5.51
5.50
5.49
4.50
3.59
3.57
3.55
3.54
3.41
3.40
3.40
3.37
3.36
3.36
OH
O
OH
4i
I
1.00
2.05
2.03
1.00
1.01
1.03
1.00
1.00
1.01
1.05
1.06
9.0 8.5 8.0 7.5 7.0 6.5 6.0 5.5 5.0 4.5 4.0 3.5 3.0 2.5 2.0 1.5 1.0
f1 (ppm)

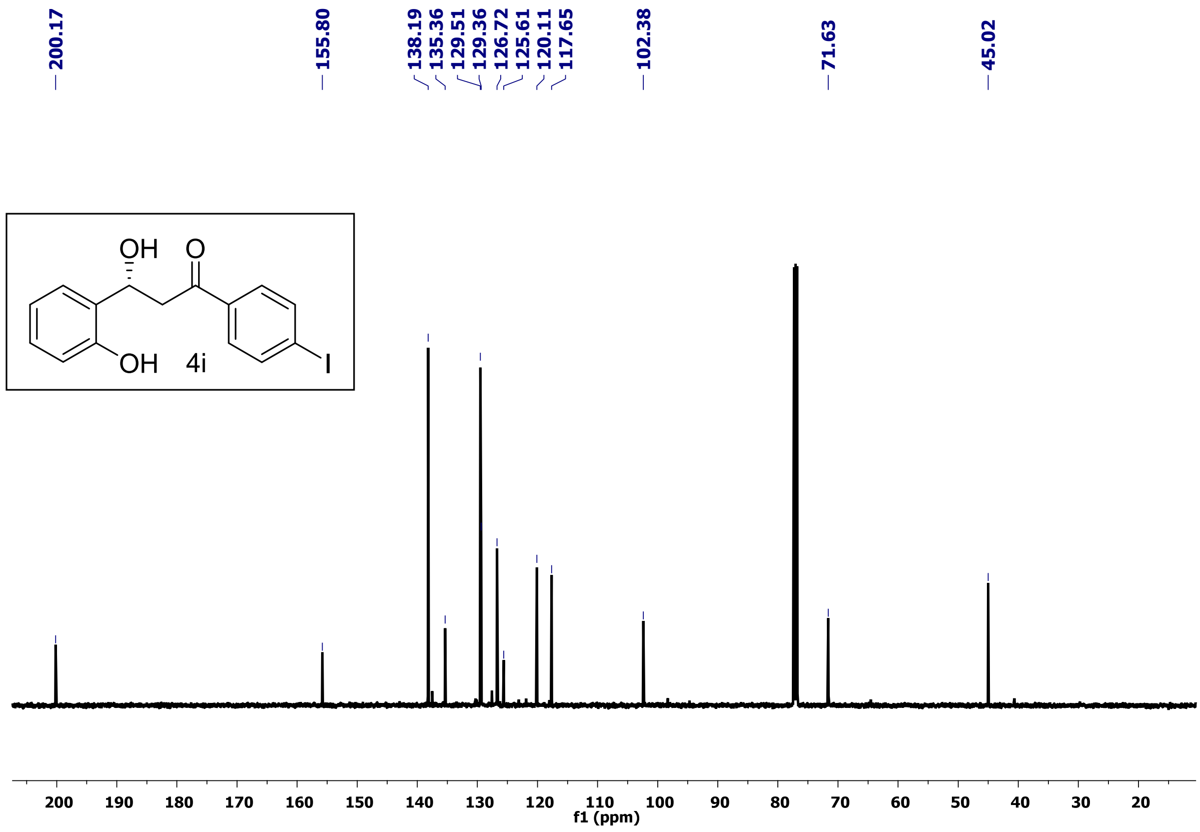
200.17
155.80
138.19
135.36
129.51
129.36
126.72
125.61
120.11
117.65
102.38
71.63
45.02
OH
O
OH
4i
I
200 190 180 170 160 150 140 130 120 110 100 90 80 70 60 50 40 30 20
f1 (ppm)

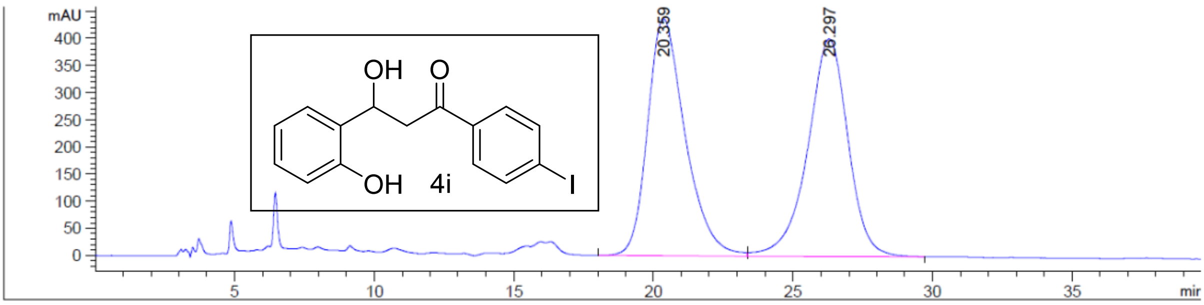

Signal 1: DAD1 A, Sig=254,4 Ref=360,100

Peak #	RetTime [min]	Type	Width [min]	Area [mAU*s]	Height [mAU]	Area %
1	20.359	BV	1.4285	4.09490e4	437.28455	50.3915
2	26.297	VV	1.5182	4.03127e4	401.23065	49.6085

Totals : 8.12617e4 838.51520

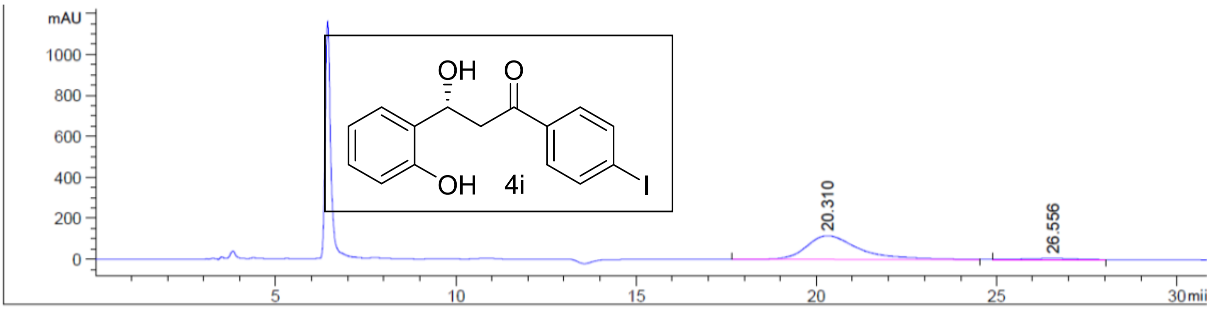

Signal 1: DAD1 A, Sig=254,4 Ref=360,100

Peak #	RetTime [min]	Type	Width [min]	Area [mAU*s]	Height [mAU]	Area %
1	20.310	VV	1.4656	1.18929e4	116.63773	94.7468
2	26.556	VV	1.2174	659.40265	6.40332	5.2532

Totals : 1.25523e4 123.04104

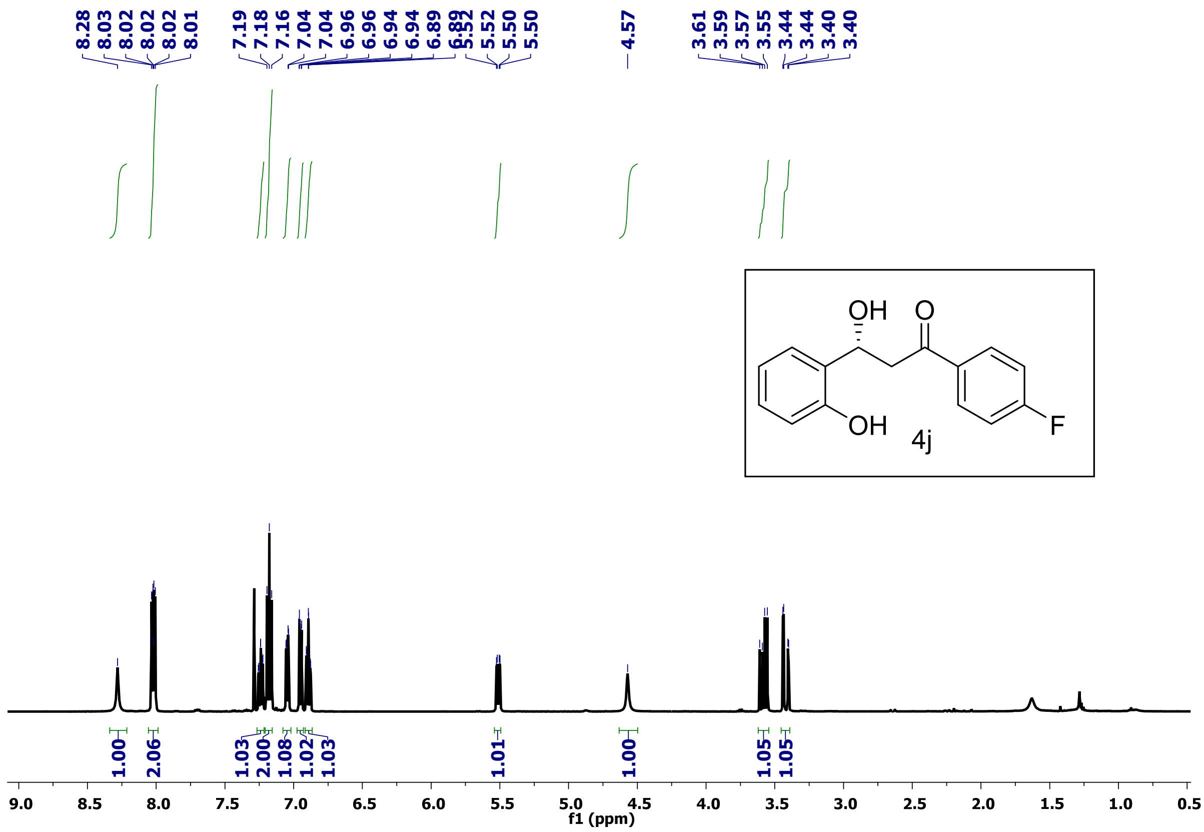
8.28
8.03
8.02
8.02
8.02
8.01
7.19
7.18
7.16
7.04
7.04
6.96
6.94
6.94
6.89
6.89
5.52
5.52
5.50
5.50
4.57
3.61
3.59
3.57
3.55
3.44
3.44
3.40
3.40
OH
O
OH
4j
F
1.00
2.06
1.03
2.00
1.08
1.02
1.03
1.01
1.00
1.05
1.05
f1 (ppm)

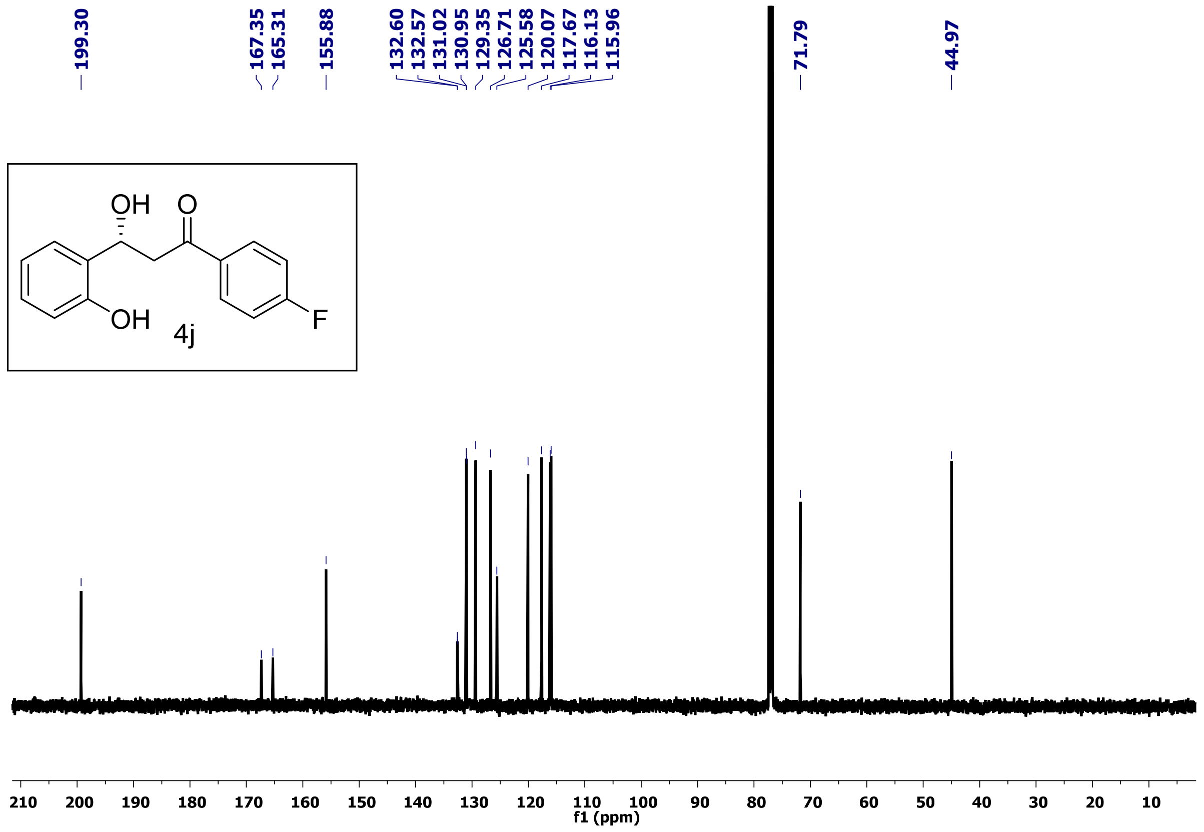
199.30
167.35
165.31
155.88
132.60
132.57
131.02
130.95
129.35
126.71
125.58
120.07
117.67
116.13
115.96
71.79
44.97
OH
O
OH
4j
F
f1 (ppm)

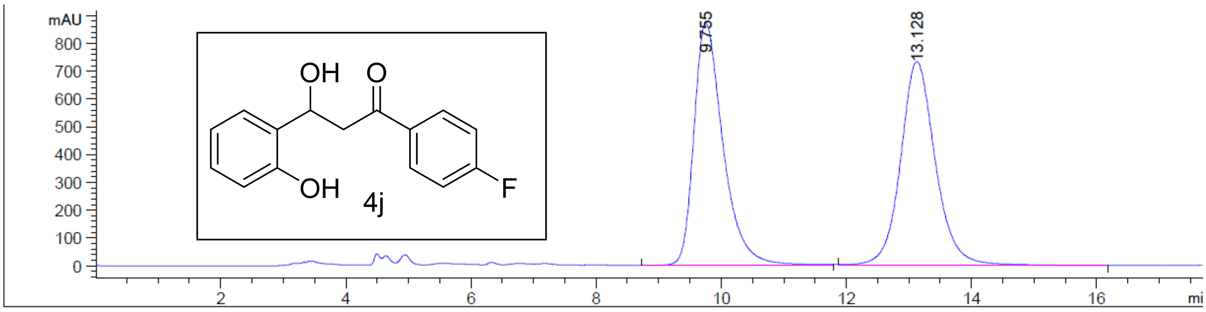

Signal 1: DAD1 A, Sig=254,4 Ref=360,100

Peak #	RetTime [min]	Type	Width [min]	Area [mAU*s]	Height [mAU]	Area %
1	9.755	BV	0.4924	2.84411e4	872.37018	49.8009
2	13.128	VB	0.5877	2.86685e4	734.00372	50.1991

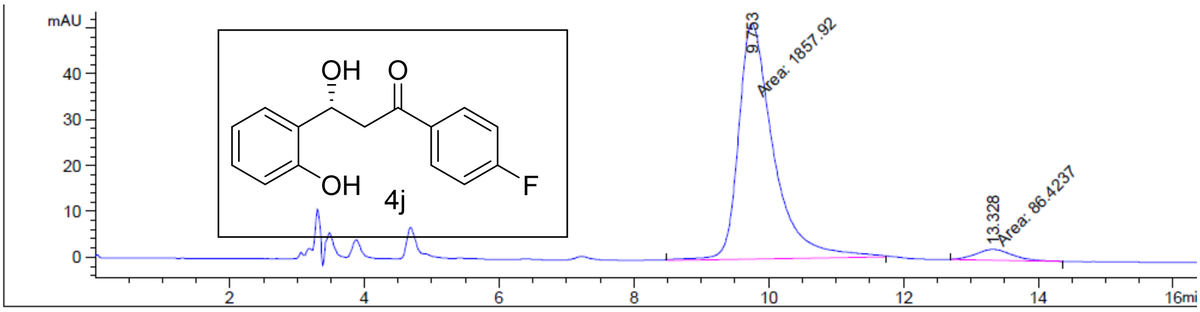

Signal 1: DAD1 A, Sig=254,4 Ref=360,100

Peak #	RetTime [min]	Type	Width [min]	Area [mAU*s]	Height [mAU]	Area %
1	9.753	MM	0.6031	1857.91577	51.34282	95.5551
2	13.328	MM	0.6136	86.42373	2.34760	4.4449

Totals : 1944.33950 53.69042

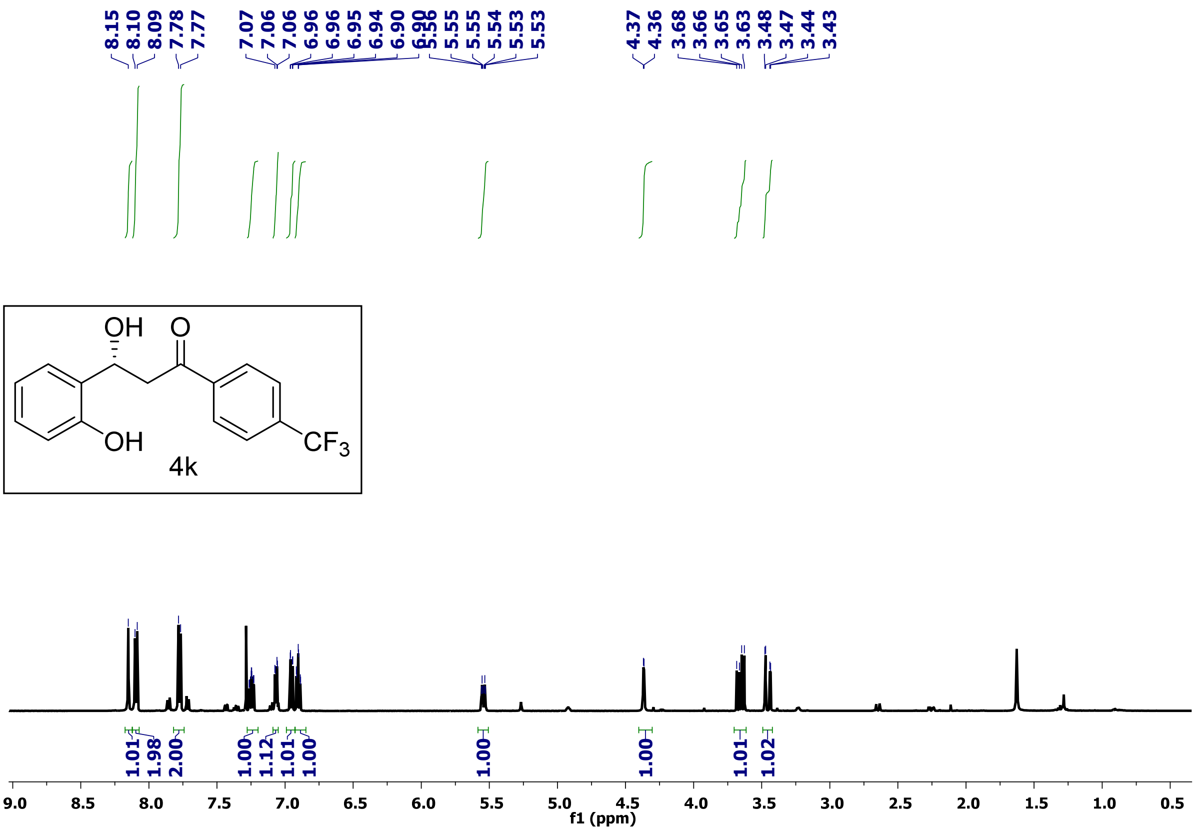
8.15 8.10 8.09 7.78 7.77 7.07 7.06 7.06 6.96 6.96 6.95 6.94 6.90 6.90 5.56 5.55 5.55 5.54 5.53 5.53 4.37 4.36 3.68 3.66 3.65 3.63 3.48 3.47 3.44 3.43
OH O
OH
CF3
4k
1.01 1.98 2.00 1.00 1.12 1.01 1.00 1.00 1.00 1.01 1.02
9.0 8.5 8.0 7.5 7.0 6.5 6.0 5.5 5.0 4.5 4.0 3.5 3.0 2.5 2.0 1.5 1.0 0.5
f1 (ppm)

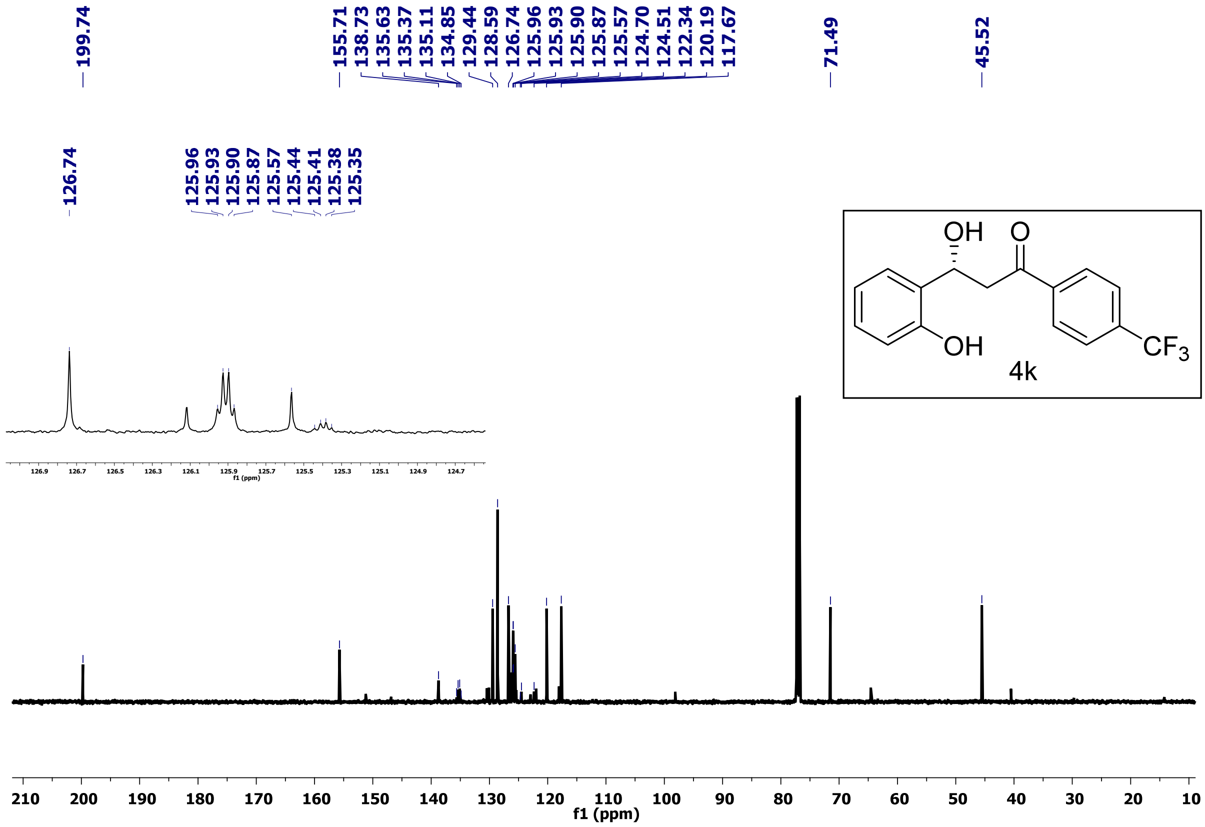
199.74
155.71 138.73 135.63 135.37 135.11 134.85 129.44 128.59 126.74 125.96 125.93 125.90 125.87 125.57 124.70 124.51 122.34 120.19 117.67
71.49
45.52
126.74
125.96 125.93 125.90 125.87 125.57 125.44 125.41 125.38 125.35
OH O
OH
CF3
4k
210 200 190 180 170 160 150 140 130 120 110 100 90 80 70 60 50 40 30 20 10
f1 (ppm)

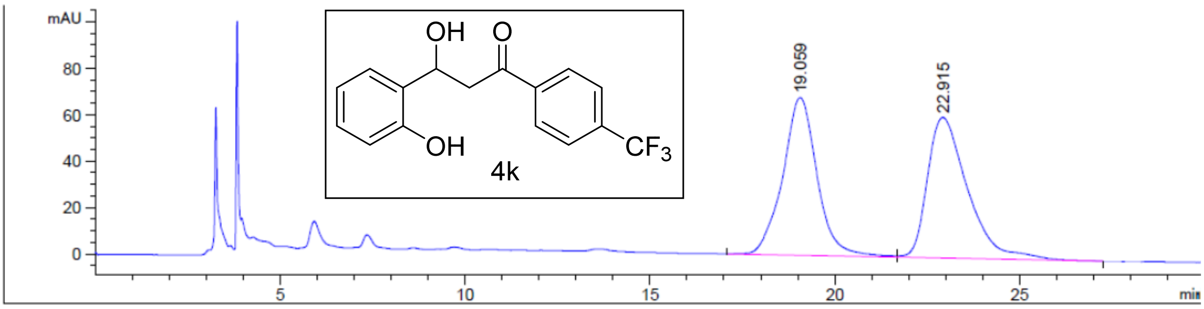

Signal 1: DAD1 A, Sig=254,4 Ref=360,100

Peak #	RetTime [min]	Type	Width [min]	Area [mAU*s]	Height [mAU]	Area %
1	19.059	BV	0.9560	4408.24219	68.02725	49.0613
2	22.915	VB	1.1368	4576.93750	60.62516	50.9387

Totals : 8985.17969 128.65241

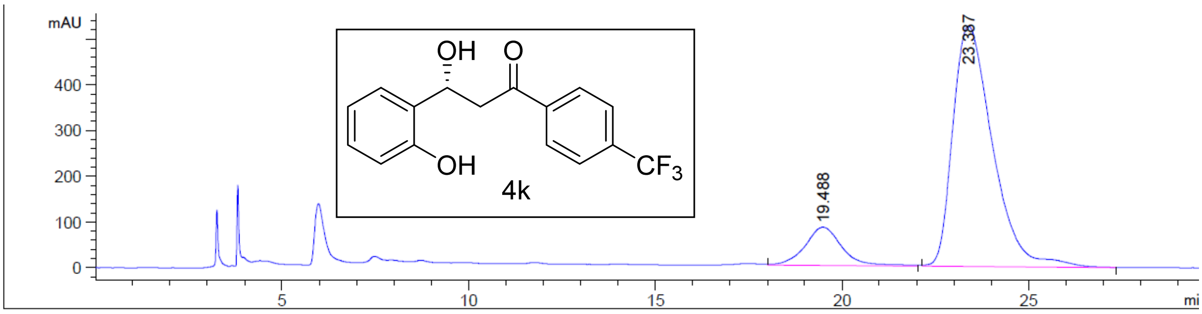

Signal 1: DAD1 A, Sig=254,4 Ref=360,100

Peak #	RetTime [min]	Type	Width [min]	Area [mAU*s]	Height [mAU]	Area %
1	19.488	VV	1.0323	5930.65088	84.34931	13.2290
2	23.387	VB	1.1361	3.89001e4	526.42816	86.7710

Totals : 4.48308e4 610.77747

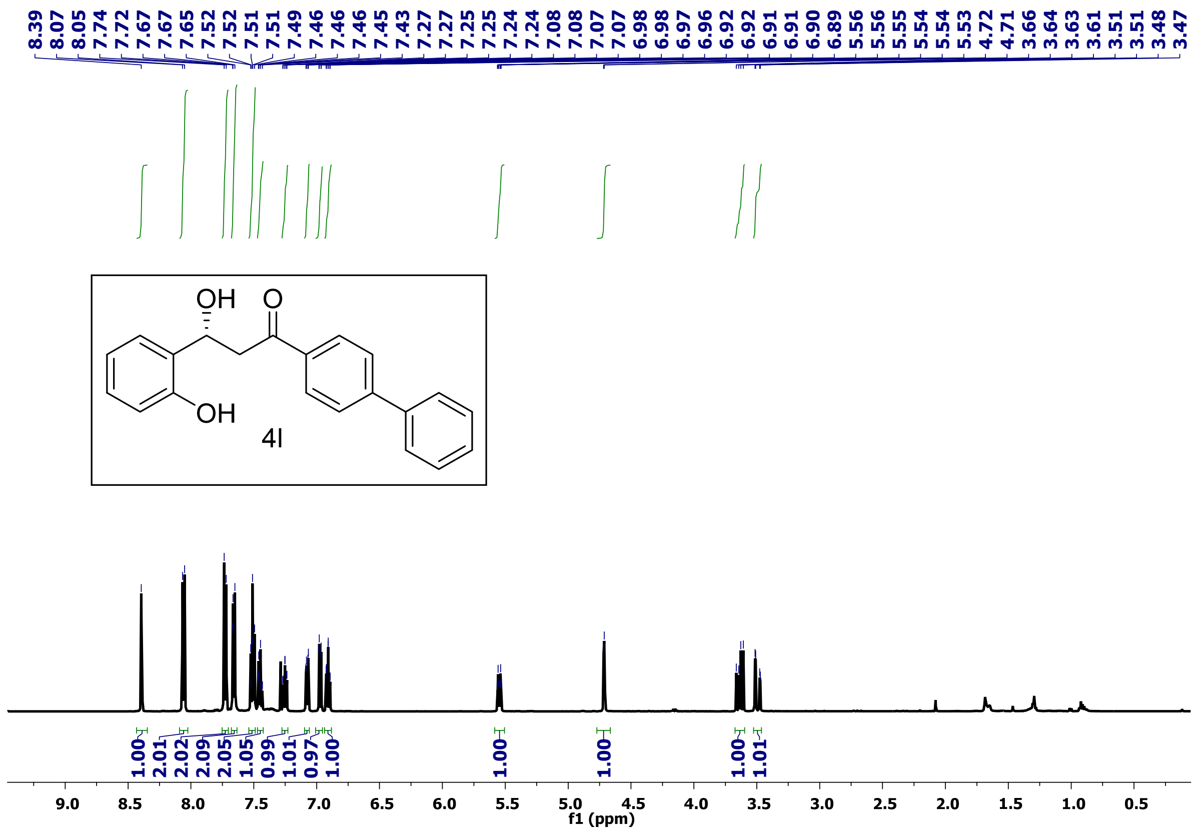
8.39 8.07 8.05 7.74 7.72 7.67 7.67 7.65 7.52 7.52 7.51 7.51 7.49 7.46 7.46 7.45 7.43 7.27 7.27 7.25 7.25 7.24 7.24 7.08 7.08 7.07 7.07 6.98 6.98 6.97 6.96 6.92 6.92 6.91 6.91 6.90 6.89 5.56 5.56 5.55 5.54 5.54 5.53 4.72 4.71 3.66 3.64 3.63 3.61 3.51 3.48 3.47
OH O
OH
4l
1.00 2.01 2.02 2.09 2.05 1.05 0.99 1.01 0.97 1.00 1.00 1.00 1.00 1.01
9.0 8.5 8.0 7.5 7.0 6.5 6.0 5.5 5.0 4.5 4.0 3.5 3.0 2.5 2.0 1.5 1.0 0.5
f1 (ppm)

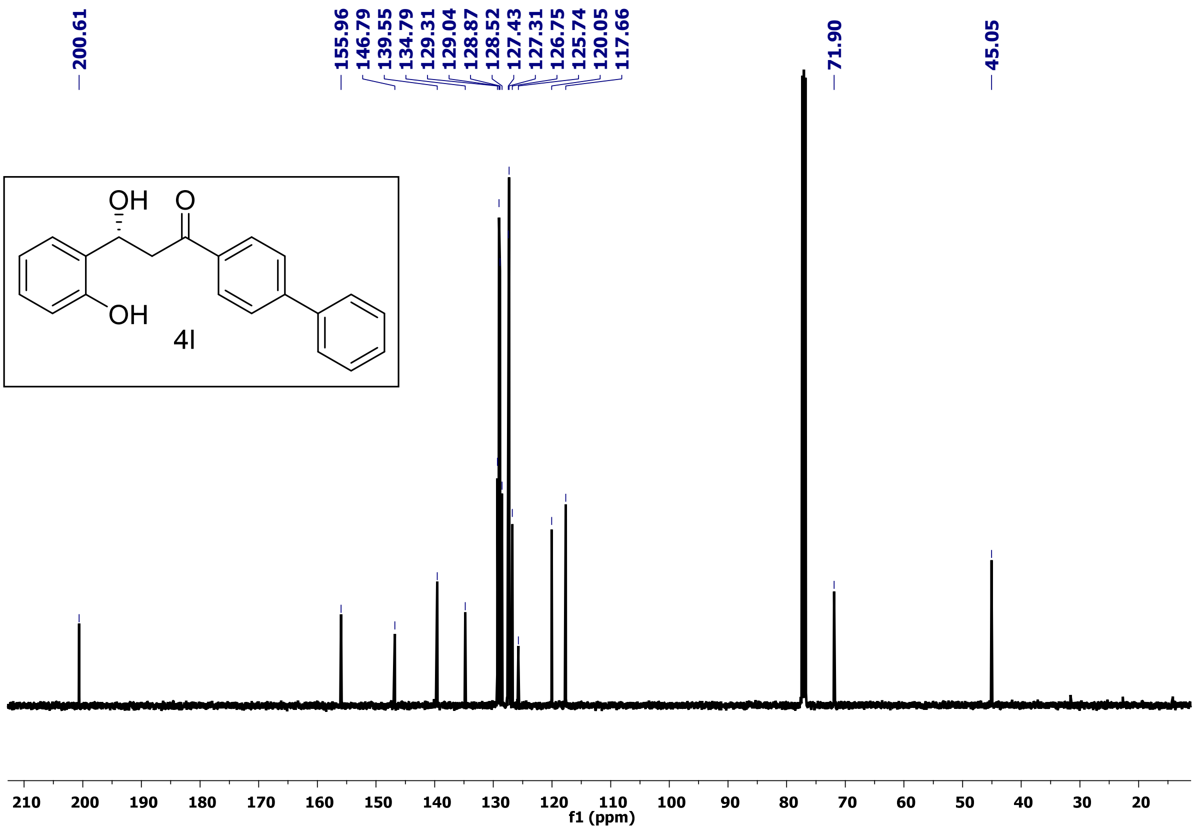
200.61
155.96 146.79 139.55 134.79 129.31 129.04 128.87 128.52 127.43 127.31 126.75 125.74 120.05 117.66
71.90
45.05
OH O
OH
4l
210 200 190 180 170 160 150 140 130 120 110 100 90 80 70 60 50 40 30 20
f1 (ppm)

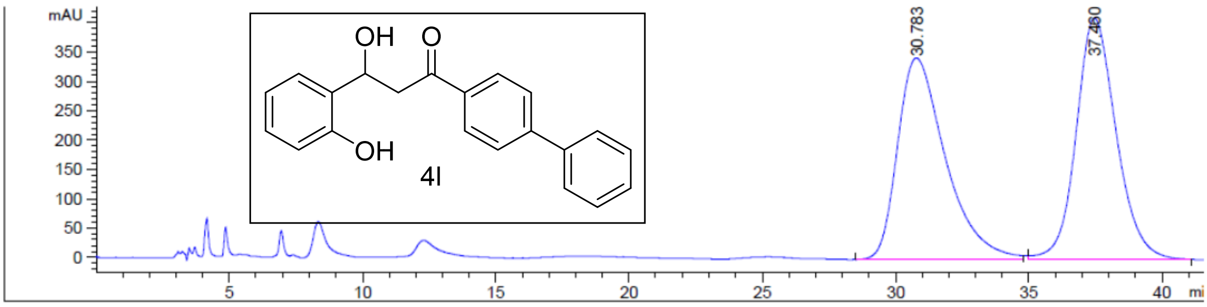

Signal 1: DAD1 A, Sig=254,4 Ref=360,100

Peak #	RetTime [min]	Type	Width [min]	Area [mAU*s]	Height [mAU]	Area %
1	30.783	BV	1.8358	4.29803e4	343.50677	49.9305
2	37.460	VV	1.5734	4.31000e4	411.70126	50.0695

Totals : 8.60803e4 755.20804

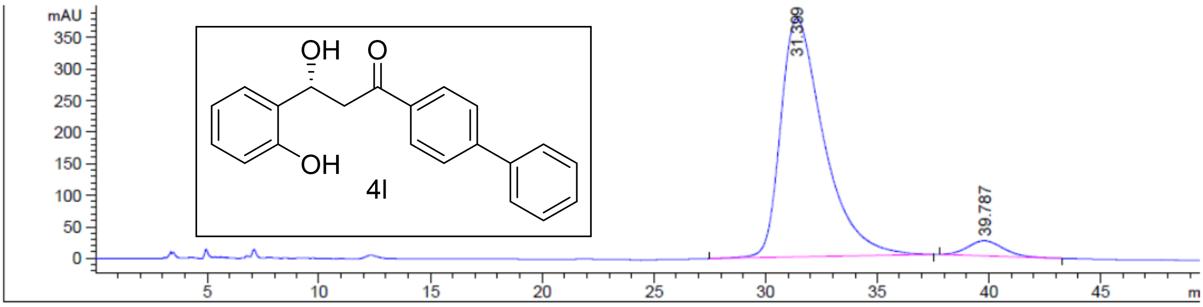

Signal 1: DAD1 A, Sig=254,4 Ref=360,100

Peak #	RetTime [min]	Type	Width [min]	Area [mAU*s]	Height [mAU]	Area %
1	31.399	BB	1.9537	5.08779e4	378.61795	95.1065
2	39.787	BB	1.4187	2617.80176	23.88510	4.8935

Totals : 5.34958e4 402.50305

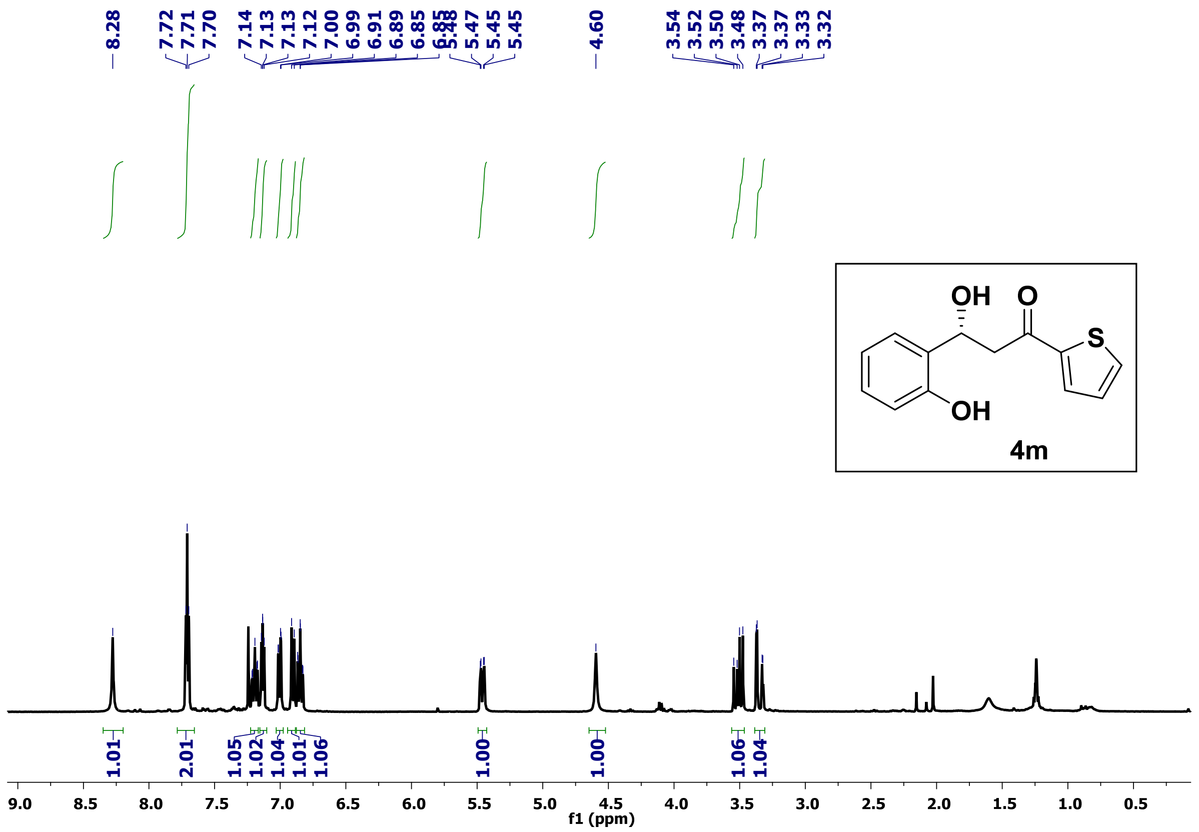

8.28
7.72
7.71
7.70
7.14
7.13
7.13
7.12
7.00
6.99
6.91
6.89
6.85
5.47
5.45
5.45
4.60
3.54
3.52
3.50
3.48
3.37
3.37
3.33
3.32
OH O
S
OH
4m
1.01
2.01
1.05
1.02
1.04
1.01
1.06
1.00
1.00
1.06
1.04
f1 (ppm)

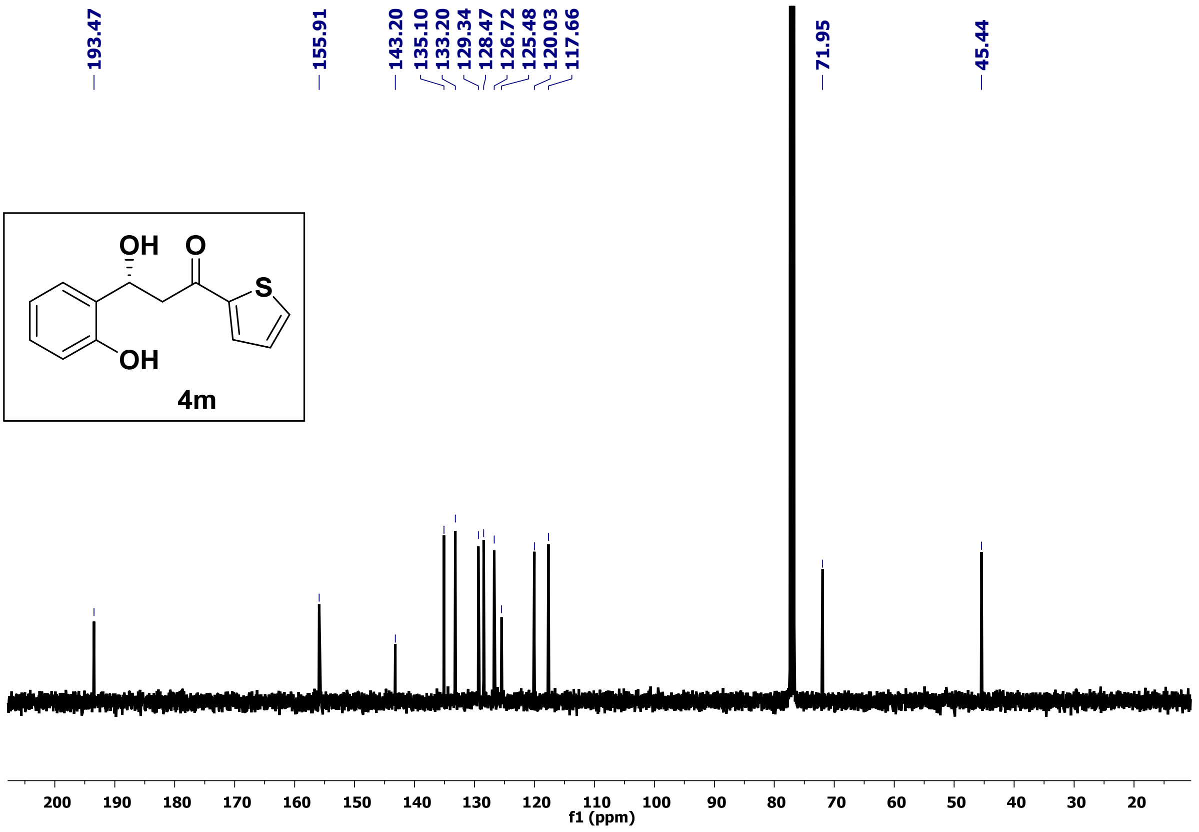

193.47
155.91
143.20
135.10
133.20
129.34
128.47
126.72
125.48
120.03
117.66
71.95
45.44
OH O
S
OH
4m
f1 (ppm)

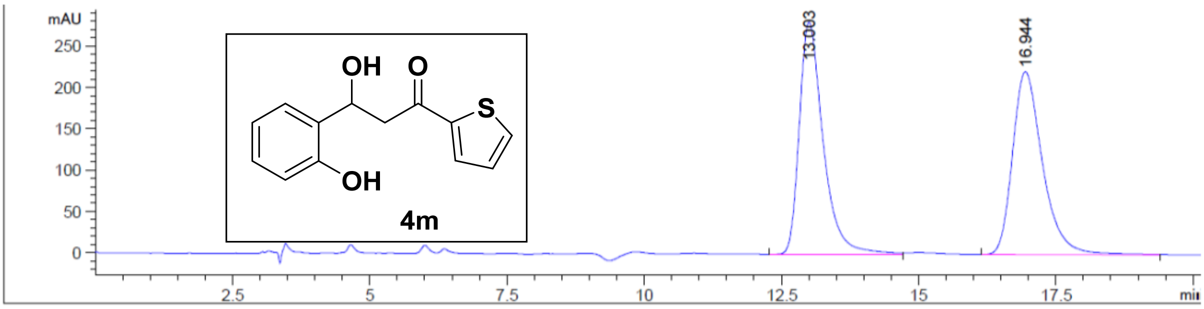

Signal 1: DAD1 A, Sig=254,4 Ref=360,100

Peak #	RetTime [min]	Type	Width [min]	Area [mAU*s]	Height [mAU]	Area %
1	13.003	BV	0.4580	8515.61328	280.65015	50.2562
2	16.944	BV	0.5767	8428.79590	221.13414	49.7438

Totals : 1.69444e4 501.78429

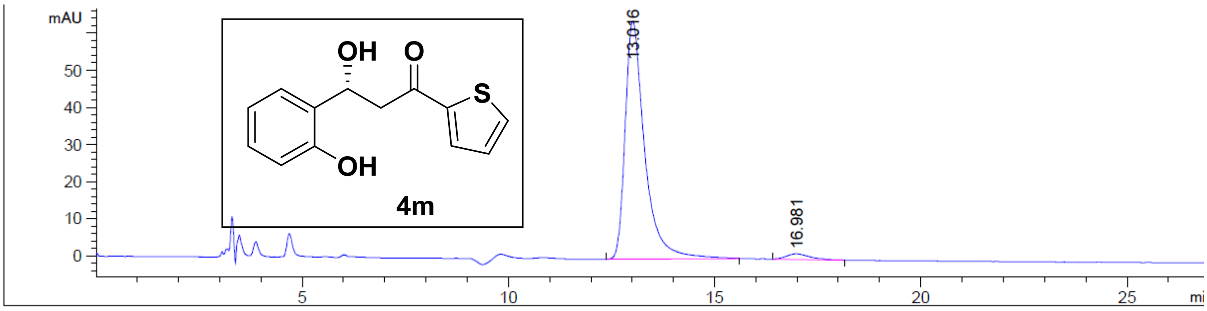

Signal 1: DAD1 A, Sig=254,4 Ref=360,100

Peak #	RetTime [min]	Type	Width [min]	Area [mAU*s]	Height [mAU]	Area %
1	13.016	BV	0.4926	2126.12329	64.15463	97.2544
2	16.981	VV	0.4916	60.02387	1.54381	2.7456

Totals : 2186.14716 65.69844

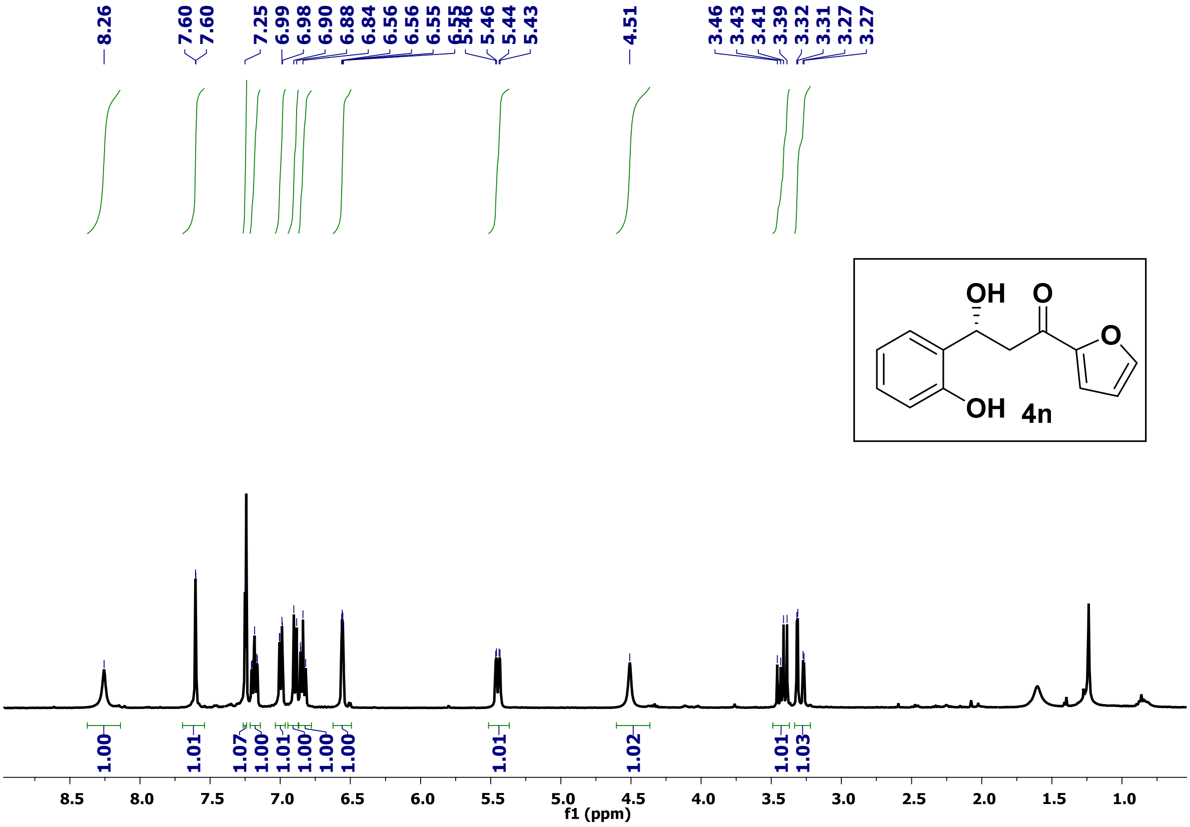

OH
O
O
OH 4n
f1 (ppm)

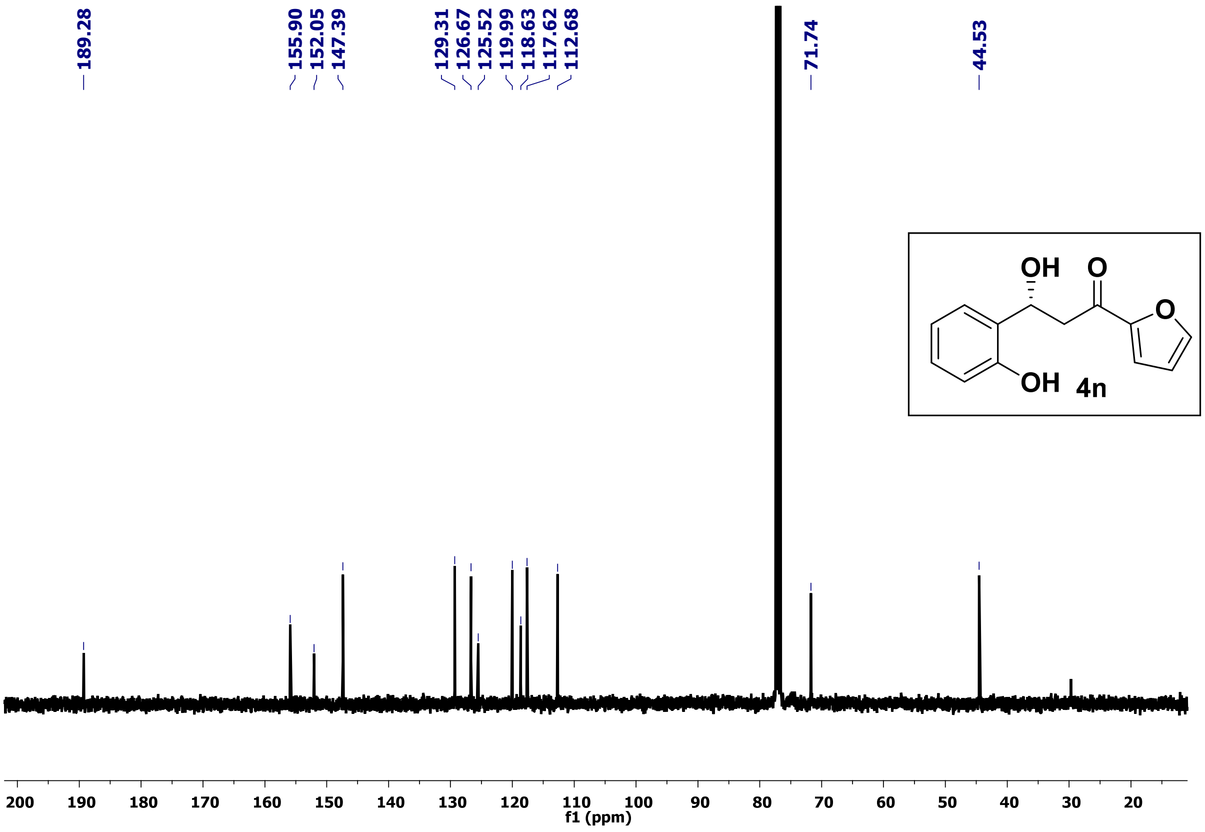

OH
O
O
OH 4n
f1 (ppm)

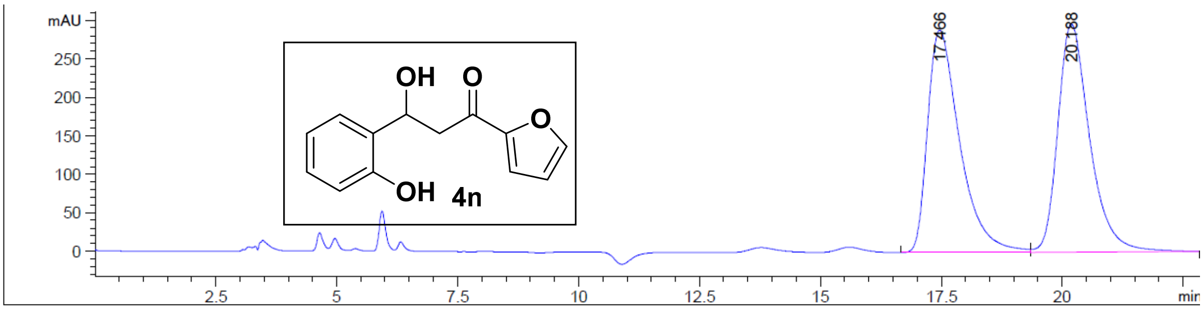

Signal 1: DAD1 A, Sig=254,4 Ref=360,100

Peak #	RetTime [min]	Type	Width [min]	Area [mAU*s]	Height [mAU]	Area %
1	17.466	BV	0.7019	1.33765e4	289.05670	49.6001
2	20.188	VV	0.6945	1.35922e4	296.68314	50.3999
Totals :				2.69687e4	585.73984	

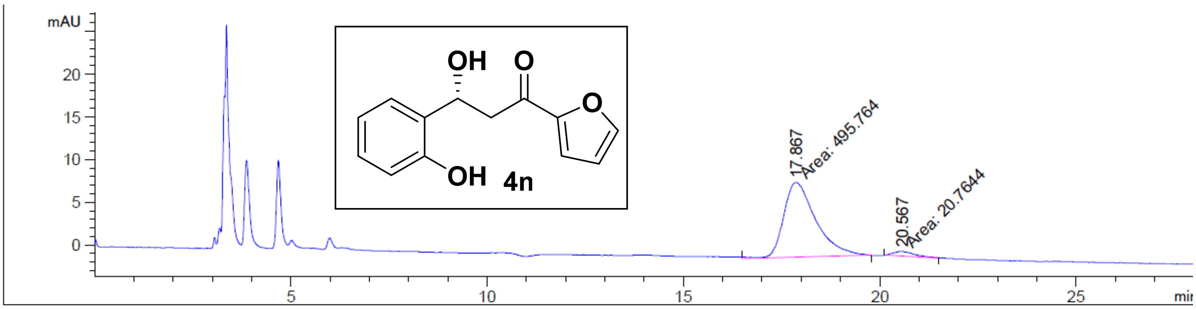

Signal 1: DAD1 A, Sig=254,4 Ref=360,100

Peak #	RetTime [min]	Type	Width [min]	Area [mAU*s]	Height [mAU]	Area %
1	17.867	MM	0.9460	495.76361	8.73403	95.9800
2	20.567	MM	0.6473	20.76440	5.34680e-1	4.0200
Totals :				516.52802	9.26871	

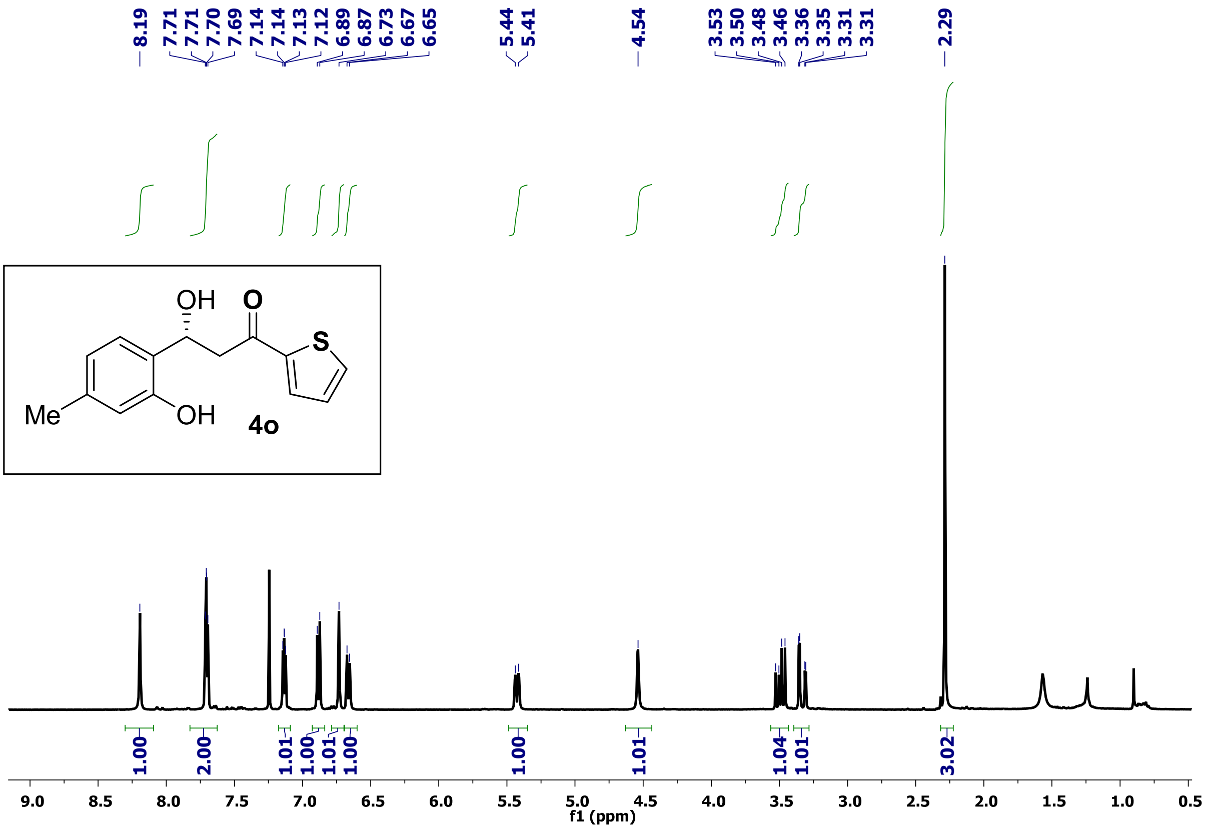
8.19
7.71
7.71
7.70
7.69
7.14
7.14
7.13
7.12
6.89
6.87
6.73
6.67
6.65
5.44
5.41
4.54
3.53
3.50
3.48
3.46
3.36
3.35
3.31
3.31
2.29
OH
O
S
Me
OH
4o
1.00
2.00
1.01
1.00
1.01
1.00
1.00
1.01
1.04
1.01
3.02
9.0 8.5 8.0 7.5 7.0 6.5 6.0 5.5 5.0 4.5 4.0 3.5 3.0 2.5 2.0 1.5 1.0 0.5
f1 (ppm)

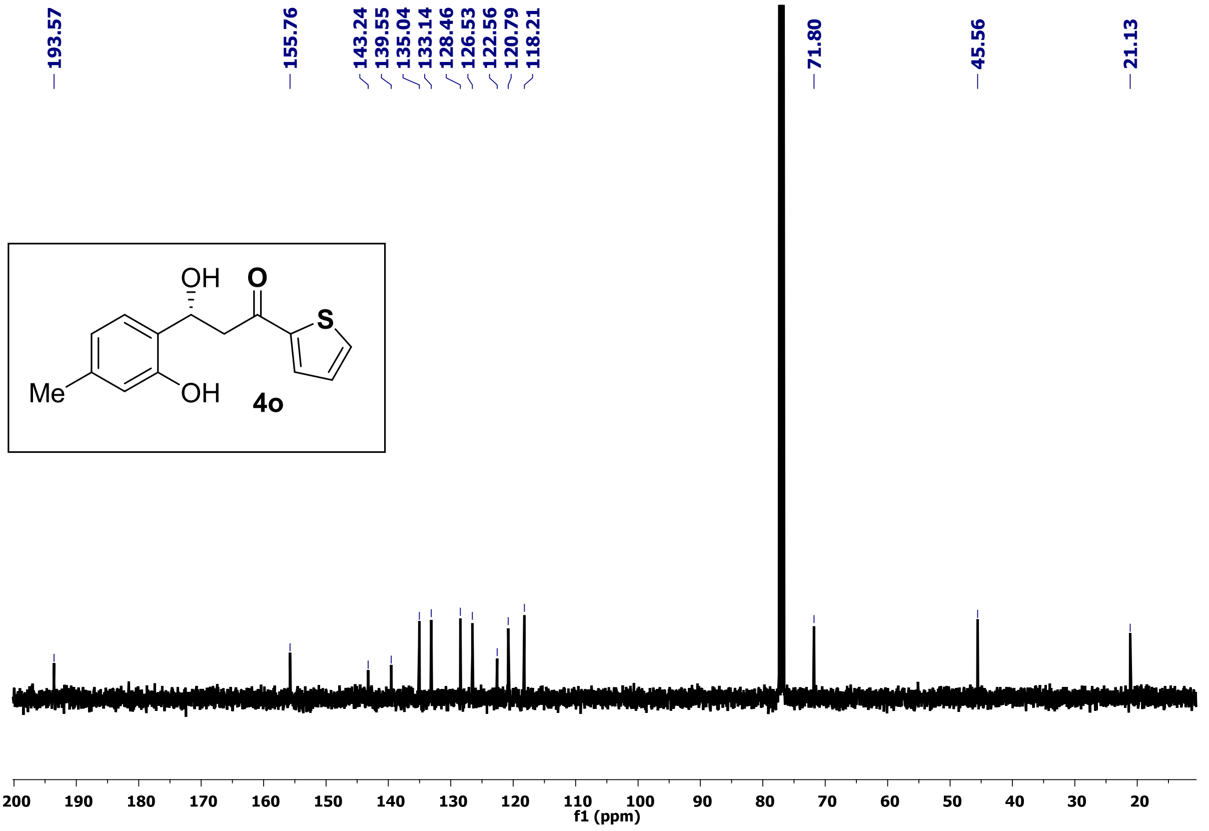
193.57
155.76
143.24
139.55
135.04
133.14
128.46
126.53
122.56
120.79
118.21
71.80
45.56
21.13
OH
O
S
Me
OH
4o
200 190 180 170 160 150 140 130 120 110 100 90 80 70 60 50 40 30 20
f1 (ppm)

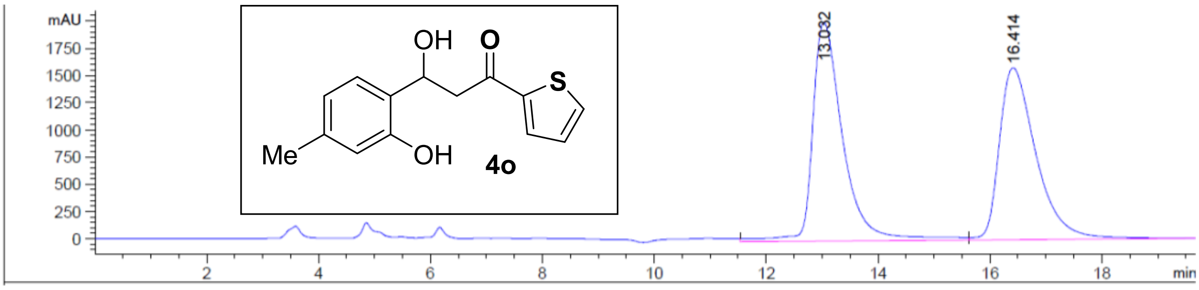

Signal 1: DAD1 A, Sig=254,4 Ref=360,100

Peak #	RetTime [min]	Type	Width [min]	Area [mAU*s]	Height [mAU]	Area %
1	13.032	VV	0.5654	7.61047e4	2011.32104	51.2915
2	16.414	VBA	0.6958	7.22721e4	1579.56274	48.7085

Totals : 1.48377e5 3590.88379

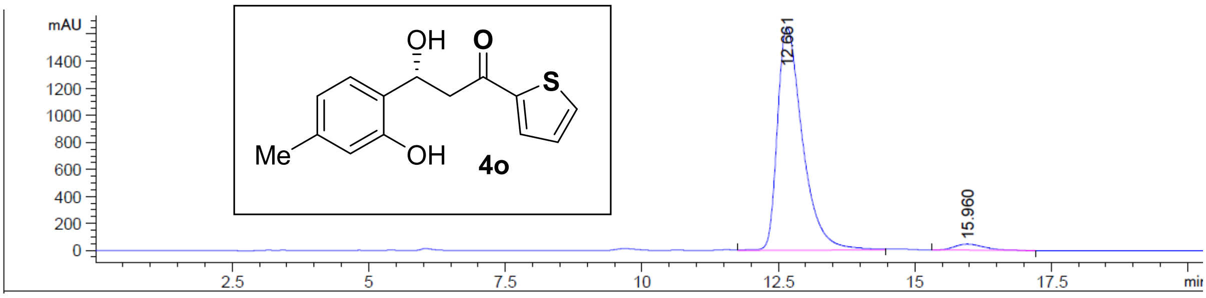

Signal 1: DAD1 A, Sig=254,4 Ref=360,100

Peak #	RetTime [min]	Type	Width [min]	Area [mAU*s]	Height [mAU]	Area %
1	12.661	VV	0.4853	5.28105e4	1650.61523	97.0025
2	15.960	BB	0.5661	1631.92285	44.26812	2.9975

Totals : 5.44424e4 1694.88335

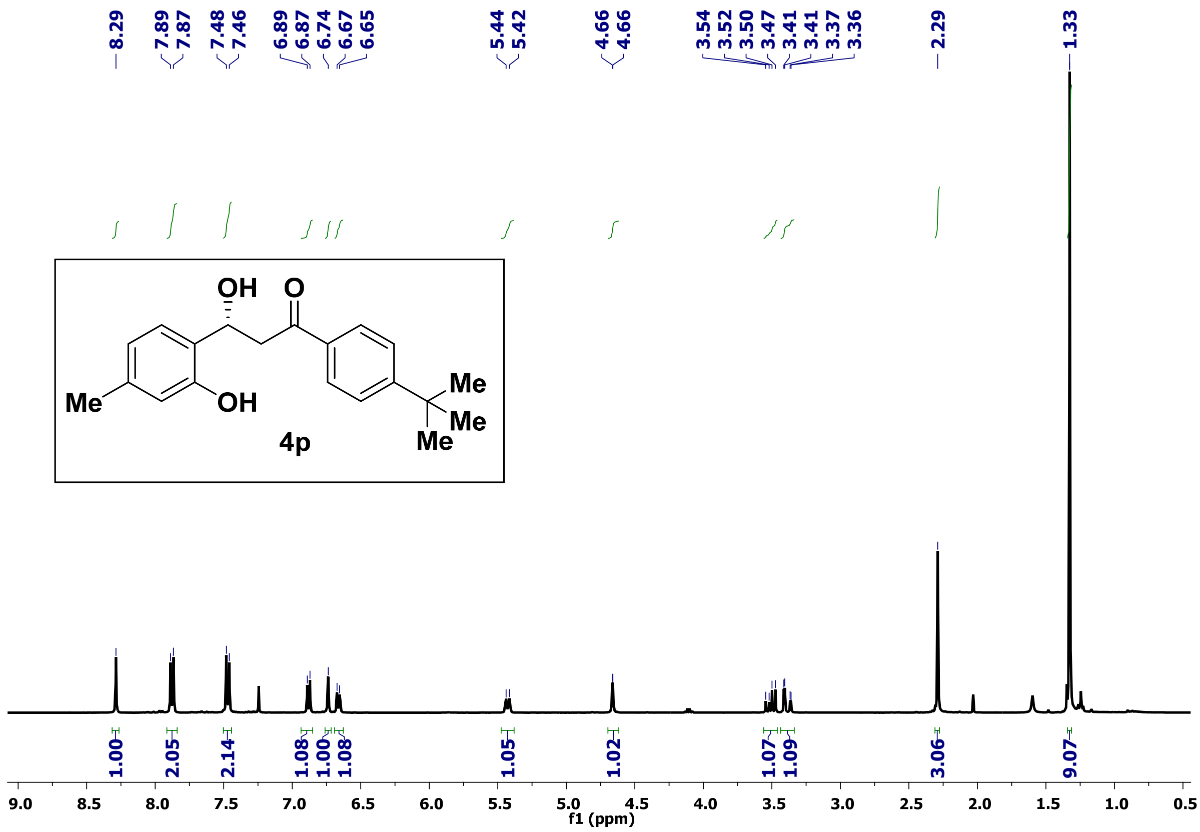
8.29
7.89
7.87
7.48
7.46
6.89
6.87
6.74
6.67
6.65
5.44
5.42
4.66
4.66
3.54
3.52
3.50
3.47
3.41
3.37
3.36
2.29
1.33
OH
O
Me
OH
4p
Me
Me
Me
1.00
2.05
2.14
1.08
1.00
1.08
1.05
1.02
1.07
1.09
3.06
9.07
9.0
8.5
8.0
7.5
7.0
6.5
6.0
5.5
5.0
4.5
4.0
3.5
3.0
2.5
2.0
1.5
1.0
0.5
f1 (ppm)

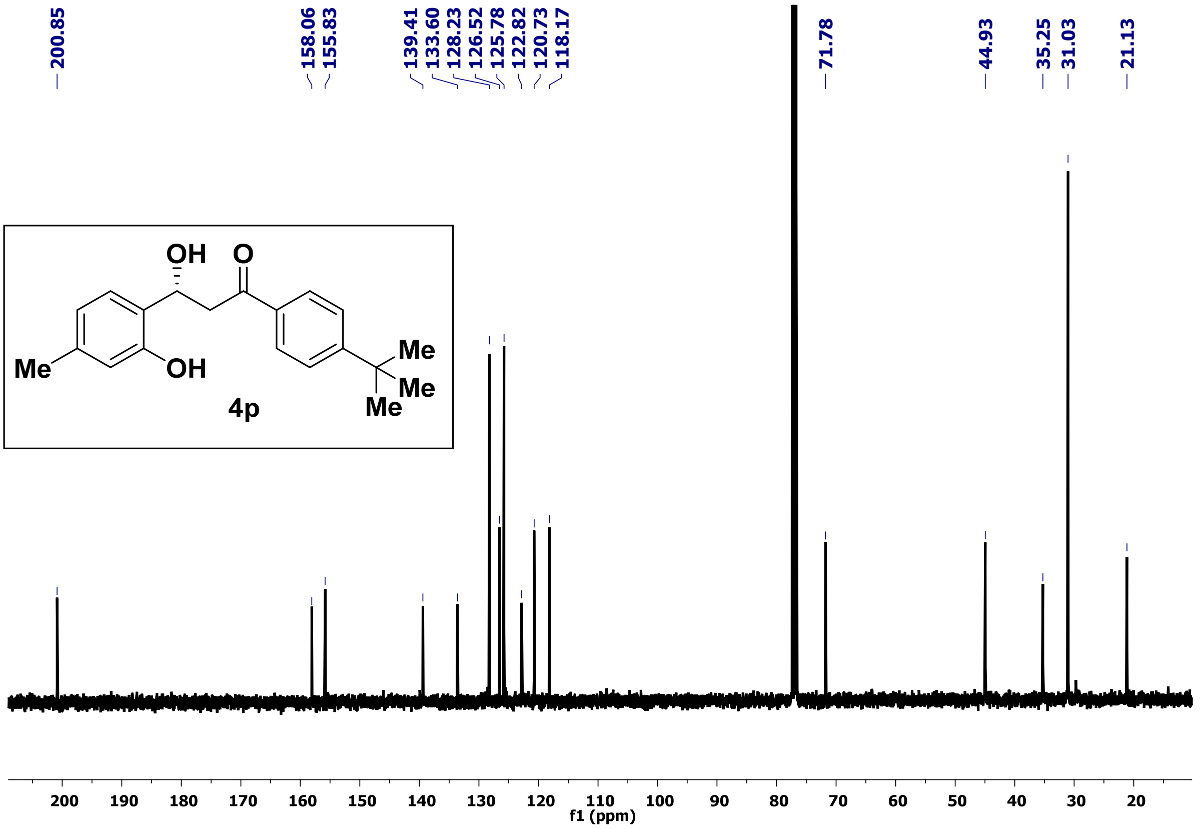
200.85
158.06
155.83
139.41
133.60
128.23
126.52
125.78
122.82
120.73
118.17
71.78
44.93
35.25
31.03
21.13
OH
O
Me
OH
4p
Me
Me
Me
200
190
180
170
160
150
140
130
120
110
100
90
80
70
60
50
40
30
20
f1 (ppm)

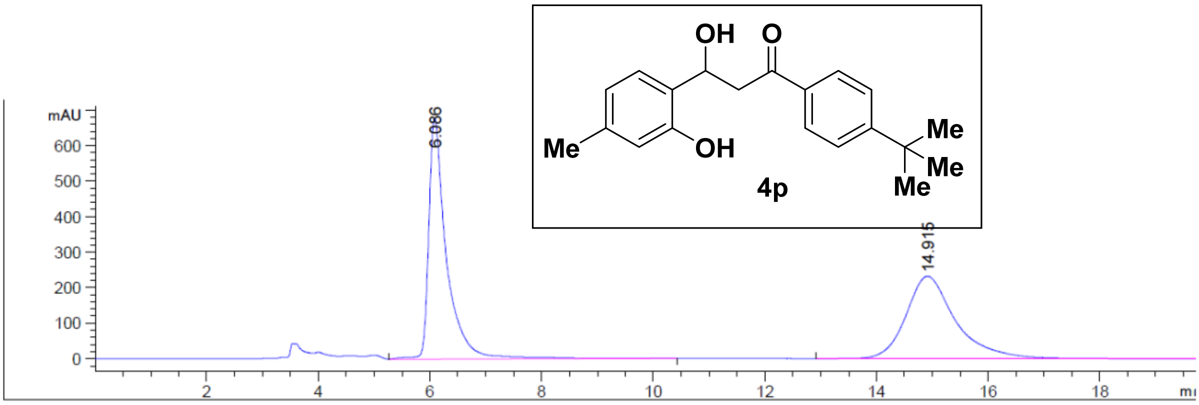

Signal 1: DAD1 A, Sig=254,4 Ref=360,100

Peak #	RetTime [min]	Type	Width [min]	Area [mAU*s]	Height [mAU]	Area %
1	6.086	BB	0.3199	1.50978e4	677.66595	51.2511
2	14.915	BBA	0.9102	1.43608e4	231.97906	48.7489

Totals : 2.94586e4 909.64502

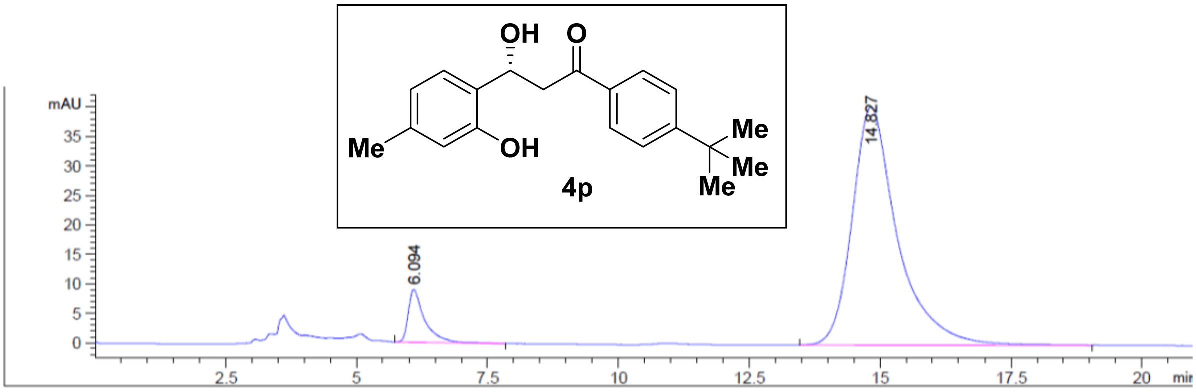

Signal 1: DAD1 A, Sig=254,4 Ref=360,100

Peak #	RetTime [min]	Type	Width [min]	Area [mAU*s]	Height [mAU]	Area %
1	6.094	BB	0.3137	194.96072	8.96444	7.4127
2	14.827	BB	0.8832	2435.13867	40.39837	92.5873

Totals : 2630.09940 49.36281

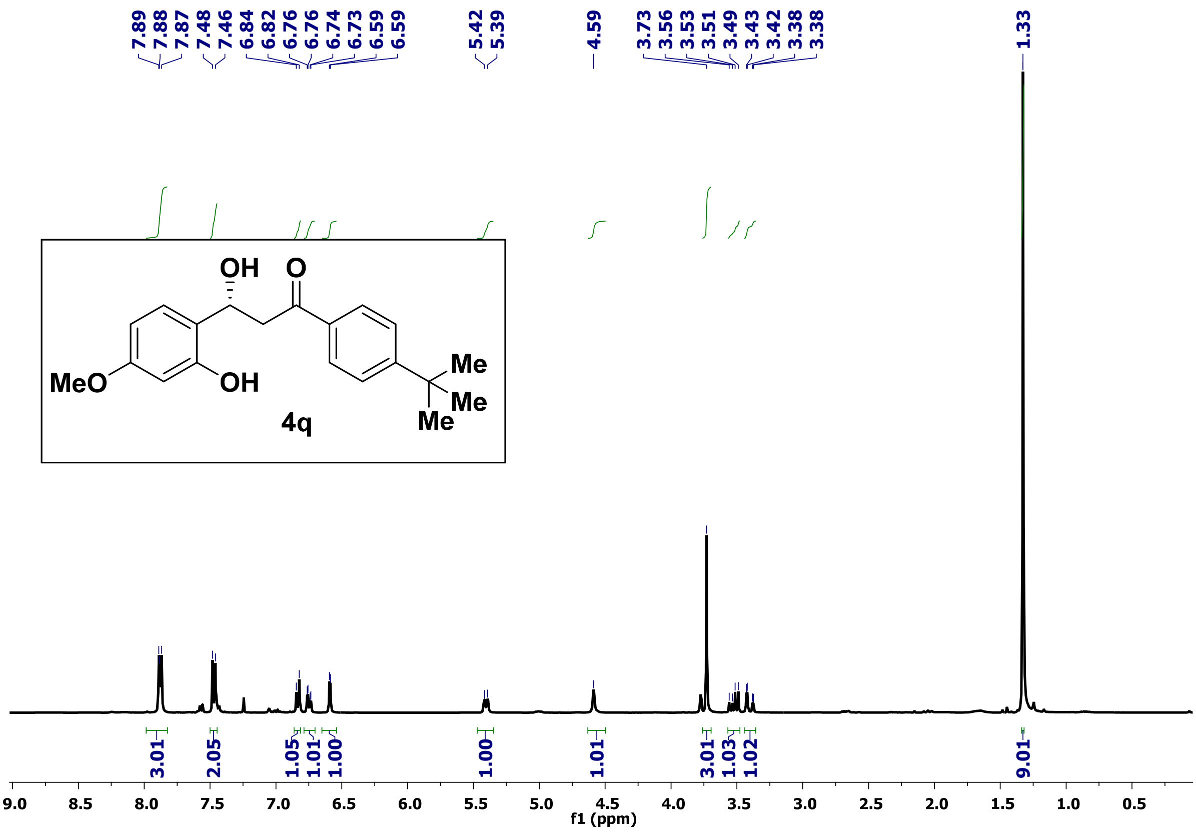
7.89 7.88 7.87 7.48 7.46 6.84 6.82 6.76 6.76 6.74 6.73 6.59 6.59 6.59
5.42 5.39
4.59
3.73 3.56 3.53 3.51 3.49 3.43 3.42 3.38 3.38
1.33
OH O
MeO
OH
4q
Me
Me
Me
3.01 2.05 1.05 1.01 1.00 1.00 1.01 3.01 1.03 1.02 9.01
f1 (ppm)

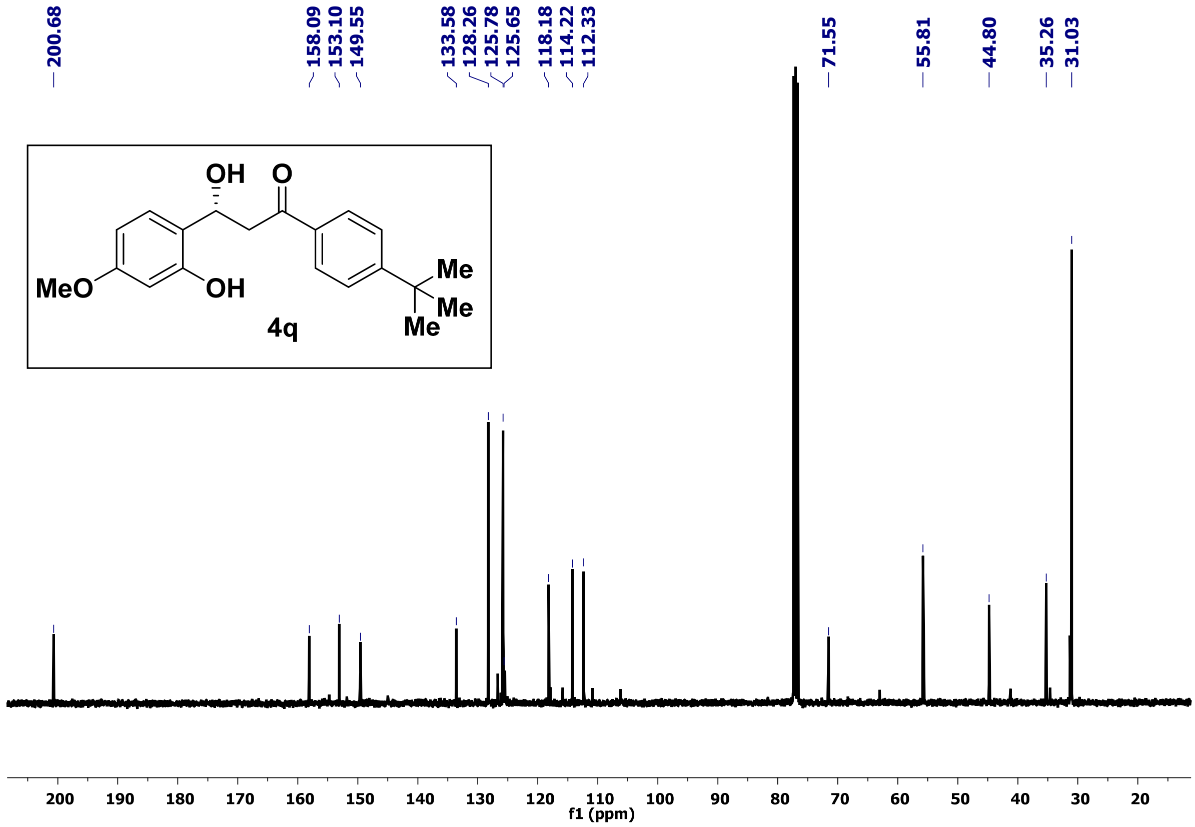
200.68
158.09 153.10 149.55
133.58 128.26 125.78 125.65 118.18 114.22 112.33
71.55
55.81
44.80
35.26 31.03
OH O
MeO
OH
4q
Me
Me
Me
f1 (ppm)

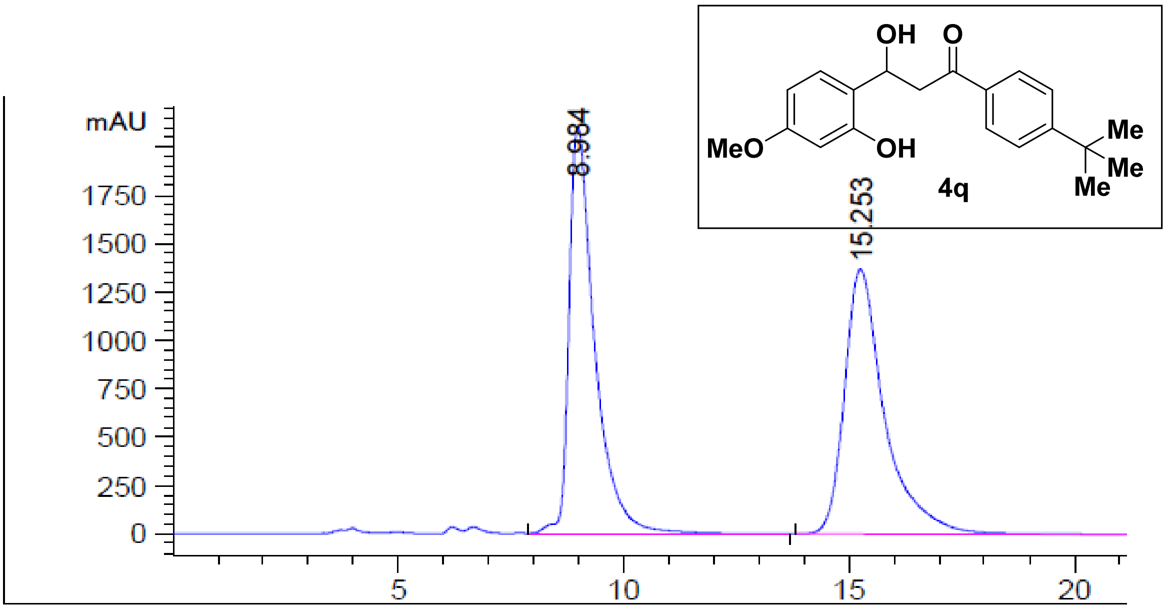

Signal 1: DAD1 A, Sig=254,4 Ref=360,100

Peak #	RetTime [min]	Type	Width [min]	Area [mAU*s]	Height [mAU]	Area %
1	8.984	VB	0.5857	8.35126e4	2101.17090	49.6360
2	15.253	BV	0.9146	8.47375e4	1368.10364	50.3640

Totals : 1.68250e5 3469.27454

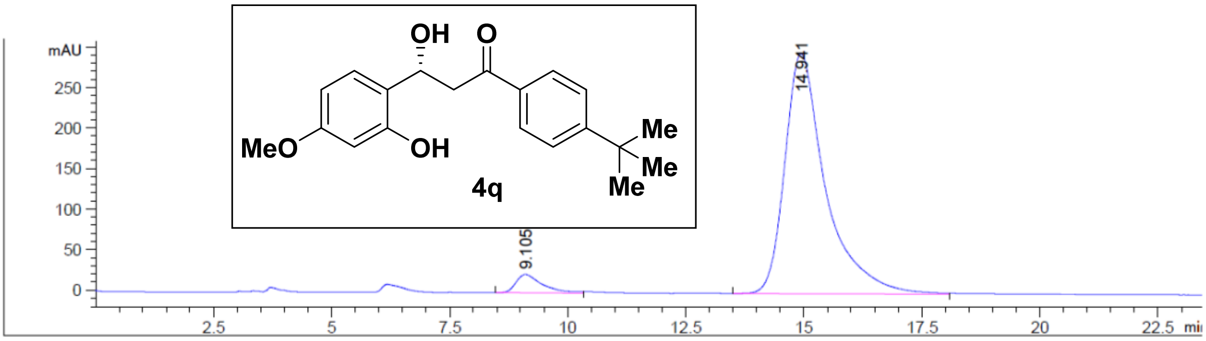

Signal 1: DAD1 A, Sig=254,4 Ref=360,100

Peak #	RetTime [min]	Type	Width [min]	Area [mAU*s]	Height [mAU]	Area %
1	9.105	VV	0.5727	880.63617	22.59742	4.7297
2	14.941	BV	0.8801	1.77385e4	295.61343	95.2703

Totals : 1.86191e4 318.21085

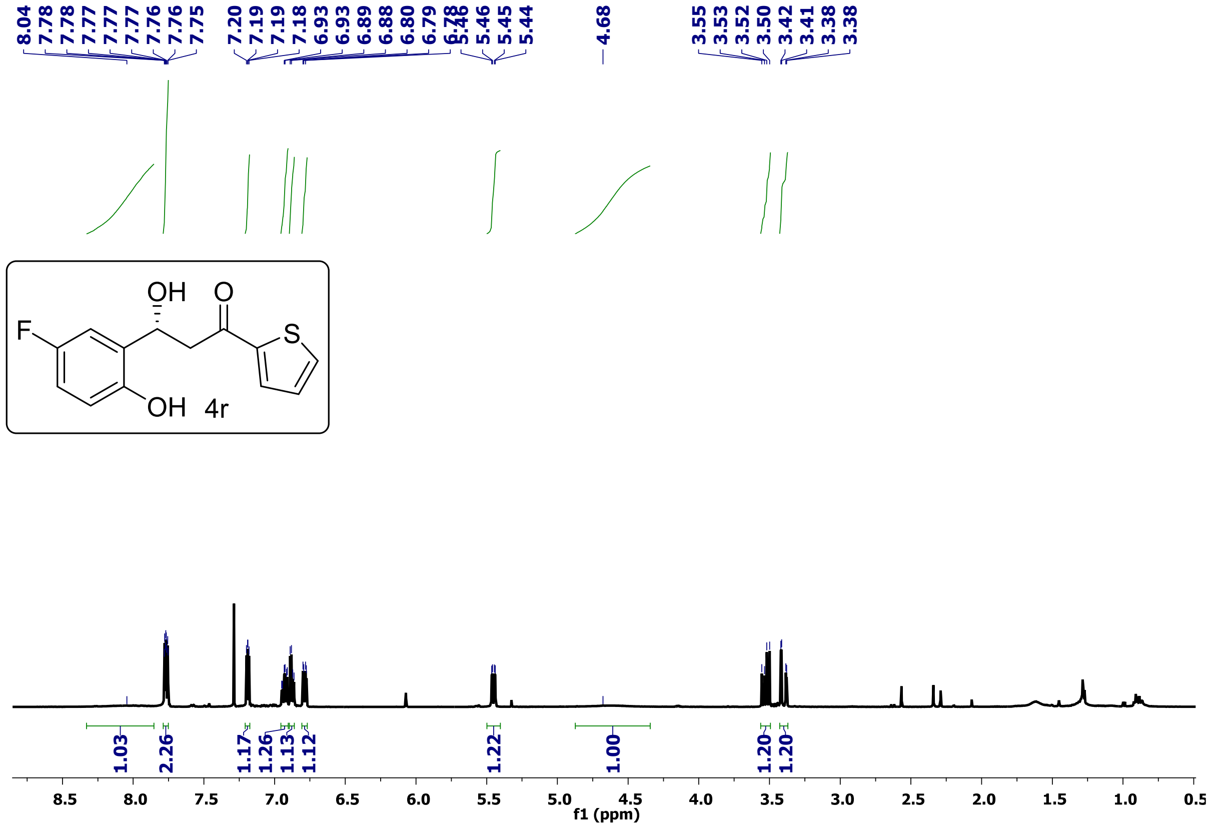
8.04 7.78 7.78 7.77 7.77 7.77 7.76 7.76 7.75 7.20 7.19 7.19 7.18 6.93 6.93 6.89 6.88 6.80 6.79 6.78 5.46 5.46 5.45 5.44 4.68 3.55 3.53 3.52 3.50 3.42 3.41 3.38 3.38
OH O
F
S
OH 4r
1.03 2.26 1.17 1.26 1.13 1.12 1.22 1.00 1.20 1.20
8.5 8.0 7.5 7.0 6.5 6.0 5.5 5.0 4.5 4.0 3.5 3.0 2.5 2.0 1.5 1.0 0.5
f1 (ppm)

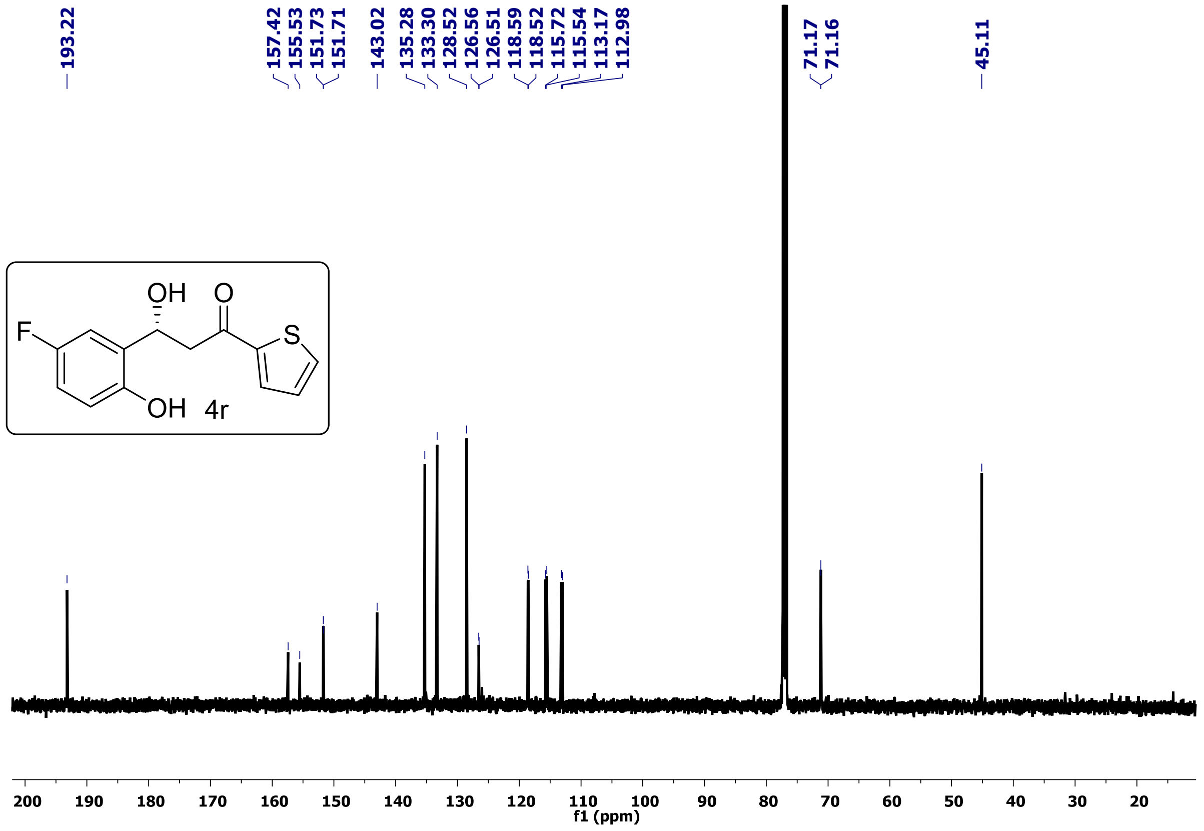
193.22 157.42 155.53 151.73 151.71 143.02 135.28 133.30 128.52 126.56 126.51 118.59 118.52 115.72 115.54 113.17 112.98 71.17 71.16 45.11
OH O
F
S
OH 4r
200 190 180 170 160 150 140 130 120 110 100 90 80 70 60 50 40 30 20
f1 (ppm)

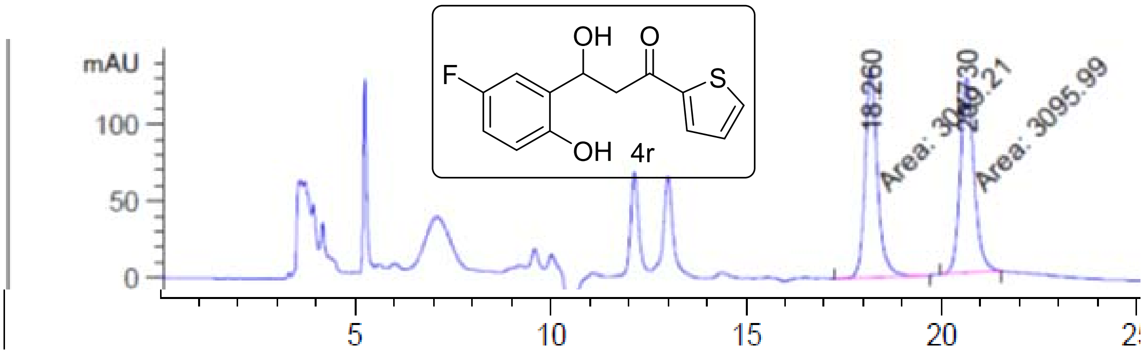

Signal 1: DAD1 A, Sig=254,4 Ref=360,100

Peak #	RetTime [min]	Type	Width [min]	Area [mAU*s]	Height [mAU]	Area %
1	18.260	MM	0.3717	3049.21191	136.71657	49.6194
2	20.730	MM	0.4078	3095.98706	126.51910	50.3806

Totals : 6145.19897 263.23567

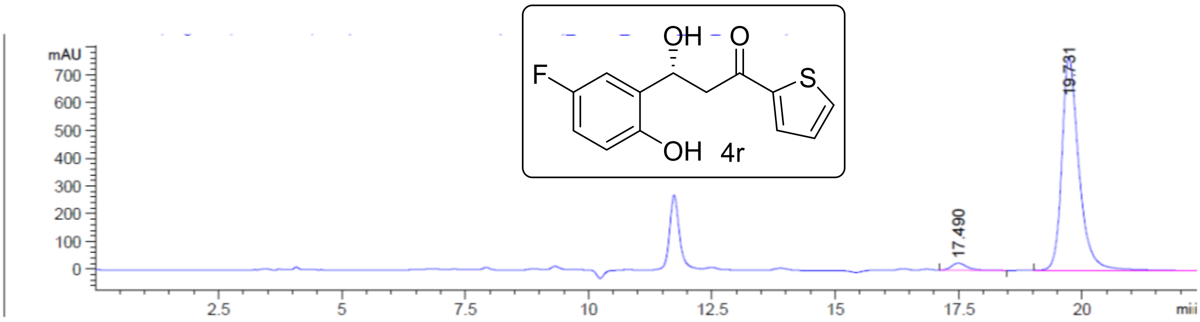

Signal 1: DAD1 A, Sig=254,4 Ref=360,100

Peak #	RetTime [min]	Type	Width [min]	Area [mAU*s]	Height [mAU]	Area %
1	17.490	BB	0.3268	562.43500	26.16525	2.9452
2	19.731	VBA	0.3612	1.85345e4	768.93341	97.0548

Totals : 1.90969e4 795.09867

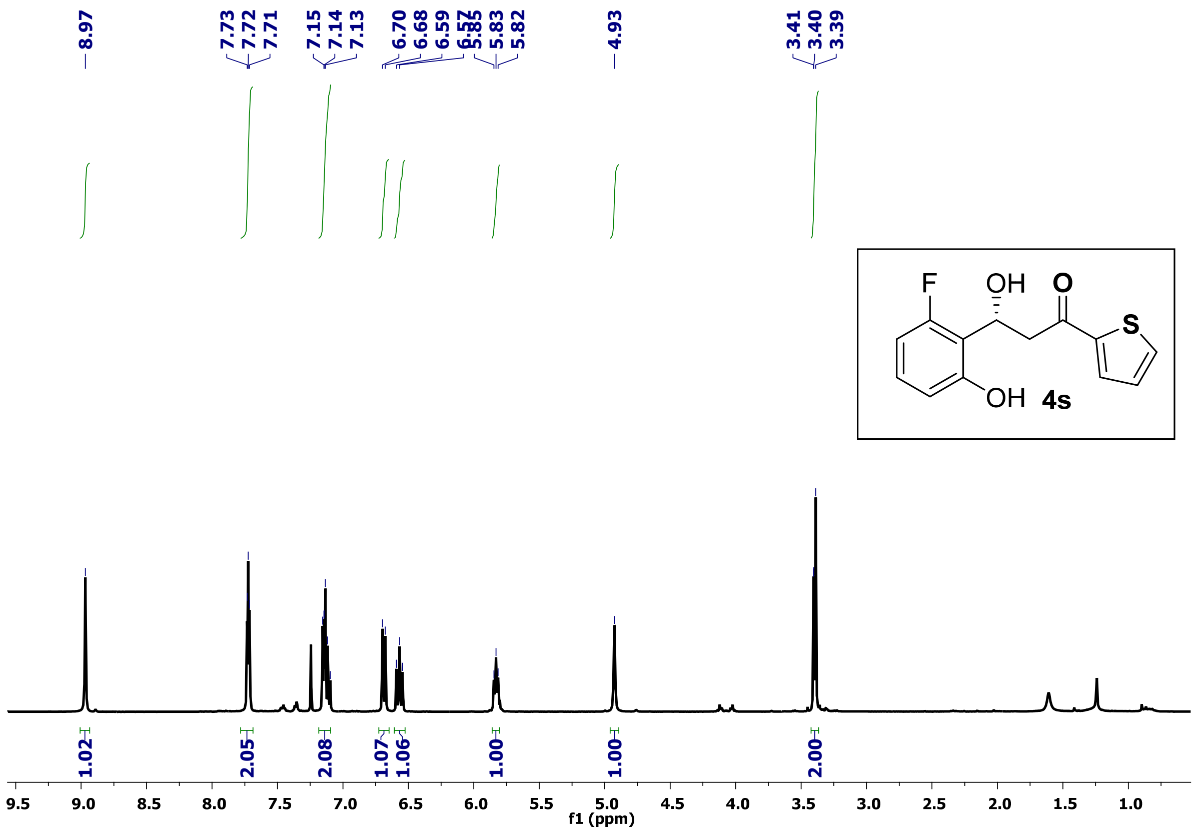
8.97
7.73
7.72
7.71
7.15
7.14
7.13
6.70
6.68
6.59
5.85
5.83
5.82
4.93
3.41
3.40
3.39
F
OH
O
S
OH
4s
1.02
2.05
2.08
1.07
1.06
1.00
1.00
2.00
f1 (ppm)

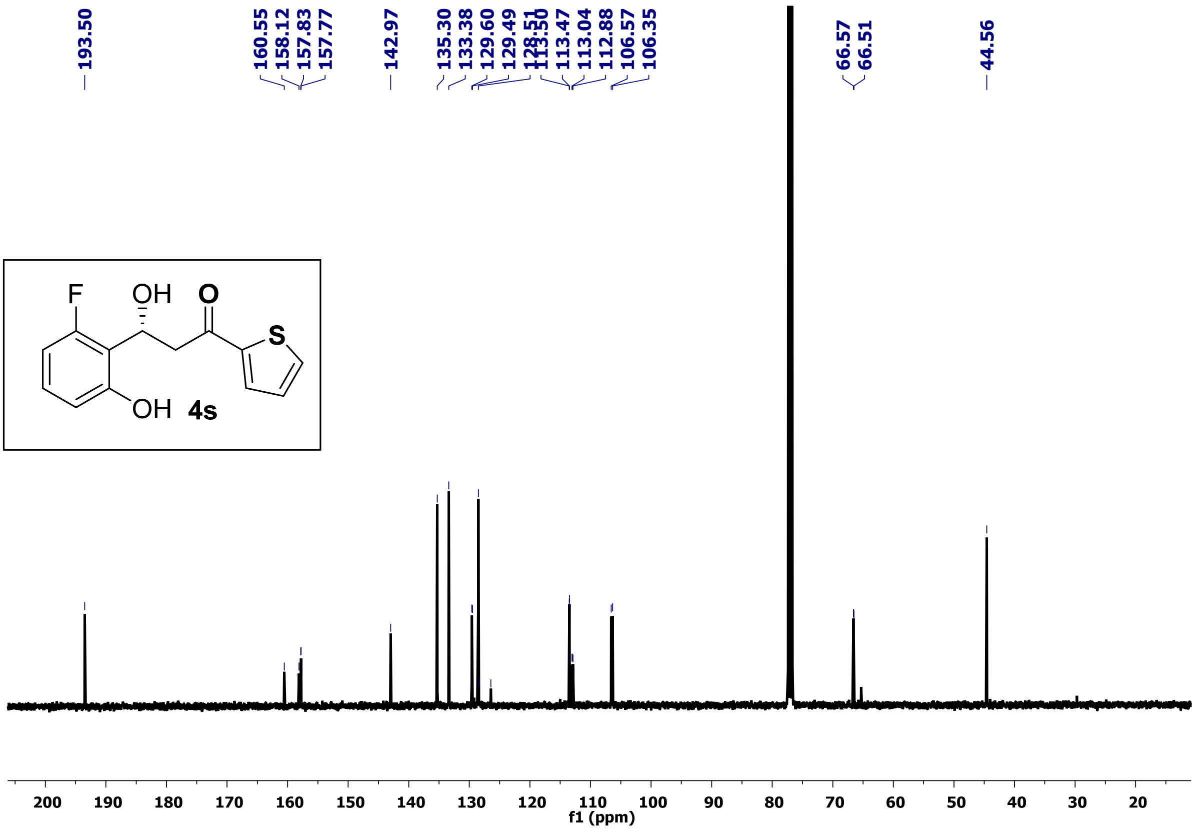
193.50
160.55
158.12
157.83
157.77
142.97
135.30
133.38
129.60
129.49
113.47
113.04
112.88
106.57
106.35
66.57
66.51
44.56
F
OH
O
S
OH
4s
f1 (ppm)

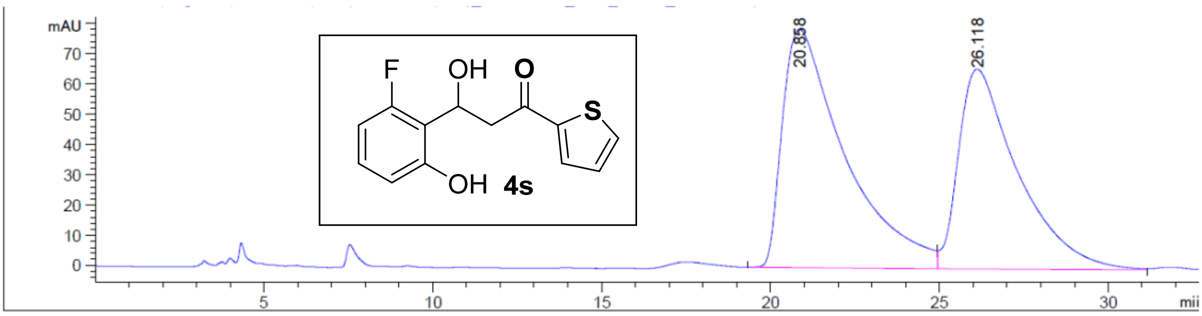

Signal 1: DAD1 A, Sig=254,4 Ref=360,100

Peak #	RetTime [min]	Type	Width [min]	Area [mAU*s]	Height [mAU]	Area %
1	20.858	BV	1.8182	1.01581e4	78.78547	55.7290
2	26.118	VV	1.7698	8069.55225	66.02747	44.2710
Totals :				1.82276e4	144.81294	

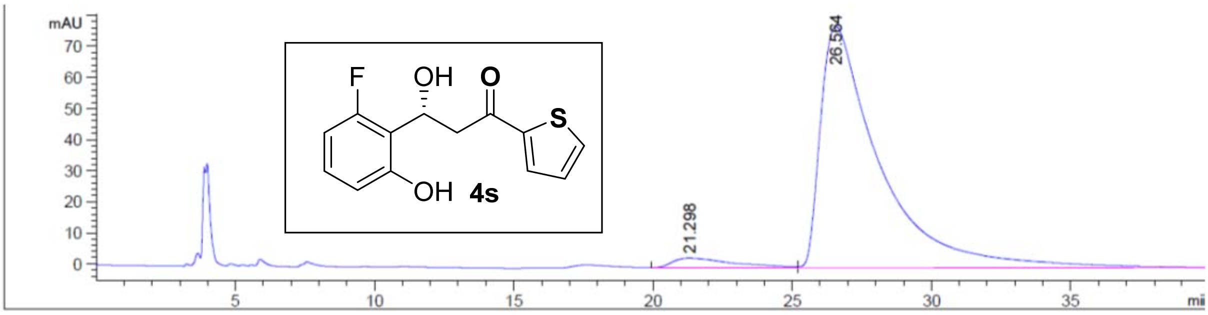

Signal 1: DAD1 A, Sig=254,4 Ref=360,100

Peak #	RetTime [min]	Type	Width [min]	Area [mAU*s]	Height [mAU]	Area %
1	21.298	VV	1.8410	497.55179	3.24103	4.1127
2	26.564	VBA	2.1052	1.16005e4	77.72594	95.8873
Totals :				1.20981e4	80.96697	

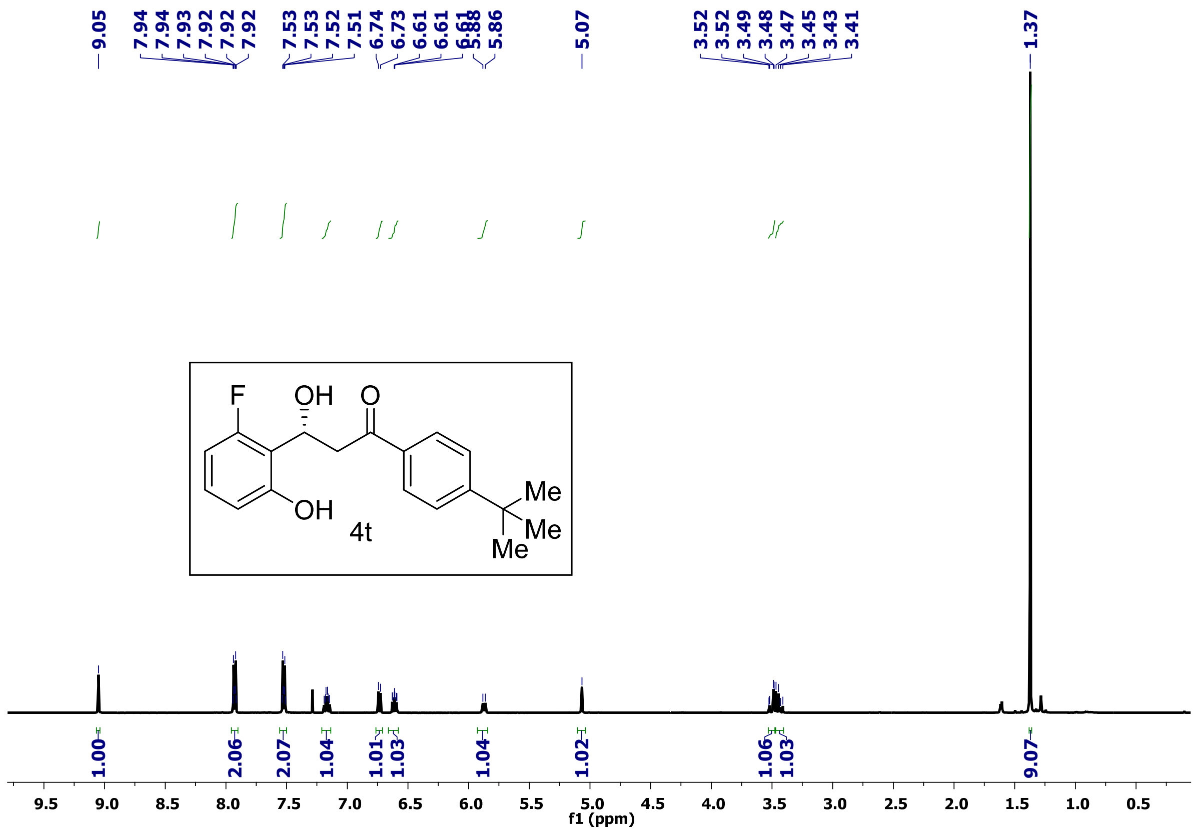
9.05
7.94
7.94
7.93
7.92
7.92
7.92
7.53
7.53
7.52
7.51
6.74
6.73
6.61
6.61
6.61
5.88
5.86
5.07
3.52
3.52
3.49
3.48
3.47
3.45
3.43
3.41
1.37
F
OH
O
OH
4t
Me
Me
Me
1.00
2.06
2.07
1.04
1.01
1.03
1.04
1.02
1.06
1.03
9.07
9.5
9.0
8.5
8.0
7.5
7.0
6.5
6.0
5.5
5.0
4.5
4.0
3.5
3.0
2.5
2.0
1.5
1.0
0.5
f1 (ppm)

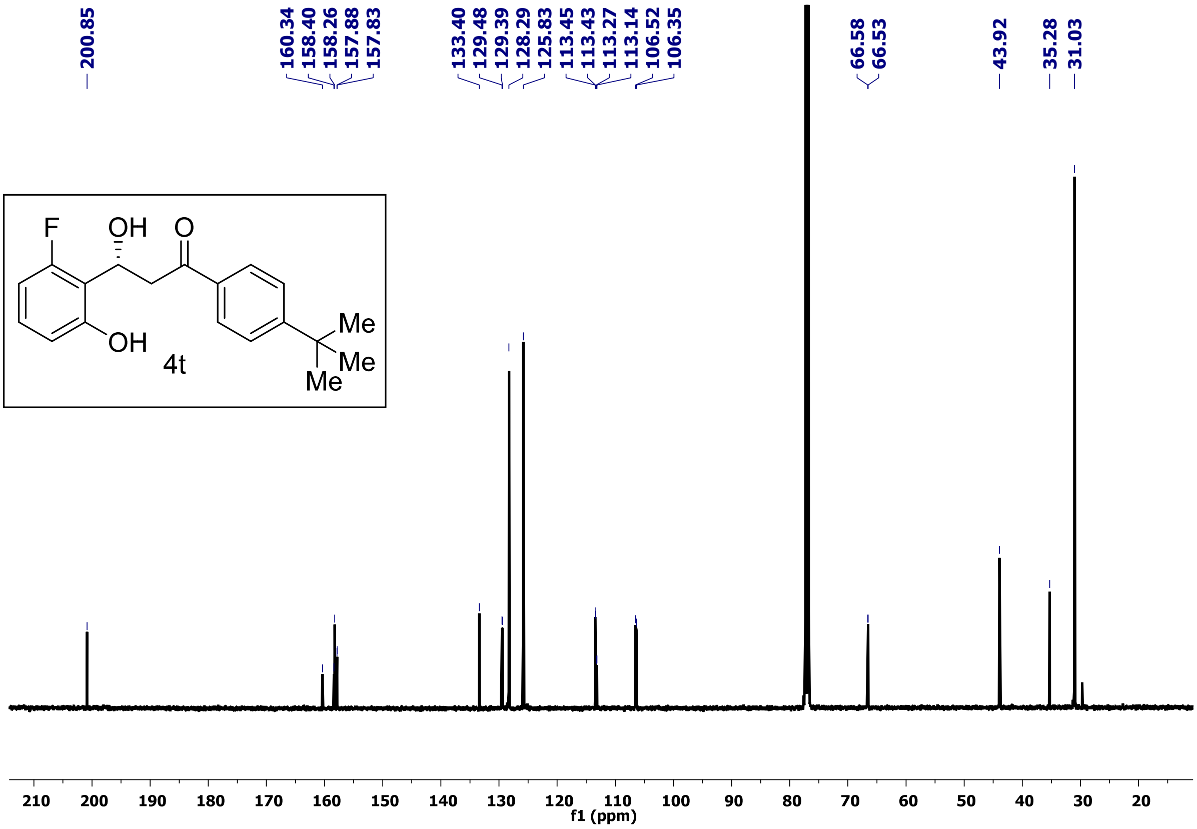
200.85
160.34
158.40
158.26
157.88
157.83
133.40
129.48
129.39
128.29
125.83
113.45
113.43
113.27
113.14
106.52
106.35
66.58
66.53
43.92
35.28
31.03
F
OH
O
OH
4t
Me
Me
Me
210
200
190
180
170
160
150
140
130
120
110
100
90
80
70
60
50
40
30
20
f1 (ppm)

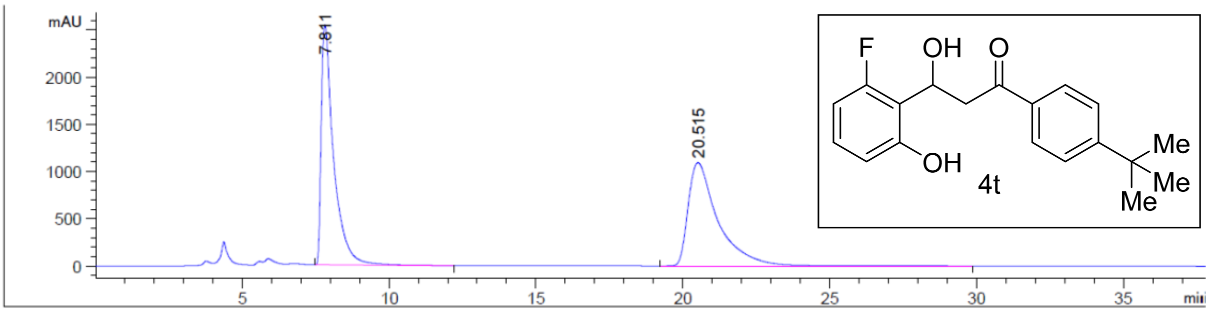

Signal 1: DAD1 A, Sig=254,4 Ref=360,100

Peak #	RetTime [min]	Type	Width [min]	Area [mAU*s]	Height [mAU]	Area %
1	7.811	BB	0.4250	7.47392e4	2542.16187	49.0547
2	20.515	BV	1.0375	7.76197e4	1094.34985	50.9453
Totals :				1.52359e5	3636.51172	

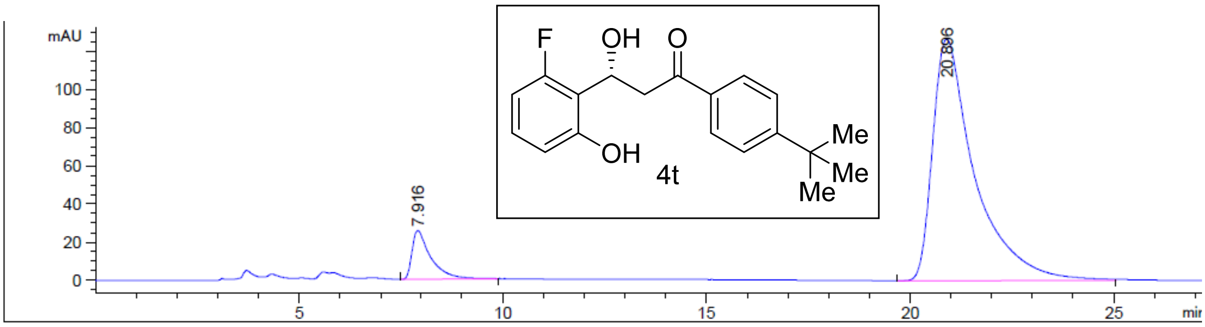

Signal 1: DAD1 A, Sig=254,4 Ref=360,100

Peak #	RetTime [min]	Type	Width [min]	Area [mAU*s]	Height [mAU]	Area %
1	7.916	BB	0.4508	814.57373	25.62724	8.2248
2	20.896	BV	1.0371	9089.28516	126.68268	91.7752
Totals :				9903.85889	152.30992	

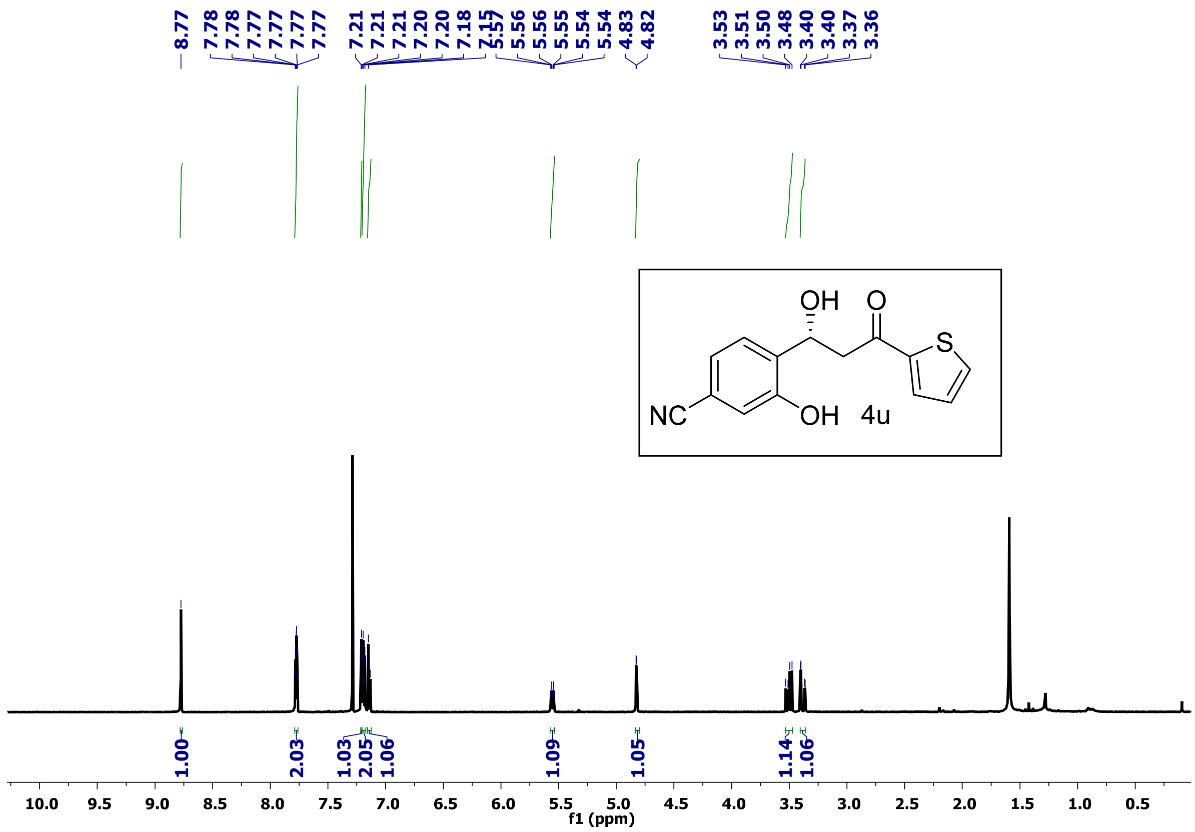
8.77
7.78 7.78 7.77 7.77 7.77 7.77
7.21 7.21 7.21 7.20 7.20 7.18 7.15
5.57 5.56 5.56 5.55 5.54 5.54
4.83 4.82
3.53 3.51 3.50 3.48 3.40 3.40 3.37 3.36
OH O
S
NC
OH 4u
1.00 2.03 1.03 2.05 1.06 1.09 1.05 1.14 1.06
10.0 9.5 9.0 8.5 8.0 7.5 7.0 6.5 6.0 5.5 5.0 4.5 4.0 3.5 3.0 2.5 2.0 1.5 1.0 0.5
f1 (ppm)

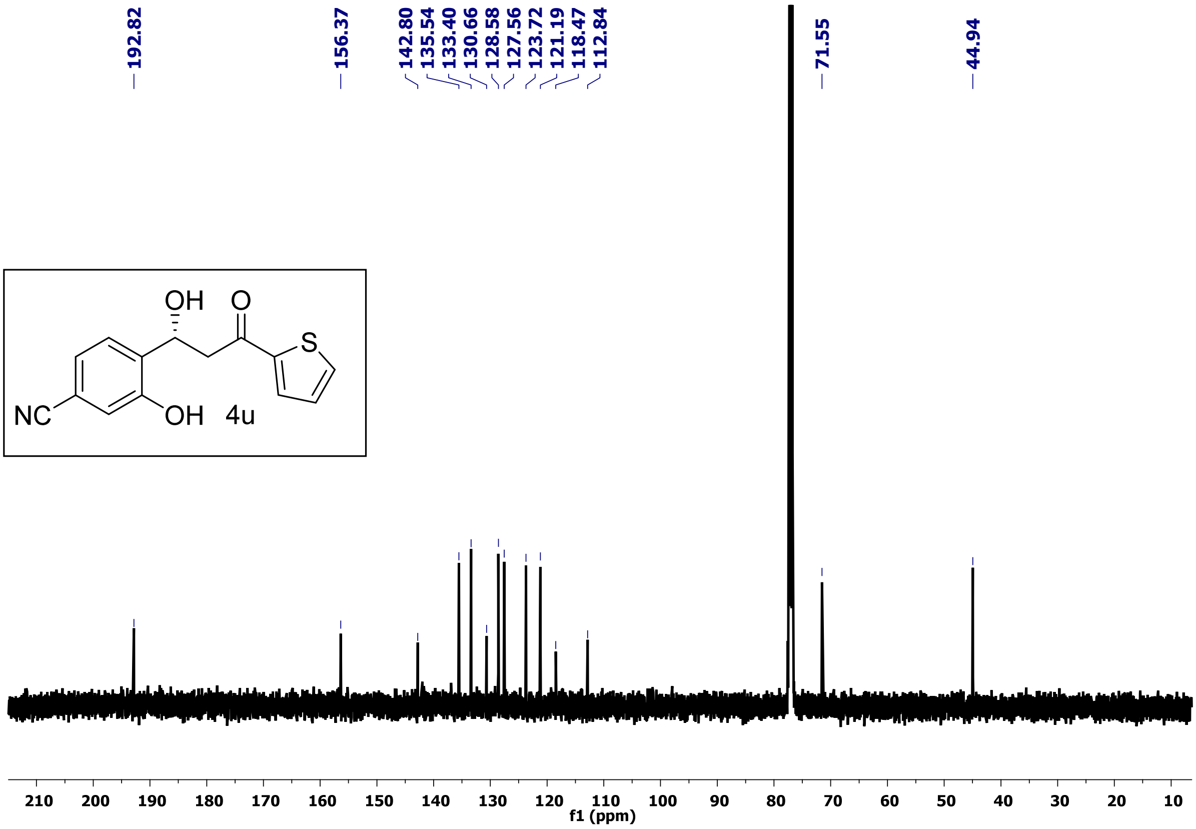
192.82
156.37
142.80 135.54 133.40 130.66 128.58 127.56 123.72 121.19 118.47 112.84
71.55
44.94
OH O
S
NC
OH 4u
210 200 190 180 170 160 150 140 130 120 110 100 90 80 70 60 50 40 30 20 10
f1 (ppm)

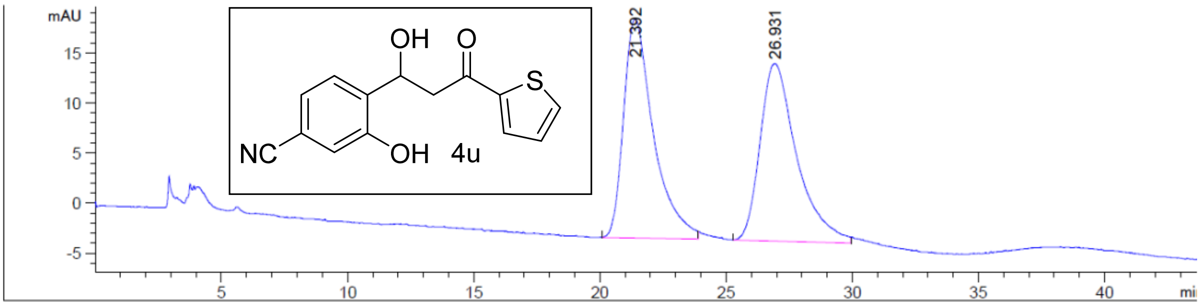

Signal 1: DAD1 A, Sig=254,4 Ref=360,100

Peak #	RetTime [min]	Type	Width [min]	Area [mAU*s]	Height [mAU]	Area %
1	21.392	VV	1.1578	1794.20068	21.80938	50.0642
2	26.931	BV	1.2563	1789.60120	17.69003	49.9358

Totals : 3583.80188 39.49941

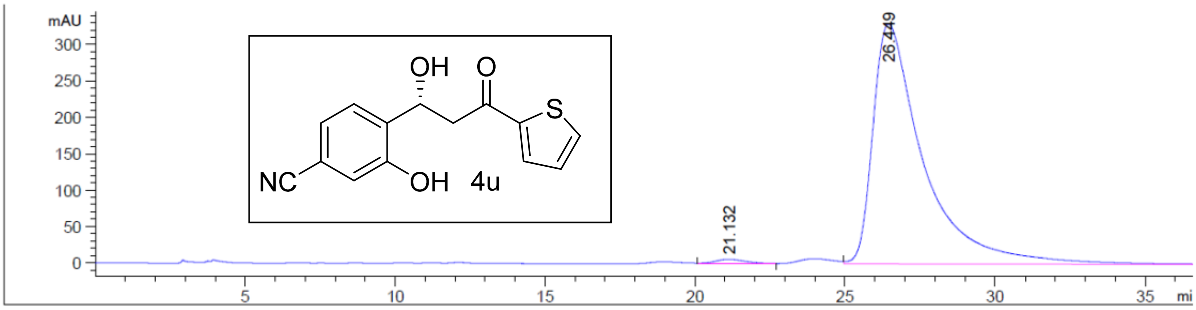

Signal 1: DAD1 A, Sig=254,4 Ref=360,100

Peak #	RetTime [min]	Type	Width [min]	Area [mAU*s]	Height [mAU]	Area %
1	21.132	VB	0.8526	435.70227	6.17973	1.1157
2	26.449	VBA	1.6925	3.86159e4	330.16058	98.8843

Totals : 3.90516e4 336.34032

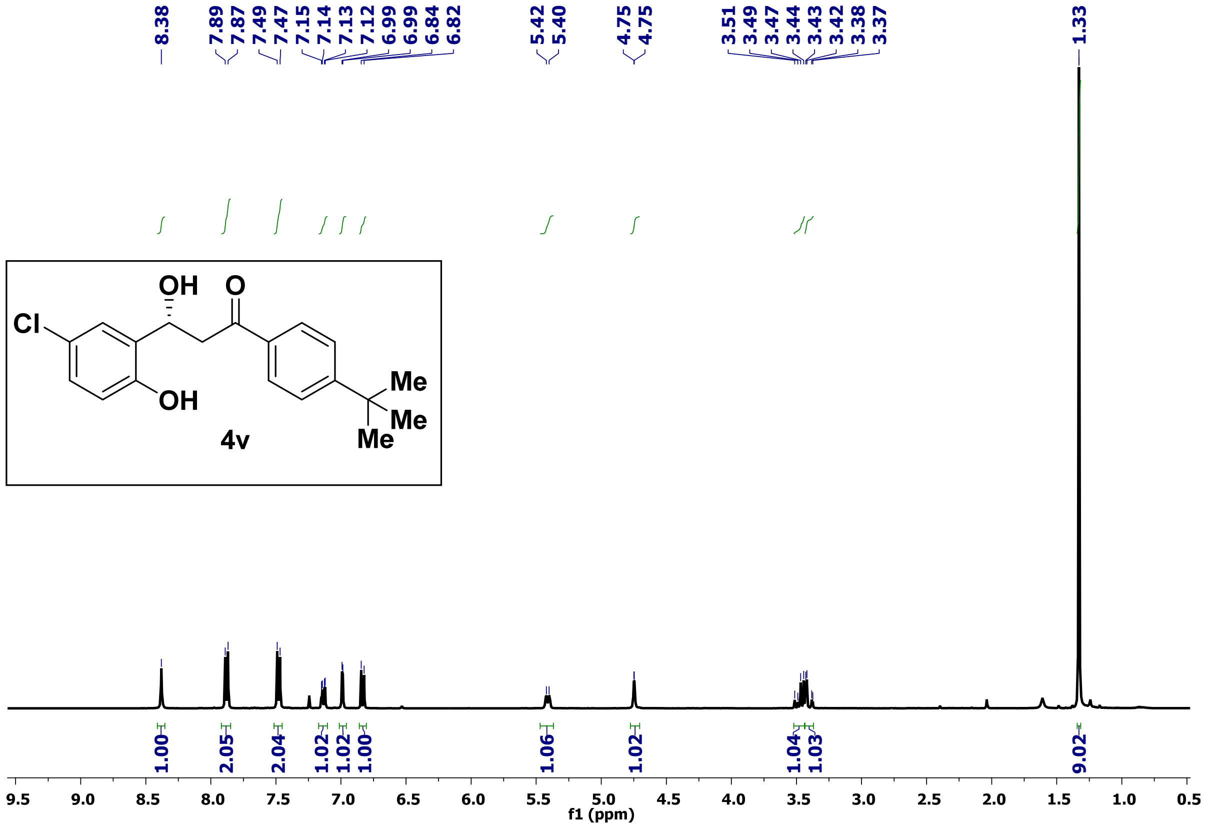
8.38
7.89
7.87
7.49
7.47
7.15
7.14
7.13
7.12
6.99
6.99
6.84
6.82
5.42
5.40
4.75
4.75
3.51
3.49
3.47
3.44
3.43
3.42
3.38
3.37
1.33
OH O
Cl
OH
Me
Me
Me
4v
1.00
2.05
2.04
1.02
1.02
1.00
1.06
1.02
1.04
1.03
9.02
9.5 9.0 8.5 8.0 7.5 7.0 6.5 6.0 5.5 5.0 4.5 4.0 3.5 3.0 2.5 2.0 1.5 1.0 0.5
f1 (ppm)

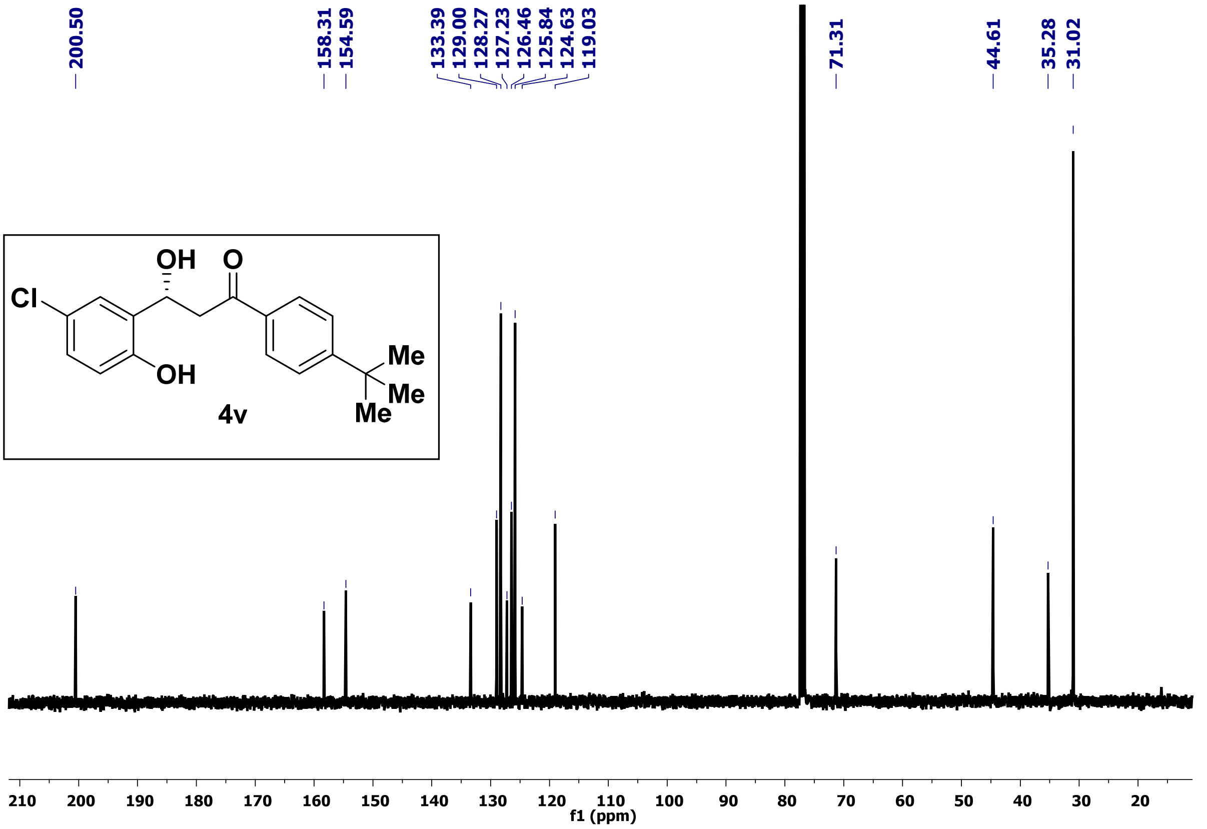
200.50
158.31
154.59
133.39
129.00
128.27
127.23
126.46
125.84
124.63
119.03
71.31
44.61
35.28
31.02
OH O
Cl
OH
Me
Me
Me
4v
210 200 190 180 170 160 150 140 130 120 110 100 90 80 70 60 50 40 30 20
f1 (ppm)

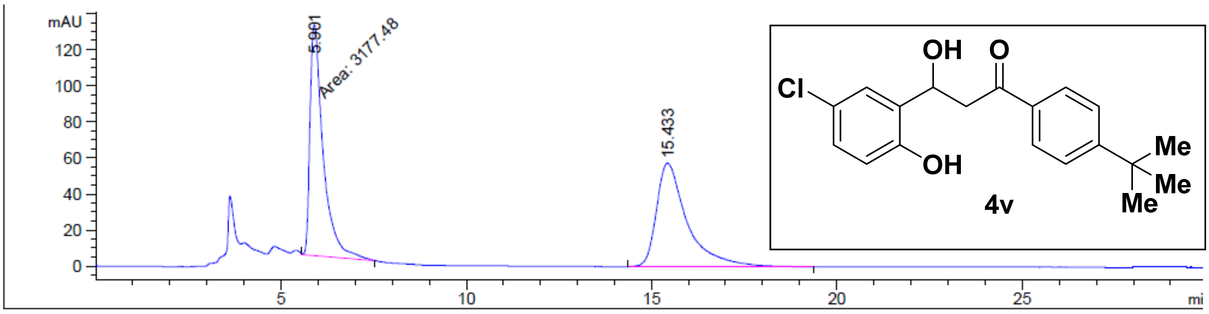

Signal 1: DAD1 A, Sig=254,4 Ref=360,100

Peak #	RetTime [min]	Type	Width [min]	Area [mAU*s]	Height [mAU]	Area %
1	5.901	MM	0.4136	3177.48242	128.05067	49.0461
2	15.433	BB	0.8455	3301.08276	57.35399	50.9539

Totals : 6478.56519 185.40467

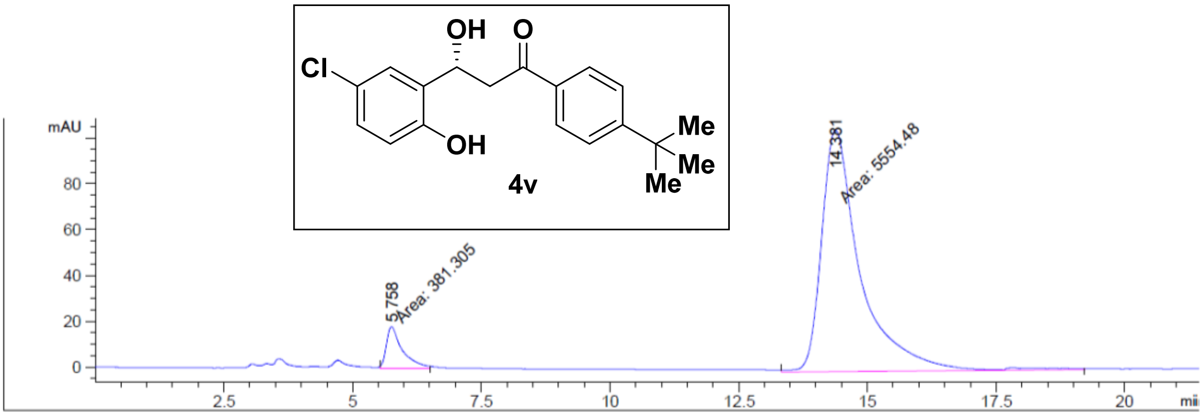

Signal 1: DAD1 A, Sig=254,4 Ref=360,100

Peak #	RetTime [min]	Type	Width [min]	Area [mAU*s]	Height [mAU]	Area %
1	5.758	MM	0.3545	381.30450	17.92626	6.4238
2	14.381	MM	0.8779	5554.47754	105.45148	93.5762

Totals : 5935.78204 123.37774

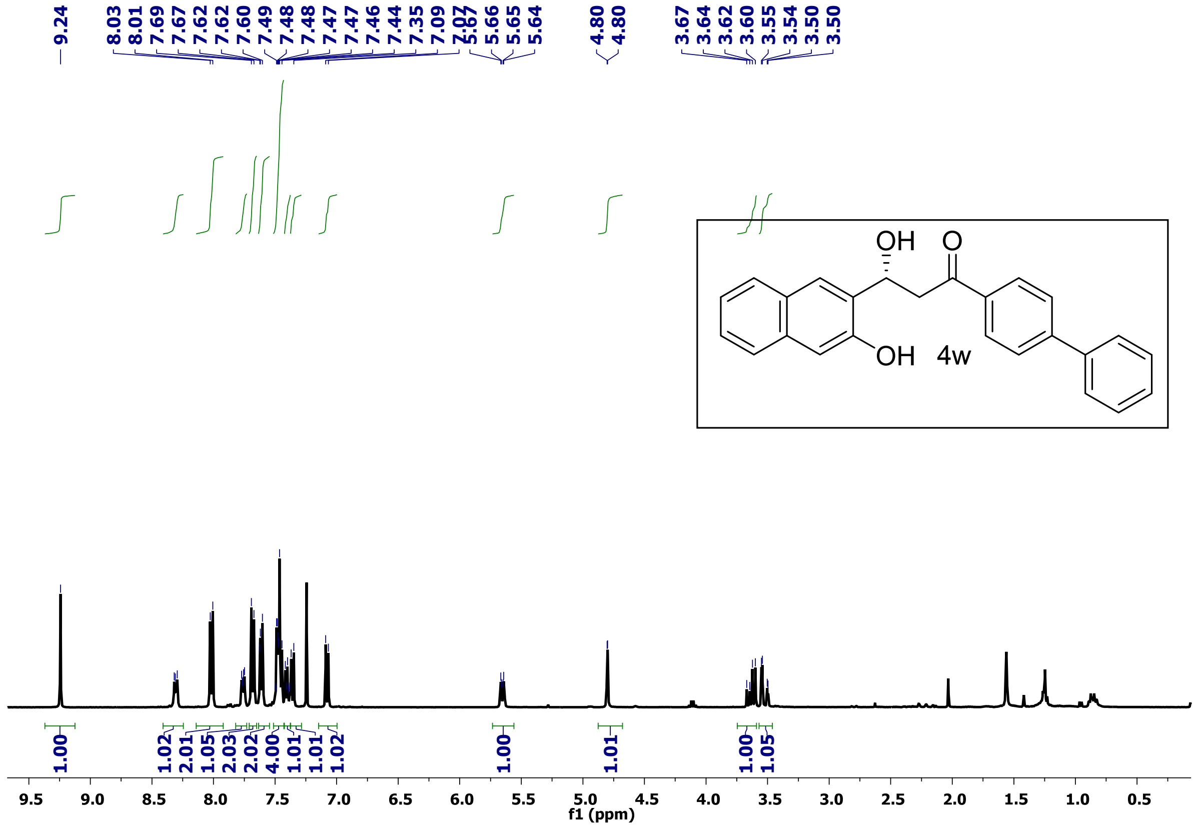
9.24
8.03 8.01 7.69 7.67 7.62 7.62 7.60 7.49 7.48 7.48 7.47 7.46 7.44 7.35 7.09 7.07 5.67 5.66 5.65 5.64
4.80 4.80
3.67 3.64 3.62 3.60 3.55 3.54 3.50 3.50
OH O
OH 4w
1.00 1.02 2.01 1.05 2.03 2.02 4.00 1.01 1.01 1.02 1.00 1.01 1.00 1.05
f1 (ppm)

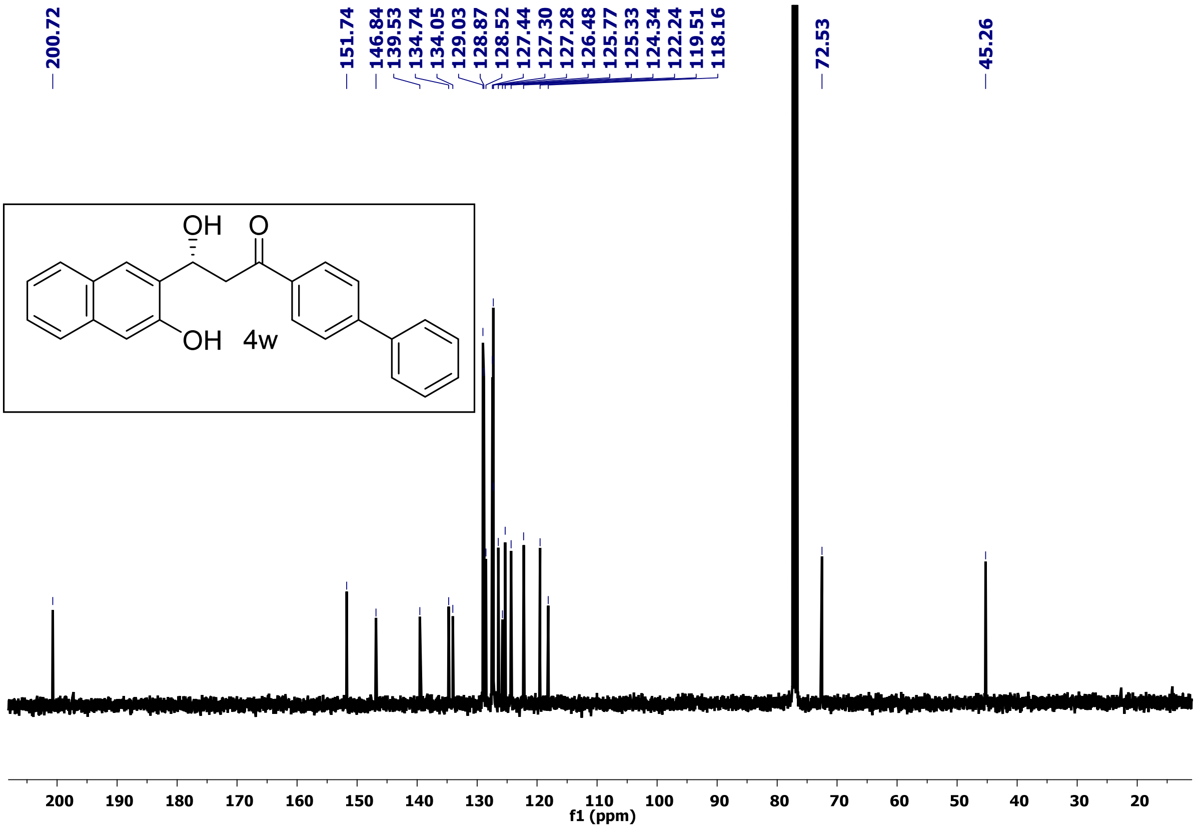
200.72
151.74 146.84 139.53 134.74 134.05 129.03 128.87 128.52 127.44 127.30 127.28 126.48 125.77 125.33 124.34 122.24 119.51 118.16
72.53
45.26
OH O
OH 4w
f1 (ppm)

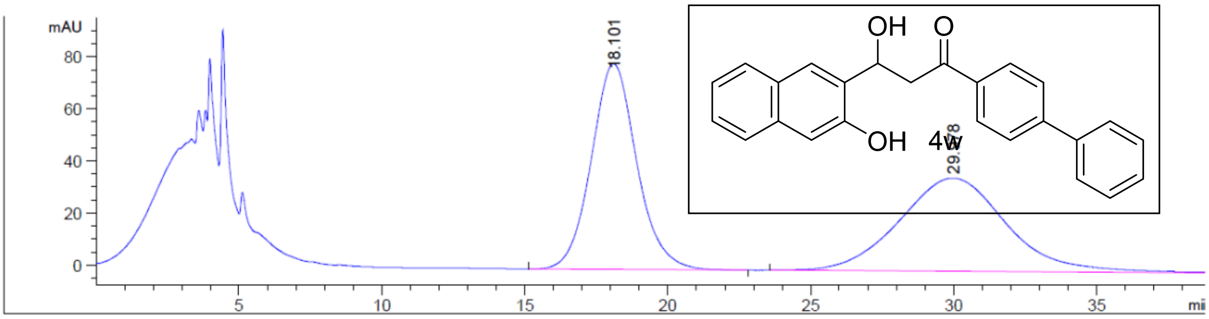

Signal 1: DAD1 A, Sig=254,4 Ref=360,100

Peak #	RetTime [min]	Type	Width [min]	Area [mAU*s]	Height [mAU]	Area %
1	18.101	BB	1.6878	8757.75781	78.96951	48.5273
2	29.978	BBA	3.0519	9289.30273	35.74474	51.4727

Totals : 1.80471e4 114.71424

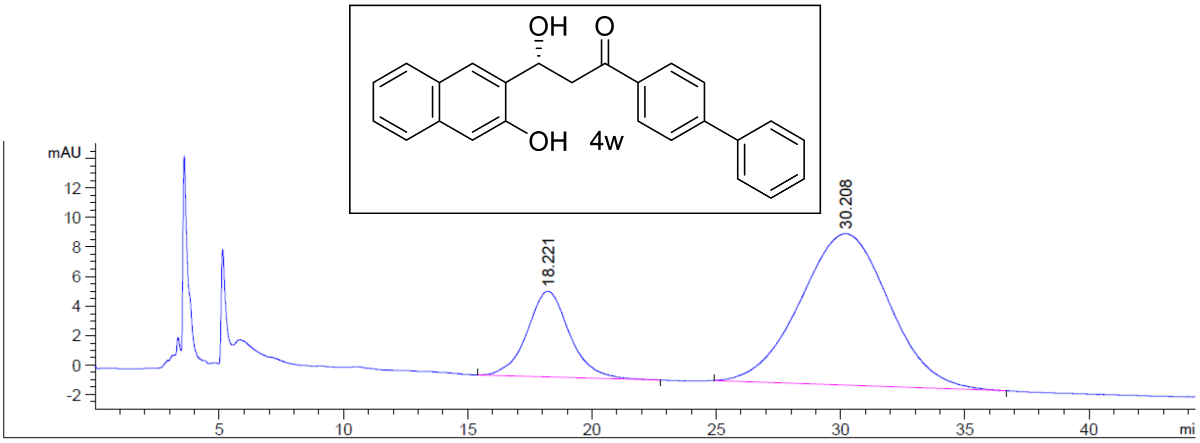

Signal 1: DAD1 A, Sig=254,4 Ref=360,100

Peak #	RetTime [min]	Type	Width [min]	Area [mAU*s]	Height [mAU]	Area %
1	18.221	BB	1.4061	692.27228	5.80157	21.2936
2	30.208	BB	2.9195	2558.80981	10.26748	78.7064

Totals : 3251.08209 16.06905

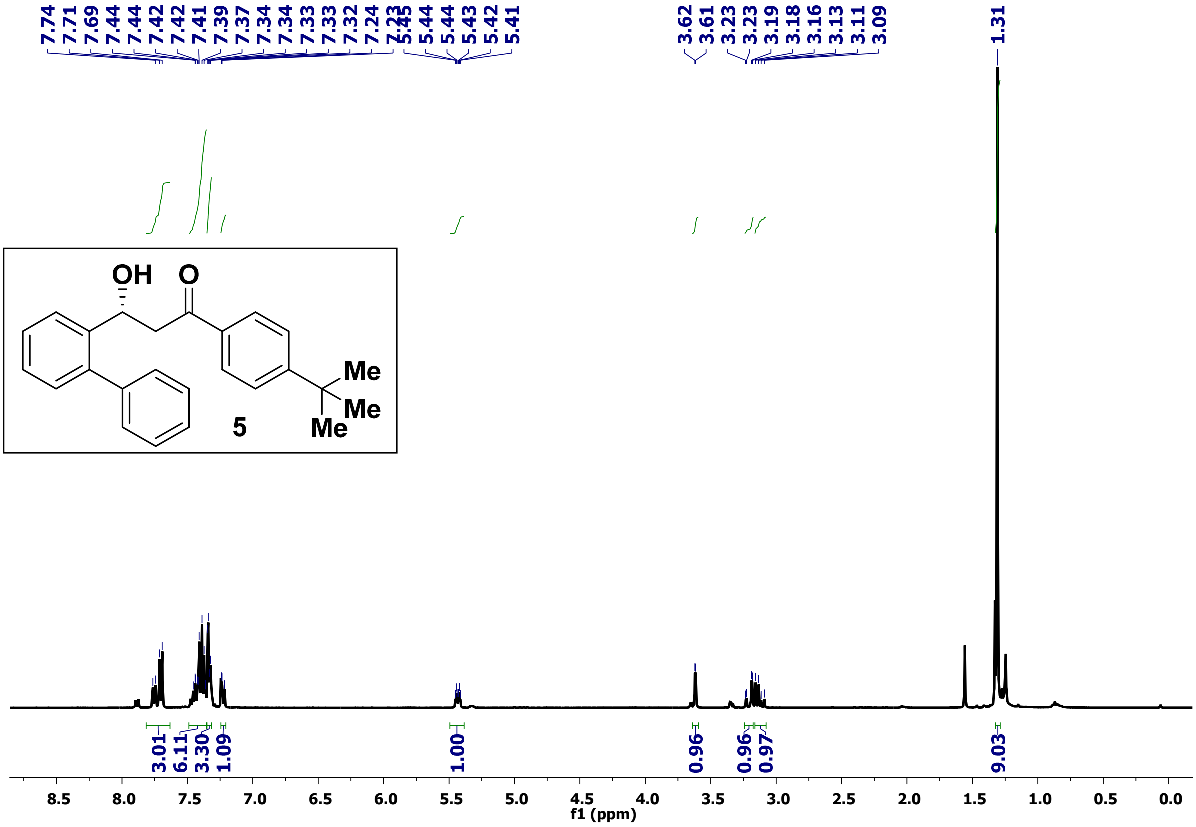
7.74 7.71 7.69 7.44 7.44 7.42 7.42 7.41 7.39 7.37 7.34 7.34 7.33 7.33 7.32 7.24 7.23 5.45 5.44 5.44 5.43 5.42 5.41
3.62 3.61 3.23 3.23 3.19 3.18 3.16 3.13 3.11 3.09
1.31
OH O
Me
Me
Me
5
3.01 6.11 3.30 1.09 1.00 0.96 0.96 0.97 9.03
8.5 8.0 7.5 7.0 6.5 6.0 5.5 5.0 4.5 4.0 3.5 3.0 2.5 2.0 1.5 1.0 0.5 0.0
f1 (ppm)

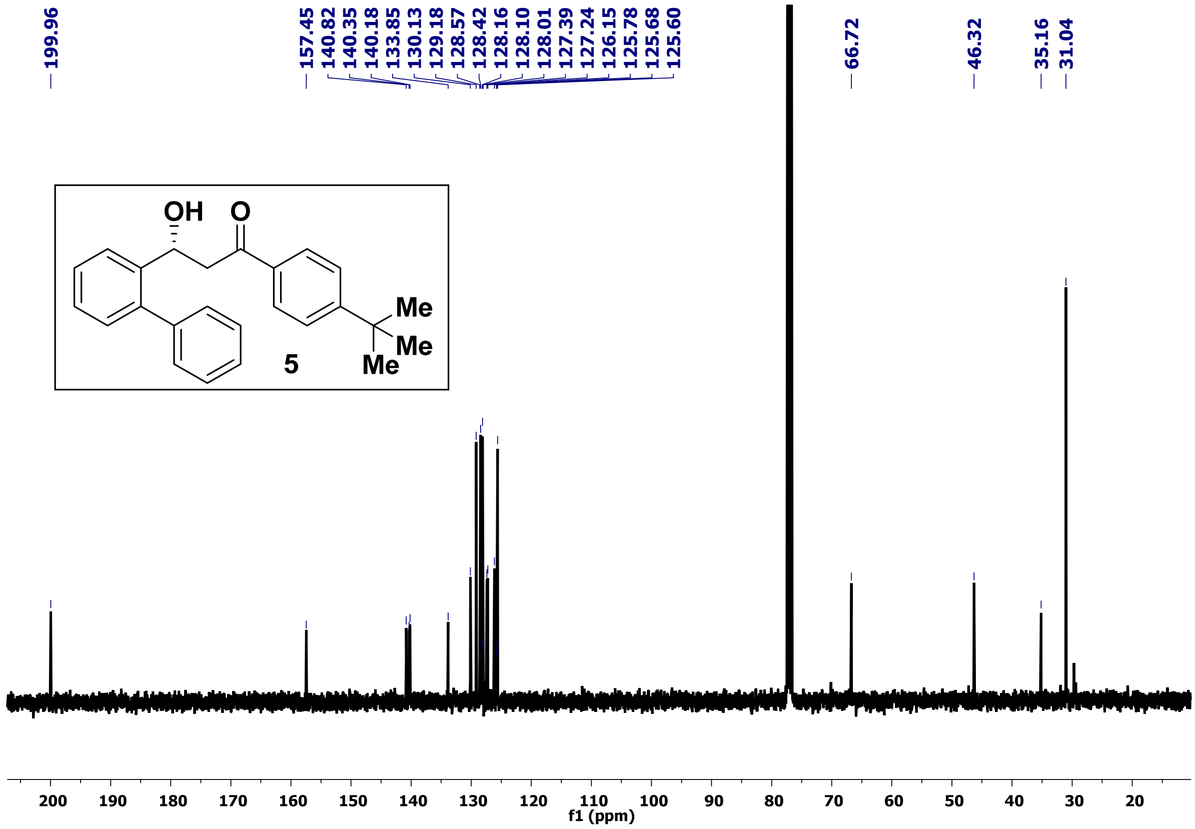
199.96
157.45 140.82 140.35 140.18 133.85 130.13 129.18 128.57 128.42 128.16 128.10 128.01 127.39 127.24 126.15 125.78 125.68 125.60
66.72
46.32
35.16 31.04
OH O
Me
Me
Me
5
200 190 180 170 160 150 140 130 120 110 100 90 80 70 60 50 40 30 20
f1 (ppm)

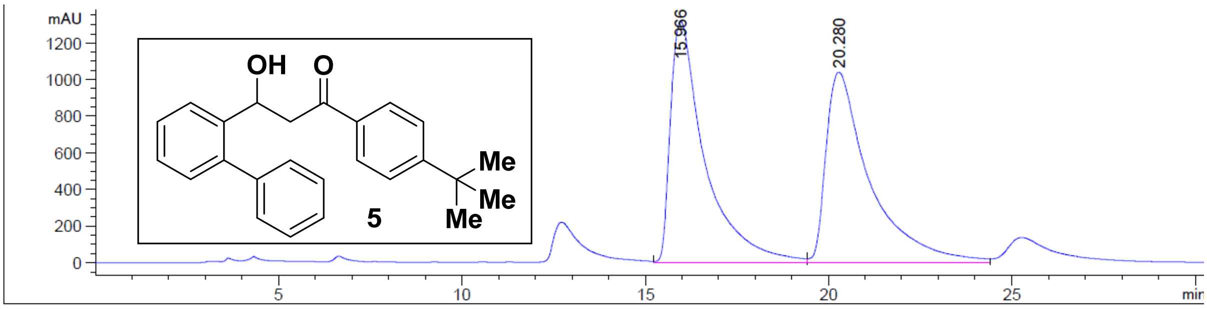

Signal 1: DAD1 A, Sig=254,4 Ref=360,100

Peak #	RetTime [min]	Type	Width [min]	Area [mAU*s]	Height [mAU]	Area %
1	15.966	VV	0.9465	8.59525e4	1315.07922	49.9041
2	20.280	VV	1.1960	8.62828e4	1037.81763	50.0959

Totals : 1.72235e5 2352.89685

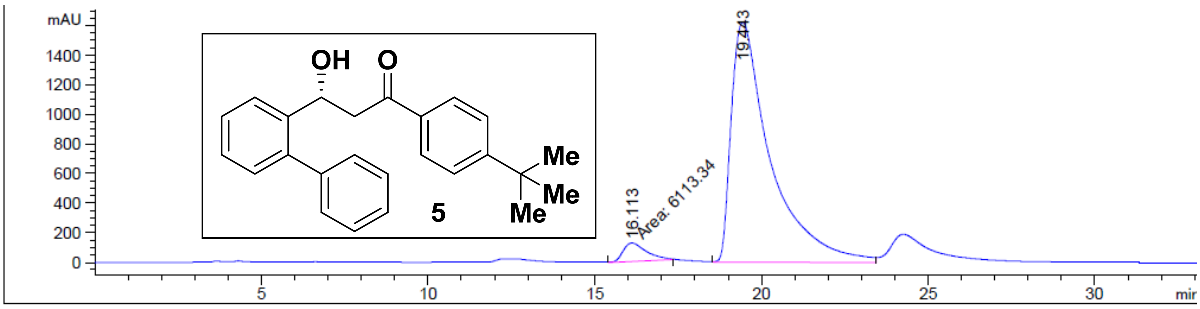

Signal 1: DAD1 A, Sig=254,4 Ref=360,100

Peak #	RetTime [min]	Type	Width [min]	Area [mAU*s]	Height [mAU]	Area %
1	16.113	MM	0.8123	6113.34473	125.44003	4.4026
2	19.443	VV	1.1862	1.32745e5	1623.05896	95.5974

Totals : 1.38858e5 1748.49899

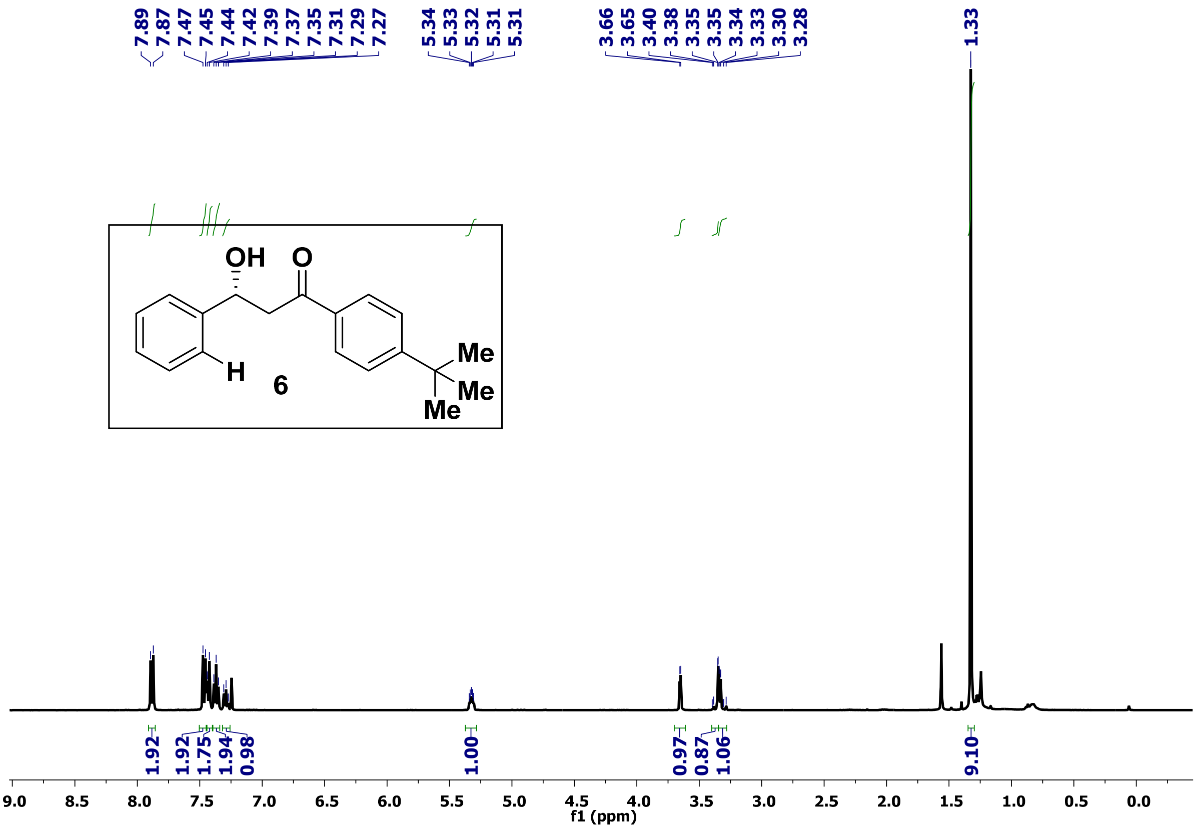
7.89 7.87 7.47 7.45 7.44 7.42 7.39 7.37 7.35 7.31 7.29 7.27
5.34 5.33 5.32 5.31 5.31
3.66 3.65 3.40 3.38 3.35 3.35 3.34 3.33 3.30 3.28
1.33
OH
O
H
6
Me
Me
Me
1.92
1.92
1.75
1.94
0.98
1.00
0.97
0.87
1.06
9.10
f1 (ppm)

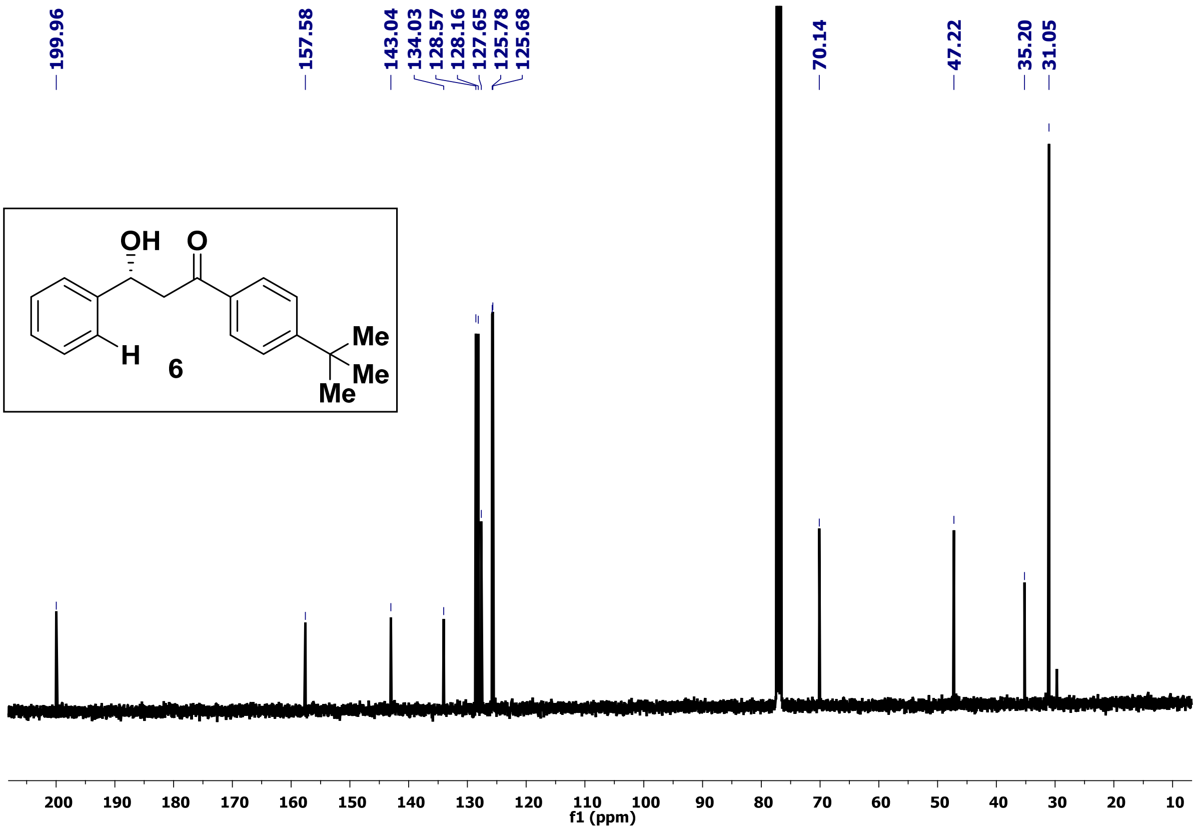
199.96
157.58
143.04 134.03 128.57 128.16 127.65 125.78 125.68
70.14
47.22
35.20 31.05
OH
O
H
6
Me
Me
Me
f1 (ppm)

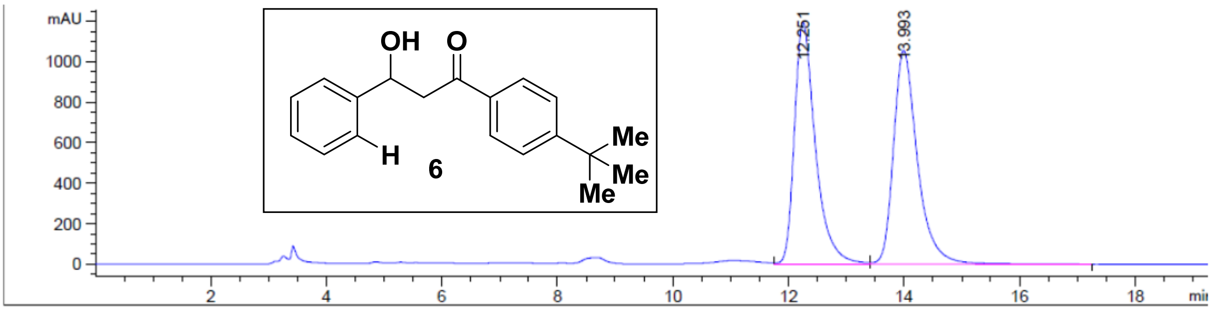

Signal 1: DAD1 A, Sig=254,4 Ref=360,100

Peak #	RetTime [min]	Type	Width [min]	Area [mAU*s]	Height [mAU]	Area %
1	12.251	VV	0.3809	3.02252e4	1195.86609	49.7629
2	13.993	VB	0.4373	3.05132e4	1055.43823	50.2371

Totals : 6.07384e4 2251.30432

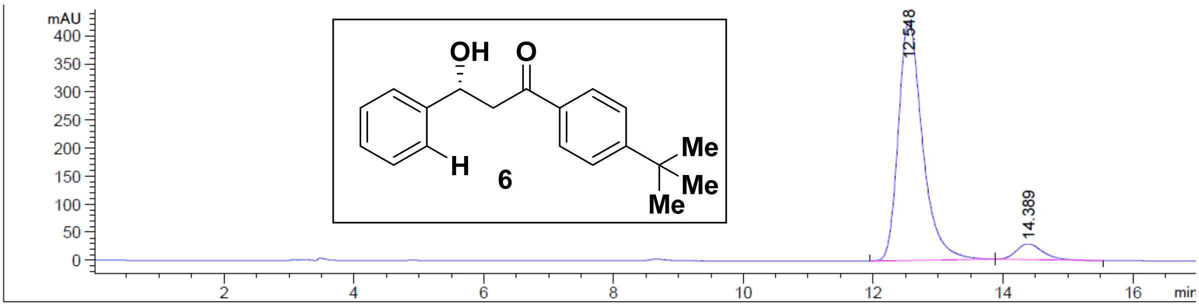

Signal 1: DAD1 A, Sig=254,4 Ref=360,100

Peak #	RetTime [min]	Type	Width [min]	Area [mAU*s]	Height [mAU]	Area %
1	12.548	BB	0.3916	1.10239e4	426.44751	93.1600
2	14.389	BB	0.4360	809.39734	28.44197	6.8400

Totals : 1.18333e4 454.88948

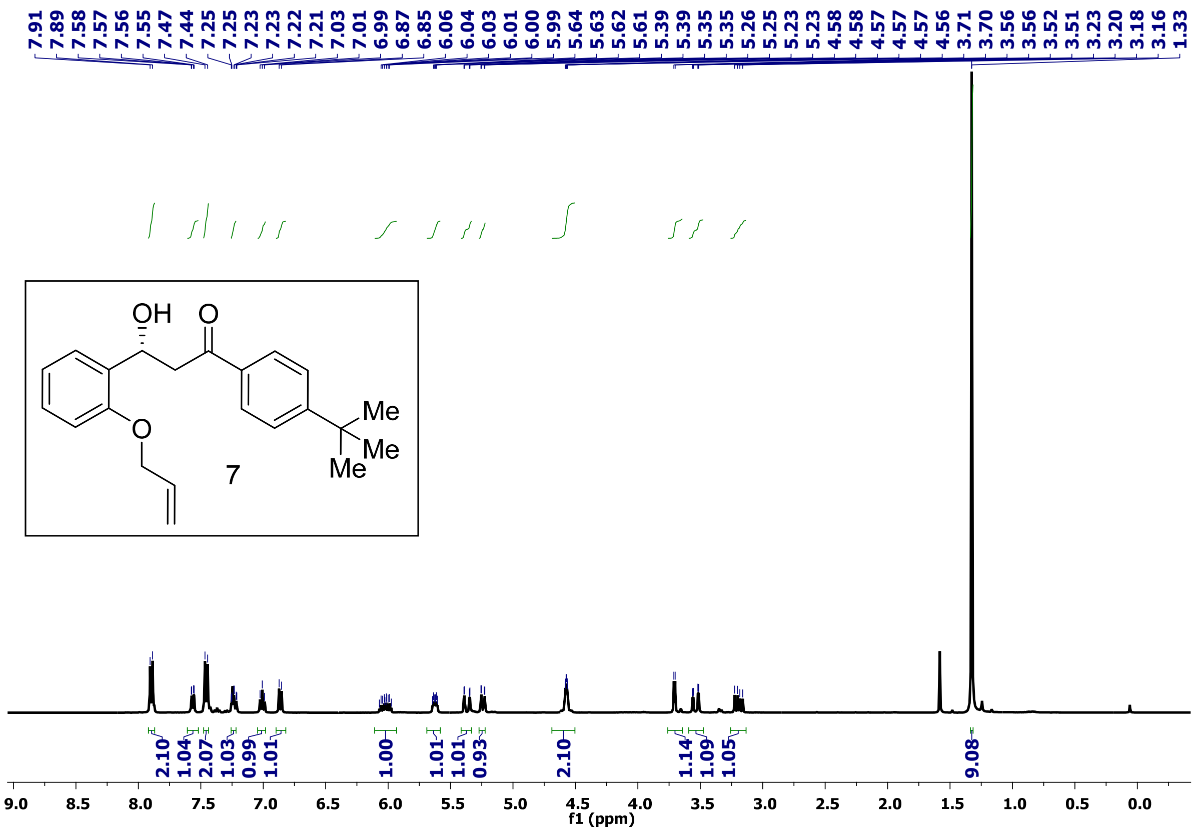
7.91 7.89 7.58 7.57 7.56 7.55 7.47 7.44 7.25 7.25 7.23 7.23 7.22 7.21 7.03 7.01 6.99 6.87 6.85 6.06 6.04 6.03 6.01 6.00 5.99 5.64 5.63 5.62 5.61 5.39 5.39 5.35 5.35 5.26 5.25 5.23 5.23 4.58 4.58 4.57 4.57 4.57 4.56 3.71 3.70 3.56 3.56 3.52 3.51 3.23 3.20 3.18 3.16 1.33
OH
O
O
Me
Me
Me
7
2.10 1.04 2.07 1.03 0.99 1.01 1.00 1.01 1.01 0.93 2.10 1.14 1.09 1.05 9.08
f1 (ppm)

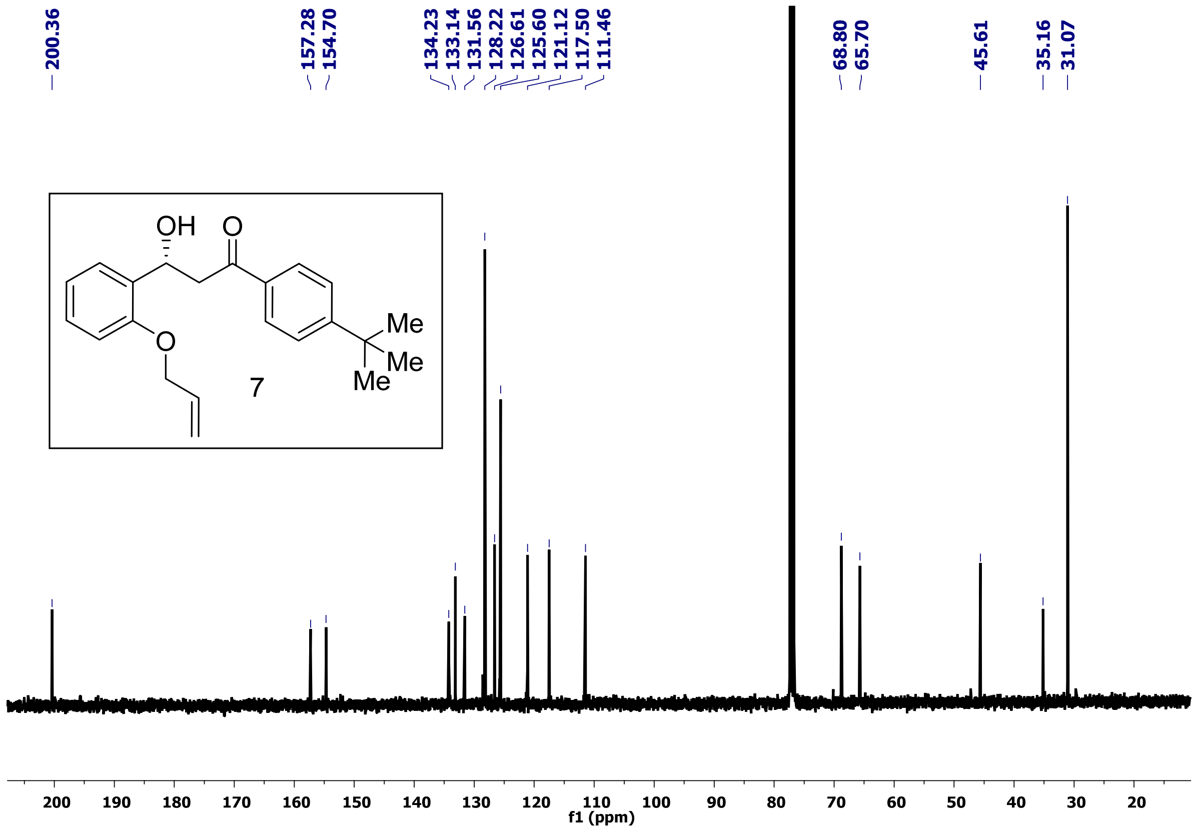
200.36 157.28 154.70 134.23 133.14 131.56 128.22 126.61 125.60 121.12 117.50 111.46 68.80 65.70 45.61 35.16 31.07
OH
O
O
Me
Me
Me
7
f1 (ppm)

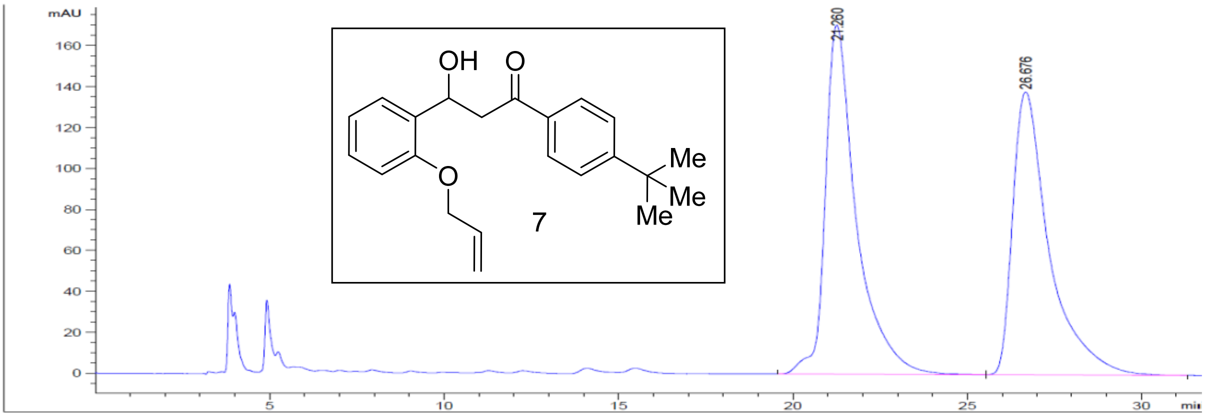

Signal 1: DAD1 A, Sig=254,4 Ref=360,100

Peak #	RetTime [min]	Type	Width [min]	Area [mAU*s]	Height [mAU]	Area %
1	21.260	BB	0.9136	1.05754e4	170.51503	51.3422
2	26.676	BB	1.0842	1.00225e4	138.17734	48.6578

Totals : 2.05978e4 308.69237

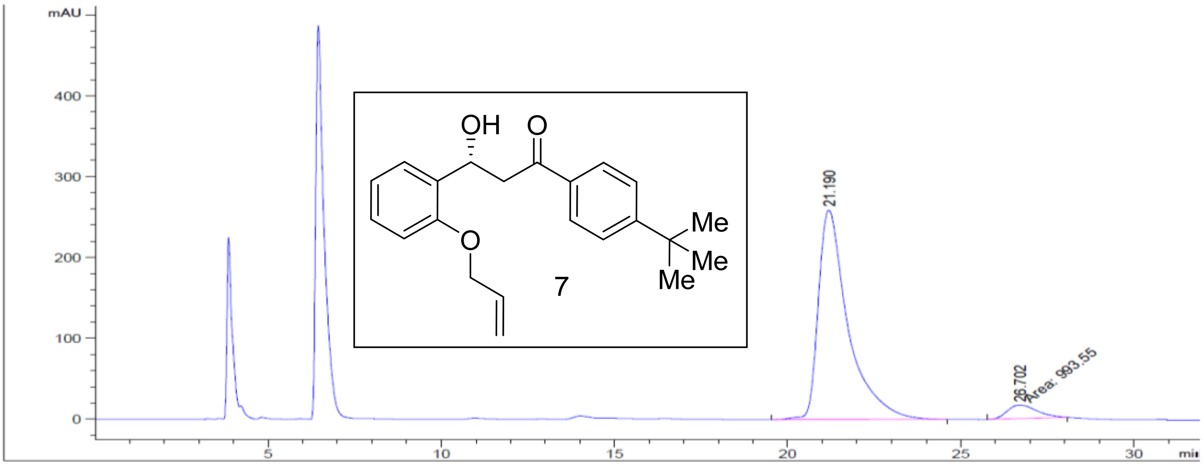

Signal 1: DAD1 A, Sig=254,4 Ref=360,100

Peak #	RetTime [min]	Type	Width [min]	Area [mAU*s]	Height [mAU]	Area %
1	21.190	BB	0.8802	1.54516e4	258.93024	93.9584
2	26.702	MM	0.9817	993.54987	16.86861	6.0416

Totals : 1.64451e4 275.79885

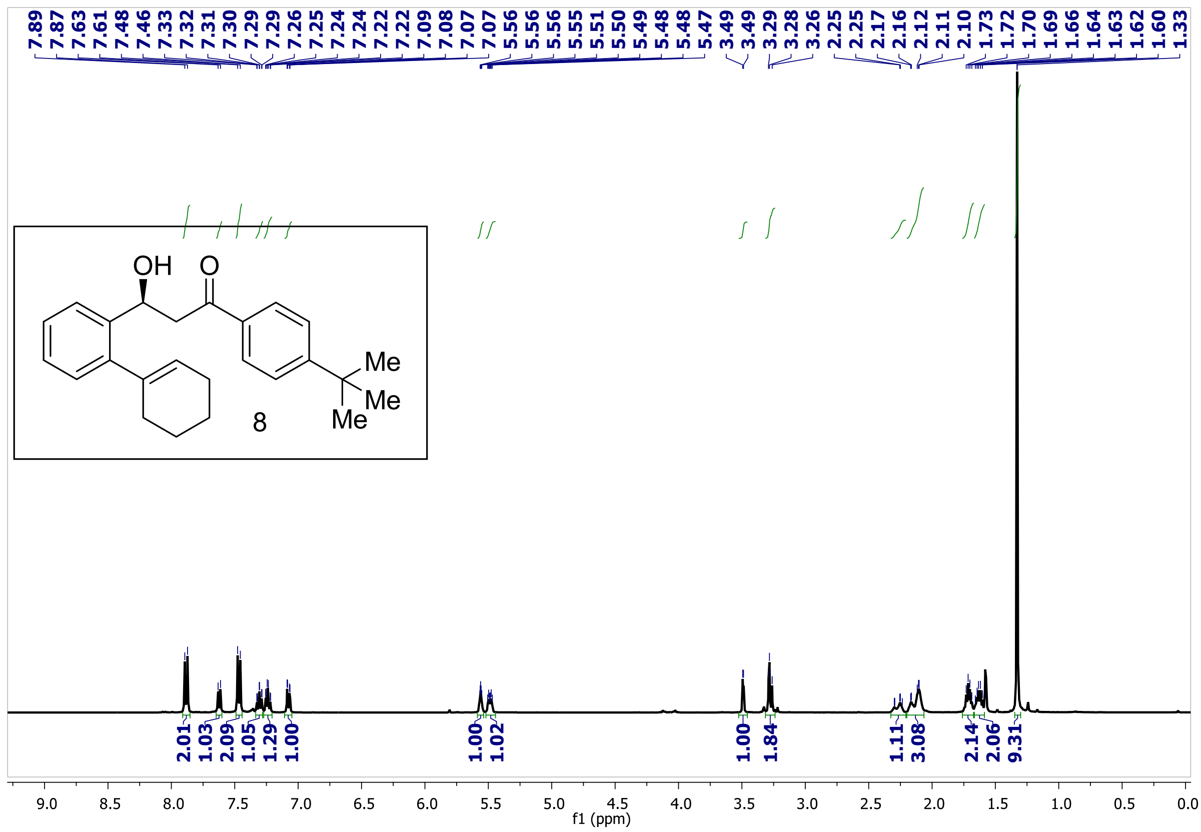
7.89 7.87 7.63 7.61 7.48 7.46 7.33 7.32 7.31 7.30 7.29 7.29 7.26 7.25 7.24 7.24 7.22 7.22 7.09 7.08 7.07 7.07 5.56 5.56 5.55 5.51 5.50 5.49 5.48 5.48 5.47 3.49 3.49 3.29 3.28 3.26 2.25 2.25 2.17 2.16 2.12 2.11 2.10 1.73 1.72 1.70 1.69 1.66 1.64 1.63 1.62 1.60 1.33
OH
O
Me
Me
Me
8
2.01 1.03 2.09 1.05 1.29 1.00 1.00 1.02 1.00 1.84 1.11 3.08 2.14 2.06 9.31
f1 (ppm)

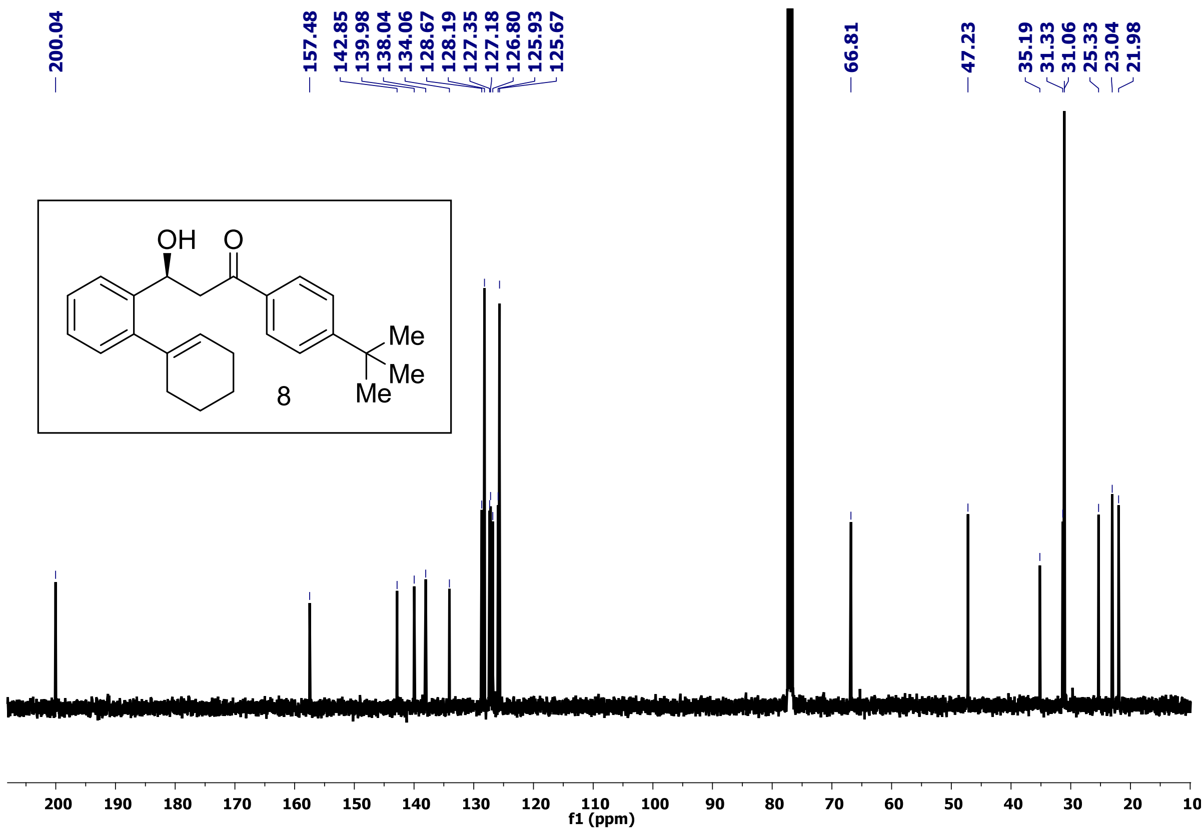
200.04 157.48 142.85 139.98 138.04 134.06 128.67 128.19 127.35 127.18 126.80 125.93 125.67 66.81 47.23 35.19 31.33 31.06 25.33 23.04 21.98
OH
O
Me
Me
Me
8
f1 (ppm)

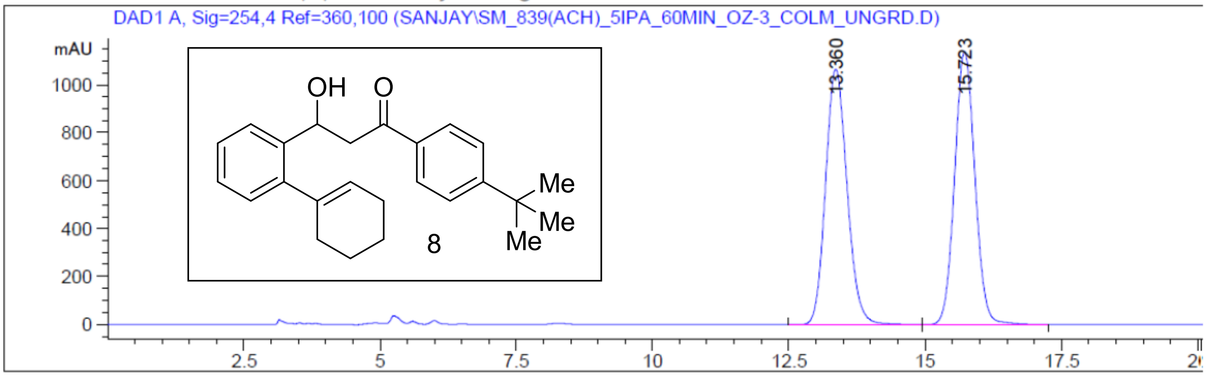

Signal 1: DAD1 A, Sig=254,4 Ref=360,100

Peak #	RetTime [min]	Type	Width [min]	Area [mAU*s]	Height [mAU]	Area %
1	13.360	BB	0.4269	2.93111e4	1065.49939	49.8828
2	15.723	BB	0.4039	2.94489e4	1137.84216	50.1172

Totals : 5.87600e4 2203.34155

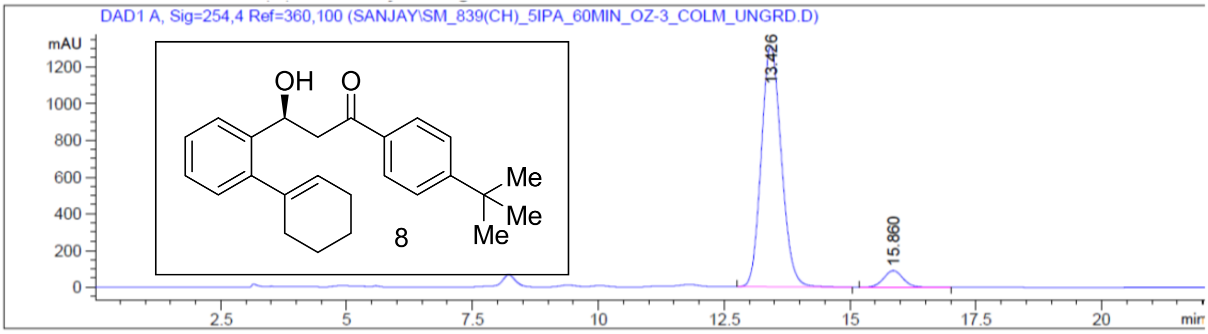

Signal 1: DAD1 A, Sig=254,4 Ref=360,100

Peak #	RetTime [min]	Type	Width [min]	Area [mAU*s]	Height [mAU]	Area %
1	13.426	BB	0.4329	3.62579e4	1310.12268	93.7282
2	15.860	BB	0.4146	2426.17603	90.55199	6.2718

Totals : 3.86841e4 1400.67467

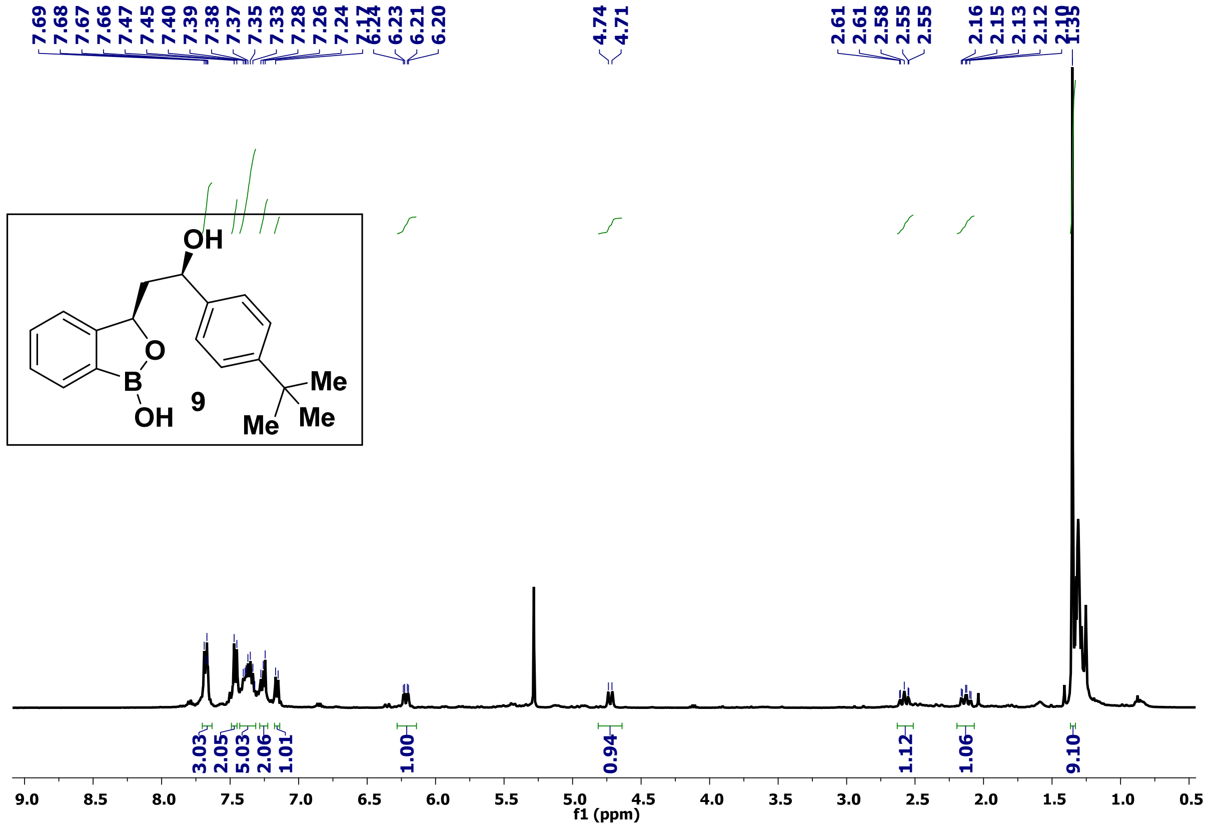

7.69 7.68 7.67 7.66 7.47 7.45 7.40 7.39 7.38 7.37 7.35 7.33 7.28 7.26 7.24 7.17 6.24 6.23 6.21 6.20
4.74 4.71
2.61 2.61 2.58 2.55 2.55 2.16 2.15 2.13 2.12 2.10 1.35
OH
O
B
OH
9
Me
Me
Me
3.03 2.05 5.03 2.06 1.01 1.00 0.94 1.12 1.06 9.10
9.0 8.5 8.0 7.5 7.0 6.5 6.0 5.5 5.0 4.5 4.0 3.5 3.0 2.5 2.0 1.5 1.0 0.5
f1 (ppm)

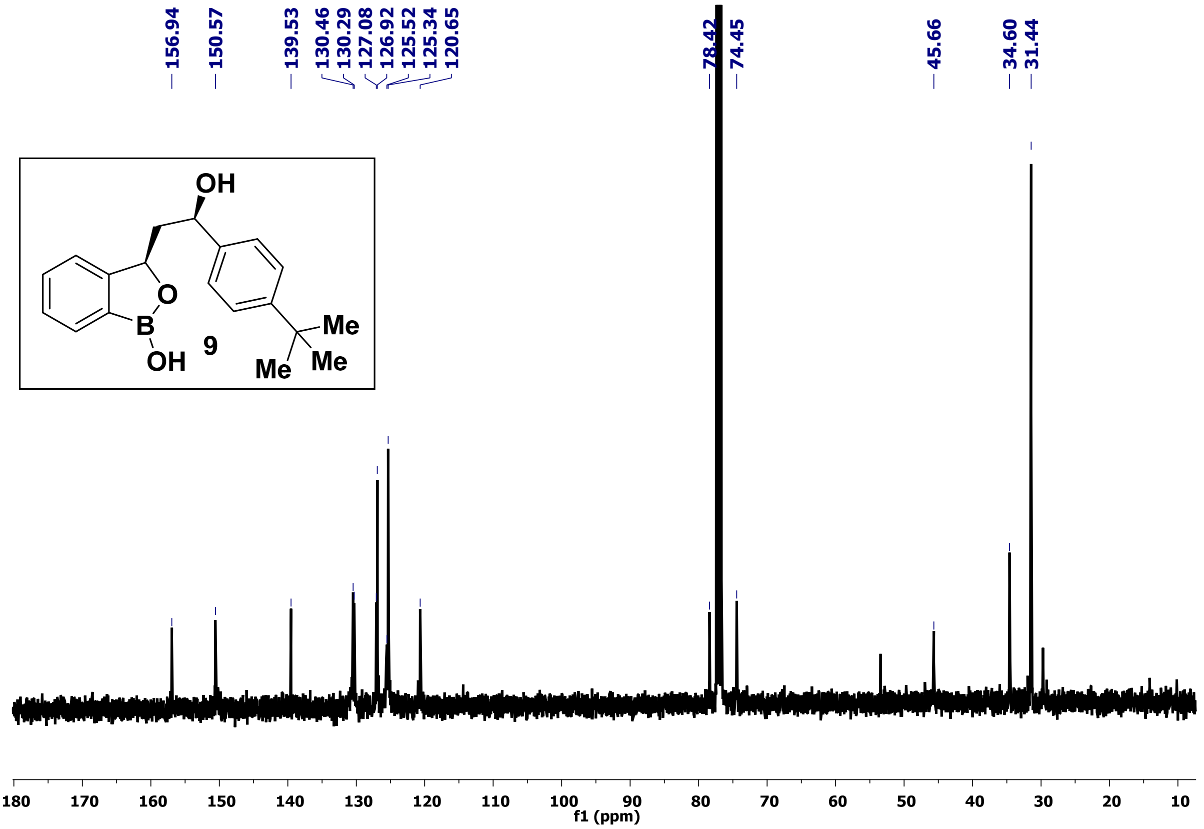

156.94
150.57
139.53
130.46 130.29 127.08 126.92 125.52 125.34 120.65
78.42
74.45
45.66
34.60
31.44
OH
O
B
OH
9
Me
Me
Me
180 170 160 150 140 130 120 110 100 90 80 70 60 50 40 30 20 10
f1 (ppm)

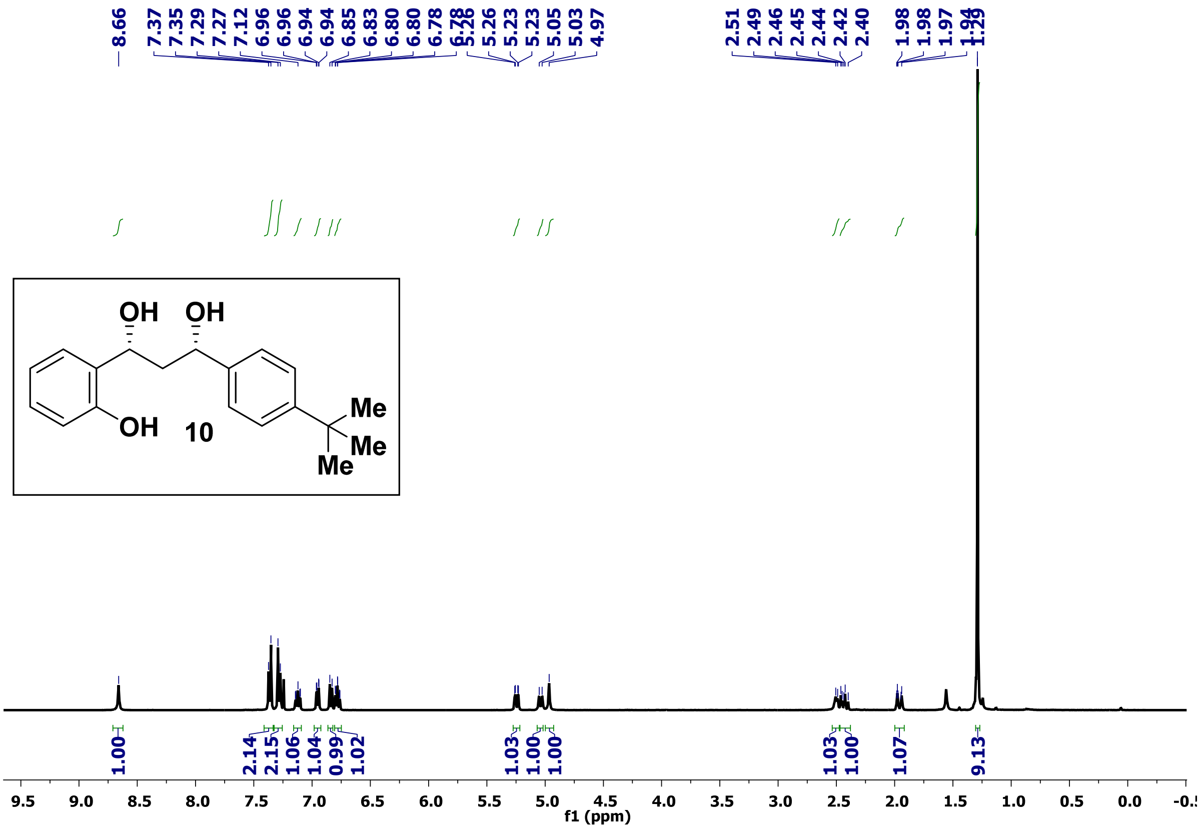
8.66
7.37 7.35 7.29 7.27 7.12 6.96 6.96 6.94 6.94 6.85 6.83 6.80 6.80 6.78 6.78 5.26 5.26 5.23 5.23 5.05 5.03 4.97
2.51 2.49 2.46 2.45 2.44 2.42 2.40
1.98 1.98 1.97 1.94 1.29
OH OH
OH 10
Me
Me
Me
1.00 2.14 2.15 1.06 1.04 0.99 1.02 1.03 1.00 1.00 1.03 1.00 1.07 9.13
f1 (ppm)

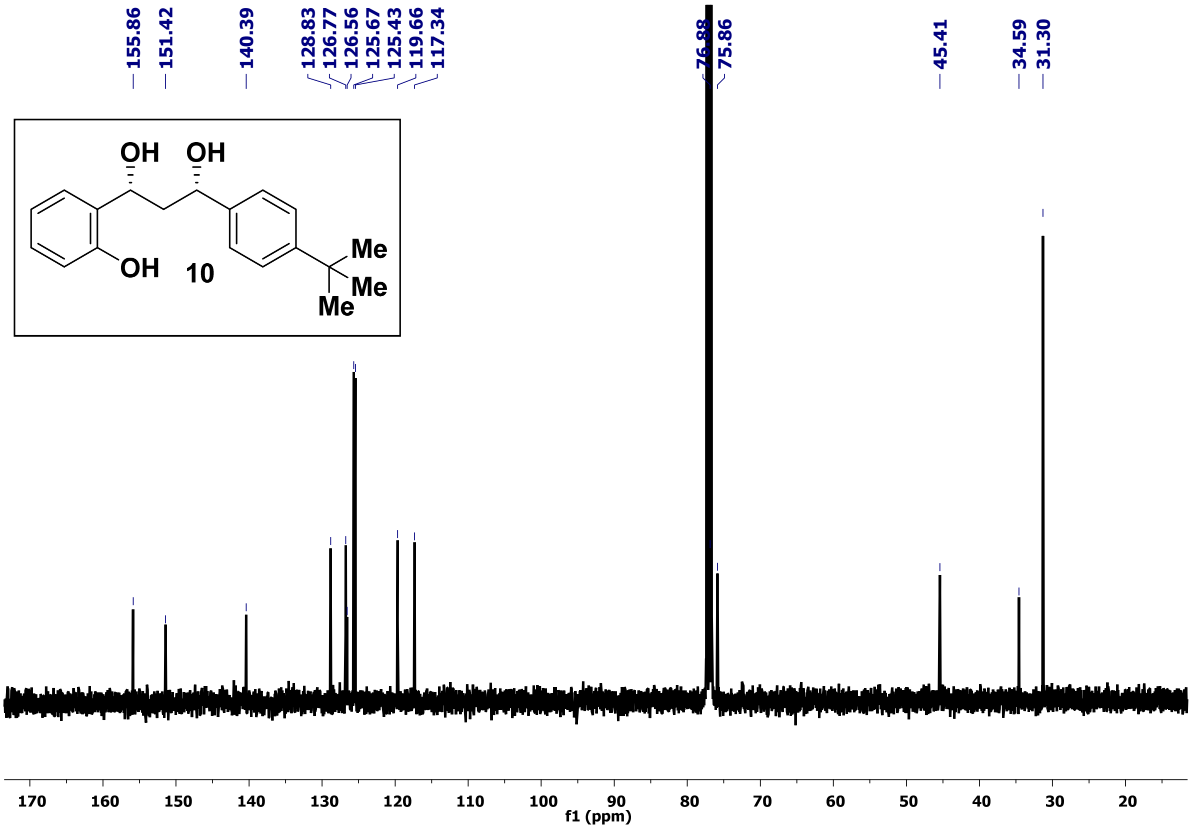
155.86 151.42 140.39 128.83 126.77 126.56 125.67 125.43 119.66 117.34 76.88 75.86 45.41 34.59 31.30
OH OH
OH 10
Me
Me
Me
f1 (ppm)

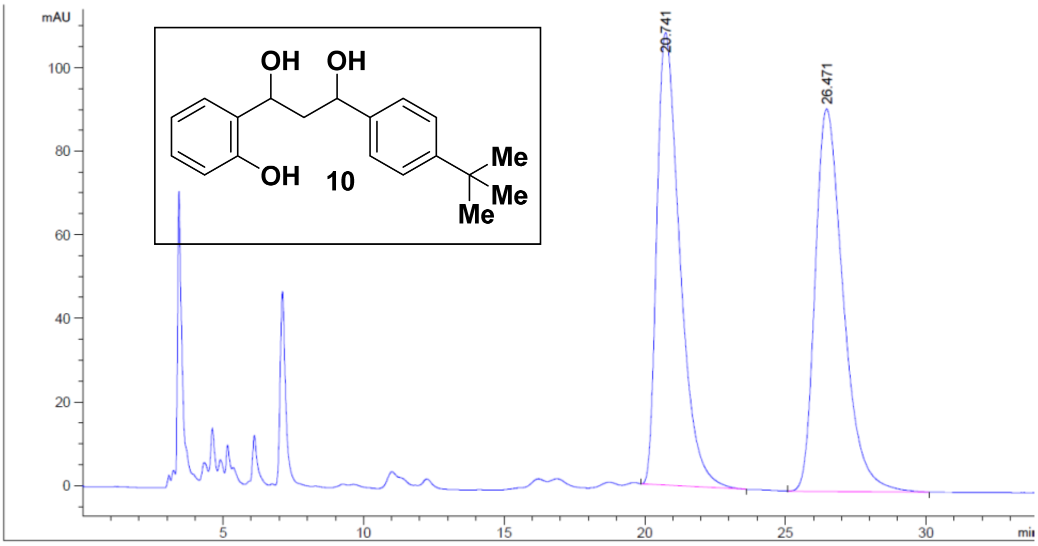

Signal 1: DAD1 A, Sig=254,4 Ref=360,100

Peak #	RetTime [min]	Type	Width [min]	Area [mAU*s]	Height [mAU]	Area %
1	20.741	BB	0.8641	6141.43262	108.21538	49.1806
2	26.471	BB	1.0537	6346.08057	91.43807	50.8194

Totals :	1.24875e4	199.65345

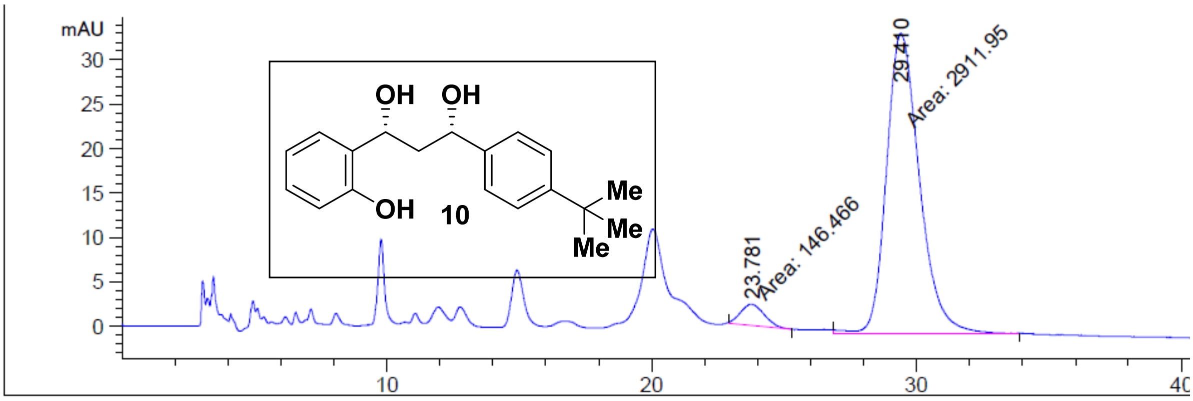

Signal 1: DAD1 A, Sig=254,4 Ref=360,100

Peak #	RetTime [min]	Type	Width [min]	Area [mAU*s]	Height [mAU]	Area %
1	23.781	MM	1.0332	146.46625	2.36271	4.7890
2	29.410	MM	1.4366	2911.94922	33.78216	95.2110

Totals :	3058.41547	36.14487

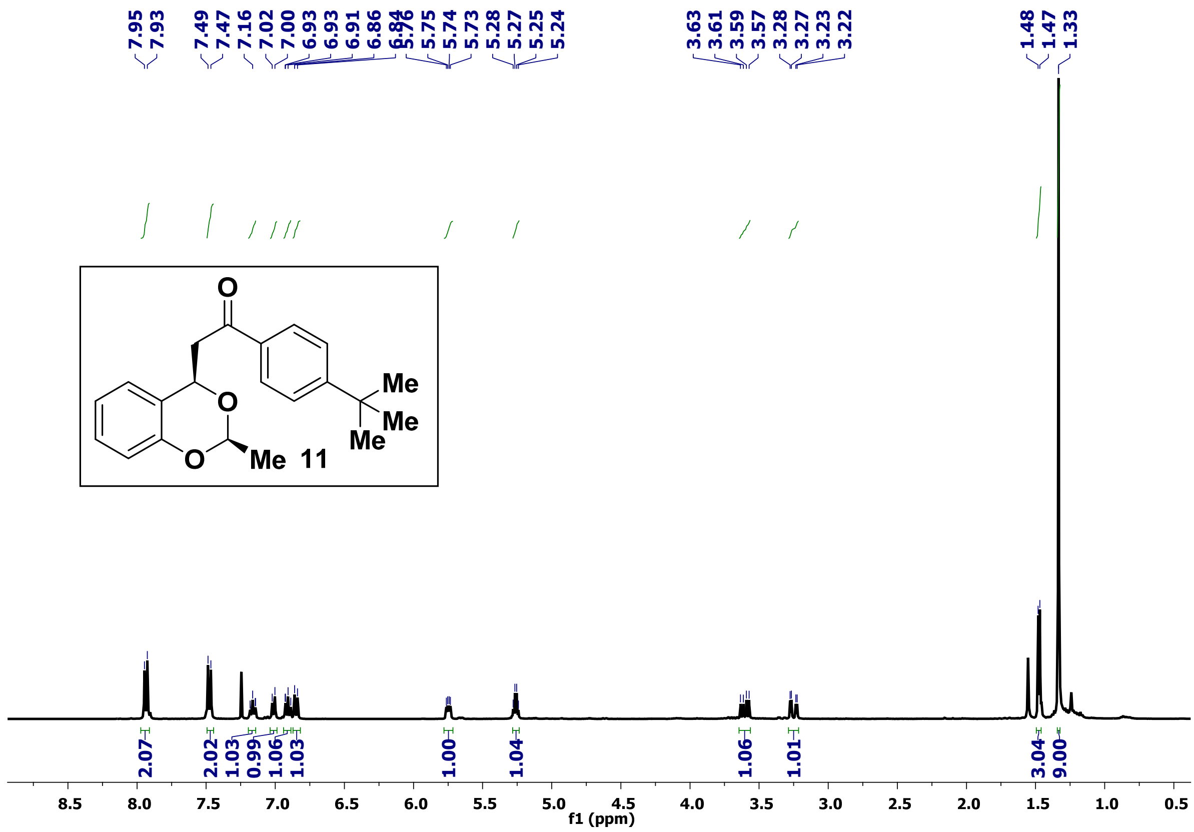
7.95
7.93
7.49
7.47
7.16
7.02
7.00
6.93
6.93
6.91
6.86
6.84
5.76
5.75
5.74
5.73
5.28
5.27
5.25
5.24
3.63
3.61
3.59
3.57
3.28
3.27
3.23
3.22
1.48
1.47
1.33
O
Me
Me
Me
O
O
Me
11
2.07
2.02
1.03
0.99
1.06
1.03
1.00
1.04
1.06
1.01
3.04
9.00
f1 (ppm)

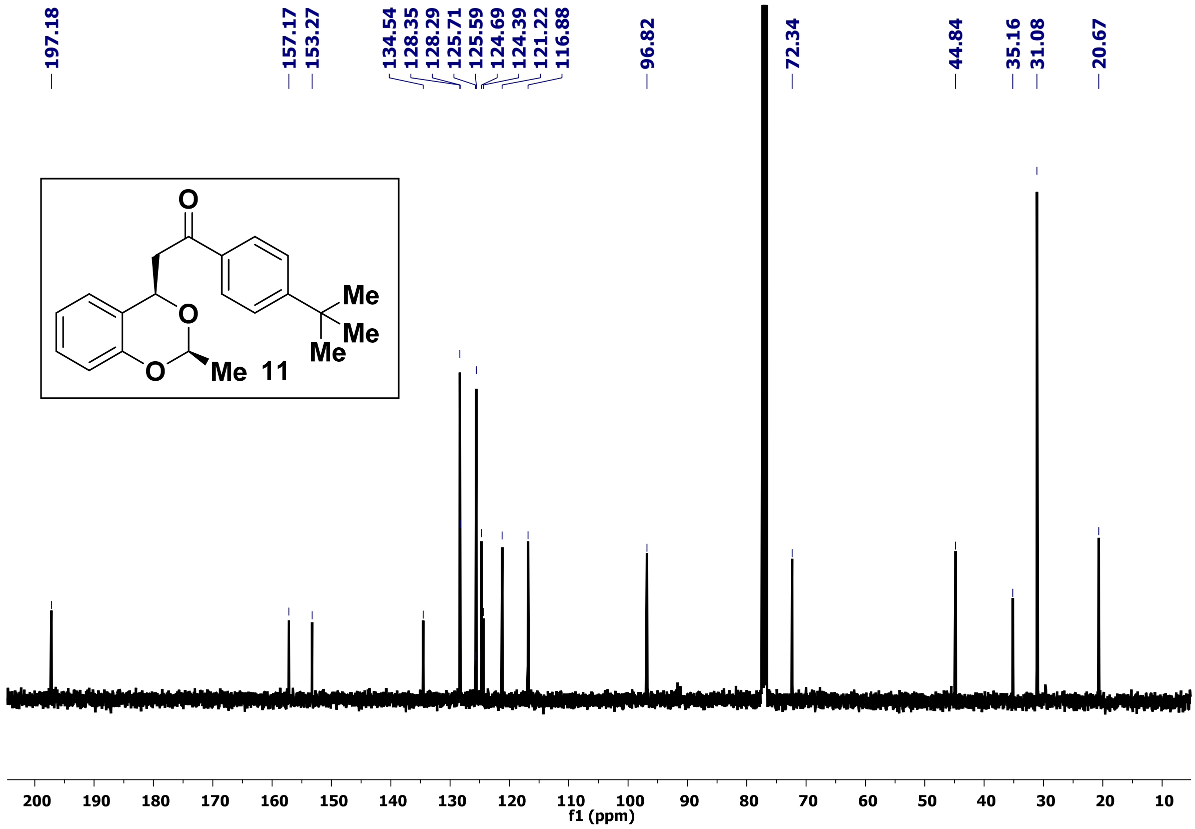
197.18
157.17
153.27
134.54
128.35
128.29
125.71
125.59
124.69
124.39
121.22
116.88
96.82
72.34
44.84
35.16
31.08
20.67
O
Me
Me
Me
O
O
Me
11
f1 (ppm)

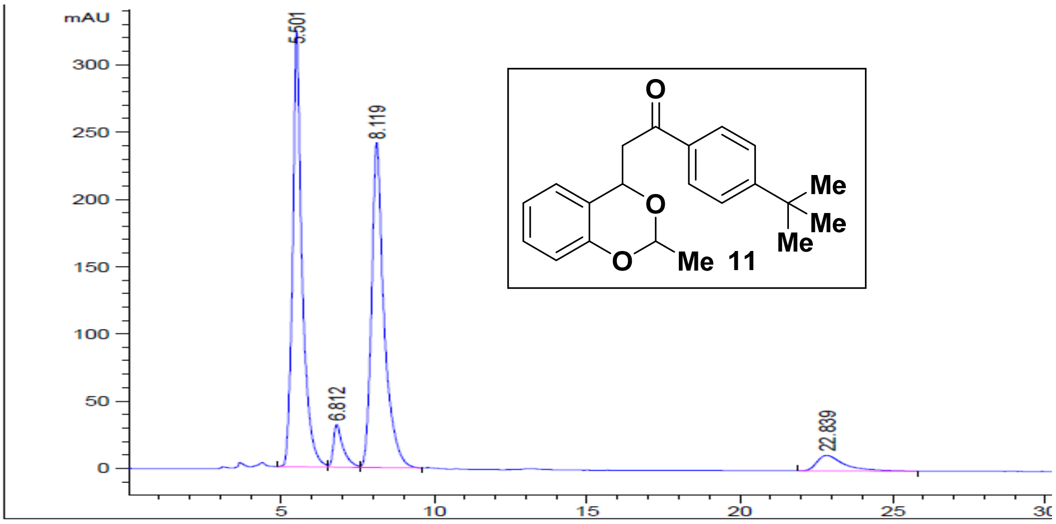

Signal 1: DAD1 A, Sig=254,4 Ref=360,100

Peak #	RetTime [min]	Type	Width [min]	Area [mAU*s]	Height [mAU]	Area %
1	5.501	BV	0.3055	7019.05859	323.03500	45.9428
2	6.812	VV	0.3190	697.98663	31.69096	4.5686
3	8.119	VB	0.4071	6850.78320	241.42389	44.8414
4	22.839	BB	0.8711	709.99231	11.45516	4.6472

Totals : 1.52778e4 607.60502

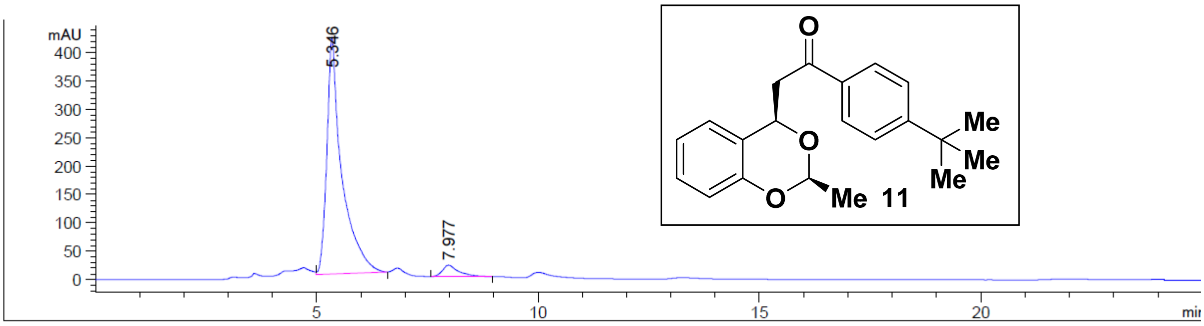

Signal 1: DAD1 A, Sig=254,4 Ref=360,100

Peak #	RetTime [min]	Type	Width [min]	Area [mAU*s]	Height [mAU]	Area %
1	5.346	VB	0.3175	9838.18750	416.54654	94.9359
2	7.977	BB	0.3660	524.79572	20.27179	5.0641

Totals : 1.03630e4 436.81833

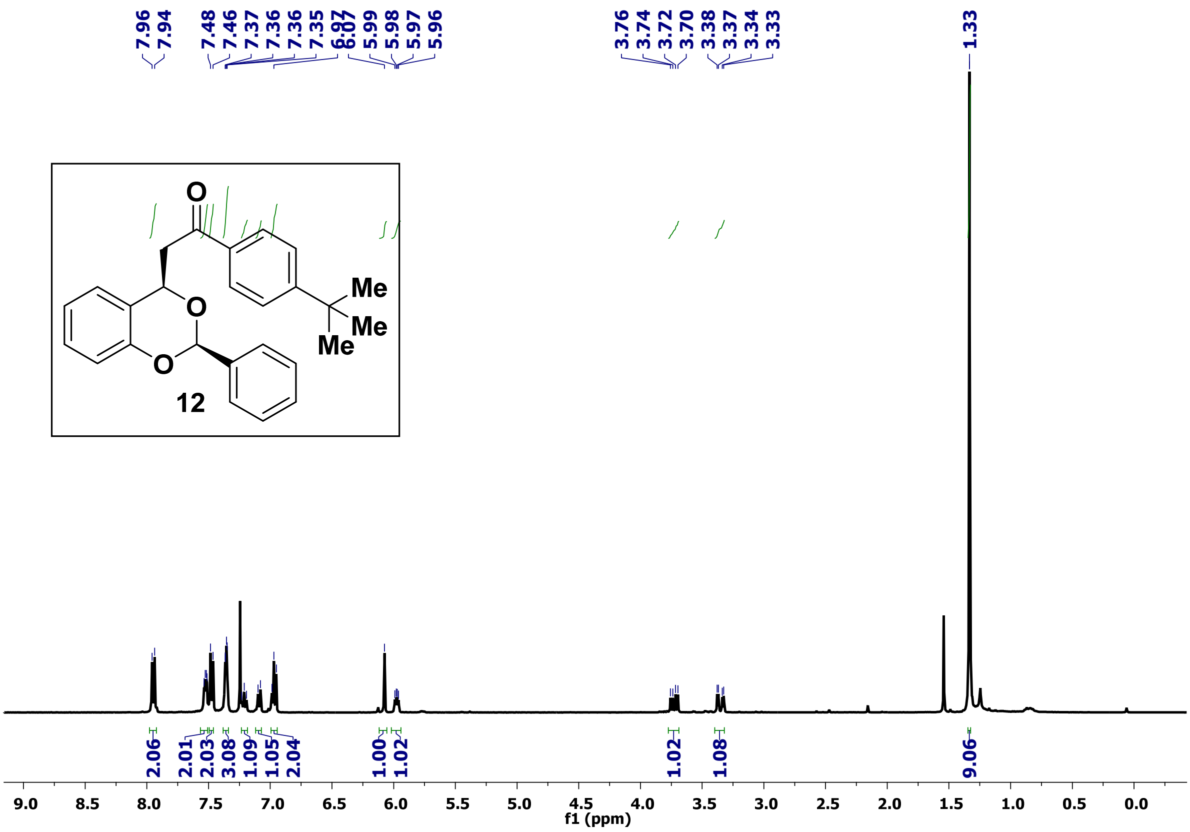
7.96
7.94
7.48
7.46
7.37
7.36
7.36
7.35
6.07
5.99
5.98
5.97
5.96
3.76
3.74
3.72
3.70
3.38
3.37
3.34
3.33
1.33
12
2.06
2.01
2.03
3.08
1.09
1.05
2.04
1.00
1.02
1.02
1.08
9.06
f1 (ppm)

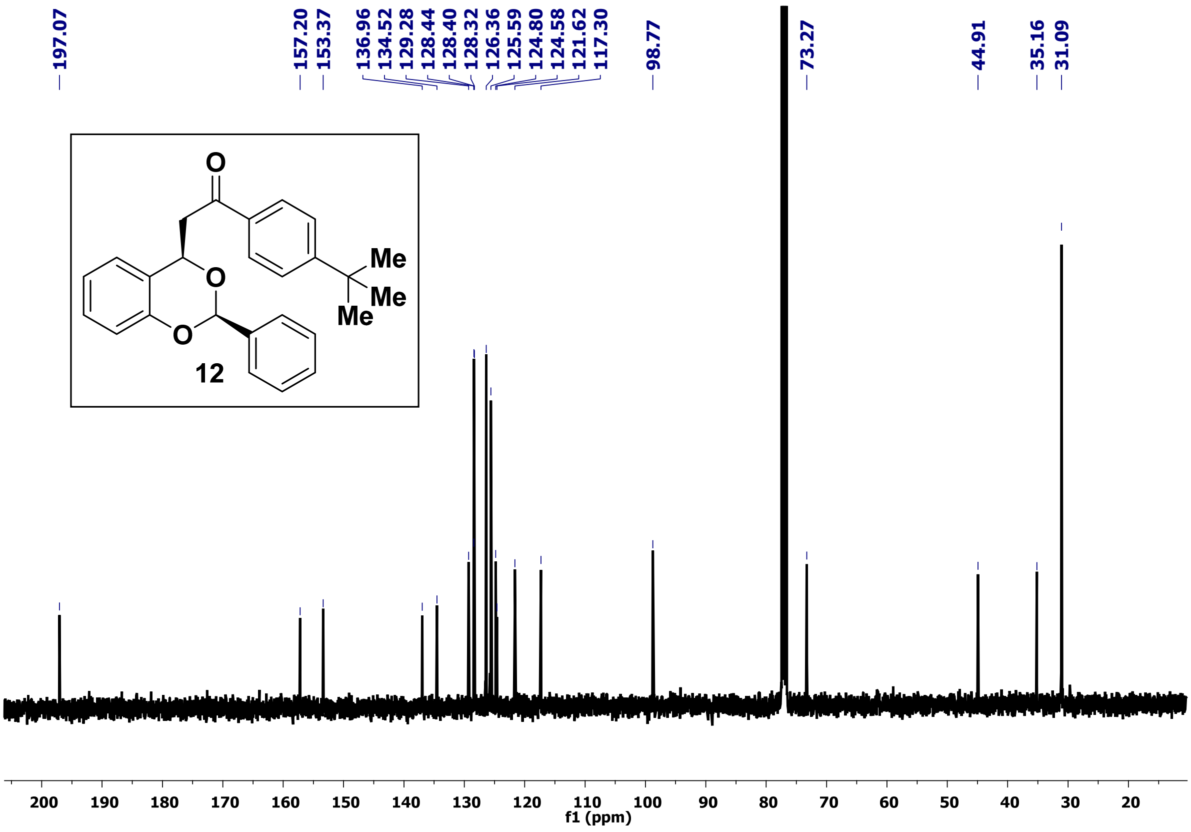
197.07
157.20
153.37
136.96
134.52
129.28
128.44
128.40
128.32
126.36
125.59
124.80
124.58
121.62
117.30
98.77
73.27
44.91
35.16
31.09
12
f1 (ppm)

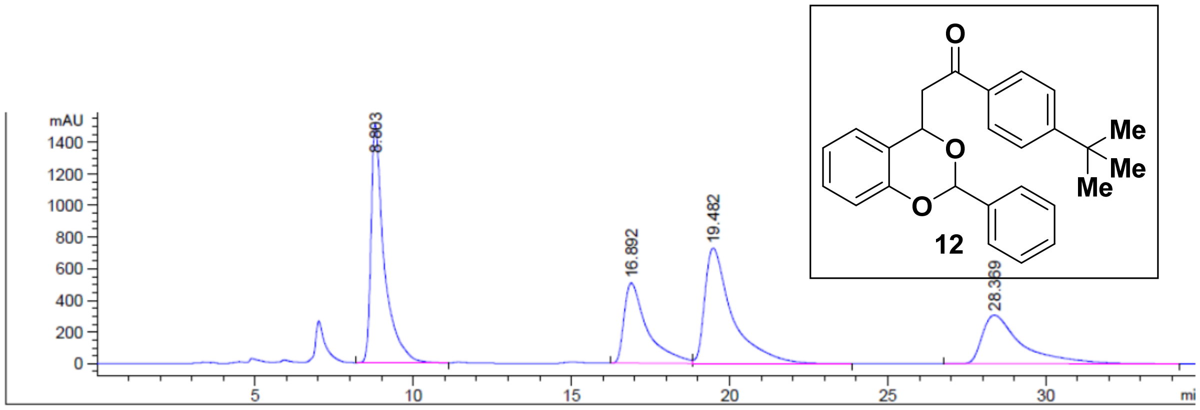

Signal 1: DAD1 A, Sig=254,4 Ref=360,100

Peak #	RetTime [min]	Type	Width [min]	Area [mAU*s]	Height [mAU]	Area %
1	8.803	BB	0.4160	4.38263e4	1512.90625	31.2699
2	16.892	BV	0.7418	2.58406e4	507.70206	18.4372
3	19.482	VB	0.8745	4.40048e4	730.80280	31.3973
4	28.369	BV	1.2576	2.64833e4	307.26086	18.8957

Totals : 1.40155e5 3058.67197

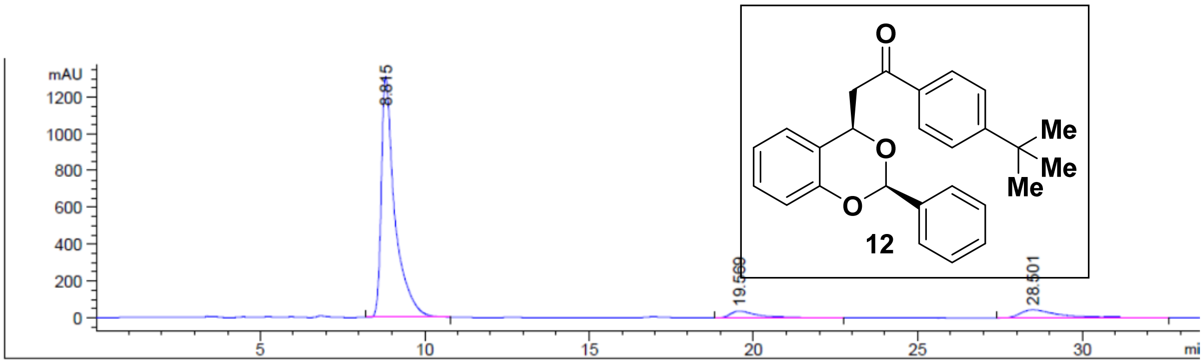

1	8.815	BB	0.4110	3.73538e4	1308.63940	86.4209
2	19.569	BB	0.8308	2158.76123	37.21481	4.9945
3	28.501	BB	1.1703	3710.55273	44.42961	8.5847

Totals : 4.32231e4 1390.28382

checkCIF/PLATON report

You have not supplied any structure factors. As a result the full set of tests cannot be run.

THIS REPORT IS FOR GUIDANCE ONLY. IF USED AS PART OF A REVIEW PROCEDURE FOR PUBLICATION, IT SHOULD NOT REPLACE THE EXPERTISE OF AN EXPERIENCED CRYSTALLOGRAPHIC REFEREE.

No syntax errors found. CIF dictionary Interpreting this report

Datablock: C1_a

```
Bond precision:  C-C = 0.0036 A          Wavelength=0.71073

Cell:            a=13.0121(10)     b=5.2339(4)     c=19.8200(16)
                 alpha=90          beta=90         gamma=90
Temperature:     142 K

                Calculated                Reported
Volume          1349.82(18)               1349.82(18)
Space group     P 21 21 21                P 21 21 21
Hall group      P 2ac 2ab                 P 2ac 2ab
Moiety formula  C15 H12 B Cl O3           ?
Sum formula     C15 H12 B Cl O3           C15 H12 B Cl O3
Mr              286.51                    286.51
Dx,g cm-3       1.410                     1.410
Z               4                         4
Mu (mm-1)       0.285                     0.285
F000            592.0                     592.0
F000'           592.86
h,k,lmax        17,7,26                   17,7,26
Nref            3478[ 2034]               3472
Tmin,Tmax       0.960,0.975
Tmin'           0.958

Correction method= Not given

Data completeness= 1.71/1.00      Theta(max)= 28.702

R(reflections)= 0.0394( 2888)     wR2(reflections)= 0.1266( 3472)

S = 0.969                  Npar= 182
```

The following ALERTS were generated. Each ALERT has the format
test-name_ALERT_alert-type_alert-level.
Click on the hyperlinks for more details of the test.

Alert level G

```
PLAT007_ALERT_5_G Number of Unrefined Donor-H Atoms ..............          1 Report
PLAT720_ALERT_4_G Number of Unusual/Non-Standard Labels ..........          5 Note
PLAT791_ALERT_4_G The Model has Chirality at C9      (Chiral SPGR)          R Verify
```

```
   0 ALERT level A = Most likely a serious problem - resolve or explain
   0 ALERT level B = A potentially serious problem, consider carefully
   0 ALERT level C = Check. Ensure it is not caused by an omission or oversight
   3 ALERT level G = General information/check it is not something unexpected

   0 ALERT type 1 CIF construction/syntax error, inconsistent or missing data
   0 ALERT type 2 Indicator that the structure model may be wrong or deficient
   0 ALERT type 3 Indicator that the structure quality may be low
   2 ALERT type 4 Improvement, methodology, query or suggestion
   1 ALERT type 5 Informative message, check
```

It is advisable to attempt to resolve as many as possible of the alerts in all categories. Often the minor alerts point to easily fixed oversights, errors and omissions in your CIF or refinement strategy, so attention to these fine details can be worthwhile. In order to resolve some of the more serious problems it may be necessary to carry out additional measurements or structure refinements. However, the purpose of your study may justify the reported deviations and the more serious of these should normally be commented upon in the discussion or experimental section of a paper or in the "special_details" fields of the CIF. checkCIF was carefully designed to identify outliers and unusual parameters, but every test has its limitations and alerts that are not important in a particular case may appear. Conversely, the absence of alerts does not guarantee there are no aspects of the results needing attention. It is up to the individual to critically assess their own results and, if necessary, seek expert advice.

Publication of your CIF in IUCr journals

A basic structural check has been run on your CIF. These basic checks will be run on all CIFs submitted for publication in IUCr journals (*Acta Crystallographica*, *Journal of Applied Crystallography*, *Journal of Synchrotron Radiation*); however, if you intend to submit to *Acta Crystallographica Section C* or *E* or *IUCrData*, you should make sure that full publication checks are run on the final version of your CIF prior to submission.

Publication of your CIF in other journals

Please refer to the *Notes for Authors* of the relevant journal for any special instructions relating to CIF submission.

PLATON version of 06/05/2016; check.def file version of 05/05/2016

Datablock C1_a - ellipsoid plot

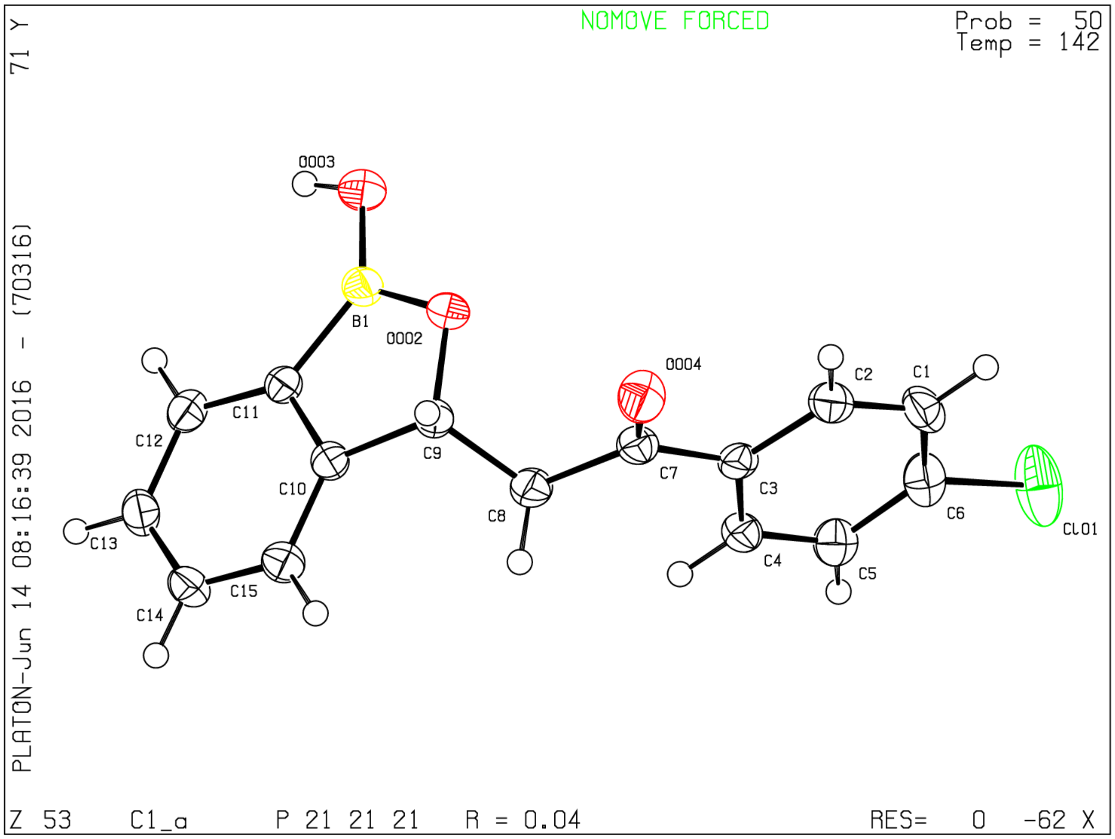

Supplement: Supplementary file 1 [file SC-008-C6SC04522G-s001.pdf]
